# Effect of Motor Skill Training in Functional Activities vs Strength and Flexibility Exercise on Function in People With Chronic Low Back Pain: A Randomized Clinical Trial

**DOI:** 10.1001/jamaneurol.2020.4821

**Published:** 2020-12-28

**Authors:** Linda R. van Dillen, Vanessa M. Lanier, Karen Steger-May, Michael Wallendorf, Barbara J. Norton, Jesse M. Civello, Sylvia L. Czuppon, Sara J. Francois, Kristen Roles, Catherine E. Lang

**Affiliations:** 1 Program in Physical Therapy, Washington University School of Medicine in St Louis, St Louis, Missouri; 2 Department of Orthopaedic Surgery, Washington University School of Medicine in St Louis, St Louis, Missouri; 3 Division of Biostatistics, Washington University School of Medicine in St Louis, St Louis, Missouri; 4 Department of Neurology, Washington University School of Medicine in St Louis, St Louis, Missouri; 5 Program in Occupational Therapy, Washington University School of Medicine in St Louis, St Louis, Missouri

## Abstract

**Importance:**

Chronic low back pain (LBP) is the most prevalent chronic pain in adults, and there is no optimal nonpharmacologic management. Exercise is recommended, but no specific exercise-based treatment has been found to be most effective.

**Objective:**

To determine whether an exercise-based treatment of person-specific motor skill training (MST) in performance of functional activities is more effective in improving function than strength and flexibility exercise (SFE) immediately, 6 months, and 12 months following treatment. The effect of booster treatments 6 months following treatment also was examined.

**Design, Setting, and Participants:**

In this single-blind, randomized clinical trial of people with chronic, nonspecific LBP with 12-month follow-up, recruitment spanned December 2013 to August 2016 (final follow-up, November 2017), and testing and treatment were performed at an academic medical center. Recruitment was conducted by way of flyers, physician and physical therapy offices, advertisements, and media interviews at Washington University in St Louis, Missouri. Of 1595 adults screened for eligibility, 1301 did not meet the inclusion criteria and 140 could not be scheduled for the first visit. A total of 154 people with at least 12 months of chronic, nonspecific LBP, aged 18 to 60 years, with modified Oswestry Disability Questionnaire (MODQ) score of at least 20% were randomized to either MST or SFE. Data were analyzed between December 1, 2017, and October 6, 2020.

**Interventions:**

Participants received 6 weekly 1-hour sessions of MST in functional activity performance or SFE of the trunk and lower limbs. Half of the participants in each group received up to 3 booster treatments 6 months following treatment.

**Main Outcomes and Measures:**

The primary outcome was the modified Oswestry Disability Questionnaire (MODQ) score (0%-100%) evaluated immediately, 6 months, and 12 months following treatment.

**Results:**

A total of 149 participants (91 women; mean [SD] age, 42.5 [11.7] years) received some treatment and were included in the intention-to-treat analysis. Following treatment, MODQ scores were lower for MST than SFE by 7.9 (95% CI, 4.7 to 11.0; *P* < .001). During the follow-up phase, the MST group maintained lower MODQ scores than the SFE group, 5.6 lower at 6 months (95% CI, 2.1 to 9.1) and 5.7 lower at 12 months (95% CI, 2.2 to 9.1). Booster sessions did not change MODQ scores in either treatment.

**Conclusions and Relevance:**

People with chronic LBP who received MST had greater short-term and long-term improvements in function than those who received SFE. Person-specific MST in functional activities limited owing to LBP should be considered in the treatment of people with chronic LBP.

**Trial Registration:**

ClinicalTrials.gov Identifier: NCT02027623

## Key Points

**Question:**

Does person-specific motor skill training in functional activities result in better short-term and long-term outcomes than strength and flexibility exercise in people with chronic, nonspecific low back pain?

**Findings:**

In this randomized clinical trial of 149 participants, motor skill training reduced disability (modified Oswestry Disability Questionnaire scores) more than strength and flexibility exercise by 7.9 points after treatment, 5.6 points 6 months after treatment, and 5.7 points 12 months after treatment, all clinically important changes from baseline and significant differences between treatment groups.

**Meaning:**

Person-specific motor skill training in functional activities limited because of low back pain should be considered to improve limited function in people with chronic low back pain.

## Teaser

This randomized clinical trial investigates whether an exercise-based treatment of person-specific motor skill training (MST) in performance of functional activities is more effective in improving function than strength and flexibility exercise immediately, 6 months, and 12 months following treatment.

## Introduction

Chronic low back pain (LBP) is the most prevalent type of chronic pain in adults,^1^ and there is no clearly optimal method of management. Exercise is an effective, nonpharmacologic treatment for chronic LBP,^2,3,4,5^ and most clinical practice guidelines recommend exercise as first-line treatment for chronic LBP.^4,6,7^ However, there is limited evidence about (1) which exercise is best^5,8,9,10^ and (2) the long-term effects of different exercise-based treatments.^2,4^

Difficulty performing daily functional activities is the primary reason that people with chronic LBP seek health care.^11,12,13^ People with spinal pain, including chronic LBP, report more pain and limitations in simple movements and complex functional activities than people with other medical conditions.^14^ Given the large detrimental effect of LBP on function, a logical form of exercise-based treatment is person-specific training to improve performance of functional activities. The goal of training would be to replace long-standing, pain-provoking movements and alignments with pain-free versions. The training should be (1) based on the person’s specific clinical presentation and limitations and (2) reinforced with repeated performance of functional activities across the day to facilitate learning.

The potential importance of training people in functional activities limited because of LBP comes from a trial comparing 2 exercise-based treatments.^15^ In the trial, both treatment conditions included 2 exercise-based components, (1) traditional therapeutic exercise (eg, abdominal strengthening) and (2) training to change functional activity performance (eg, reducing the initial movement of the lumbar spine when picking up an object). Both groups demonstrated clinically important improvement^16^ in short-term and long-term outcomes. However, adherence to functional activity training and not traditional exercise had a unique, independent effect on outcomes. Such findings suggested that the functional activity training was key to short-term and long-term improvement. However, one limitation was that all people performed both traditional exercise and functional activity training. To understand the independent effects of the 2 types of exercise, this trial compared the efficacy of a treatment of strength and flexibility exercise (SFE), a commonly prescribed treatment for chronic LBP, with person-specific training in functional activities. To improve the protocol from the prior study, the training was based on principles that facilitate learning new motor skills, hereinafter referred to as motor skill training (MST). To direct the person-specific aspect of the MST, we classified the person’s LBP condition.^17,18^ Our primary goal was to evaluate improvement in LBP-related functional limitation immediately and at 6 and 12 months after treatment. A secondary goal was to determine whether we could prevent decline in outcomes after 6 months with booster treatments.

## Methods

### Participants

People included were (1) between age 18 and 60 years, (2) had chronic LBP for at least 12 months, (3) currently experiencing LBP but not in an acute flare-up,^19^ (4) with a modified Oswestry Disability Questionnaire (MODQ) score of at least 20%, (5) with at least 3 functional activities limited due to LBP, (6) who could stand and walk without assistance, and (7) who could understand and read English and understand and sign a consent form. People were excluded if they had any structural spinal deformity, osteoporosis, ankylosing spondylitis, rheumatoid arthritis, symptomatic disc herniation, or spondylolisthesis. They also were excluded if they had a history of spinal fracture, surgery, neurologic disease requiring hospitalization, LBP owing to trauma, or unresolved cancer. Other exclusion criteria were body mass index greater than 30 (calculated as weight in kilograms divided by height in meters squared); spinal tumor or infection, frank neurological loss, pain, or paresthesia below the knee; active treatment for cancer; LBP etiology other than the lumbar spine; pregnancy; LBP-related worker’s compensation, disability, or litigation; or inability to classify the LBP condition.

Recruitment was by way of flyers placed in the community and physician offices and advertisements and interviews through local media and clinics in the region. Recruitment spanned December 2013 to August 2016. Final follow-up outcomes were obtained in November 2017.

### Design

The study was a 2-treatment group, 1-center, prospective, single blind, randomized clinical trial. Testing was conducted in the Movement Science Research Center at Washington University in St Louis, Missouri. Initially, a standardized examination was performed by a trained assessor to classify the person’s LBP.^20,21,22,23^ Classification was based on the person’s altered lumbar movements and alignments and pain reports during clinical tests and was used to design person-specific treatment in the MST condition. At enrollment, participants were randomized into 1 of 4 groups (ie, MST with no booster, MST plus booster, SFE with no booster, or SFE plus booster) with randomization sequences generated a priori by the study statistician using a formal probability model, a 1:1:1:1 allocation ratio, and a block size of 16. Randomization was stratified by LBP classification (ie, rotation, extension, flexion, rotation with flexion, or rotation with extension) and elicited from the data capture system. Treatment duration was 6 weeks for 1 hour per week. At 6 months after treatment, participants randomized to treatment plus booster received up to 3 booster treatments in their initial treatment assignment. The number of booster treatments was based on the participant’s ability to perform his home program without coaching.^24^ Data collected included self-report and laboratory measures. All data were collected at baseline and both immediately and 6 months after the end of treatment. Additionally, a subset of self-report data was collected monthly for 12 months via electronic mail.

The trial ended on attainment of 12-month outcomes. In November 2013, trial exclusion criteria were changed to exclude people with fibromyalgia, Marfan syndrome, and Graves disease. These were excluded to avoid enrolling people with conditions characterized by diffuse pain owing to a systemic disorder. In October 2015, trial exclusion criteria were changed to exclude people with a history of disc herniation only if they had current symptoms below the knee, indicating the herniation was contributing to the current clinical presentation. The trial design had no other changes. The study protocol was approved by the human research protection office at Washington University School of Medicine. Written informed consent was obtained from all participants.

### Interventions

Treatment was provided at a university-based outpatient physical therapy clinic. Each therapist (n = 8) was given 8 hours of training in 1 of 2 treatment conditions by 2 of the authors (L.V.D. and C.E.L.). Initially and annually, each therapist was required to pass (score ≥90%) a written and practical examination. The SFE therapists were masked to LBP classification. Therapists and participants were not masked to treatment assignment. In both conditions, education and 1 of 2 types of exercise was provided; MST or SFE. Once educational principles were mastered, treatment focused solely on the exercise component. Progression was based on the participant’s ability to perform each treatment item independently.^24^ A home program was prescribed and progressed across the treatment phase. Participants were instructed to receive no other treatments for LBP during the treatment phase. At the final clinic visit, participants were instructed to continue the home program.

Motor skill training involved supervised, massed practice of challenging functional activities that were difficult to perform because of LBP.^25,26^ Participants assisted in choosing activities. Difficulty was graded continuously within and across visits to match motor capabilities. Extrinsic feedback was minimized during practice and removed as quickly as possible. Practice was based on the (1) participant’s ability to perform the activity and (2) level of challenge the participant was faced with daily. Emphasis was on changing the altered movements and alignments relevant to the person-specific classification^17,18^ during activities to reduce LBP. The primary treatment principles were to teach the participant to (1) move the lumbar spine later and reduce the amount of lumbar spine movement(s) related to their LBP classification (eg, flexion), (2) increase use of other joints (eg, hips), and (3) avoid end-range positioning of the lumbar spine in specific direction(s) related to the patient’s LBP classification. Participants were given cues for using trunk muscles needed to facilitate the correct movement or alignment during activities. The training focused on problem solving by the participant to learn to perform the activities without increased LBP.

Strength and flexibility exercises focused on improving the strength of all of the trunk muscles and improving trunk and lower limb flexibility in all planes. All exercises were prescribed and progressed based on American College of Sports Medicine guidelines.^27^ A change in LBP (increase or decrease) during exercise was not used to guide prescription or progression. The full trial protocol including a detailed description of each treatment condition is in Supplement 1 and in the eMethods in Supplement 2.

### Outcomes

Outcomes were measured with patient-reported data obtained from validated questionnaires. All patient-reported data were collected using Research Electronic Data Capture.^28,29^ The primary outcome was the MODQ (0%-100%), a validated measure of LBP-related functional limitation where higher scores indicate greater limitation.^30^ Secondary outcomes included (1) the Numeric Pain Rating Scale for average and worst LBP in prior 7 days^31^; (2) number, length, and intensity of acute flare-ups of LBP in prior 6 months^19,32^; (3) current LBP medication use; (4) 36-Item Short Form Health Survey Physical and Mental Component Summary scores^33,34,35^; (5) absenteeism from usual activities^36^; (6) presenteeism related to work impairment, work output, and work absenteeism^37,38^; (7) care seeking for LBP; (8) equipment use for LBP; (9) adherence^15,39^; (10) fear-avoidance beliefs^40,41^; and (11) satisfaction with care.^42^ Testers were masked to treatment assignment throughout the study.

### Power and Statistical Analysis

The power analysis for detecting a minimal clinically important difference of 6 on the MODQ^43^ based on hierarchical multiple regression indicated that 154 participants needed to be enrolled for 80% power, assuming 20% attrition, α = .05, and 2-tailed tests. The original protocol called for analysis with hierarchical linear modeling to model repeated measures without requiring time between samples to be constant. However, the times between samples were sufficiently consistent to allow mixed-effects repeated-measures analysis.

For MODQ, mixed random-effects repeated-measures analyses were conducted separately on each phase (treatment and follow-up), with participant within treatment as a random effect and a first-order autoregressive covariance structure to account for correlation between points. The baseline MODQ score was used as a covariate to control for baseline participant differences. Treatment, time, and time by treatment interaction were included in the model as fixed effects. Booster treatments after follow-up month 6 did not affect subsequent MODQ scores (eTable in Supplement 2); treatment estimates from data after month 6 were created from combined booster and no booster groups within treatment. The analyses were intention to treat where all randomized participants who started the allocated intervention were included. Some data were missing at random; however, 68 MST and 67 SFE participants provided at least 6 of 7 MODQ scores during the treatment phase. No missing data imputation was performed.^44^ Mean estimates for single points were model-based least square (LS) means (unless otherwise noted). The standardized mean difference also was calculated for the treatment and follow-up phase.

## Results

### Participants

**Figure 1.**
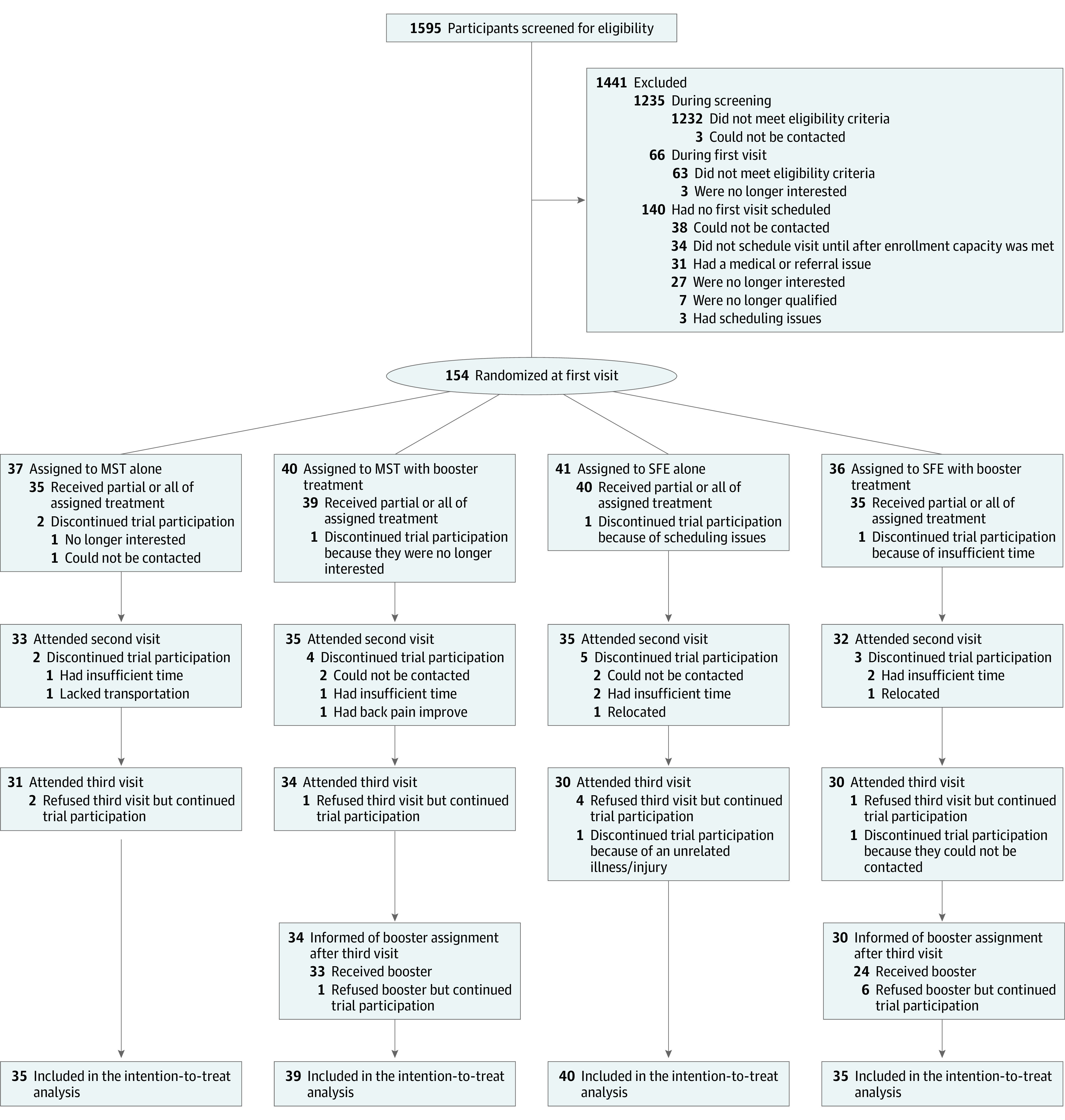
Enrollment, Randomization, Treatment, and Follow-up Participant randomization was determined at laboratory visit 1 after the participant completed the clinical examination. Each participant enrolled was randomized to 1 of 4 groups: motor skill training without booster (MST-B), MST with booster (MST+B), strength and flexibility exercise without booster (SFE-B), or SFE with booster (SFE+B). Participants were informed of their assigned treatment condition (MST or SFE) after laboratory visit 1 (baseline visit) and of their booster randomization after laboratory visit 3 (6-month follow-up visit).

One hundred fifty-four participants were enrolled (Figure 1). Fourteen percent of participants (21 of 154) withdrew across the study period. Five withdrawals were prior to treatment (3 in the MST arm and 2 in the SFE arm; LBP classification: 4 rotation and 1 rotation with extension; 4 women and 1 man) and were not included in the data analyses. Sixteen withdrawals were during or after treatment (MST = 6 and SFE = 10). Baseline characteristics of participants were not significantly different between the 2 treatment groups (Table 1).

**Table 1.** Characteristics for Enrolled Participants Who Started Treatment

Characteristic	No. (%)			*P* value^a^
	Complete sample (N = 149)	Treatment group		
		Motor skill training (n = 74)	Strength and flexibility exercise (n = 75)	
Demographic variables				
Female^b^	91 (61)	50 (68)	41 (55)	.11
Age, mean (SD), y	42.5 (11.7)	42.4 (11.8)	42.6 (11.7)	.90
White race/ethnicity^c^	115 (77)	58 (78)	57 (76)	.73
BMI, mean (SD)	25.7 (3.2)	25.3 (3.2)	26.1 (3.1)	.16
Married or living with significant other	100 (67)	45 (61)	55 (73)	.10
Completed at least some college	139 (93)	67 (91)	72 (96)	.21^d^
Employment situation^c^				
Working full time	101 (68)	50 (68)	51 (68)	.32^d^
Working part time	28 (19)	15 (20)	13 (17)	
Student (not working)	5 (3)	4 (5)	1 (1)	
Other employment status	15 (10)	5 (7)	10 (13)	
LBP-related variables				
LBP classification				
Rotation	82 (55)	42 (57)	40 (53)	.71^d^
Rotation with flexion	4 (3)	1 (1)	3 (4)	
Rotation with extension	63 (42)	31 (42)	32 (43)	
Duration of LBP, median (IQR), y	7.0 (12.0)	7.0 (17.0)	7.0 (11.0)	.98^d^
Symptoms only in back^e^	112 (76)	58 (79)	54 (72)	.29
Medication use^f^				
Taking nonprescription medication	90 (60)	46 (31)	43 (29)	.63
Taking prescription medication	28 (19)	13 (46)	15 (54)	>.99
Nonsteroidal anti-inflammatory	8 (5)	3 (4)	5 (7)	.70^d^
Opioid or opiate pain reliever	4 (3)	1 (1)	3 (4)	.60^d^
Prescription acetaminophen	22 (15)	11 (15)	11 (15)	>.99^d^
Skeletal muscle relaxant	11 (7)	4 (5)	7 (9)	.50^d^
Antidepressants	1 (0.7)	1 (1)	0	.50^d^
Glucocorticoids	1 (0.7)	0	1 (1)	>.99^d^

Abbreviations: BMI, body mass index (calculated as weight in kilograms divided by height in meters squared); IQR, interquartile range; LBP, low back pain.

^a^ Unless otherwise noted, *P* value compares treatment groups by unpaired *t* test (for continuous variables) or χ^2^ test (for categorical variables).

^b^ Participant-reported gender identity.

^c^ Data captured by participant report with several “check all that apply” categories. White race/ethnicity includes a multiracial identification that includes white. Race/ethnicity category options are those required for reporting to the funding agency. Employment includes multiemployment identification, where a single category is assigned in the listed order of priority.

^d^ *P* value compares treatment groups by Wilcoxon test (for nonnormal continuous variables) or Fisher exact test (for categorical variables with small cell sizes).

^e^ Data missing for 1 MST participant. Operational definition for location “only in back” is symptoms in region from T12 to gluteal fold.

^f^ Participants could be taking more than 1 medication.

### Outcomes

#### Primary Efficacy End Point: MODQ

**Figure 2.**
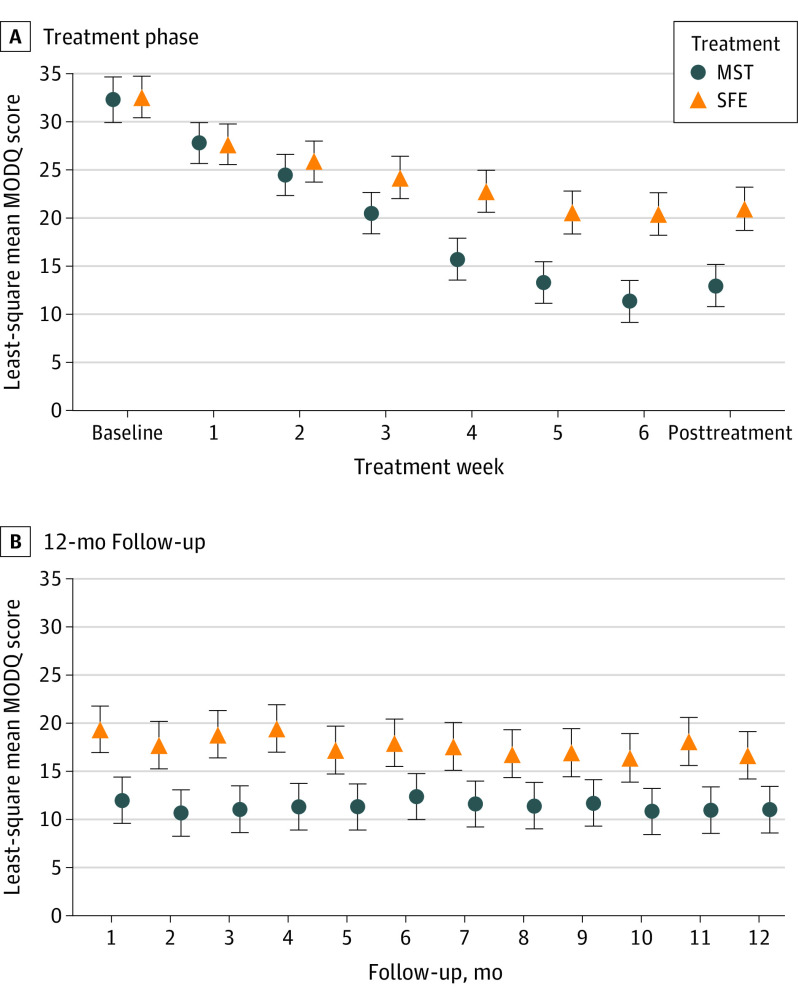
Modified Oswestry Disability Questionnaire Scores (MODQ) Over Time A, Least-square mean MODQ scores (with 95% confidence interval bars) over time during the treatment phase for the motor skill training (MST) group and the strength and flexibility exercise (SFE) group. Scores on the MODQ range from 0% to 100%; 100% represents the highest limitation. The MST group improved to a greater degree than the SFE group. B, Least-square mean MODQ scores (with 95% confidence interval bars) over time in the 12 month follow-up phase for the 2 treatment conditions. Both groups maintained improvements obtained with treatment. The mean MODQ score over the 12 month follow-up phase for the MST group was lower than for the SFE group.

During the treatment phase, MST reduced MODQ scores^30^ more than SFE. At the posttreatment stage, MST was lower than SFE by 7.9 (95% CI, 4.7-11.0; *P* < .001) (Figure 2A, Table 2). During the follow-up phase, the MST group maintained lower levels of MODQ scores than the SFE group, 5.6 lower at 6 months (95% CI, 2.1-9.1) and 5.7 lower at 12 months (95% CI, 2.2-9.1) (Figure 2B). Booster sessions after follow-up month 6 did not change MODQ scores in either treatment (eTable in Supplement 2). The standardized mean difference (SMD) after treatment was large (SMD, 0.85; 95% CI, 0.51-1.19) and after 12 months was moderate (0.56; 95% CI, 0.22-0.90).^45^

**Table 2.** Modified Oswestry Disability Questionnaire Score Differences at Baseline, Posttreatment, Follow-up Month 6, and Follow-up Month 12^a^

Time	Means (SD)^b^		Mean difference (95% CI)^c^	*P* value
	Motor skill training	Strength and flexibility exercise		
Baseline	32.3 (10.2)	32.6 (9.4)	NA	NA
Posttreatment	12.8 (10.7)	21.2 (10.7)	7.9 (4.7-11.0)	<.001
Follow-up				
Month 6	12.0 (12.6)	18.2 (10.5)	5.6 (2.1-9.1)	.002
Month 12	10.8 (11.3)	16.7 (11.3)	5.7 (2.2-9.1)	.001

Abbreviation: NA, not applicable.

^a^ Scores on the modified Oswestry Disability Questionnaire range from 0% to 100%; 100% represents the highest level of limitation.

^b^ Sample means and standard deviations.

^c^ Model-based contrasts.

#### Secondary Efficacy End Points

Many of the secondary outcomes also showed statistically significant differences in favor of MST vs SFE (Table 3). Posttreatment MST resulted in higher satisfaction with care, greater improvement in average and worst LBP and physical function, less LBP-related medication use, less absenteeism from usual activities, and lower work-related fear avoidance beliefs compared with SFE. Six months after treatment, MST also resulted in fewer and shorter acute LBP flare-ups and greater adherence compared with SFE. Finally, at 12 months, MST yielded lower average and worst LBP than SFE. However, benefits of MST vs SFE did not occur at any point for intensity of acute flare-ups, mental function, work impairment and work absenteeism, physical function–related fear avoidance beliefs, equipment use, or care seeking for LBP.

**Table 3.** Secondary Outcome Treatment Least Square Mean Differences and Odds Ratios at Posttreatment, Follow-up Month 6, and Follow-up Month 12

Variable	Single time points	Sample means (SD)		LS mean difference: SFE − MST (95% CI)
		Motor skill training (n = 74)	Strength and flexibility exercise (n = 75)	
Numeric Pain Rating Scale^a^				
Average	Baseline	4.7 (1.9)	4.7 (1.5)	NA
	Posttreatment	1.4 (1.1)	2.1 (1.2)	0.8 (0.3 to 1.2)
	Follow-up mo 6	2.0 (1.8)	2.6 (1.8)	0.5 (−0.1 to 1.1)
	Follow-up mo 12	1.8 (1.9)	2.6 (2.0)	0.8 (0.2 to 1.4)
Worst	Baseline	6.3 (2.0)	6.9 (1.6)	NA
	Posttreatment	2.7 (1.7)	4.0 (1.9)	1.0 (0.4 to 1.7)
	Follow-up mo 6	2.9 (2.2)	3.8 (2.2)	0.6 (−0.2 to 1.3)
	Follow-up mo 12	2.8 (2.3)	3.9 (2.5)	1.0 (0.2 to 1.7)
Acute flare-ups of LBP in prior 6 mos^b^				
No.	Baseline	7.1 (7.6)	9.8 (11.9)	NA
	Follow-up mo 6	2.0 (3.3)	4.2 (8.2)	0.8 (0.1 to 1.5)
	Follow-up mo 12	1.3 (1.8)	2.0 (2.8)	0.3 (−0.2 to 0.9)
Length	Baseline	4.4 (5.4)	3.7 (6.9)	NA
	Follow-up mo 6	1.7 (2.1)	3.9 (7.0)	0.9 (0.2 to 1.8)
	Follow-up mo 12	2.0 (4.0)	2.8 (7.6)	0.3 (−0.3 to 1.0)
Intensity	Baseline	6.6 (2.5)	6.1 (2.6)	NA
	Follow-up mo 6	3.1 (3.2)	3.9 (2.9)	0.7 (−0.3 to 1.8)
	Follow-up month 12	2.6 (3.0)	3.4 (3.2)	0.7 (−0.4 to 1.8)
SF-36 Component Summary Scores^c^				
Physical	Baseline	43.2 (6.6)	40.8 (6.9)	NA
	Posttreatment	50.8 (6.5)	46.3 (7.0)	−2.9 (−5.0 to −0.9)
	Follow-up mo 6	50.9 (6.6)	47.8 (7.4)	−1.8 (−4.3 to 0.7)
	Follow-up mo 12	51.2 (8.0)	48.3 (7.4)	−2.0 (−4.5 to 0.4)
Mental	Baseline	49.2 (11.6)	52.1 (9.3)	NA
	Posttreatment	50.7 (8.6)	51.0 (11.6)	−1.3 (−4.2 to 1.6)
	Follow-up mo 6	49.6 (10.5)	50.8 (10.4)	0.1 (−3.2 to 3.5)
	Follow-up mo 12	50.4 (10.3)	50.3 (11.5)	−1.3 (−4.6 to 2.0)
Stanford Presenteeism Scale^d^				
Work Impairment Score	Baseline	20.3 (5.3)	19.9 (6.2)	NA
	Posttreatment	17.4 (4.6)	18.3 (5.2)	1.3 (−0.3 to 2.8)
	Follow-up mo 6	15.9 (5.6)	16.5 (5.3)	1.4 (−0.4 to 3.2)
	Follow-up mo 12	15.4 (5.1)	16.7 (6.0)	1.6 (−0.2 to 3.4)
Work Output Score	Baseline	87.0 (15.3)	83.4 (19.3)	NA
	Posttreatment	95.4 (7.4)	91.6 (11.4)	−1.9 (−3.6 to −0.5)
	Follow-up mo 6	95.2 (9.0)	93.1 (16.9)	−0.2 (−1.5 to 1.0)
	Follow-up mo 12	95.7 (8.6)	96.2 (6.2)	0.3 (−0.8 to 1.3)
Adherence to home program^e^	Posttreatment	83 (14)	90 (15)	6.7 (2.8 to 12.0)
	Follow-up mo 6	70 (21)	42 (31)	−40.8 (−52.9 to −27.9)
	Follow-up mo 12^f^	70 (21)	51 (35)	−19.1 (−37.9 to 0.4)
Fear-Avoidance Beliefs Questionnaire^g^				
Physical score	Baseline	14.7 (6.1)	14.1 (5.1)	NA
	Posttreatment	11.4 (5.9)	11.6 (5.3)	0.4 (−1.4 to 2.2)
	Follow-up mo 6	11.0 (5.8)	11.4 (5.5)	0.7 (−1.2 to 2.5)
Work score	Baseline	10.9 (8.4)	11.8 (9.0)	NA
	Posttreatment	7.7 (7.1)	10.9 (9.5)	2.3 (0.01 to 4.6)
	Follow-up mo 6	7.2 (8.2)	9.0 (8.7)	0.9 (−1.5 to 3.3)
Satisfaction with care^h^				
Total	Posttreatment	68.8 (0.9)	61.4 (0.9)	−7.4 (−9.8 to −4.9)
Dichotomous variables				
Current LBP medication use, No. (%)^i^	Baseline	50 (68)	47 (63)	NA
	Posttreatment	21 (30)	31 (46)	Odds ratio^j^, 2.4 (1.1 to 5.4)
	Follow-up mo 6	27 (40)	29 (47)	Odds ratio^j^, 1.3 (0.6 to 2.9)
	Follow-up mo 12	23 (34)	25 (38)	Odds ratio^j^, 1.4 (0.6 to 3.1)
Absenteeism from usual activities, No. (%)^i^	Baseline	42 (57)	47 (63)	NA
	Posttreatment	11 (16)	29 (43)	Odds ratio^j^, 4.2 (1.8 to 10.0)
	Follow-up mo 6	9 (14)	11 (18)	Odds ratio^j^, 1.4 (0.5 to 3.4)
	Follow-up mo 12	11 (16)	12 (18)	Odds ratio^j^, 1.1 (0.5 to 2.7)
Stanford Presenteeism Scale^i^				
Work absenteeism, No. (%)^i^	Baseline	19 (29)	22 (35)	NA
	Posttreatment	3 (5)	6 (10)	Odds ratio^j^, 3.7 (0.6 to 21.3)
	Follow-up month 6	3 (6)	6 (11)	Odds ratio^j^, 1.4 (0.6 to 3.5)
	Follow-up month 12	4 (7)	3 (6)	Odds ratio^j^, 1.2 (0.4 to 3.4)
Health professional care seeking for LBP, No. (%)^i^	Baseline	35 (47)	29 (39)	NA
	Posttreatment	7 (10)	6 (9)	Odds ratio^j^, 1.0 (0.3 to 3.3)
	Follow-up mo 6	13 (19)	13 (21)	Odds ratio^j^, 1.1 (0.5 to 2.5)
	Follow-up mo 12	8 (12)	11 (17)	Odds ratio^j^, 1.5 (0.5 to 4.0)
Equipment use for LBP, No. (%)^i^	Baseline	66 (89)	65 (87)	NA
	Posttreatment	62 (91)	48 (71)	Odds ratio^j^, 0.21 (0.07 to 0.58)
	Follow-up mo 6	33 (49)	29 (47)	Odds ratio^j^, 0.5 (0.2 to 1.1)
	Follow-up mo 12	28 (41)	30 (46)	Odds ratio^j^, 1.1 (0.5 to 2.2)

Abbreviations: LBP, low back pain; LS, least square; MST, motor skill training; NA, not applicable; NRS, Numeric Pain Rating Scale; SFE, strength and flexibility exercise.

^a^ Numeric Pain Rating Scale ranges from 0 to 10, with higher scores indicating more pain.

^b^ A flare-up is an increase in symptoms of at least 2 points on the NRS greater than a participant’s typical low back pain that lasts for at least 2 consecutive days. Participants provided the number in the past 6 months, the length (days), and the average pain intensity (NRS) during the flare-ups.

^c^ 36-Item Short Form Health Survey Physical and Mental Component Summary Scores are scaled and normalized to have a mean of 50 and standard deviation of 10 in the normal 1998 US population.

^d^ Stanford Presenteeism Scale Work Impairment Score ranges from 10 to 50, with 50 indicating the highest degree of impairment. The Work Output Score is the participant’s estimate of the percentage of his usual productivity level during work over the past 4 weeks (0%-100%).

^e^ Adherence to home program ranges from 0% to 100%, with higher values indicating higher adherence to treatment. Participants reported weekly adherence during the treatment phase and monthly adherence during the follow-up phase.

^f^ Estimates were based on data from nonbooster treatment groups because booster sessions affected SFE adherence after follow-up month 6. The SFE adherence increased after booster sessions.

^g^ Fear-Avoidance Beliefs Questionnaire physical activity subscale score ranges from 0 to 24 and work subscale score ranges from 0 to 42 with higher scores indicating higher fear avoidance.

^h^ Satisfaction with care ranges from 15 to 75 with higher scores indicating more satisfaction.

^i^ Sample counts.

^j^ Model-based odds ratio; SFE odds/MST odds.

### Adverse Events

#### Treatment Phase

No serious adverse events were reported. Nonserious adverse events included reports of a worsening of LBP (operational definition in the protocol) by 62 participants (107 occurrences); 2 SFE participants and 1 MST participant (4 occurrences; 4%) related the increase to treatment. All reports were resolved by the next treatment visit.

#### Follow-up Phase

No serious adverse events were related to treatment. Two serious adverse events unrelated to treatment were reported. One participant was diagnosed as having ductal carcinoma. A second participant had minimally invasive surgery for disc herniation. There were no nonserious adverse events related to treatment. Nonserious adverse events unrelated to treatment included 5 reports of lower extremity injury or pain, 1 increase in LBP as an adverse effect of medication use, 1 unexplained increase in LBP for 1 month, 2 reports of influenza, 1 diagnosis of osteoporosis, 1 diagnosis of facial palsy, and 1 pregnancy.

## Discussion

Our study provides evidence that person-specific MST in LBP-limited functional activities results in greater short-term and long-term improvements in function than traditional strength and flexibility exercise (SFE). Immediately after treatment, both the MST and SFE groups displayed clinically meaningful improvement^16,43^ in function. However, MST demonstrated almost twice the improvement in function (60% change) as SFE (35%). Most importantly, the between-group differences in MODQ scores were sustained at the 6-month and 12-month follow-up. These findings are highly relevant given the primary reason people with chronic LBP seek health care is difficulty performing functional activities.^11,12,13^ The effects also were obtained with only 6 one-hour treatments. In addition, self-reported adherence to MST was consistently high, suggesting MST provides a feasible means for self-management (Table 3).

The findings across the secondary outcomes display a pattern that also supports MST as more effective than SFE, although the mean differences tend to be small or the range of the confidence intervals are large (Table 3). Five of the 6 pain-related variables (average and worst pain, acute flare-up number and length, and medication use) favored MST to SFE at early and late points. Three of the 6 physical function-related variables (36-Item Short Form Health Survey physical function, work output, and absenteeism from usual activities) and work-related fear avoidance improved to a greater degree for MST than SFE at the earliest point. In addition, people were more satisfied and tended to adhere more to MST than SFE.

Our results suggest that the use of principles of motor learning^25,46,47,48^ to drive change in function is critical for people with chronic LBP. Instead of assuming that the benefits of traditional exercise generalize to functional activities, we provided person-specific MST directly targeting how people performed functional activities. Specifically, challenging behavioral demands were repeatedly imposed to facilitate learning to change LBP-provoking strategies used across multiple activities. The goal was to change long-standing strategies to improve the short-term and long-term course of the condition. Indeed, our results showed that the MST group displayed greater and more durable improvements in function than the SFE group. This is a major outcome because a key recommendation of clinical practice guidelines for LBP is to use treatments that increase function and discourage behaviors that contribute to persistent disability.^6,10^

Treatment guidelines for chronic, nonspecific LBP recommend exercise-based treatments as first-line care.^4,6,7,10^ However, there is no strong evidence for any specific type of exercise-based treatment.^6,7,10^ Some have suggested that classifying a person’s LBP based on relevant characteristics and providing person-specific treatment based on that classification could improve outcomes.^49,50,51^ A 2018 systematic review^52^ compared person-specific treatment targeting altered movements and alignments (as in the current study) with other treatments. The authors concluded that person-specific treatment results in greater improvement in function than other treatments in the short term and long term, but the effect sizes were small. Additionally, the conclusions were tentative because of multiple study limitations. In our high-quality trial, person-specific MST targeting LBP-limited functional activities resulted in greater improvements in function than SFE in the short term and long term with large and moderate effect sizes.

Training in functional activities has been examined as an exercise-based treatment in prior clinical trials for chronic, nonspecific LBP.^53,54,55,56,57^ A common feature across prior trials was that the training was provided in combination with other treatment components, making it impossible to determine the specific effect of training in functional activities. Given the MST condition only included training in functional activities, our findings signal the importance of directly addressing person-specific strategies used during functional activities with MST to attain large and long-lasting improvement in function. Additionally, because therapists could identify activities and scale the level of training for participants presenting with varying levels of limitations, the findings also support the use of MST in functional activities from the outset of treatment.

A secondary goal of this study was to test the efficacy of booster treatments to prevent the decline in function observed from 6 to 12 months in the prior clinical trial.^15^ On average, in the MST condition, there was no effect of booster sessions on outcomes. The lack of booster effects likely is because both MST groups (booster and no booster) maintained the gains obtained during the treatment phase and did not decline in the 6 to 12 months after treatment as in the previous trial.^15^ The lack of decline is attributed to improvements in the MST design made based on the prior clinical trial^15^ experience. It is notable that the amount of improvement in function initially attained with 6 weekly treatments (60%) and then maintained over 6 months (63%) in MST was similar to gains at the 6-month point (65%) in the prior trial. These large improvements also were achieved in a sample of people who were not experiencing an acute flare-up and had higher levels of functional limitation and LBP than in the prior trial.^15^ In the SFE condition, boosters only affected adherence; other outcomes did not differ. Specifically, adherence was maintained in the booster group but decreased in the no-booster group (eTable in Supplement 2), suggesting boosters may be important for reminding people what they should be doing.

Strengths of the study include the randomized, controlled trial design, inclusion of moderately involved people who were not experiencing an acute LBP flare-up, use of person-specific treatment, inclusion of 2 active exercise-based treatments, tracking of adherence, minimal loss to follow-up across the study period, and an intention-to-treat analysis. Therapists also were trained in standardized procedures; knowledge and performance of therapists was examined regularly^24^; standardized procedures for progression of treatment were used^24^; and medical records were audited for treatment fidelity regularly by masked assessors.

### Limitations

A limitation of our study is that our findings may not be generalizable to people with anatomically specific LBP conditions, substantial behavioral or psychological comorbidities, symptoms below the knee, or high levels of pain and functional limitation. In addition, we cannot know how well people who have lower levels of education or employment than our participants would perform or whether similar outcomes would be attained if therapists did not have the specific training, testing, monitoring, and feedback provided in our study. Finally, we did not include an attention control group.

## Conclusions

People with chronic LBP who received person-specific MST to change functional activity performance displayed greater short-term and long-term improvements in function than those who received SFE. A number of pain, physical function, and psychological outcomes also improved to a greater degree in the MST group compared with the SFE group. These findings suggest that a priority of treatment for people with chronic LBP is to provide person-specific, challenging practice that promotes learning new strategies of movement and alignment during LBP-limited functional activities. Use of MST appeared to (1) result in improved short-term but more importantly long-term outcomes with only 6 one-hour treatments, (2) promote better adherence to training for a prolonged period, and (3) enable a person to practice the activities across the day, thus providing a means of self-management. Such benefits could be key in a condition typically characterized by a clinical course of recurrent, fluctuating, or persistent functional limitation and pain.

## Notes

Supplement 1. Trial Protocol.

Supplement 2. **eTable.** Primary and Secondary Outcome Least square (LS) Mean Differences and Odds Ratios Pre-Booster (Months 1-6) and Post-Booster (Months 7-12) for People in the Booster Treatment Condition and People in the No Booster Treatment Condition **eMethods.** Detailed Descriptions of Motor Skill Training and Strength and Flexibility Exercise Treatment Conditions **eReferences.**

Supplement 3. Data Sharing Statement
