# Supplementary material for: Effect of Motor Skill Training in Functional Activities vs Strength and Flexibility Exercise on Function in People With Chronic Low Back Pain: A Randomized Clinical Trial
Source: JAMA Neurol. 2020 Dec 28;78(4):1–11. doi: 10.1001/jamaneurol.2020.4821 (PMC7770617; doi:10.1001/jamaneurol.2020.4821)
Supplement: Supplement 1. — Trial Protocol. [file jamaneurol-e204821-s001.pdf]

This supplement contains the following items:

1. Original protocol, original statistical analysis plan
2. Final protocol, final statistical analysis plan
3. Summary of changes to the statistical analysis plan, summary of changes to the protocol.

Protocol Summary

Original protocol and statistical analysis plan

Final protocol and statistical analysis plan

Summary of changes to the statistical analysis plan

Summary of changes to the protocol

Spinal control during functional activities to improve low back pain outcomes

---

Principal Investigator:
Linda Van Dillen, PT, PhD
Professor, Program in Physical Therapy and Department of Orthopaedic Surgery
Campus Box 8502
4444 Forest Park Avenue
St. Louis, MO 63108
(314) 286-1427
vandillenl@wustl.edu

NIH/NICHD/NCMRR R01 HD047709
ClinicalTrials.gov: NCT02027623

Washington University HRPO#: 201205051

Washington University in St. Louis
SCHOOL OF MEDICINE
Physical Therapy

Table of Contents

Original Protocol and Statistical Analysis Plan 5
1. Background and Introduction 5
2. Specific Aims 7
3. Approach 8
3.1 Design 8
3.2 Subjects 8
3.3 Study flow chart 9
4. Enrollment of Participants 9
4.2 Inclusion Criteria 9
4.3 Exclusion Criteria 9
4.4 Recruitment 10
4.4a Clinics/Offices 10
4.4b Clinic Enrollment Flowchart 11
4.5 Initial Screening 11
4.5a Screening to Enrollment Flow Chart 12
5. Laboratory Visits 12
5.1 Informed Consent 12
5.2 Baseline Examination/Laboratory Measures 13
5.3 Randomization 13
5.4 Laboratory Visit 2 & 3 13
6. Treatment Protocol 13
6.1 Overview 13
6.2 Motor Skill Training (MST) 14
6.3 Strength and Flexibility Exercise (SFE) 15
6.4 Training and monitoring of clinicians providing treatment 15
7. Treatment Visit Procedures 16
7.1 Clinic Visit 1 16
7.2 Follow-up Clinic Visits 16
7.3 Final Visit 17
7.4 Booster Phase 17
7.5 Reporting Worsening of Status and Procedures 17
7.6 Adherence/Non-Adherence During Treatment Period 18

8. Human Protection (HRPO) **18**
8.1 Charts 18
8.2 Visit Documentation 18
8.3 Blinding, Contamination, and Privacy Issues 18
8.4 Log of Adverse Events 19
8.5 Data and Safety Monitoring Plan 19
9. Assessments and Outcome Measures **20**
9.1 Outcome Measures Overview 20
9.2 Key Self-Report Measures 20
9.3 Adherence to Home Program 20
9.4 Descriptions of Self-Report Measures 20
9.4a Time Table for Administration 24
10. Data Management **25**
10.1 Data Management Overview 25
10.2 Sample Size Estimate 25
10.3 Analyses 26
11. References **28**

Final Protocol and Statistical Analysis Plan **34**
1. Background and Introduction **34**
2. Specific Aims **36**
3. Approach **37**
3.1 Design 37
3.2 Subjects 37
3.3 Study flow chart 37
4. Enrollment of Participants **38**
4.2 Inclusion Criteria 38
4.3 Exclusion Criteria 38
4.4 Recruitment 38
4.4a Clinics/Offices 39
4.4b Clinic Enrollment Flowchart 39
4.5 Initial Screening 40
4.5a Screening to Enrollment Flow Chart 40
5. Laboratory Visits **40**

5.1 Informed Consent 40
5.2 Baseline Examination/Laboratory Measures 41
5.3 Randomization 41
5.4 Laboratory Visit 2 & 3 42
6. Treatment Protocol 42
6.1 Overview 42
6.2 Motor Skill Training (MST) 42
6.3 Strength and Flexibility Exercise (SFE) 43
6.4 Training and monitoring of clinicians providing treatment 44
7. Treatment Visit Procedures 44
7.1 Clinic Visit 1 44
7.2 Follow-up Clinic Visits 45
7.3 Final Visit 45
7.4 Booster Phase 45
7.5 Reporting Worsening of Status and Procedures 46
7.6 Adherence/Non-Adherence During Treatment Period 46
8. Human Protection (HRPO) 46
8.1 Charts 46
8.2 Visit Documentation 47
8.3 Blinding, Contamination, and Privacy Issues 47
8.4 Log of Adverse Events 47
8.5 Data and Safety Monitoring Plan 48
9. Assessments and Outcome Measures 48
9.1 Outcome Measures Overview 48
9.2 Key Self-Report Measures 49
9.3 Adherence to Home Program 49
9.4 Descriptions of Self-Report Measures 49
9.4a Time Table for Administration 53
10. Data Management 54
10.1 Data Management Overview 54
10.2 Sample Size Estimate 54
10.3 Analyses 54
11. References 55

Summary of Changes to Statistical Analysis Plan......60
Summary of Changes to the Protocol......60

Original Protocol and Statistical Analysis Plan

1. Background and Introduction

Significance

At least 60%-80% of adults will experience LBP,[1] and almost ½ of them will have had a major episode by age 30.[2] Chronic LBP is the most common type of chronic pain in adults[1,3] and its prevalence is increasing.[4,5] At least 75% of people who have a LBP episode, fail to recover fully within 1 year.[6] Pain persists as do limitations in simple movements and complex functional activities like self-care, social role, and work.[3] Recurrence rates are as high as 78%.[7] Thus, for many people LBP is a long-term, function-limiting condition rather than a short-term, self-limiting condition.[6,8-11] LBP-related costs are staggering. Estimated annual costs for LBP-related health-care are $30.7 billion and are increasing.[12]

Treatment of chronic LBP

Exercise is one of the primary non-surgical approaches used worldwide for managing LBP.[13-16] Based on systematic reviews, experts concluded that exercise is as efficacious, if not more efficacious for managing chronic LBP than 1) no treatment, 2) usual care, and 3) many other treatments, such as massage or laser therapy.[13,15-22] Experts also concluded that specific characteristics of exercise programs influence their efficacy[23] and that the most efficacious programs are 1) tailored to the individual, 2) delivered with supervision, and 3) designed to achieve high doses by emphasizing adherence. Despite the evidence for the beneficial effects of exercise in chronic LBP, several issues remain unresolved. First, little is known about which exercise is best for which patient.[15,22] There is no evidence that any particular type of exercise is clearly and consistently more efficacious than any other.[13-16,20-22,24,25] Many experts have suggested that differences between approaches have been missed because the heterogeneity of subjects in samples[13,15,16,20-22,26] and variability in the treatments studied have diluted the effects.[23,27] Second, when exercise is compared to non-exercise approaches, the effects tend to be small and inconsistent for outcomes like pain and function[14,20,22] and most of the significant effects are obtained only at the earliest follow-up.[14,22] Third, adherence and tests of the effects of adherence on outcomes are rarely examined.[16,18,21,23,28] Fourth, few studies examine the mechanisms underlying exercise effects,[20-22,25,27] so we do not know whether improvements in outcome with exercise are consistent with mechanism-based predictions for specific variables. The primary recommendation from review groups is that future studies should investigate the effect of individualized exercise strategies in well-defined populations of people with LBP.[14,21-24,27] The exercises should be aimed at restoring normal function and facilitating the person's performance of daily activities.[13,18,29] Furthermore, they recommend that future studies be designed to examine both the 1) specific mechanisms underlying the effect of exercise, and 2) effects of adherence on outcomes.[16,20-22,25,30] We will address all 3 of the recommendations.

Individualized exercises for restoring normal function and performance of daily activities

Given the profound impact of LBP on function,[3,6,12] a logical approach requires inclusion of explicit, individualized practice in performance of functional activities as a form of exercise. Interestingly, only physical conditioning programs, such as work hardening, work conditioning, and functional restoration include practice of activities as part of treatment.[31-36] However, these programs are primarily intended for people with disabling, work-related LBP. Thus, many of the activities that are practiced are 1) heavy work activities rather than routine functional

activities,[32,33,36-38] and 2) not typically tailored to the individual. Finally, the programs tend to include a variety of co-interventions so the effect of any specific component is unclear. [32,35]

Neural mechanisms underlying the effect of exercise

Because both exercise and practice in performance of functional activities are types of motor training, studies in the motor training literature can inform our understanding of the mechanisms underlying their effects. Motor training induces structural and functional adaptations across a variety of motor regions of the nervous system.[39-43] In a review of the effects of skill, endurance, and strength training on the motor cortex and spinal cord, Adkins et al. emphasized the fact that the nature and location of adaptations are dependent on the specific behavioral demands of the motor experience.[44] Skill training is defined as the acquisition and refinement of novel combinations of motor sequences. Skill training results in synaptogenesis, synaptic potentiation, and reorganization of movement representations in the motor cortex.[44,45] Such adaptations are associated with the acquisition of new motor skills and vary with the degree of motor skill a person displays.[43,46-48] By contrast, strength and endurance exercises do not produce these adaptations.[49,50] If a goal of LBP rehabilitation is to aid acquisition, retention, and refinement of new motor behaviors to improve function, then the use of motor skill training is a necessity.

Model for development and course of LBP

The Kinesiopathologic Model (KPM) provides a conceptual basis for understanding the development and course of LBP. Specifically, LBP is proposed to be the result of repeatedly using direction-specific (flexion, extension, rotation, some combination) stereotypic movement and alignment patterns in the lumbar spine. The patterns begin as the result of an interaction between an individual's structural characteristics and the movements and alignments needed to perform daily functional activities.[51-57] Over time, the same patterns are used repeatedly for many different functional activities. The typical stereotypic pattern is characterized by the lumbar spine moving more readily in its available range in a specific direction(s) than the thoracic spine or hip.[57-61] Because the same patterns are used repeatedly, the loading is always on the same spinal tissue and tissue stress is concentrated in specific areas.[62] Repetitive loading of the same tissue could also alter the tissue's tolerance over time, accelerate the rate of mechanical injury, and potentially lead to tissue degeneration. Thus, the KPM describes a mechanism by which repetitive loading during performance of daily functional activities could contribute to the development and course of this often long-term, function-limiting condition.

Potential for individualized motor skill training to improve long-term course of LBP

In our recently completed clinical trial, we compared the effects of a classification-specific (Sp) treatment to a non-specific (NSp) treatment over a 12 month period. The Movement System Impairment (MSI) classification system that we used to classify subgroups of people with LBP is based on the KPM.[51] People in the various LBP subgroups exhibited direction-specific, stereotypic patterns of lumbar movement and alignment that were associated with their LBP. The Sp treatment included both exercise and training in functional activities directed at modifying the specific pattern(s) associated with the person's LBP. The NSp treatment included strength and flexibility exercise as well as training to maintain a neutral alignment of the lumbar spine during functional activities. We found that people in both the Sp and NSp groups had very large clinically meaningful improvements (61% at 6 mos) in pain and function[63] but differences between groups were smaller than expected.[64] We attributed the results to 3 related facts: 1) in addition to exercise, both groups received training to improve lumbar spine control during functional activities, 2) in both groups, adherence levels were higher and more prolonged for training in functional activities than for exercise, and 3) outcomes continued to improve for about 6 months after treatment but then gradually reversed along with declines in adherence to functional activity training. Changes in pain and function were much more strongly associated

with adherence to functional activity training than with adherence to exercise. Thus, training in functional activities appears to be the essential active ingredient for improvement in both groups. Accordingly, the next important steps are to 1) disentangle the effects of training in functional activities and exercise, 2) maximize the effect by providing individualized motor skill training during functional activities based on principles of motor skill learning, 3) prolong the treatment effects by providing a booster treatment at 6 months, and 4) enhance our understanding of the underlying mechanism by testing for predicted differences in the effects of the interventions on laboratory measurements of movement and alignment.

Impact on clinical practice

Currently rehabilitation for people with chronic LBP is highly variable and is usually focused only on treatment of acute recurrences.[28,65-67] Successful completion of this project will provide specific recommendations for the use and timing of individualized motor skill training for functional activities that can be applied immediately in practice. Because the training is focused on how an individual can painlessly perform functional activities that are limited, our findings will provide clinicians with an approach to treatment that directly addresses the factors proposed to contribute to the costly, long-term course of LBP. Also because the treatment is associated with prolonged periods of high levels of adherence, and uses specifically timed, periodic booster sessions to reinforce behaviors that minimize the patient's symptoms and functional limitations, our findings will provide the clinician and the person with LBP a feasible approach to long-term management of the condition, rather than just treating acute recurrences. Such an approach to LBP care could have an important impact on the long-term course (i.e., persistent or recurrent symptoms) and associated health-care costs of chronic LBP.

Focus on a treatment associated with high levels of adherence

The level of adherence during long-term treatment for most chronic diseases is only ~50%.[68] Poor adherence equates with low treatment dosage and consequent diminution of treatment effects. Data from our clinical trial demonstrated that 1) adherence levels were higher and more prolonged for training in functional activities than for exercise, and 2) changes in pain and function were more strongly related to functional activity adherence than to exercise adherence. Thus, in the proposed project we are focusing on a treatment to which people are able to adhere at high levels for prolonged periods of time.

Booster phase to prolong initial treatment effects

The purpose of the booster phase is to prolong the initial improvements seen in our clinical trial. Others have tested the effect of booster treatments in people with musculoskeletal pain conditions, including chronic LBP.[69-72] The innovativeness of our approach lies in the basis for the criteria we use for determining when to intervene and how much treatment to provide. Rather than providing additional treatment arbitrarily, we have scheduled the booster phase to start just before the time when outcomes began to reverse in our prior trial. The criterion for the number of booster sessions (i.e., how much treatment) is the number needed to attain independence in performance of the treatment items.[73]

2. Specific Aims

Specific Aim 1: Test if motor skill training results in better outcomes and better adherence than strength and flexibility exercise in the 12 months after treatment.

Compared to the strength and flexibility exercise group, the motor skill training group will

Hypothesis 1a. report lower LBP intensity, fewer days with LBP, and less frequent LBP recurrences.

Hypothesis 1b. report fewer functional limitations as well as better perceived physical health and mental health, work attendance, and productivity.

Hypothesis 1c. use fewer LBP medications and fewer healthcare services.

Hypothesis 1d. report higher levels of adherence over longer periods of time.

Hypothesis 1e. display improved movement and alignment patterns during functional activities.

Specific Aim 2: Test the effect of a booster phase that is provided 6 months after the initial phase.

Hypothesis 2a. The effect of a booster treatment condition will be longer-lasting improvement in function and better adherence than a no booster treatment condition.

Hypothesis 2b. The effect of a booster treatment condition will be greater for the motor skill training group than for the strength and flexibility exercise group.

Specific Aim 3: Examine the relationship between functional limitations and 2 factors 1) adherence, and 2) movement and alignment patterns displayed during functional activities.

Hypothesis 3a. Adherence to motor skill training will be related more strongly to functional limitations than will adherence to strength and flexibility exercise.

Hypothesis 3b. Movement and alignment patterns displayed by the motor skill training group will be related more strongly to functional limitations than will patterns displayed by the strength and flexibility exercise group.

3. Approach

3.1 Design

The study is a single-blind, prospective, randomized, controlled clinical trial. Figure 1 illustrates the organization and flow of events. After we collect baseline self-report and laboratory measures, people will be randomized to 1 of 2 treatment conditions, motor skill training (MST) or strength and flexibility exercise (SFE). The initial phase will be 6, 1-hour treatment sessions, scheduled once/week for 6 weeks. Post-treatment self-report and laboratory measures will be obtained immediately after the initial phase. A select subset of web-based, self-reports will be obtained monthly for the next 12 months (9.4a Time Table for Administration). At 6 months after treatment, self-report and laboratory measures will be obtained again. Subsequently, people will be randomized to a booster treatment condition, booster or no booster. The booster treatment will be specific to the person's initial, condition-specific treatment assignment. The number of booster sessions will be limited to the number needed to attain independence in the exercises or functional activities prescribed. Independence will be based on a standardized measure used in our prior trial.[73] The 6-month time point was chosen based on change in the Modified Oswestry Disability Questoinnaire (MODQ) scores from our prior trial.

3.2 Subjects

We will recruit 154 people between the ages of 18 and 60 years who have had chronic, non-specific LBP for at least 1 year from the St. Louis metropolitan area. Chronic LBP is defined as pain present for at least half the days in a year in the region between T12 and the gluteal fold, with or without leg symptoms to the knee.[11,74] Non-specific LBP is defined as LBP in which no specific pathophysiologic mechanism can be identified to account for the person's perceived pain.[75] The goal of this study is to obtain a representative sample of people who have both symptoms and disability related to chronic LBP. The target participant population is people with chronic LBP, with or without recurrences. The goal is to examine the generalizability of the effects seen in the previous clinical trial to people who have more LBP-related limitation than people previously studied. This will be accomplished by recruiting participants from the Washington University School of Medicine Physical Therapy Clinic (WUPT Clinic) and local outpatient clinics and offices. Only participants who report a specific criterion level of disability and difficulty with a specific number of functional activities will be enrolled. Both genders and all races and ethnicities will be included in the study population.

3.3 Study flow chart

Figure 1

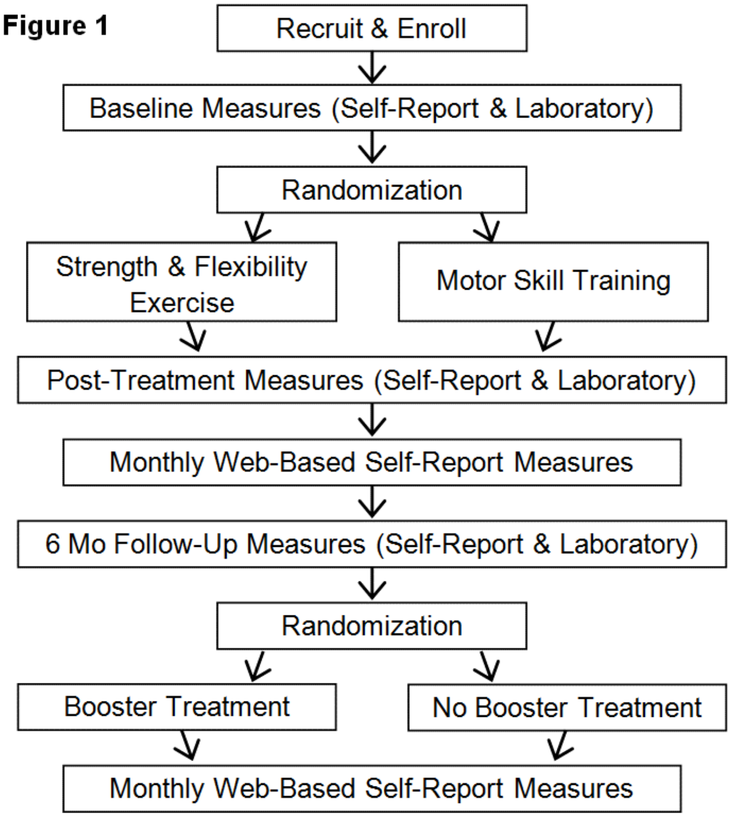

4. Enrollment of Participants

4.2 Inclusion Criteria

The inclusion criteria are 1) people ages 18-60 with chronic LBP for a minimum of 12 months, 2) currently experiencing LBP symptoms but not in a recurrence or an acute flare-up, 3) MODQ score of ≥ 20%, 4) 3 or more functional activities (simple or complex) limited due to LBP, 5) able to stand and walk without assistance, and 6) able to understand and sign a consent form.

4.3 Exclusion Criteria

The exclusion criteria are 1) BMI >30, 2) any structural spinal deformity including scoliosis, kyphosis, or stenosis, 3) spinal fracture or dislocation, 4) low back pain due to trauma, 5) osteoporosis, 6) ankylosing spondylitis, 7) rheumatoid arthritis, 8) disc herniation (i.e. diagnosis with symptoms below the knee), 9) spondylolisthesis, 10) serious spinal complications such as tumor or infection, 11) previous spinal surgery, 12) frank neurological loss, i.e., weakness or sensory loss, 13) pain or paresthesia below the knee, 14) etiology of LBP other than the lumbar spine, e.g., hip joint, 15) history of neurologic disease which required hospitalization, 16) active treatment for cancer, 17) history of unresolved cancer, 18) pregnancy, 19) worker’s compensation or disability case, and 20) in litigation for the LBP problem.

4.4 Recruitment
People with LBP will be recruited from the WUPT clinic, local clinics and offices, and local advertisements and health fairs. In all clinics, a list of the inclusion/exclusion criteria and possible diagnoses will be available. Flyers will also be placed in all office areas and given to clinic staff for reference.

4.4a Clinics/Offices
Study flyers will be placed in all area clinics/offices that see potential LBP participants and are willing to assist with study recruitment. Patients that are being referred for physical therapy treatment of their LBP also will be asked if they are interested in the study. If they are interested, their information can be given to the research coordinator (RC) or research assistant (RA) or the patient can contact the RC or RA directly. If the person is not interested, the person will be scheduled as usual. The RA or RC will log everyone she contacts on the Telephone Screening Log. If the person does not qualify for the study, he will be told to schedule his appointment with the clinic. If a person exceeds three callbacks, he will no longer be contacted.

4.4b Clinic Enrollment Flowchart

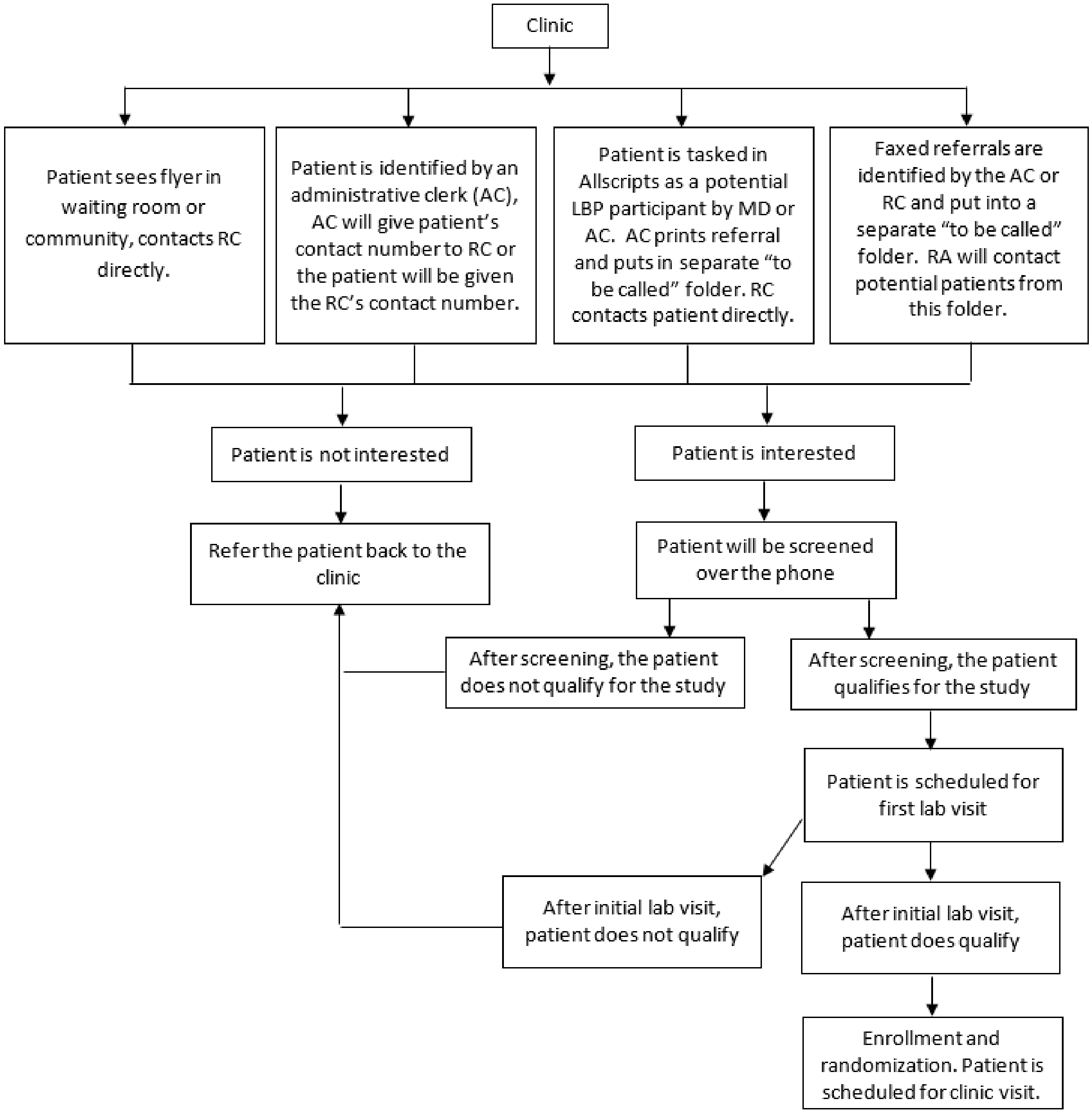

4.5 Initial Screening

The RA will speak with interested individuals via phone. The RA will briefly describe the trial and the procedures the person will participate in. Informed consent for a telephone screening will be obtained. The RA will then interview the individual using a series of screening questions to determine eligibility for the trial. If the person is deemed ineligible by phone screening, then the RA will explain the reason why he does not qualify (DQ). If the person is deemed eligible by phone screening, he then will be scheduled for a baseline laboratory visit where additional screening will be performed. All individuals who are screened will be entered into a database indicating their eligibility/ineligibility for the trial.

4.5a Screening to Enrollment Flow Chart

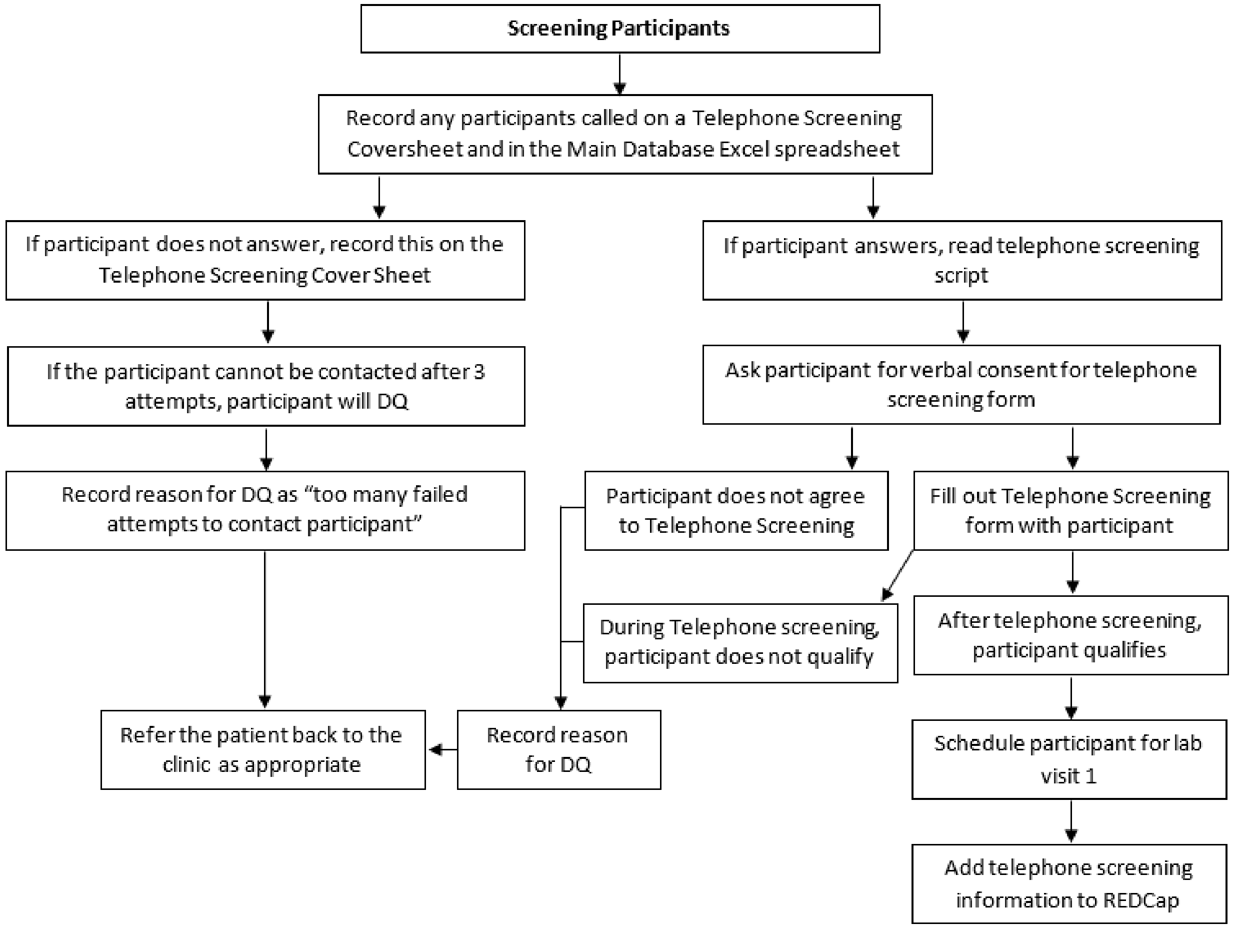

5. Laboratory Visits

5.1 Informed Consent

If the person is eligible, he will be scheduled to come in for examination with the Research Physical Therapist (RPT). On the first visit to the laboratory the Principal Investigator (PI), RPT, RC, or RA will explain the informed consent process to the person. The person will then be taken through the specific details on the informed consent form approved by Washington University's Human Research Protection Office (HRPO). In particular, the person will be told the procedures he will participate in, the potential risks and benefits of participation, and his right to refuse participation at any time without any effect on his subsequent health care. The person then will be given an opportunity to read the consent form. The PI, RPT, RC, or RA will be available to answer any specific questions, highlight the important details of the study again, and remind the person of his rights as a participant. The person will then be asked to sign the consent form. A copy of the form and a brochure explaining the person's rights under the Health Insurance Portability and Accountability Act (HIPAA) regulations will be given to the person for his records. The signed consent form will then be signed by the person administering consent. The consent form will be filed in a locked cabinet separate from the person's data.

5.2 Baseline Examination/Laboratory Measures

Participants who are eligible for participation in the study and provide informed consent will be examined. At the initial visit, height and weight will be collected from each participant with clothing on and shoes off using a manual scale. Height will be collected to the nearest ¼" and weight will be collected to the nearest pound. The participant will then complete a Demographics and LBP history questionnaire, the Numeric Pain Rating Scale (NRS), and the MODQ. Participant eligibility of MODQ ≥ 20% score and at least 3 functional activities limited due to LBP will be confirmed. Next, the participant will be examined by a licensed, trained RPT who will use a standardized exam to classify the participant's LBP based on the MSI classification system.[51,76-79] The participant then will complete additional self-report measures and laboratory testing. Because all baseline testing occurs *before* randomization, research personnel involved in testing will be *blinded* to the participant's treatment assignment throughout the study.

Laboratory Measures

Participants will perform functional activity tests that (1) parallel activities reported as problematic for the majority of participants in prior studies, and (2) have been examined previously. Retroreflective markers will be taped to the skin at predetermined locations on the participant's trunk, pelvis and legs. Data from 3 trials of each test will be captured. LBP will be recorded during each trial.

5.3 Randomization

The sample will be stratified based on LBP classification. After baseline assessment, a participant will be assigned randomly, within his strata, to either the (1) MST with no booster, (2) MST with booster, (3) SFE with no booster, or (4) SFE with booster condition. The purpose of stratification by LBP classification is to ensure similar representation of LBP subgroups in the treatment conditions and eliminate any confounding of classification and treatment. Note that it is not our goal to determine if LBP classification moderates treatment impact; there will not be a sufficient number of subjects in some LBP classifications to address that issue with adequate power. Randomization will be conducted using a computer-generated list of random numbers provided by the biostatistics team. The randomization scheme will be prepared *before* initiation of the trial and will be adhered to *throughout* the trial. Assignments will be performed within the REDCap system once the Screening & Randomization form is completed. When the participant with LBP is enrolled, and assigned a group, the RC will print the computer-generated group assignment and schedule the participant with the appropriate therapist for all 6 clinic visits. The first clinic visit should be scheduled within one week of the laboratory visit. The participant will not be blinded to treatment assignment, but will be blinded to his assignment to booster/no booster until completion of Laboratory Visit 3 (the 6 month visit).

5.4 Laboratory Visit 2 & 3

All measures and procedures used during Laboratory Visit 1 will be used again for the post-treatment follow-ups. All participants will be reminded not to discuss the details of their treatment with *blinded* research personnel so that blinding to treatment assignment is maintained. Laboratory Visit 2 will be scheduled within one week after discharge from the treatment phase. Laboratory Visit 3 will be scheduled 6 months following Laboratory Visit 2.

6. Treatment Protocol

6.1 Overview

Both treatment groups will participate in a 1-hour treatment, once a week for six weeks. Each group will also be given a home program to perform during the entire 12-month study period. All treatments will be provided by licensed physical therapists trained in the respective treatments.

The therapists cannot be blinded to treatment assignment, but are blinded to booster/no booster assignment until the participant completes Laboratory Visit 3. None of the therapists, however, will be involved in either (1) collecting baseline or follow-up data, or (2) laboratory meetings with research personnel. In the initial phase of treatment, both groups will be provided the same amount of time in treatment. Documentation of treatment prescription and progress is standardized (including harm). This documentation will be used to track treatment fidelity. Rules for prescribing and instructions for performing treatment items are standardized and documented; instructions with photos will be available in hard copy.

6.2 Motor Skill Training (MST)
The motor skill training involves supervised, massed practice of novel, challenging functional activities. The overarching principle of training is that the participant needs to practice performing the functional activities in ways that (1) minimize use of the participant's specific, stereotypic lumbopelvic movement and alignment patterns (based on the participant's LBP classification), and (2) encourage use of other joints (thoracic spine, hips, knees) to complete the activity painlessly. The lumbopelvic patterns related to the participant's specific LBP classification identified during the laboratory examination (flexion, extension, rotation, extension & rotation, or flexion & rotation) will be targeted. We will use information about the participant's LBP classification to guide the training because (1) our data suggest there is added benefit to classification-based training, (2) a meta-analysis of treatment studies showed that individualized treatment provides added benefit to outcome, and (3) there is evidence supporting the existence of LBP subgroups. Participants will be educated that the primary contributors to their LBP symptoms are repeated movements and prolonged positions of the lumbopelvic movement patterns related to their specific LBP classification. Additionally, participants will be educated that the emphasis of treatment is to learn how to modify or change how they perform their daily activities so that they do not use these patterns repeatedly throughout the day. During practice, the participant will be given cues for using the trunk muscles needed to facilitate a correct lumbopelvic pattern. Thus, rather than prescribing exercises for individual trunk muscles, we will attempt to modify how the participant uses multiple trunk muscles within the context of the activity in which they will be used. Such an approach should facilitate the use of these muscles in daily functional activities and contribute to enhanced adherence.

Specific activities are selected for a participant to practice if performing them is painful or difficult because of his LBP. To enhance salience, the participant will be involved in selection of the activities to be practiced. In addition, a pre-determined set of activities will be included for all participants because we know from prior studies that those activities are difficult for most people with chronic LBP to perform. The set of activities parallels the functional activities that will be tested in the laboratory. Three activities will be practiced for ~15-20 minutes each. Rest will be provided, as requested. All activities will have 3 essential components: (1) contraction of groups of specific trunk muscles, (2) earlier and greater movement of the hip, knee, and/or thoracic spine relative to the lumbar spine, (3) later and less movement of the lumbar spine relative to other regions. The muscles that need to be activated and the directions of movement and alignment that need to be modified will be specific to the participant's LBP classification. Within each activity, the conditions of practice will vary. For example, sit ↔ stand will involve practicing with various (1) seat surface heights, (2) seating materials, and (3) constraints of surroundings. The order in which activities are practiced will be randomized. All participants will practice simple activities (e.g., moving in bed) before complex activities (e.g., gardening). The level of difficulty of the activity will be graded to match the motor capabilities of the participant and progressed based on ability to (1) verbalize the key concept for that skill (2) appropriately modify the movement or alignment and (3) control LBP symptoms during performance of 5 repetitions of the skill. We use a highly reliable measure to assess these characteristics and decide if the

participant can be progressed to a higher level of difficulty.[73] Participants need to practice activities without reproduction of their LBP for three reasons. First, in experimentally-induced pain conditions, researchers have shown that motor learning and related neuroplasticity can be hindered. Second, if the stereotypic patterns do contribute to the participant's LBP, modification of the patterns should alleviate the LBP. Inability to perform the activity without pain could mean the pattern has not been fully modified. Third, increased pain levels during exercise are associated with poor adherence. A home program will be prescribed of 1-2 sets of 5-10 repetitions of formal practice of the activities practiced in treatment sessions.

An abdominal brace or tape can be used with any skill as indicated based on the participant's LBP classification and severity level. The use of a brace or tape is optional. Performing the skill without the brace or tape would make the skill more difficult. The decision to use or not use a brace or tape can be used in grading the skill. Some participants may need to always use a brace or tape for performance of specific skills. If the patient sits for prolonged periods of time, has anatomy that makes him vulnerable to poor positioning or any other factors that would contribute to making adherence to the positioning recommendation difficult, providing positioning devices is appropriate and should be considered.

6.3 Strength and Flexibility Exercise (SFE)

Exercises for trunk strength and trunk and lower extremity flexibility commonly cited in the literature as being appropriate for people with chronic LBP and included in the previous trial will be used. The overall goals of treatment will be to increase the participant's trunk strength and trunk and lower limb flexibility; if necessary, equipment will be provided. Participants will be educated on possible contributors to pain, including poor posture, stress, loss of strength and flexibility, and general decline in physical fitness. The therapist will be blinded to the LBP classification to avoid any potential bias in the prescription of exercise or education, and participants will not be educated about their LBP classification. Additionally, participants will be educated about the benefits of strength and flexibility exercises and the expectation that performance of these exercises will improve performance in daily activities limited by LBP. Participants will be instructed that muscle soreness is expected with performance of new exercises.

Strengthening exercises will target all trunk muscles. Flexibility exercises will target all trunk and lower limb motions. Treatment is divided into 3 phases with each phase being progressively more difficult. All participants will start with Phase 1 exercises regardless of strength or previous experience with back pain exercises. All participants will be instructed to perform a home program of stretching every other day and strengthening every other day. At each treatment session, the therapist will assess the participant's independence in performance of the exercises and if the participant has met the criteria to advance to the next phase. Progression of exercise will be based on independence in: knowledge of the key concept (purpose of the exercise), correct performance without cues, and performance of the maximum number of repetitions of each of the exercises (4 repetitions of 30 second holds for stretching and 3 sets of 10 repetitions for strengthening).

6.4 Training and monitoring of clinicians providing treatment

Clinicians administering treatment in both conditions will be trained before data collection begins. SFE clinicians will be trained by the PI. MST clinicians will be trained by the PI and a co-investigator with expertise in motor learning. Clinicians will be provided with training manuals describing the treatment procedures in detail for each condition. Clinician proficiency in delivering the treatment will be assessed with practical examinations administered yearly by the co-investigator (MST) or the RPT (SFE). Clinician knowledge of treatment protocols will be

assessed yearly with written examinations. Treatment fidelity will also be monitored by regular review of clinician documentation. The PI will hold monthly meetings with the treating clinicians in each condition to discuss any questions about the treatment protocol or implementation. The treating clinicians will not discuss any participants' names or other identifying information during these meetings to maintain blinding.

7. Treatment Visit Procedures

7.1 Clinic Visit 1

Duration

The initial treatment is 1 hour in duration. This does not include the time the participant is completing outcome measures or the therapist's documentation of the visit. It is acceptable if the participant requires more time this first visit for initial education and instruction, the therapist should record the amount of time spent in treatment on the Clinic Visit 1 form.

Before the participant arrives for his first visit, the therapist should review the participant's Clinic Visit 1 folder that includes all of the information from Laboratory Visit 1. This includes the following forms: (1) Medical Screening, (2) Demographics, LBP and medical history, (3) NRS, (4) SF-36, (5) MODQ, (6) Absenteeism & Presenteeism, (7) FABQ, and (8) Clinical Examination Data (History and Physical). The clinical examination data related to a participant's LBP classification is deleted from the forms for the clinicians providing the SFE treatment. This is done to maintain blinding of a clinician to a participant's LBP classification. There should also be a Clinic Visit 1 form and a Treatment Progress Table appropriate to the participant's treatment group. The therapist will receive an additional folder that includes all of the paperwork for the follow-up clinic visits.

Initial Interaction

While the participant is waiting for treatment to be initiated, the therapist should give the participant the following forms to complete: (1) the NRS, and (2) the MODQ. The participant's progress since Laboratory Visit 1 should be reviewed. The therapist will then (1) provide the overview of the trial and the specific treatment condition and (2) explain the overall goals of the treatment condition.

Treatment

Treatment during Clinic Visit 1 will consist of (1) an explanation of Educational Concepts/Principles, and (2) treatment based on the treatment condition assignment. At the end of Visit 1 the participant is provided with (1) handouts for initiation of the home exercise program (HEP), (2) the Daily Treatment Adherence Log to be filled out during the days between visits, (3) an explanation of how to the complete the Daily Treatment Adherence Log, and (4) the therapist's contact information in case the participant needs to reschedule a visit, has any questions, or experiences a worsening of status. The therapist records the events of Visit 1 in the participant's chart on the Clinic Visit 1 documentation form and Treatment Progress Table. The time spent in treatment should be recorded on the Clinic Visit 1 documentation form.

7.2 Follow-up Clinic Visits

All treatment visits should be 1 hour in duration. This does not include the time the participant is completing outcome measures. The time spent in treatment should be recorded on the Clinic Follow-Up Visit documentation form. The participant should come to the reception area of the clinic. The therapist should take the participant to a clinic treatment room upon arrival to keep the participant from spending time in the reception area where discussion of treatment with other participants may occur, potentially contributing to contamination of treatment. At this time

the therapist should provide the participant with the (1) NRS, (2) MODQ, and (3) Educational Principles quiz to be completed before treatment is initiated. The therapist reviews the NRS, MODQ, Daily Treatment Adherence Log and Educational Principles quiz as well as events since the last visit. The participant's questions are answered and treatment is reviewed and progressed. Education is focused on principles not understood based on quiz performance. The majority of time should be spent on the exercises. The participant is given updated handouts of the HEP as needed, a Daily Treatment Adherence Log, and is reminded of the next visit date and time. The therapist records the events of the visit in the participant's chart on the Clinic Follow-Up Visit documentation form (including the time spent in treatment) and the Treatment Progress Table.

Each clinic visit needs to be at least 5 calendar days to no more than 2 weeks after the previous clinic visit. If a participant is up to 30 minutes late to his/her clinic visit and the clinician cannot treat for the full hour, he/she should treat for 30 minutes and schedule an additional treatment visit as soon as possible for the additional 30 minutes. The clinician should also inform the PI within 24 hours of the late visit. Clinic visits should be split no more than 2 times, for a maximum of 8 clinic visits for a participant. If the participant is more than 30 minutes late, it is the discretion of the therapist as to whether the visit will be completed or rescheduled.

7.3 Final Visit

On the final visit, the participant's HEP should be reviewed so that the participant knows what he is expected to continue upon discharge. The therapist should encourage the participant to continue to be active and continue with exercises regularly as prescribed. The therapist should make sure the participant has scheduled his follow-up laboratory visit. The participant should be reminded that he will be receiving monthly follow-up surveys via email which he will be paid to complete. The therapist should provide his current contact information if the participant has questions or has a status change after discharge. The participant's follow-up laboratory visit should be within the week after discharge from the treatment phase.

7.4 Booster Phase

The booster phase will occur 6 months after discharge from the initial treatment. Participants will be randomized to a booster condition (their original condition-specific treatment) or a no booster condition at the initial laboratory visit. The participant is told his assignment to boosters (booster/no booster) at completion of Laboratory Visit 3 (the 6 month visit). Each booster session will be 1 hour. The content of the booster treatment will consist of review/practice of the home program from the initial treatment phase. The number of sessions will be based on the number needed to attain independence in the prescribed treatment items, with a maximum of 3 visits. Participants will see the same treating therapist during the treatment phase and all booster sessions. If a participant is randomized to the booster condition, the RPT will contact the referring provider and inform him/her that the participant will be receiving additional treatment sessions.

7.5 Reporting Worsening of Status and Procedures

For both MST and SFE, a worsening of symptoms is defined as an increase in the intensity of LBP symptoms or peripheralization of symptoms (symptoms located more laterally and/or distally from the original location) that lasts for greater than 4 hours after performance of the treatment items and has not fully resolved after 24 hours. The participant should call the therapist if there is a worsening of status (worsened symptoms or red flags are present) or if the participant is concerned for any reason about his status. If the participant reports a worsening of symptoms (as operationally defined), the therapist should discuss the possible causes of worsening such as a change in activity level, specific activities, work conditions, sleeping habits,

etc. If worsening appears to be caused by the HEP the participant should be instructed to stop exercising for 24 hours and encouraged to use pain relieving techniques. These include using medications as prescribed by his physician, and application of cold if within 48 hours of worsening or heat if greater than 48 hours since worsening. After the 24 hours has passed, the participant should begin the exercises/skills being practiced, starting with 1 exercise/skill the first day. The participant should add 1 exercise/skill per day and report to the therapist if any specific exercise/skill increases his symptoms. In the SFE condition an increase in symptoms during exercise is not contraindicated and the participant should continue. If a particular exercise/skill appears to cause a worsening, the participant should be instructed not to perform that specific exercise/skill until the next visit. Performance will then be reviewed and modified as needed. If the participant feels that certain exercises/skills are worsening his symptoms, these should be stopped until the next visit. These, however, typically would not be expected to worsen the participant's status if performed correctly, so these exercises should specifically be reviewed for correct performance at the treatment visit. If red flags are present, the participant should contact his physician for follow-up and contact his therapist once he has communicated with the physician. The therapist should complete the adverse event form and follow the procedure for reporting an adverse event.

7.6 Adherence/Non-Adherence During Treatment Period

All participants should be encouraged to actively participate in their treatment program across the 6 visits as well as after discharge. If the participant is having difficulty adhering, the therapist should discuss strategies for adhering with treatment. If adherence becomes a problem, the therapist should discuss this in the regularly scheduled meetings with the PI or contact her directly to discuss strategies, making sure to not identify the participant so that blinding can be maintained.

8. Human Protection (HRPO)

8.1 Charts

All paperwork in charts will have the participant's ID number. No names or identifying information will be on the paperwork or the chart when the participant's chart is filed away for the day. In the instance there is a name provided, the treating therapist should mask the name by crossing out with a black marker. Charts will be kept in a locked file drawer that is only accessible to the therapist treating the participant.

8.2 Visit Documentation

All documentation for each visit will be completed the day of the visit. This includes the clinic visit form and the Treatment Progress Table. Chart audits will be conducted throughout the study period to ensure adherence to the protocols and complete data sets per participant per measure. For this reason, the charts should always be up to date and locked in the designated filing area.

8.3 Blinding, Contamination, and Privacy Issues

Blinding

There can be no discussion of participants' names when interacting with research personnel. All research personnel are to remain blinded across the study period to all participants' treatment condition assignment. Research personnel include the following: (1) the Principal Investigator, (3) the Research Physical Therapist, and (3) the Research Assistant. All paperwork associated with the treatment of the participant must be coded with a participant ID number. The Research Coordinator is unblinded to the participant's treatment condition assignment, and can be contacted with regard to any participant issues.

Privacy Issues
Risks of confidentiality: All participants will be informed of HIPAA and will be given written information reiterating HIPAA regulations. All people at the clinic and laboratory sites have been trained in HIPAA regulations and procedures. All people interacting with the participant will be reminded of the HIPAA procedures across the study period so that confidentiality is maintained. The clinical and laboratory sites have also been set up to comply with HIPAA regulations. All data forms (hard copy or electronic) will be immediately coded and personal identification information will be removed. All hard copy data forms are stored in a locked cabinet. Only research personnel authorized to access data for the proposed project will have access to the locked cabinet. Informed consent forms will be kept in the PI's office in a locked cabinet separate from the data forms. All data collected at the clinical site will be coded and identifying information removed immediately. Data will be stored in a locked file cabinet at the site. A member of the research personnel will pick up clinical data from the clinical site when the participant has completed the treatment phase and bring it back to the laboratory to be stored with the laboratory data. Only the participating therapists and the RC will have access to this file cabinet in the clinic. Therapists who participate in the study will also sign an agreement not to discuss the treatments or progress of any of the participants in the study with other therapists in the clinic, with other participants in the study, or with research personnel blinded to treatment condition assignment. In addition, the institution in which the clinic is housed has agreed to adhere to all guidelines for protection of human subjects outlined by the NIH.

8.4 Log of Adverse Events
All adverse events (AE) should be documented on the AE form. Serious adverse events (SAE) should be immediately reported to the research personnel. The research personnel will report the SAE directly to the IRB using the following protocol set forth by the Human Research Protection Office at Washington University School of Medicine: a) death within 24 hours, and b) all other internal events within 10 days. Should any adverse events deemed to increase risks to participants be identified, the study will stop immediately and an investigation will be conducted. A report indicating the findings will be produced prior to study resumption.

8.5 Data and Safety Monitoring Plan
In addition to ensuring the safety of research subjects, the PI will be responsible for ensuring data safety and confidentiality. This will be accomplished by keeping all identifying information about research data or health identification data in a locked cabinet that can be accessed only by authorized research personnel. In addition to the daily safety procedures outlined, a committee of 3 health professionals at Washington University School of Medicine who are independent of the study have agreed to act as an internal Data and Safety Monitoring Committee (DSMC) to monitor the progress of the study and the procedures for maintaining the integrity and safety of data collection, processing, and analysis procedures as well as the reporting of adverse events. A professor in the Program in Occupational Therapy and the Department of Neurological Surgery and a professor in the Program in Physical Therapy and the Department of Radiology will provide expertise in the area of the overall integrity of the research process, and in particular, maintenance of the integrity of the data collection and analysis process. A physician and associate professor in the Department of Orthopedic Surgery and the Department of Neurology with a specialty in spine disorders and board certification in pain management will provide expertise with regard to spinal conditions, function as the medical monitor, and guide the committee with regard to actions necessary for dealing with and reporting any adverse events. The PI, and other Co-Investigators as appropriate, will meet with the DSMC two times a year to review progress of the data collection process, evaluate any unanticipated or anticipated side effects of participation in the study, and monitor the integrity

and the accuracy of the data generated from the study. The biostatistics team will be responsible for overseeing the presentation of data at the DSMC meetings. In particular, The PI, the biostatistics team and other members of the research team will present recruitment and data issues and analyses at each meeting. Reports will be kept of each meeting and kept on record in the PI's laboratory. The PI and the research personnel will be responsible for implementing any procedures recommended by the DSMC. In addition to data and safety monitoring by the DSMC, adverse events will be reported to the Washington University Human Research Protection Office (HRPO) by the Principal Investigator within 24 hours of the event. Serious adverse events will also be reported to the funding institute. The Washington University HRPO will also monitor the progress of the study, the safety and privacy compliance of the recruitment and data collection procedures, and the proper reporting of adverse events through required yearly progress reports. The yearly progress reports include cumulative reports of any unanticipated or adverse events, yearly renewals of our informed consent document, and approval of all recruitment materials used for the study. If a serious adverse event occurs, the Washington University HRPO will be contacted immediately and the forms accessible through the HRPO website will be accessed to document the circumstances of the event. Changes in the consent form or protocol will be made if indicated based on the nature of the unanticipated or adverse event.

9. Assessments and Outcome Measures

9.1 Outcome Measures Overview

There will be an array of measures to assess outcomes, examine potential treatment effect modifiers, prescribe and progress treatment, and describe the sample. The measures were chosen to capture the important dimensions recommended for the study of people with LBP. All self-report measures will be collected using the automated, web-based system. The Time Table for Administration includes information about the time points of administration. The total administration time for all study measures is ~3 hours. All participants will receive a subset of self-report measures monthly that require ~30 minutes to complete.

9.2 Key Self-Report Measures

Modified Oswestry Disability Questionnaire (MODQ): The primary outcome variable is the 10-item MODQ, a disease-specific measure that provides an index of a participant's perceived LBP-related functional limitation. The scale for the MODQ ranges from 0-100; 100 represents the highest level of limitation. The MODQ is reliable, valid, and sensitive to change.

Numeric Pain Rating Scale (NRS): An 11-point scale (0-10) will be used to measure current pain, as well as average and worst pain over the prior 7 days; larger numbers indicate more intense pain. NRS measurements are reliable, valid, can be treated as ratio scale data, and provide sufficient levels of discrimination to describe pain intensity at varying levels of acuity.

9.3 Adherence to Home Program

During both the initial treatment phase and the booster phase, a standardized self-report measure of daily treatment adherence will be used. Participants will use a Visual Analog Scale (VAS) to indicate the percentage of the treatment they were able to perform as prescribed within a specific time interval (e.g., daily). The scale ranges from 0-100% with higher values indicating higher adherence. This measure will also be used to quantify adherence as part of the monthly, web-based, self-reports.

9.4 Descriptions of Self-Report Measures

Modified Oswestry Disability Questionnaire (MODQ)

Background/Purpose: This questionnaire is designed to give the therapist information as to how LBP has affected a participant's ability to manage in everyday life. It is a 10-item, disease-specific measure that provides an index of the patient's perceived LBP-related functional limitation.
Time points of administration: Laboratory Visit 1, Clinic Visits 1-6, Laboratory Visit 2, follow-up months 1-12, Booster Sessions
Procedure: Participants will answer each of the 10 questions by placing a mark in the one box that best describes his current condition. Since a participant may feel that 2 of the statements describe his condition, he is instructed to mark only the box that most closely describes his current condition.
Scoring: Each item is given a value from 0-5. The total score is the sum of all questions divided by 50, multiplied by 100 to get a percent. 100 represents the highest level of limitation. The minimal clinically important difference (MCID) for the MODQ for people with chronic LBP is 4-6 points.[63]

Numeric Pain Rating Scale (NRS)
Background/Purpose: This questionnaire is designed to give the therapist information on the intensity of a participant's LBP symptoms.
Time points of administration: Laboratory Visit 1, Clinic Visits 1-6, Laboratory Visit 2, follow-up months 1-12, Booster Sessions
Procedure: Participants will rate their LBP symptoms on a numeric scale of 0-10 where 0 represents no symptoms and 10 represents symptoms as bad as can be. They will rate their average symptoms over the prior 7 days, and worst symptoms over the prior 7 days.
Scoring: The score for each item is the rating the participant provides for each symptom category (average and worst). The MCID for the NRS is 2[80,81] points or 30%[80] change from baseline.

Acute Flare-Ups of LBP in Past 6 Months
Background/Purpose: This questionnaire is designed to give the therapist information about the history of a participant's LBP flare-ups in the past 6 months.
Definition: A flare-up is an increase in symptoms of at least 2 points on the NRS above a person's typical low back pain and lasts for at least 2 consecutive days[82]
Time points of administration: Laboratory Visit 1, Laboratory Visit 3, follow-up month 12
Procedure: Participants will fill in information on how many acute flare-ups they have had over the past 6 months, how many days each flare-up averaged in length, and the average pain intensity during the flare-ups.
Scoring: The score for each item will be the number the participant provides for each flare-up category: (1) Number of acute flare-ups over the last 6 months, (2) Average length in days of acute flare-ups, (3) Average intensity of acute flare-ups.

LBP Recurrences
Background/Purpose: This questionnaire is designed to give the therapist information about the history of a participant's LBP recurrences in the past 6 months.
Definition: A recurrence is an increase in LBP symptoms of at least a 2/10 that lasts for at least 24 hours and is preceded and followed by at least 30 days of no symptoms[74]
Time points of administration: Laboratory Visit 1, Laboratory Visit 3, follow-up month 12
Procedure: Participants will fill in how many recurrences of LBP they have had over the past 6 months, how many days each recurrence averaged in length, and the average pain intensity during the recurrences.

Scoring: The score for each item will be the number the participant provides for each recurrence category: (1) Number of recurrences over the last 6 months, (2) Average length in days of recurrences, (3) Average intensity of recurrences.

36-Item Short Form Health Survey (SF-36)
Background/Purpose: This questionnaire is designed to assess information about a participant's mental and physical health, and how well he is able to do his usual activities.
Time points of administration: Laboratory Visit 1, Laboratory Visit 2, Laboratory Visit 3, follow-up month 12
Procedure: Participants will answer each question by marking the answer as indicated. If participants are unsure how to answer a question, they are instructed to choose the best answer they can.
Scoring: Using a scoring application, the SF-36 provides 8 scales: a Physical Functioning, Role-Physical, Bodily Pain, General Health, Vitality, Social Functioning, Role-Emotional, and a Mental Health score. Each score is transformed to a 0-100 scale. The scores also are combined to provide a Physical Component (PCS) and Mental Component (MCS) Summary score. For the PCS and the MCS, norm-based scores are scaled and normalized to have a mean of 50 and a standard deviation of 10 based on the 1998 population norms.[83]

Absenteeism from Usual Activities
Background/Purpose: This questionnaire is designed to assess the number of days a participant has been kept from his usual activities (work, school or housework) because of LBP.[84,85]
Time points of administration: Laboratory Visit 1, Laboratory Visit 2, follow-up months 1-12
Procedure: Participants will answer each question by marking the answer as indicated.
Scoring: Quantified as number of days the participant did not participate in usual activities over the past 4 weeks. Range is from 0-28 days. Higher numbers indicate more days kept from activity/more affected by LBP.

Stanford Presenteeism Scale
Background/Purpose: This questionnaire is designed to assess how a participant's LBP affected his participation in usual activities and the impact of LBP on his ability to do his job over the past 4 weeks.
Time points of administration: Laboratory Visit 1, Laboratory Visit 2, follow-up months 1-12
Procedure: Participants will answer each question by marking the answer as indicated.
Scoring: Presenteeism is indexed in 3 ways. First, a Work Impairment Score (WIS) is calculated as the sum of answers on the questions regarding how LBP has affected job ability over the past 4 weeks. Each of the WIS questions is ranked on a 5-point Likert scale. All scores are 1-5, with questions 2, 5, 6, 8, and 10 reverse scored (5-1). The score ranges from 10-50 with 50 indicating the highest degree of impairment. Second, a Work Output Score (WOS) is quantified using the participant's estimate of the percentage of his usual productivity level during work over the past 4 weeks (1-100%). Third, a Work Absenteeism Score (WAS) is quantified using the participant's response to the number of work hours (1-40+) he missed in the past 4 weeks.[86]

Current Medication Use for LBP
Background/Purpose: This questionnaire is designed to provide general information on the use of medication for a participant's LBP.
Time points of administration: Laboratory Visit 1, Laboratory Visit 2, follow-up months 1-12

Procedure: Participants will answer each question by marking if they are taking non-prescription medication and prescription medication for their LBP. If yes, participants will indicate which medications they are taking and how many pills per day they are taking. For Laboratory Visit 1 and Laboratory Visit 2, the participant will answer regarding medications he is currently taking. At follow-up months 1-12, the participant will answer regarding the past 4 weeks.

Health Professional Care Seeking for LBP
Background/Purpose: This questionnaire is designed to provide general information on the use of additional treatments for a participant's LBP.
Time points of administration: Laboratory Visit 1, Laboratory Visit 2, follow-up months 1-12
Procedure: Participants will answer each question by marking yes or no for a list of other healthcare professionals they are seeing for treatment of their LBP. At Laboratory Visit 2 and follow-up months 1-12, if participants mark yes for a healthcare professional, they are subsequently asked how many times they saw that healthcare professional in the past 4 weeks.

Equipment Use for LBP
Background/Purpose: This questionnaire is designed to provide general information on the use of equipment for a participant's LBP.
Time points of administration: Laboratory Visit 1, Laboratory Visit 2, follow-up months 1-12
Procedure: Participants will answer each question by marking yes or no for a list of equipment they are using to treat their LBP. At Laboratory Visit 1 and Laboratory Visit 2 participants will answer regarding equipment they are currently using. At follow-up months 1-12, participants will answer if they obtained additional equipment in the past 4 weeks.

Satisfaction with Care
Background/Purpose: This questionnaire assesses information about how satisfied a participant feels with the physical therapist and treatment he was provided.
Time points of administration: Laboratory Visit 2
Procedure: Participants will answer each question by marking the answer as indicated. They may only check off one answer per item.
Scoring: Each question is ranked on a 5-point Likert scale. All scores are 1-5, with questions 1, 3, 4, 8, 9, 10 and 13 reverse scored (5-1). The total score is the sum of all of the answers (15-75).

Adherence to Home Program
Background/Purpose: This questionnaire is designed to provide information about how often a participant performs his treatment as it was prescribed. For the MST participants, the adherence is a combination of their prescribed exercises and the amount they applied the principles learned in treatment and performed the activities as prescribed across their day. For the SFE participants, the adherence is a combination of their prescribed strengthening and flexibility exercises.
Time points of administration: Clinic Visits 2-6, follow-up months 1-12, Booster Sessions 2-3
Clinic Visits (Daily Adherence)
Procedure: Participants will use a VAS to indicate for each day the percentage of the treatment they were able to perform as prescribed. At each clinic visit the therapist will ask the participant to provide an estimate of the average percentage of the treatment he was able to perform as prescribed in the interval of time between 2 clinic visits.
Scoring: Average adherence is calculated by averaging the participants' daily adherence. Scores range from 0-100%**.** Higher values indicate higher adherence to treatment.[64]
Follow-up months 1-12 (Monthly Adherence)

Procedure: Participants will use a VAS to indicate an average percent adherence to performance of the treatment as prescribed over the past month.
Scoring: Value (percentage of treatment performed) provided by the participant on each monthly survey. Scores range from 0-100%. Higher values indicate higher adherence to treatment.[64]

Demographics and LBP and Medical History
Background/Purpose: This questionnaire is designed to provide general information about the participant's demographics, LBP and medical history.
Time points of administration: Laboratory Visit 1
Procedure: Participants will provide information about each of the following items: age, handedness, gender, race, ethnicity, occupation, employment situation, marital status, education, LBP history, medical history.

Fear-Avoidance Beliefs Questionnaire (FABQ)
Background/Purpose: This questionnaire is designed to assess a participant's fear of pain and beliefs about how work and physical activity affect his LBP.
Time points of administration: Laboratory Visit 1, Laboratory Visit 2, Laboratory Visit 3
Procedure: Participants will answer each question by marking the answer as indicated.
Scoring: Each question is ranked on a 7-point Likert scale (0-6). Higher scores indicate higher fear-avoidance**.** Two subscale scores are calculated. The physical activity subscale score (FABQ-PA) is the sum of questions 2, 3, 4, and 5 and ranges from 0-24. The work subscale score (FABQ-W) is the sum of items 6, 7, 9, 10, 11, 12, and 15 and ranges from 0-42**.**

Treatment Preference Assessment Measure (TPA)
Background/Purpose: This questionnaire is designed to provide information about a participant's treatment preferences and the participant's perceptions of four attributes of each treatment: effectiveness, acceptability, suitability/appropriateness, and convenience. The questionnaire is modified from a preference questionnaire designed by Sidani et al[87] to reflect the treatments provided in the trial.
Time points of administration: Laboratory Visit 1, before randomization
Procedure: The two treatment descriptions will be given in a random order to each participant. First, the RA will inform the participant that she is interested in learning about his perception of each treatment. Second, the RA will read the description of the first treatment option. The RA will read the description slowly, clearly, and in an unbiased manner, in order to facilitate understanding. Third, the RA will ask the participant to rate the attributes of the treatment option just described. Fourth, the RA will repeat the three steps as they relate to the second treatment option. Fifth, the RA will ask the participant if he has a preference for either of the two treatment options, and if so, which one.
Scoring: The four treatment attributes (effectiveness, acceptability, suitability/appropriateness, and convenience) will be rated on a 5-point Likert scale (0-4)**.** A total scale score for each treatment will be computed as the mean of the four attribute ratings. Whether the participant prefers one of the two treatments will be recorded as yes/no. If the participant has a preference, then his preferred treatment will be recorded as MST or SFE.

9.4a Time Table for Administration

Measures	Laboratory Visit 1 (Baseline)	Clinic Visits 1-6	Laboratory Visit 2 (Post-Treatment)	Follow-up months 1-12 (Web-Based)	Laboratory Visit 3 (Follow-Up Month 6)	Booster Session (At Month 6)

MODQ	X	X (paper)	X	X		X (paper)
NRS (average & worst in prior 7 days)	X	X (paper)	X	X		X (paper)
Acute Flare-Ups of LBP	X			X (12 month only)	X	
LBP Recurrences	X			X (12 month only)	X	
SF-36	X		X	X (12 month only)	X	
Absenteeism from Usual Activities	X		X	X		
Stanford Presenteeism Scale	X		X	X		
Use of LBP-related medications & Additional treatment for LBP	X		X	X		
Satisfaction with care			X			
Adherence to Home Program		X (paper)		X (electronic)		X (paper)
Demographics/LBP & medical history	X					
FABQ	X		X		X	
TPA	X					
Contact Information	X					
Email Confirmation			X		X	
Clinical examination to classify LBP	X		X		X	
Clinic visit documentation		X				X

10. Data Management

10.1 Data Management Overview

The biostatistics team will oversee all activities. The PI and the Research Biostatistician will supervise data management. The research biostatistician will prepare web-based forms for data entry and editing, oversee data entry, write programs for data validation, manage the security and privacy of the electronic forms and data sets, prepare data sets for analysis, create progress reports, and provide for regular, secure back up of data. We will use a secure database created using Research Electronic Data Capture (REDCap) tools hosted by Washington University Biostatistics.

10.2 Sample Size Estimate

A power analysis[88] was conducted to determine adequate sample size for detecting a minimally important clinical difference of 6 on the MODQ.[89] Because power procedures for HLM models are not yet well developed, we used a multiple regression approach,[90] a reasonable alternative given both HLM and multiple regression are similar in form and intent. Furthermore, we took the conservative approach of powering the study for detection of a difference within a given time

point. A study adequately powered to detect within-time differences will then have equal or greater power to detect differences over time to the extent that measures over time are correlated. This is a reasonable assumption, and in fact, analyses of data from our prior trial indicate correlations among the MODQ scores collected at different times averaged .49. The calculations based on a single time period thus provide a conservative estimate and “worst case” approach to determining sample size. [90] The power analysis assumed 1) the basic 4 group (Treatment x Booster) design (3 df), 2) inclusion of 3 additional predictors (e.g., age, adherence, classification; 3 df), 3) an interaction component reflecting how the effect of treatment could depend on other predictors (9 df), 4) attrition of 20%, 5) a .05 level of significance (two-tailed), and 6) minimum power of .80. The chosen power value is the common standard used in most power analyses. Using the target effect of 6 on the MODQ and variability estimates from the prior trial, the power analysis indicated that 154 participants would need to be enrolled. Note that although examining moderation of treatment impact by LBP classification is not a specific aim, we will be able to explore such moderation by including dummy codes representing classification as additional predictors in the model. Note also that there are a large number of potential moderators that could be included in the statistical model, but we made provisions for including 3 additional predictors in any given analysis because of the likely intercorrelations that will exist among candidate predictors. Preliminary analyses will identify relative independent predictors or create composite predictors from highly correlated measures.

10.3 Analyses

Data analyses will proceed in several stages. <u>Stage 1</u>. Preliminary analyses will examine the distributional characteristics of variables to detect the presence of outliers or the need for transformations. Resampling procedures (e.g., bootstrapping) will be used if appropriate transformations are not possible.[91-93] Basic descriptive statistics for each measure and the inter-correlations among variables will provide a simple initial view of the sample characteristics. Similarly, simple univariate analyses (e.g., t-tests, chi-square) will be used to examine simple group differences. Major analyses to examine the specific aims will use hierarchical linear modeling, described in detail later. <u>Stage 2</u>. To ensure that subjects are representative, the baseline characteristics (e.g., age, gender) of the sample will be compared to those eligible to participate in the study, but who refused to participate. <u>Stage 3</u>. To determine if the randomization scheme provided a balanced sample, the baseline sociodemographic and medical characteristics will be compared *across* treatment groups. These comparisons will be made in 2 ways to reflect the 2-stage nature of the treatment. One set of analyses will examine differences between subjects randomly assigned to the 2 treatment groups following collection of baseline measures. A 2nd set of analyses will compare subjects that reach the booster phase of the study. These analyses will examine differences between subjects randomly assigned to the 2 booster conditions on measures collected prior to the booster randomization. The prior assignment to treatment condition will also be considered in this 2nd set of analyses. If random assignment to booster groups is balanced there should be neither significant main effect differences between booster conditions nor any significant Treatment Group x Booster Group interactions. <u>Stage 4</u>. Because of the large number of outcome measures, steps will be taken to control inflation of the Type I error rate. Primary hypotheses will be tested with an uncorrected alpha level of .05, as is warranted by their *a priori* nature.[94] Other hypotheses will be examined using Bonferroni corrected alpha levels. <u>Stage 5</u>. The primary analyses will be based on the intention to treat approach. In this approach, all subjects who are randomized to a particular treatment condition will be analyzed as part of that treatment group, regardless of subsequent events (e.g., lost to follow-up).[95] <u>Stage 6</u>. Missing data can be handled in a variety of ways (e.g., last value forward, multiple imputation), but each method has potential biases and makes assumptions about the underlying basis for the missing data and other features of the data that

are present. Without prior knowledge of the missing data process, we will explore the implications of applying different strategies.[96-98]

The major statistical approach for testing the Specific Aims will be hierarchical linear modeling (HLM).[99-101] This approach is well suited to longitudinal data that contain spacing of measures that can vary across subjects, missing data patterns that can vary across time and subjects, inclusion of time-varying covariates (e.g., adherence), and outcomes that can be either continuous (e.g., MODQ) or categorical (e.g., use of medication). These complexities limit the use of standard repeated measure analyses (e.g., ANOVA) that require fixed spacing of measures and complete data or are designed for particular kinds of outcomes (e.g., normally distributed continuous variables).

For longitudinal data, HLM models the data at 2 levels: change over time at the level of the individual, and, between-individual moderators of the change trajectories. These are referred to as Level 1 and Level 2 models respectively. At Level 1, the data for each individual is conceived as follows (using MODQ as the example outcome): $Oswestry_{ti} = \pi_{0i} + \pi_{1i} Time_{ti} + \pi_{2i} Time_{ti}^2 + e_{ti}$ In this model, π_{0i} is the intercept or level of the outcome when time is 0 (which depends on how the data are centered, described later) for person i, π_{1i} is the linear time parameter (at the centering point) and π_{2i} is the quadratic time parameter. Based on analyses from our recent trial, this basic quadratic model will be sufficient for estimating the time course of outcomes. Importantly, this basic model can be expanded to include other predictors that are collected over time, such as adherence, allowing investigation of interactions that could, for example, detect the differential effect of adherence over time on MODQ scores.

The Level 1 coefficients become the outcomes at Level 2 and are predicted by variables that vary across subjects. These include the treatment groups to which subjects are assigned as well as history and demographic variables that can be expected to influence the time course of outcomes. The Level 2 model can be illustrated as follows: For example, the model for π_{1i} tests whether the linear

$$\pi_{0i} = \beta_{00} + \beta_{01}(\text{Treatment})_i + \beta_{02}(\text{Booster})_i + \beta_{03}(\text{Treatment x Booster})_i + \beta_{04}(\text{Age})_i$$
$$\pi_{1i} = \beta_{10} + \beta_{11}(\text{Treatment})_i + \beta_{12}(\text{Booster})_i + \beta_{13}(\text{Treatment x Booster})_i + \beta_{14}(\text{Age})_i$$
$$\pi_{2i} = \beta_{20} + \beta_{21}(\text{Treatment})_i + \beta_{22}(\text{Booster})_i + \beta_{23}(\text{Treatment x Booster})_i + \beta_{24}(\text{Age})_i$$

change in outcome at the centering point differs from 0 (β_{10}), differs for subjects in the 2 treatment groups (β_{11}), differs for subjects in the 2 booster conditions (β_{12}), jointly influenced by treatment and booster conditions (the interaction, β_{13}), and varies as a function of age (β_{14}). Treatment group and booster group would be represented by dummy codes. The age predictor is simply illustrative of how predictors other than treatment can be incorporated into the Level 2 model. The other Level 2 equations are interpreted similarly, but refer to prediction of the Level 1 intercept (π_{0i}) or the Level 1 quadratic component (π_{2i}) and test the overall magnitude of the outcome or the departure from linearity, respectively. The latter is important, for example, in detecting whether the booster treatment can reduce the reversal of improvement that we observed at 12 months in our recent clinical trial.

The interpretation of the Level 1 intercept (π_{0i}) and Level 1 linear component (π_{1i}) depend on how the Level 1 data are centered. If, for example, time is centered at 6 months, then the intercept is the expected magnitude of the outcome at 6 months and the linear component is the rate of change at that time point (the tangent to the curve at the centering point). We will analyze the data at multiple centering points to allow full exploration of the nature of change over time and to detect treatment and booster condition differences at particular time points.

Specific Aim 1 will focus on the effects of treatment on outcomes (coefficients β_{01}, β_{11}, β_{21}) and examine how LBP (H_R1a), functional limitation (H_R1b), use of LBP medication/healthcare services (H_R1c), levels of adherence (H_R1d) and movement and alignment patterns (H_R1e) change over time and, in particular, as a function of treatment group. Specific Aim 2 will focus on booster group effects and examine how function and adherence are influenced by the booster treatment (coefficients β_{02}, β_{12}, β_{22}) and how this may be moderated by the type of treatment group to which participants are assigned (i.e., Treatment x Booster interactions, coefficients β_{03}, β_{13}, β_{23}). Specific Aim 3 will focus on two additional moderators, adherence and

movement and alignment patterns. These will be incorporated at Level 1 because they will be collected repeatedly. To add a measure of adherence would modify the model as follows: $Oswestry_{ti} = \pi_{0i} + \pi_{1i}Time_{ti} + \pi_{2i}Time_{ti}^2 + \pi_{3i}Adherence_{ti} + e_{ti}$ The coefficient, π_{3i}, then is modeled at Level 2 where the impact of treatment group can be investigated (H_R3a for adherence, H_R3b for movement and alignment patterns).

Economic analyses will focus on direct and indirect health costs. Direct health costs will be measured from the payer perspective. We will estimate the cost of medication using both the average wholesale price (AWP) and the wholesale acquisition cost (WAC) and assume that medication is used as prescribed. The cost of health services and medical equipment will be estimated using the Medicare allowable. Resources for which there is no AWP, WAC, or Medicare allowable will be estimated from a review of average retail prices. Analysis of the healthcare costs over time as a function of treatment will use the same HLM procedures proposed for our primary outcome measure, with the particular approach (e.g., generalized linear modeling) depending on the underlying sampling distribution (e.g., normal, Poisson). We will measure indirect health costs using absenteeism and presenteeism questionnaires. As with the measurement of expenditures, we will use HLM methods to evaluate the association between the treatment condition and days (or hours) lost (absenteeism and presenteeism will be evaluated separately) over time. As these data are counts, it is likely that a Poisson or negative binomial model will be necessary.

11. References

1. Frymoyer JW. Back pain and sciatica. *New England Journal of Medicine.* 1988;318(5):291-300.
2. Papageorgiou AC, Croft PR, Thomas E, Ferry S, Jayson MI, Silman AJ. Influence of previous pain experience on the episode incidence of low back pain: results from the South Manchester Back Pain Study. *Pain.* 1996;66(2-3):181-185.
3. Pizzo PA, Clark NM, Carter-Pokras O, et al. *Relieving Pain in America: A Blueprint for Transforming Prevention, Care, Education, and Research.* Washington, DC June 2011 2011.
4. Knauer SR, Freburger JK, Carey TS. Chronic low back pain among older adults: a population-based perspective. *J Aging Health.* 2010;22(8):1213-1234.
5. Sinnott P, Wagner TH. Low back pain in VA users. *Arch Intern Med.* 2009;169(14):1338-1339.
6. Croft PR, Macfarlane GJ, Papageorgiou AC, Thomas E, Silman AJ. Outcome of low back pain in general practice: a prospective study. *BMJ.* 1998;316(7141):1356-1359.
7. Wahlgren DR, Atkinson JH, Epping-Jordan JE, et al. One-year follow-up of first onset low back pain. *Pain.* 1997;73(2):213-221.
8. Hestbaek L, Leboeuf-Yde C, Kyvik KO, Manniche C. The course of low back pain from adolescence to adulthood: eight-year follow-up of 9600 twins. *Spine (Phila Pa 1976).* 2006;31(4):468-472.
9. Hestbaek L, Leboeuf-Yde C, Manniche C. Low back pain: what is the long-term course? A review of studies of general patient populations. *Eur Spine J.* 2003;12(2):149-165.
10. Hestbaek L, Leboeuf-Yde C, Engberg M, Lauritzen T, Bruun NH, Manniche C. The course of low back pain in a general population. Results from a 5-year prospective study. *J Manipulative Physiol Ther.* 2003;26(4):213-219.
11. Von Korff M. Studying the natural history of back pain. *Spine (Phila Pa 1976).* 1994;19(18 Suppl):2041S-2046S.
12. Martin BI, Deyo RA, Mirza SK, et al. Expenditures and health status among adults with back and neck problems. *JAMA.* 2008;299(6):656-664.

13. van Tulder M, Malmivaara A, Esmail R, Koes B. Exercise therapy for low back pain: a systematic review within the framework of the Cochrane collaboration back review group. *Spine (Phila Pa 1976).* 2000;25(21):2784-2796.
14. Hayden JA, van Tulder MW, Malmivaara A, Koes BW. Meta Analysis: Exercise therapy for treatment of non-specific low back pain. *Ann Intern Med.* 2005;142(9):765-775.
15. Van Middelkoop M, Rubinstein SM, Kuijpers T, et al. A systematic review on the effectiveness of physical and rehabilitation interventions for chronic non-specific low back pain. *Eur Spine J.* 2011;20(1):19-39.
16. Van Middelkoop M, Rubinstein SM, Verhagen AP, Ostelo RW, Koes BW, van Tulder MW. Exercise therapy for chronic nonspecific low-back pain. *Best Pract Res Clin Rheumatol.* 2010;24:193-204.
17. Hayden JA, van Tulder MW, Malmivaara A, Koes BW. Exercise therapy for treatment of non-specific low back pain. *Cochrane Database Syst Rev.* 2005;3:CD000335.
18. Liddle SD, Baxter GD, Gracey JH. Exercise and chronic low back pain: what works? *Pain.* 2004;107(1-2):176-190.
19. Hayden JA, van Tulder M, Tomlinson G. Systematic review: Strategies for using exercise therapy to improve outcomes in chronic low back pain. *Ann Intern Med.* 2005;142(9):776-785.
20. Macedo LG, Maher CG, Latimer J, McAuley JH. Motor control exercise for persistent, nonspecific low back pain: a systematic review. *Phys Ther.* 2009;89(1):9-25.
21. Rackwitz B, de Bie R, Limm H, von Garnier K, Ewert T, Stucki G. Segmental stabilizing exercises and low back pain. What is the evidence? A systematic review of randomized controlled trials. *Clin Rehabil.* 2006;20(7):553-567.
22. Chou R, Huffman LH. Nonpharmacologic therapies for acute and chronic low back pain: a review of the evidence for an American Pain Society/American College of Physicians clinical practice guideline. *Ann Intern Med.* 2007;147(7):492-504.
23. Hayden JA, van Tulder MW, Tomlinson G. Systematic review: strategies for using exercise therapy to improve outcomes in chronic low back pain. *Ann Intern Med.* 2005;142(9):776-785.
24. Henchoz Y, Kai-Lik SA. Exercise and nonspecific low back pain: a literature review. *Joint Bone Spine.* 2008;75(5):533-539.
25. Ferreira PH, Ferreira ML, Maher CG, Herbert RD, Refshauge K. Specific stabilisation exercise for spinal and pelvic pain: a systematic review. *Aust J Physiother.* 2006;52(2):79-88.
26. Croft P, Papageorgiou A, McNally R. Low back pain. *Health Care Needs Assessment*. New York, NY: Radcliffe Medical Press Oxford; 1997:129-182.
27. Slade SC, Keating JL. Trunk-strengthening exercises for chronic low back pain: a systematic review. *J Manipulative Physiol Ther.* 2006;29(2):163-173.
28. Liddle SD, David BG, Gracey JH. Physiotherapists' use of advice and exercise for the management of chronic low back pain: A national survey. *Man Ther.* 2008;14(2):189-196.
29. Waddell G. *The Back Pain Revolution.* Edinburgh: Churchill Livingstone; 1998.
30. Jordan JL, Holden MA, Mason EE, Foster NE. Interventions to improve adherence to exercise for chronic musculoskeletal pain in adults. *Cochrane Database Syst Rev.* 2010(1):CD005956.
31. Lechner DE. Work hardening and work conditioning interventions: do they affect disability? *Phys Ther.* 1994;74(5):471-493.
32. Poiraudeau S, Rannou F, Revel M. Functional restoration programs for low back pain: a systematic review. *Ann Readapt Med Phys.* 2007;50(6):425-429.

33. Schonstein E, D K, J K, B K, RD H. Physical conditioning programs for workers with back and neck pain: A cochrane systematic review. *Spine (Phila Pa 1976).* 2003;19(28):E391-E395.
34. Guzman J, Esmail R, Karjalainen K, Malmivaara A, Irvin E, Bombardier C. Multidisciplinary rehabilitation for chronic low back pain: systematic review. *BMJ.* 2001;322(7301):1511-1516.
35. Schaafsma F, Schonstein E, Ojajarvi A, Verbeek J. Physical conditioning programs for improving work outcomes among workers with back pain. *Scand J Work Environ Health.* 2011;37(1):1-5.
36. Schonstein E, Kenny DT, Keating J, Koes BW. Work conditioning, work hardening and functional restoration for workers with back and neck pain. *Cochrane Database Syst Rev.* 2010;CD001822(1).
37. Teasell RW, Harth M. Functional restoration. Returning patients with chronic low back pain to work--revolution or fad? *Spine (Phila Pa 1976).* 1996;21(7):844-847.
38. Guzman J, Esmail R, Karjalainen K, Malmivaara A, Irvin E, Bombardier C. Multidisciplinary bio-psycho-social rehabilitation for chronic low back pain. *Cochrane Database Syst Rev.* 2002(1):CD000963.
39. Conner JM, Culberson A, Packowski C, Chiba AA, Tuszynski MH. Lesions of the Basal forebrain cholinergic system impair task acquisition and abolish cortical plasticity associated with motor skill learning. *Neuron.* 2003;38(5):819-829.
40. Graybiel AM. The basal ganglia: learning new tricks and loving it. *Curr Opin Neurobiol.* 2005;15(6):638-644.
41. De Zeeuw CI, Yeo CH. Time and tide in cerebellar memory formation. *Curr Opin Neurobiol.* 2005;15(6):667-674.
42. Kleim JA, Swain RA, Armstrong KA, Napper RM, Jones TA, Greenough WT. Selective synaptic plasticity within the cerebellar cortex following complex motor skill learning. *Neurobiol Learn Mem.* 1998;69(3):274-289.
43. Karni A, Meyer G, Jezzard P, Adams MM, Turner R, Ungerleider LG. Functional MRI evidence for adult motor cortex plasticity during motor skill learning. *Nature.* 1995;377(6545):155-158.
44. Adkins DL, Boychuk J, Remple MS, Kleim JA. Motor training induces experience-specific patterns of plasticity across motor cortex and spinal cord. *J Appl Physiol.* 2006;101(6):1776-1782.
45. Nudo RJ. Plasticity. *NeuroRx.* 2006;3(4):420-427.
46. Classen J, Knorr U, Werhahn KJ, et al. Multimodal output mapping of human central motor representation on different spatial scales. *J Physiol.* 1998;512(Pt 1):163-179.
47. Hund-Georgiadis M, von Cramon DY. Motor-learning-related changes in piano players and non-musicians revealed by functional magnetic-resonance signals. *Exp Brain Res.* 1999;125(4):417-425.
48. Pascual-Leone A, Wassermann EM, Sadato N, Hallett M. The role of reading activity on the modulation of motor cortical outputs to the reading hand in Braille readers. *Ann Neurol.* 1995;38(6):910-915.
49. Kleim JA, Cooper NR, VandenBerg PM. Exercise induces angiogenesis but does not alter movement representations within rat motor cortex. *Brain Res.* 2002;934(1):1-6.
50. Remple MS, Bruneau RM, VandenBerg PM, Goertzen C, Kleim JA. Sensitivity of cortical movement representations to motor experience: evidence that skill learning but not strength training induces cortical reorganization. *Behav Brain Res.* 2001;123(2):133-141.
51. Sahrmann SA. Movement impairment syndromes of the lumbar spine. *Diagnosis and Treatment of Movement Impairment Syndromes*. 1st ed. St. Louis, MO: Mosby; 2002:51-118.

52. Hoffman SL, Johnson MB, Zou D, Van Dillen LR. Sex differences in lumbopelvic movement patterns during hip medial rotation in people with chronic low back pain. *Arch Phys Med Rehabil.* 2011;92(7):1053-1059.
53. Gombatto SP, Collins DR, Sahrmann SA, Engsberg JR, Van Dillen LR. Gender differences in pattern of hip and lumbopelvic rotation in people with low back pain. *Clin Biomech (Bristol, Avon).* 2006;21(3):263-271.
54. Scholtes SA, Gombatto SP, Van Dillen LR. Gender differences in timing of lumbopelvic movement during the clinical test of active knee flexion. *J Orthop Sports Phys Ther.* 2008;38(1):A69.
55. Van Dillen LR, Sahrmann SA, Engsberg JR, Lenke LG. Trunk rotation-related impairments in people with low back pain who participate in rotational sports activities. Paper presented at: 5th Interdisciplinary World Congress on Low Back Pain & Pelvic Pain; November 10-13, 2004; Melbourne, Australia.
56. Van Dillen LR, Sahrmann SA, Caldwell CA, McDonnell MK, Bloom NJ, Norton BJ. Trunk rotation-related impairments in people with low back pain who participated in two different types of leisure activities: a secondary analysis. *J Orthop Sports Phys Ther.* 2006;36(2):58-71.
57. Scholtes SA, Gombatto SP, Van Dillen LR. Differences in lumbopelvic motion between people with and people without low back pain during two lower limb movement tests. *Clin Biomech (Bristol, Avon).* 2009;24(1):7-12.
58. Norton BJ, Sahrmann SA, Van Dillen LR. Differences in measurements of lumbar curvature related to gender and low back pain. *J Orthop Sports Phys Ther.* 2004;34(9):524-534.
59. Van Dillen LR, Gombatto SP, Collins DR, Engsberg JR, Sahrmann SA. Symmetry of timing of hip and lumbopelvic rotation motion in 2 different subgroups of people with low back pain. *Arch Phys Med Rehabil.* 2007;88(3):351-360.
60. Hoffman SL, Harris-Hayes M, Zou D, Baxter KR, Tateuchi H, Van Dillen LR. Spinal and hip flexion end-range of motion with forward bending in two low back pain subgroups based on the Movement System Impairment model. *J Orthop Sports Phys Ther.* 2010;40(1):A76.
61. Hoffman SL, Harris-Hayes M, Zou D, Baxter KR, Tateuchi H, Van Dillen LR. Pattern of movement during return from forward bending in two low back pain subgroups based on the Movement System Impairment model. *J Orthop Sports Phys Ther.* 2010;40(1):A77.
62. Adams M, Bogduk N, Burton K, Dolan P. Functional pathology. *The Biomechanics of Back Pain*. New York, NY: Churchill Livingstone; 2002:168-172.
63. Fritz JM, Irrgang JJ. A comparison of a modified Oswestry Low Back Pain Disability Questionnaire and the Quebec Back Pain Disability Scale. *Phys Ther.* 2001;81(2):776-788.
64. Van Dillen LR, Norton BJ, Sahrmann SA, et al. Effects of classification specific treatment versus non specific treatment on low back pain related functional disability in people with chronic low back pain over a one year period. Paper presented at: XIX Biennial ISEK Congress; July 19-21, 2012; Brisbane, Australia.
65. Jette DU, Jette AM. Physical therapy and health outcomes in patients with spinal impairments. *Phys Ther.* 1996;76(9):930-941.
66. Jette AM, Smith K, Haley SM, Davis KD. Physical therapy episodes of care for patients with low back pain. *Phys Ther.* 1994;74(2):101-110.
67. Jette AM, Delitto A. Physical therapy treatment choices for musculoskeletal impairments. *Phys Ther.* 1997;77(2):145-154.
68. Organization WH. *Adherence to Long-Term Therapies: Evidence for action.* Switzerland 2003 2003.

69. Pisters MF, Veenhof C, van Meeteren NL, et al. Long-term effectiveness of exercise therapy in patients with osteoarthritis of the hip or knee: a systematic review. *Arthritis Rheum.* 2007;57(7):1245-1253.
70. Pisters MF, Veenhof C, de Bakker DH, Schellevis FG, Dekker J. Behavioural graded activity results in better exercise adherence and more physical activity than usual care in people with osteoarthritis: a cluster-randomised trial. *J Physiother.* 2010;56(1):41-47.
71. Friedrich M, Gittler G, Arendasy M, Friedrich KM. Long-term effect of a combined exercise and motivational program on the level of disability of patients with chronic low back pain. *Spine (Phila Pa 1976).* 2005;30(9):995-1000.
72. Friedrich M, Gittler G, Halberstadt Y, Cermak T, Heiller I. Combined exercise and motivation program: effect on the compliance and level of disability of patients with chronic low back pain: a randomized controlled trial. *Arch Phys Med Rehabil.* 1998;79(5):475-487.
73. Harris-Hayes M, Holtzman GW, Early J, Van Dillen LR. Development and preliminary reliability testing of an assessment of patient independence in performing a treatment program: standardized scenarios. *J Rehabil Med.* 2010;42(3):221-227.
74. Stanton TR, Latimer J, Maher CG, Hancock MJ. A modified Delphi approach to standardize low back pain recurrence terminology. *Eur Spine J.* 2011;20(5):744-752.
75. Nordin M, Weiser S, van Doorn JW, Hiebert R. Nonspecific low back pain. In: Rom WN, ed. *Environmental and Occupational Medicine*. 3rd ed. Philadelphia, PA: Lippincott-Raven Publishers; 1998:947-956.
76. Harris-Hayes M, Van Dillen LR. The inter-tester reliability of physical therapists classifying low back pain problems based on the movement system impairment classification system. *PM R.* 2009;1(2):117-126.
77. Van Dillen LR, Sahrmann SA, Norton BJ, Caldwell CA, McDonnell MK, Bloom NJ. Movement system impairment-based categories for low back pain: stage 1 validation. *J Orthop Sports Phys Ther.* 2003;33(3):126-142.
78. Trudelle-Jackson E, Sarvaiya-Shah SA, Wang SS. Interrater reliability of a movement impairment-based classification system for lumbar spine syndromes in patients with chronic low back pain. *J Orthop Sports Phys Ther.* 2008;38(6):371-376.
79. Van Dillen LR, Maluf KS, Sahrmann SA. Further examination of modifying patient-preferred movement and alignment strategies in patients with low back pain during symptomatic tests. *Man Ther.* 2009;14(1):52-60.
80. Farrar JT, Young JP, Jr., LaMoreaux L, Werth JL, Poole RM. Clinical importance of changes in chronic pain intensity measured on an 11-point numerical pain rating scale. *Pain.* 2001;94(2):149-158.
81. Childs JD, Piva SR, Fritz JM. Responsiveness of the numeric pain rating scale in patients with low back pain. *Spine (Phila Pa 1976).* 2005;30(11):1331-1334.
82. Von Korff M. Studying the natural history of back pain. *Spine (Phila Pa 1976).* 1994;19(18 Suppl):2041S-2046S.
83. Ware JE, Jr. SF-36 health survey update. *Spine (Phila Pa 1976).* 2000;25(24):3130-3139.
84. Von Korff M, Ormel J, Keefe FJ, Dworkin SF. Grading the severity of chronic pain. *Pain.* 1992;50(2):133-149.
85. Patrick DL, Deyo RA, Atlas SJ, Singer DE, Chapin A, Keller RB. Assessing health-related quality of life in patients with sciatica. *Spine (Phila Pa 1976).* 1995;20(17):1899-1908.
86. Turpin RS, Ozminkowski RJ, Sharda CE, et al. Reliability and validity of the Stanford Presenteeism Scale. *J Occup Environ Med.* 2004;46(11):1123-1133.
87. Sidani S, Epstein DR, Bootzin RR, Moritz P, Miranda J. Assessment of preferences for treatment: validation of a measure. *Res Nurs Health.* 2009;32(4):419-431.

88. Cohen J. A power primer. *Psychological Bulletin.* 1992;112(1):155-159.
89. Fritz JM, Irrgang JJ. A comparison of a modified Oswestry Low Back Pain Disability Questionnaire and the Quebec Back Pain Disability Scale. *Phys Ther.* 2001;81(2):776-788.
90. Cohen J, Cohen P. *Applied Multiple Regression/Correlation Analysis for the Behavioral Sciences.* 2nd ed. Mahwah, NJ: Lawrence Erlbaum Associates; 1983.
91. Chernick MR. *Bootstrap methods: a practitioner's guide.* New York, NY: Wiley; 1999.
92. Mooney CZ, Duval RD. *Bootstrapping: A Nonparametric Approach to Statistical Influence.* Pacific Grove, CA: Sage; 1993.
93. Hesterberg T, Monaghan S, Moore DS, Clipson A, Epstein R. *The Practice of Business Statistics.* New York, NY: W.H. Freeman; 2003.
94. Rosenthal R. *Constrast analysis: Focused comparisons in the analysis of variance.* New York, NY: Cambridge University Press; 1985.
95. Hollis S, Campbell F. What is meant by intention to treat analysis? Survey of published randomised controlled trials. *BMJ.* 1999;319(7211):670-674.
96. Collins LM, Schafer JL, Kam CM. A comparison of inclusive and restrictive strategies in modern missing data procedures. *Psychol Methods.* 2001;6(4):330-351.
97. Schafer JL, Graham JW. Missing data: our view of the state of the art. *Psychol Methods.* 2002;7(2):147-177.
98. Sinharay S, Stern HS, Russell D. The use of multiple imputation for the analysis of missing data. *Psychol Methods.* 2001;6(4):317-329.
99. Heck RH, Thomas SL. *An Introduction to Multilevel Modeling Techniques.* 2nd ed. New York, NY: Routledge; 2009.
100. Hox JJ. *Multilevel Analysis: Techniques and Applications.* 2nd ed. New York, NY: Routledge; 2010.
101. Raudenbush SW, Bryk AS. *Hierarchical Linear Models: Applications and Data Analysis Methods.* 2nd ed. Thousand Oaks, CA: Sage Publications; 2002.

Final Protocol and Statistical Analysis Plan

1. Background and Introduction

Significance

At least 60%-80% of adults will experience LBP,[1] and almost ½ of them will have had a major episode by age 30.[2] Chronic LBP is the most common type of chronic pain in adults[1,3] and its prevalence is increasing.[4,5] At least 75% of people who have a LBP episode, fail to recover fully within 1 year.[6] Pain persists as do limitations in simple movements and complex functional activities like self-care, social role, and work.[3] Recurrence rates are as high as 78%.[7] Thus, for many people LBP is a long-term, function-limiting condition rather than a short-term, self-limiting condition.[6,8-11] LBP-related costs are staggering. Estimated annual costs for LBP-related health-care are $30.7 billion and are increasing.[12]

Treatment of chronic LBP

Exercise is one of the primary non-surgical approaches used worldwide for managing LBP.[13-16] Based on systematic reviews, experts concluded that exercise is as efficacious, if not more efficacious for managing chronic LBP than 1) no treatment, 2) usual care, and 3) many other treatments, such as massage or laser therapy.[13,15-22] Experts also concluded that specific characteristics of exercise programs influence their efficacy[23] and that the most efficacious programs are 1) tailored to the individual, 2) delivered with supervision, and 3) designed to achieve high doses by emphasizing adherence. Despite the evidence for the beneficial effects of exercise in chronic LBP, several issues remain unresolved. First, little is known about which exercise is best for which patient.[15,22] There is no evidence that any particular type of exercise is clearly and consistently more efficacious than any other.[13-16,20-22,24,25] Many experts have suggested that differences between approaches have been missed because the heterogeneity of subjects in samples[13,15,16,20-22,26] and variability in the treatments studied have diluted the effects.[23,27] Second, when exercise is compared to non-exercise approaches, the effects tend to be small and inconsistent for outcomes like pain and function[14,20,22] and most of the significant effects are obtained only at the earliest follow-up.[14,22] Third, adherence and tests of the effects of adherence on outcomes are rarely examined.[16,18,21,23,28] Fourth, few studies examine the mechanisms underlying exercise effects,[20-22,25,27] so we do not know whether improvements in outcome with exercise are consistent with mechanism-based predictions for specific variables. The primary recommendation from review groups is that future studies should investigate the effect of individualized exercise strategies in well-defined populations of people with LBP.[14,21-24,27] The exercises should be aimed at restoring normal function and facilitating the person's performance of daily activities.[13,18,29] Furthermore, they recommend that future studies be designed to examine both the 1) specific mechanisms underlying the effect of exercise, and 2) effects of adherence on outcomes.[16,20-22,25,30] We will address all 3 of the recommendations.

Individualized exercises for restoring normal function and performance of daily activities

Given the profound impact of LBP on function,[3,6,12] a logical approach requires inclusion of explicit, individualized practice in performance of functional activities as a form of exercise. Interestingly, only physical conditioning programs, such as work hardening, work conditioning, and functional restoration include practice of activities as part of treatment.[31-36] However, these programs are primarily intended for people with disabling, work-related LBP. Thus, many of the activities that are practiced are 1) heavy work activities rather than routine functional activities,[32,33,36-38] and 2) not typically tailored to the individual. Finally, the programs tend to include a variety of co-interventions so the effect of any specific component is unclear.[32,35]

Neural mechanisms underlying the effect of exercise

Because both exercise and practice in performance of functional activities are types of motor training, studies in the motor training literature can inform our understanding of the mechanisms underlying their effects. Motor training induces structural and functional adaptations across a variety of motor regions of the nervous system.[39-43] In a review of the effects of skill, endurance, and strength training on the motor cortex and spinal cord, Adkins et al. emphasized the fact that the nature and location of adaptations are dependent on the specific behavioral demands of the motor experience.[44] Skill training is defined as the acquisition and refinement of novel combinations of motor sequences. Skill training results in synaptogenesis, synaptic potentiation, and reorganization of movement representations in the motor cortex.[44,45] Such adaptations are associated with the acquisition of new motor skills and vary with the degree of motor skill a person displays.[43,46-48] By contrast, strength and endurance exercises do not produce these adaptations.[49,50] If a goal of LBP rehabilitation is to aid acquisition, retention, and refinement of new motor behaviors to improve function, then the use of motor skill training is a necessity.

Model for development and course of LBP

The Kinesiopathologic Model (KPM) provides a conceptual basis for understanding the development and course of LBP. Specifically, LBP is proposed to be the result of repeatedly using direction-specific (flexion, extension, rotation, some combination) stereotypic movement and alignment patterns in the lumbar spine. The patterns begin as the result of an interaction between an individual's structural characteristics and the movements and alignments needed to perform daily functional activities.[51-57] Over time, the same patterns are used repeatedly for many different functional activities. The typical stereotypic pattern is characterized by the lumbar spine moving more readily in its available range in a specific direction(s) than the thoracic spine or hip.[57-61] Because the same patterns are used repeatedly, the loading is always on the same spinal tissue and tissue stress is concentrated in specific areas.[62] Repetitive loading of the same tissue could also alter the tissue's tolerance over time, accelerate the rate of mechanical injury, and potentially lead to tissue degeneration. Thus, the KPM describes a mechanism by which repetitive loading during performance of daily functional activities could contribute to the development and course of this often long-term, function-limiting condition.

Potential for individualized motor skill training to improve long-term course of LBP

In our recently completed clinical trial, we compared the effects of a classification-specific (Sp) treatment to a non-specific (NSp) treatment over a 12 month period. The Movement System Impairment (MSI) classification system that we used to classify subgroups of people with LBP is based on the KPM.[51] People in the various LBP subgroups exhibited direction-specific, stereotypic patterns of lumbar movement and alignment that were associated with their LBP. The Sp treatment included both exercise and training in functional activities directed at modifying the specific pattern(s) associated with the person's LBP. The NSp treatment included strength and flexibility exercise as well as training to maintain a neutral alignment of the lumbar spine during functional activities. We found that people in both the Sp and NSp groups had very large clinically meaningful improvements (61% at 6 mos) in pain and function[63] but differences between groups were smaller than expected.[64] We attributed the results to 3 related facts: 1) in addition to exercise, both groups received training to improve lumbar spine control during functional activities, 2) in both groups, adherence levels were higher and more prolonged for training in functional activities than for exercise, and 3) outcomes continued to improve for about 6 months after treatment but then gradually reversed along with declines in adherence to functional activity training. Changes in pain and function were much more strongly associated with adherence to functional activity training than with adherence to exercise. Thus, training in functional activities appears to be the essential active ingredient for improvement in both groups. Accordingly, the next important steps are to 1) disentangle the effects of training in functional activities and exercise, 2) maximize the effect by providing individualized motor skill

training during functional activities based on principles of motor skill learning, 3) prolong the treatment effects by providing a booster treatment at 6 months, and 4) enhance our understanding of the underlying mechanism by testing for predicted differences in the effects of the interventions on laboratory measurements of movement and alignment.

Impact on clinical practice

Currently rehabilitation for people with chronic LBP is highly variable and is usually focused only on treatment of acute recurrences.[28,65-67] Successful completion of this project will provide specific recommendations for the use and timing of individualized motor skill training for functional activities that can be applied immediately in practice. Because the training is focused on how an individual can painlessly perform functional activities that are limited, our findings will provide clinicians with an approach to treatment that directly addresses the factors proposed to contribute to the costly, long-term course of LBP. Also because the treatment is associated with prolonged periods of high levels of adherence, and uses specifically timed, periodic booster sessions to reinforce behaviors that minimize the patient's symptoms and functional limitations, our findings will provide the clinician and the person with LBP a feasible approach to long-term management of the condition, rather than just treating acute recurrences. Such an approach to LBP care could have an important impact on the long-term course (i.e., persistent or recurrent symptoms) and associated health-care costs of chronic LBP.

Focus on a treatment associated with high levels of adherence

The level of adherence during long-term treatment for most chronic diseases is only ~50%.[68] Poor adherence equates with low treatment dosage and consequent diminution of treatment effects. Data from our clinical trial demonstrated that 1) adherence levels were higher and more prolonged for training in functional activities than for exercise, and 2) changes in pain and function were more strongly related to functional activity adherence than to exercise adherence. Thus, in the proposed project we are focusing on a treatment to which people are able to adhere at high levels for prolonged periods of time.

Booster phase to prolong initial treatment effects

The purpose of the booster phase is to prolong the initial improvements seen in our clinical trial. Others have tested the effect of booster treatments in people with musculoskeletal pain conditions, including chronic LBP.[69-72] The innovativeness of our approach lies in the basis for the criteria we use for determining when to intervene and how much treatment to provide. Rather than providing additional treatment arbitrarily, we have scheduled the booster phase to start just before the time when outcomes began to reverse in our prior trial. The criterion for the number of booster sessions (i.e., how much treatment) is the number needed to attain independence in performance of the treatment items.[73]

2. Specific Aims

Specific Aim 1: Test if motor skill training results in better outcomes and better adherence than strength and flexibility exercise in the 12 months after treatment.

Compared to the strength and flexibility exercise group, the motor skill training group will

Hypothesis 1a. report lower LBP intensity, fewer days with LBP, and less frequent LBP recurrences.

Hypothesis 1b. report fewer functional limitations as well as better perceived physical health and mental health, work attendance, and productivity.

Hypothesis 1c. use fewer LBP medications and fewer healthcare services.

Hypothesis 1d. report higher levels of adherence over longer periods of time.

Hypothesis 1e. display improved movement and alignment patterns during functional activities.

Specific Aim 2: Test the effect of a booster phase that is provided 6 months after the initial phase.

Hypothesis 2a. The effect of a booster treatment condition will be longer-lasting improvement in function and better adherence than a no booster treatment condition.

Hypothesis 2b. The effect of a booster treatment condition will be greater for the motor skill training group than for the strength and flexibility exercise group.

Specific Aim 3: Examine the relationship between functional limitations and 2 factors 1) adherence, and 2) movement and alignment patterns displayed during functional activities.

Hypothesis 3a. Adherence to motor skill training will be related more strongly to functional limitations than will adherence to strength and flexibility exercise.

Hypothesis 3b. Movement and alignment patterns displayed by the motor skill training group will be related more strongly to functional limitations than will patterns displayed by the strength and flexibility exercise group.

3. Approach

3.1 Design

The study is a single-blind, prospective, randomized, controlled clinical trial. Figure 1 illustrates the organization and flow of events. After we collect baseline self-report and laboratory measures, people will be randomized to 1 of 2 treatment conditions, motor skill training (MST) or strength and flexibility exercise (SFE). The initial phase will be 6, 1-hour treatment sessions, scheduled once/week for 6 weeks. Post-treatment self-report and laboratory measures will be obtained immediately after the initial phase. A select subset of web-based, self-reports will be obtained monthly for the next 12 months (9.4a Time Table for Administration). At 6 months after treatment, self-report and laboratory measures will be obtained again. Subsequently, people will be randomized to a booster treatment condition, booster or no booster. The booster treatment will be specific to the person's initial, condition-specific treatment assignment. The number of booster sessions will be limited to the number needed to attain independence in the exercises or functional activities prescribed. Independence will be based on a standardized measure used in our prior trial.[73] The 6-month time point was chosen based on change in the Modified Oswestry Disability Questoinnaire (MODQ) scores from our prior trial.

3.2 Subjects

We will recruit 154 people between the ages of 18 and 60 years who have had chronic, non-specific LBP for at least 1 year from the St. Louis metropolitan area. Chronic LBP is defined as pain present for at least half the days in a year in the region between T12 and the gluteal fold, with or without leg symptoms to the knee.[11,74] Non-specific LBP is defined as LBP in which no specific pathophysiologic mechanism can be identified to account for the person's perceived pain.[75] The goal of this study is to obtain a representative sample of people who have both symptoms and disability related to chronic LBP. The target participant population is people with chronic LBP, with or without recurrences. The goal is to examine the generalizability of the effects seen in the previous clinical trial to people who have more LBP-related limitation than people previously studied. This will be accomplished by recruiting participants from the Washington University School of Medicine Physical Therapy Clinic (WUPT Clinic) and local outpatient clinics and offices. Only participants who report a specific criterion level of disability and difficulty with a specific number of functional activities will be enrolled. Both genders and all races and ethnicities will be included in the study population.

3.3 Study flow chart

Figure 1

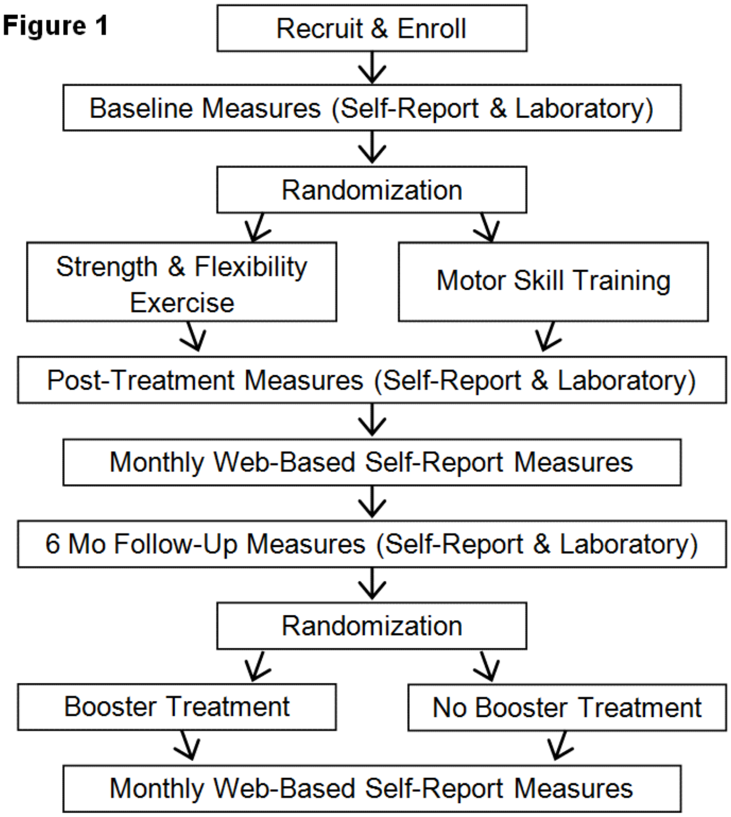

4. Enrollment of Participants

4.2 Inclusion Criteria

The inclusion criteria are 1) people ages 18-60 with chronic LBP for a minimum of 12 months, 2) currently experiencing LBP symptoms but not in a recurrence or an acute flare-up, 3) MODQ score of ≥ 20%, 4) 3 or more functional activities (simple or complex) limited due to LBP, 5) able to stand and walk without assistance, and 6) able to understand and sign a consent form.

4.3 Exclusion Criteria

The exclusion criteria are 1) BMI >30, 2) any structural spinal deformity including scoliosis, kyphosis, or stenosis, 3) spinal fracture or dislocation, 4) low back pain due to trauma, 5) osteoporosis, 6) ankylosing spondylitis, 7) rheumatoid arthritis, 8) current symptomatic disc herniation (i.e. diagnosis with symptoms below the knee), 9) spondylolisthesis, 10) serious spinal complications such as tumor or infection, 11) previous spinal surgery, 12) frank neurological loss, i.e., weakness or sensory loss, 13) pain or paresthesia below the knee, 14) etiology of LBP other than the lumbar spine, e.g., hip joint, 15) history of neurologic disease which required hospitalization, 16) active treatment for cancer, 17) history of unresolved cancer, 18) pregnancy, 19) worker's compensation or disability case, and 20) in litigation for the LBP problem, 21) fibromyalgia, 22) Marfan syndrome, 23) Graves' disease.

4.4 Recruitment

People with LBP will be recruited from the WUPT clinic, local clinics and offices, and local advertisements and health fairs. In all clinics, a list of the inclusion/exclusion criteria and

possible diagnoses will be available. Flyers will also be placed in all office areas and given to clinic staff for reference.

4.4a Clinics/Offices

Study flyers will be placed in all area clinics/offices that see potential LBP participants and are willing to assist with study recruitment. Patients that are being referred for physical therapy treatment of their LBP also will be asked if they are interested in the study. If they are interested, their information can be given to the research coordinator (RC) or research assistant (RA) or the patient can contact the RC or RA directly. If the person is not interested, the person will be scheduled as usual. The RA or RC will log everyone she contacts on the Telephone Screening Log. If the person does not qualify for the study, he will be told to schedule his appointment with the clinic. If a person exceeds three callbacks, he will no longer be contacted.

4.4b Clinic Enrollment Flowchart

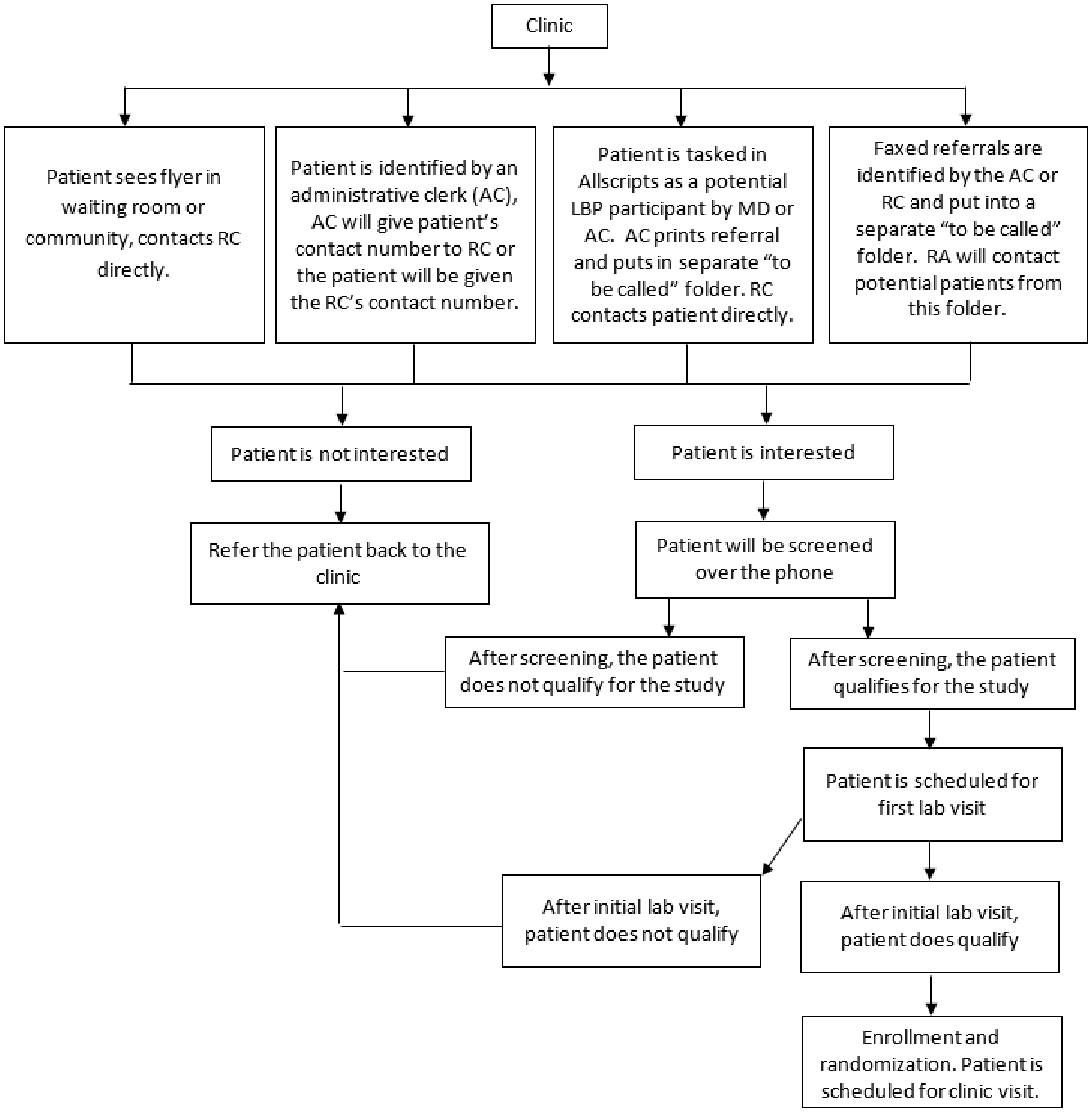

4.5 Initial Screening

The RA will speak with interested individuals via phone. The RA will briefly describe the trial and the procedures the person will participate in. Informed consent for a telephone screening will be obtained. The RA will then interview the individual using a series of screening questions to determine eligibility for the trial. If the person is deemed ineligible by phone screening, then the RA will explain the reason why he does not qualify (DQ). If the person is deemed eligible by phone screening, he then will be scheduled for a baseline laboratory visit where additional screening will be performed. All individuals who are screened will be entered into a database indicating their eligibility/ineligibility for the trial. Individuals who do not qualify at Laboratory Visit 1 because MODQ is <20% can be rescreened if LBP status has worsened.

4.5a Screening to Enrollment Flow Chart

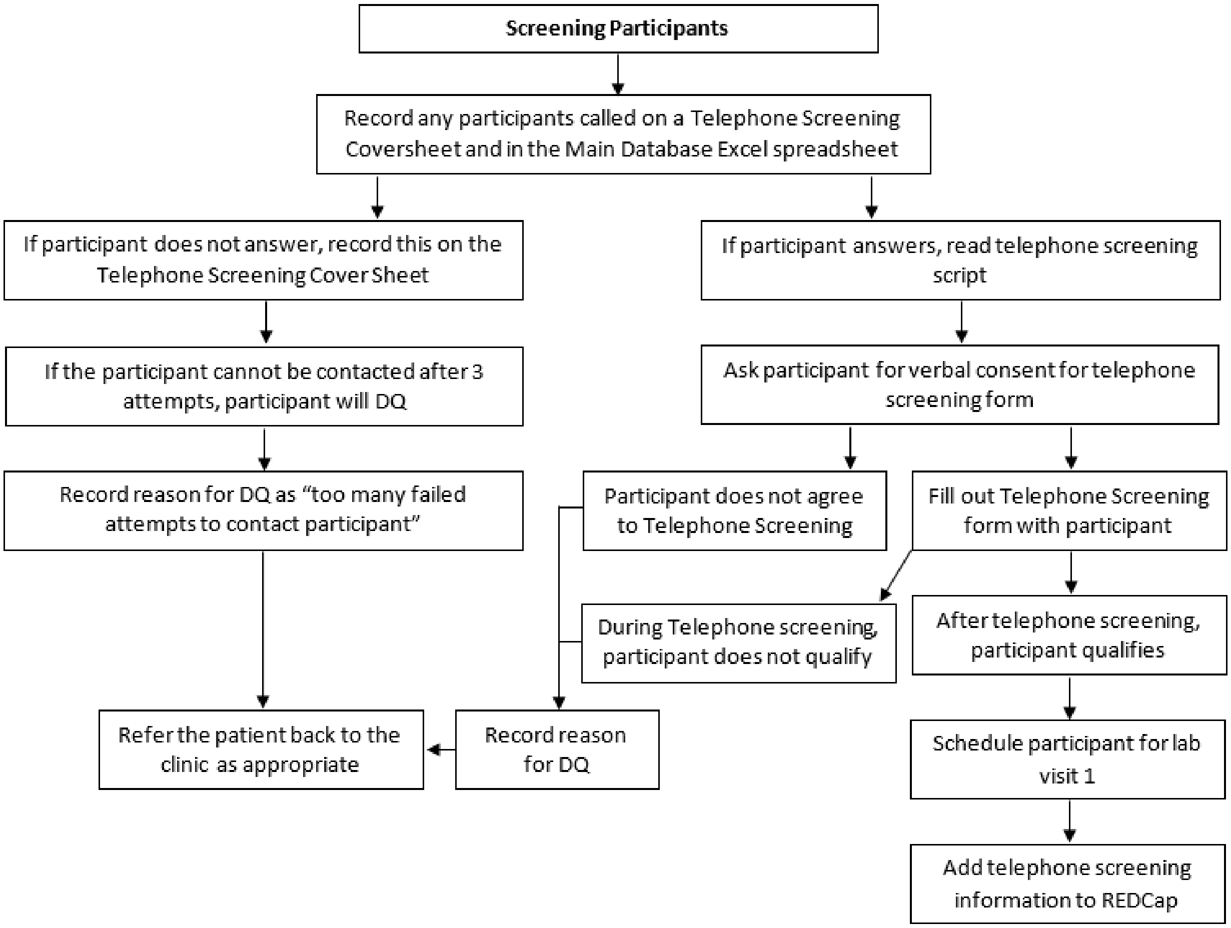

5. Laboratory Visits

5.1 Informed Consent

If the person is eligible, he will be scheduled to come in for examination with the Research Physical Therapist (RPT). On the first visit to the laboratory the Principal Investigator (PI), RPT, RC, or RA will explain the informed consent process to the person. The person will then be taken through the specific details on the informed consent form approved by Washington University's Human Research Protection Office (HRPO). In particular, the person will be told the

procedures he will participate in, the potential risks and benefits of participation, and his right to refuse participation at any time without any effect on his subsequent health care. The person then will be given an opportunity to read the consent form. The PI, RPT, RC, or RA will be available to answer any specific questions, highlight the important details of the study again, and remind the person of his rights as a participant. The person will then be asked to sign the consent form. A copy of the form and a brochure explaining the person's rights under the Health Insurance Portability and Accountability Act (HIPAA) regulations will be given to the person for his records. The signed consent form will then be signed by the person administering consent. The consent form will be filed in a locked cabinet separate from the person's data.

5.2 Baseline Examination/Laboratory Measures

Participants who are eligible for participation in the study and provide informed consent will be examined. At the initial visit, height and weight will be collected from each participant with clothing on and shoes off using a manual scale. Height will be collected to the nearest ¼" and weight will be collected to the nearest pound. The participant will then complete a Demographics and LBP history questionnaire, the Numeric Pain Rating Scale (NRS), and the MODQ. Participant eligibility of MODQ ≥ 20% score and at least 3 functional activities limited due to LBP will be confirmed. Next, the participant will be examined by a licensed, trained RPT who will use a standardized exam to classify the participant's LBP based on the MSI classification system.[51,76-79] The participant then will complete additional self-report measures and laboratory testing. Because all baseline testing occurs *before* randomization, research personnel involved in testing will be *blinded* to the participant's treatment assignment throughout the study. If a participant is in an acute flare-up when he or she comes in for a laboratory visit, the research physical therapist will determine if it is an actual acute flare-up. If it is determined to be an actual acute flare-up, the laboratory visit will not be completed and the participant will be rescheduled.

Laboratory Measures

Participants will perform functional activity tests that (1) parallel activities reported as problematic for the majority of participants in prior studies, and (2) have been examined previously. Retroreflective markers will be taped to the skin at predetermined locations on the participant's trunk, pelvis and legs. Data from 3 trials of each test will be captured. LBP will be recorded during each trial.

5.3 Randomization

The sample will be stratified based on LBP classification. After baseline assessment, a participant will be assigned randomly, within his strata, to either the (1) MST with no booster, (2) MST with booster, (3) SFE with no booster, or (4) SFE with booster condition. The purpose of stratification by LBP classification is to ensure similar representation of LBP subgroups in the treatment conditions and eliminate any confounding of classification and treatment. Note that it is not our goal to determine if LBP classification moderates treatment impact; there will not be a sufficient number of subjects in some LBP classifications to address that issue with adequate power. Randomization will be conducted using a computer-generated list of random numbers provided by the biostatistics team. The randomization scheme will be prepared *before* initiation of the trial and will be adhered to *throughout* the trial. Assignments will be performed within the REDCap system once the Screening & Randomization form is completed. When the participant with LBP is enrolled, and assigned a group, the RC will print the computer-generated group assignment and schedule the participant with the appropriate therapist for all 6 clinic visits. The first clinic visit should be scheduled within one week of the laboratory visit. The participant will not be blinded to treatment assignment, but will be blinded to his assignment to booster/no booster until completion of Laboratory Visit 3 (the 6 month visit).

5.4 Laboratory Visit 2 & 3

All measures and procedures used during Laboratory Visit 1 will be used again for the post-treatment follow-ups. All participants will be reminded not to discuss the details of their treatment with *blinded* research personnel so that blinding to treatment assignment is maintained. Laboratory Visit 2 will be scheduled within one week after discharge from the treatment phase. Laboratory Visit 3 will be scheduled within the 2 week period surrounding 6 months following Laboratory Visit 2.

6. Treatment Protocol

6.1 Overview

Both treatment groups will participate in a 1-hour treatment, once a week for six weeks. Each group will also be given a home program to perform during the entire 12-month study period. All treatments will be provided by licensed physical therapists trained in the respective treatments. The therapists cannot be blinded to treatment assignment, but are blinded to booster/no booster assignment until the participant completes Laboratory Visit 3. None of the therapists, however, will be involved in either (1) collecting baseline or follow-up data, or (2) laboratory meetings with research personnel. In the initial phase of treatment, both groups will be provided the same amount of time in treatment. Documentation of treatment prescription and progress is standardized (including harm). This documentation will be used to track treatment fidelity. Rules for prescribing and instructions for performing treatment items are standardized and documented; instructions with photos will be available in hard copy.

6.2 Motor Skill Training (MST)

The motor skill training involves supervised, massed practice of novel, challenging functional activities. The overarching principle of training is that the participant needs to practice performing the functional activities in ways that (1) minimize use of the participant's specific, stereotypic lumbopelvic movement and alignment patterns (based on the participant's LBP classification), and (2) encourage use of other joints (thoracic spine, hips, knees) to complete the activity painlessly. The lumbopelvic patterns related to the participant's specific LBP classification identified during the laboratory examination (flexion, extension, rotation, extension & rotation, or flexion & rotation) will be targeted. We will use information about the participant's LBP classification to guide the training because (1) our data suggest there is added benefit to classification-based training, (2) a meta-analysis of treatment studies showed that individualized treatment provides added benefit to outcome, and (3) there is evidence supporting the existence of LBP subgroups. Participants will be educated that the primary contributors to their LBP symptoms are repeated movements and prolonged positions of the lumbopelvic movement patterns related to their specific LBP classification. Additionally, participants will be educated that the emphasis of treatment is to learn how to modify or change how they perform their daily activities so that they do not use these patterns repeatedly throughout the day. During practice, the participant will be given cues for using the trunk muscles needed to facilitate a correct lumbopelvic pattern. Thus, rather than prescribing exercises for individual trunk muscles, we will attempt to modify how the participant uses multiple trunk muscles within the context of the activity in which they will be used. Such an approach should facilitate the use of these muscles in daily functional activities and contribute to enhanced adherence.

Specific activities are selected for a participant to practice if performing them is painful or difficult because of his LBP. To enhance salience, the participant will be involved in selection of the activities to be practiced. In addition, a pre-determined set of activities will be included for all participants because we know from prior studies that those activities are difficult for most people with chronic LBP to perform. The set of activities parallels the functional activities that will be

tested in the laboratory. Three activities will be practiced for ~15-20 minutes each. Rest will be provided, as requested. All activities will have 3 essential components: (1) contraction of groups of specific trunk muscles, (2) earlier and greater movement of the hip, knee, and/or thoracic spine relative to the lumbar spine, (3) later and less movement of the lumbar spine relative to other regions. The muscles that need to be activated and the directions of movement and alignment that need to be modified will be specific to the participant's LBP classification. Within each activity, the conditions of practice will vary. For example, sit ↔ stand will involve practicing with various (1) seat surface heights, (2) seating materials, and (3) constraints of surroundings. The order in which activities are practiced will be randomized. All participants will practice simple activities (e.g., moving in bed) before complex activities (e.g., gardening). The level of difficulty of the activity will be graded to match the motor capabilities of the participant and progressed based on ability to (1) verbalize the key concept for that skill (2) appropriately modify the movement or alignment and (3) control LBP symptoms during performance of 5 repetitions of the skill. We use a highly reliable measure to assess these characteristics and decide if the participant can be progressed to a higher level of difficulty.[73] Participants need to practice activities without reproduction of their LBP for three reasons. First, in experimentally-induced pain conditions, researchers have shown that motor learning and related neuroplasticity can be hindered. Second, if the stereotypic patterns do contribute to the participant's LBP, modification of the patterns should alleviate the LBP. Inability to perform the activity without pain could mean the pattern has not been fully modified. Third, increased pain levels during exercise are associated with poor adherence. A home program will be prescribed of 1-2 sets of 5-10 repetitions of formal practice of the activities practiced in treatment sessions.

An abdominal brace or tape can be used with any skill as indicated based on the participant's LBP classification and severity level. The use of a brace or tape is optional. Performing the skill without the brace or tape would make the skill more difficult. The decision to use or not use a brace or tape can be used in grading the skill. Some participants may need to always use a brace or tape for performance of specific skills. If the patient sits for prolonged periods of time, has anatomy that makes him vulnerable to poor positioning or any other factors that would contribute to making adherence to the positioning recommendation difficult, providing positioning devices is appropriate and should be considered.

6.3 Strength and Flexibility Exercise (SFE)

Exercises for trunk strength and trunk and lower extremity flexibility commonly cited in the literature as being appropriate for people with chronic LBP and included in the previous trial will be used. The overall goals of treatment will be to increase the participant's trunk strength and trunk and lower limb flexibility; if necessary, equipment will be provided. Participants will be educated on possible contributors to pain, including poor posture, stress, loss of strength and flexibility, and general decline in physical fitness. The therapist will be blinded to the LBP classification to avoid any potential bias in the prescription of exercise or education, and participants will not be educated about their LBP classification. Additionally, participants will be educated about the benefits of strength and flexibility exercises and the expectation that performance of these exercises will improve performance in daily activities limited by LBP. Participants will be instructed that muscle soreness is expected with performance of new exercises.

Strengthening exercises will target all trunk muscles. Flexibility exercises will target all trunk and lower limb motions. Treatment is divided into 3 phases with each phase being progressively more difficult. All participants will start with Phase 1 exercises regardless of strength or previous experience with back pain exercises. All participants will be instructed to perform a home program of stretching every other day and strengthening every other day. At each treatment

session, the therapist will assess the participant's independence in performance of the exercises and if the participant has met the criteria to advance to the next phase. Progression of exercise will be based on independence in: knowledge of the key concept (purpose of the exercise), correct performance without cues, and performance of the maximum number of repetitions of each of the exercises (4 repetitions of 30 second holds for stretching and 3 sets of 10 repetitions for strengthening).

6.4 Training and monitoring of clinicians providing treatment

Clinicians administering treatment in both conditions will be trained before data collection begins. SFE clinicians will be trained by the PI. MST clinicians will be trained by the PI and a co-investigator with expertise in motor learning. Clinicians will be provided with training manuals describing the treatment procedures in detail for each condition. Clinician proficiency in delivering the treatment will be assessed with practical examinations administered yearly by the co-investigator (MST) or the RPT (SFE). Clinician knowledge of treatment protocols will be assessed yearly with written examinations. Treatment fidelity will also be monitored by regular review of clinician documentation. The PI will hold monthly meetings with the treating clinicians in each condition to discuss any questions about the treatment protocol or implementation. The treating clinicians will not discuss any participants' names or other identifying information during these meetings to maintain blinding.

7. Treatment Visit Procedures

7.1 Clinic Visit 1

Duration

The initial treatment is 1 hour in duration. This does not include the time the participant is completing outcome measures or the therapist's documentation of the visit. It is acceptable if the participant requires more time this first visit for initial education and instruction, the therapist should record the amount of time spent in treatment on the Clinic Visit 1 form.

Before the participant arrives for his first visit, the therapist should review the participant's Clinic Visit 1 folder that includes all of the information from Laboratory Visit 1. This includes the following forms: (1) Medical Screening, (2) Demographics, LBP and medical history, (3) NRS, (4) SF-36, (5) MODQ, (6) Absenteeism & Presenteeism, (7) FABQ, and (8) Clinical Examination Data (History and Physical). The clinical examination data related to a participant's LBP classification is deleted from the forms for the clinicians providing the SFE treatment. This is done to maintain blinding of a clinician to a participant's LBP classification. There should also be a Clinic Visit 1 form and a Treatment Progress Table appropriate to the participant's treatment group. The therapist will receive an additional folder that includes all of the paperwork for the follow-up clinic visits.

Initial Interaction

While the participant is waiting for treatment to be initiated, the therapist should give the participant the following forms to complete: (1) the NRS, and (2) the MODQ. The participant's progress since Laboratory Visit 1 should be reviewed. The therapist will then (1) provide the overview of the trial and the specific treatment condition and (2) explain the overall goals of the treatment condition.

Treatment

Treatment during Clinic Visit 1 will consist of (1) an explanation of Educational Concepts/Principles, and (2) treatment based on the treatment condition assignment. At the end of Visit 1 the participant is provided with (1) handouts for initiation of the home exercise program

(HEP), (2) the Daily Treatment Adherence Log to be filled out during the days between visits, (3) an explanation of how to the complete the Daily Treatment Adherence Log, and (4) the therapist's contact information in case the participant needs to reschedule a visit, has any questions, or experiences a worsening of status. The therapist records the events of Visit 1 in the participant's chart on the Clinic Visit 1 documentation form and Treatment Progress Table. The time spent in treatment should be recorded on the Clinic Visit 1 documentation form.

7.2 Follow-up Clinic Visits

All treatment visits should be 1 hour in duration. This does not include the time the participant is completing outcome measures. The time spent in treatment should be recorded on the Clinic Follow-Up Visit documentation form. The participant should come to the reception area of the clinic. The therapist should take the participant to a clinic treatment room upon arrival to keep the participant from spending time in the reception area where discussion of treatment with other participants may occur, potentially contributing to contamination of treatment. At this time the therapist should provide the participant with the (1) NRS, (2) MODQ, and (3) Educational Principles quiz to be completed before treatment is initiated. The therapist reviews the NRS, MODQ, Daily Treatment Adherence Log and Educational Principles quiz as well as events since the last visit. The participant's questions are answered and treatment is reviewed and progressed. Education is focused on principles not understood based on quiz performance. The majority of time should be spent on the exercises. The participant is given updated handouts of the HEP as needed, a Daily Treatment Adherence Log, and is reminded of the next visit date and time. The therapist records the events of the visit in the participant's chart on the Clinic Follow-Up Visit documentation form (including the time spent in treatment) and the Treatment Progress Table.

Each clinic visit needs to be at least 5 calendar days to no more than 2 weeks after the previous clinic visit. Clinic Visit 1 can be scheduled within two weeks of Laboratory Visit 1.If a participant is up to 30 minutes late to his/her clinic visit and the clinician cannot treat for the full hour, he/she should treat for 30 minutes and schedule an additional treatment visit as soon as possible for the additional 30 minutes. The clinician should also inform the PI within 24 hours of the late visit. Clinic visits should be split no more than 2 times, for a maximum of 8 clinic visits for a participant. If the participant is more than 30 minutes late, it is the discretion of the therapist as to whether the visit will be completed or rescheduled. Attendance scripts will be reviewed with participants for Clinic Visit 1 and Clinic Visits 2-6. These scripts will guide the clinicians to discuss with the participant the importance of scheduling visits and adhering to the schedule

7.3 Final Visit

On the final visit, the participant's HEP should be reviewed so that the participant knows what he is expected to continue upon discharge. The therapist should encourage the participant to continue to be active and continue with exercises regularly as prescribed. The therapist should make sure the participant has scheduled his follow-up laboratory visit. The participant should be reminded that he will be receiving monthly follow-up surveys via email which he will be paid to complete. The therapist should provide his current contact information if the participant has questions or has a status change after discharge. The participant's follow-up laboratory visit should be within two weeks after discharge from the treatment phase.

7.4 Booster Phase

The booster phase will occur 6 months after discharge from the initial treatment. Participants will be randomized to a booster condition (their original condition-specific treatment) or a no booster condition at the initial laboratory visit. The participant is told his assignment to boosters

(booster/no booster) at completion of Laboratory Visit 3 (the 6 month visit). Each booster session will be 1 hour. The content of the booster treatment will consist of review/practice of the home program from the initial treatment phase. The number of sessions will be based on the number needed to attain independence in the prescribed treatment items, with a maximum of 3 visits. Participants will see the same treating therapist during the treatment phase and all booster sessions. If a participant is randomized to the booster condition, the RPT will contact the referring provider and inform him/her that the participant will be receiving additional treatment sessions.

7.5 Reporting Worsening of Status and Procedures

For both MST and SFE, a worsening of symptoms is defined as an increase in the intensity of LBP symptoms or peripheralization of symptoms (symptoms located more laterally and/or distally from the original location) that lasts for greater than 4 hours after performance of the treatment items and has not fully resolved after 24 hours. The participant should call the therapist if there is a worsening of status (worsened symptoms or red flags are present) or if the participant is concerned for any reason about his status. If the participant reports a worsening of symptoms (as operationally defined), the therapist should discuss the possible causes of worsening such as a change in activity level, specific activities, work conditions, sleeping habits, etc. If worsening appears to be caused by the HEP the participant should be instructed to stop exercising for 24 hours and encouraged to use pain relieving techniques. These include using medications as prescribed by his physician, and application of cold if within 48 hours of worsening or heat if greater than 48 hours since worsening. After the 24 hours has passed, the participant should begin the exercises/skills being practiced, starting with 1 exercise/skill the first day. The participant should add 1 exercise/skill per day and report to the therapist if any specific exercise/skill increases his symptoms. In the SFE condition an increase in symptoms during exercise is not contraindicated and the participant should continue. If a particular exercise/skill appears to cause a worsening, the participant should be instructed not to perform that specific exercise/skill until the next visit. Performance will then be reviewed and modified as needed. If the participant feels that certain exercises/skills are worsening his symptoms, these should be stopped until the next visit. These, however, typically would not be expected to worsen the participant’s status if performed correctly, so these exercises should specifically be reviewed for correct performance at the treatment visit. If red flags are present, the participant should contact his physician for follow-up and contact his therapist once he has communicated with the physician. The therapist should complete the adverse event form and follow the procedure for reporting an adverse event.

7.6 Adherence/Non-Adherence During Treatment Period

All participants should be encouraged to actively participate in their treatment program across the 6 visits as well as after discharge. If the participant is having difficulty adhering, the therapist should discuss strategies for adhering with treatment. If adherence becomes a problem, the therapist should discuss this in the regularly scheduled meetings with the PI or contact her directly to discuss strategies, making sure to not identify the participant so that blinding can be maintained.

8. Human Protection (HRPO)

8.1 Charts

All paperwork in charts will have the participant’s ID number. No names or identifying information will be on the paperwork or the chart when the participant’s chart is filed away for the day. In the instance there is a name provided, the treating therapist should mask the name

by crossing out with a black marker. Charts will be kept in a locked file drawer that is only accessible to the therapist treating the participant.

8.2 Visit Documentation

All documentation for each visit will be completed the day of the visit. This includes the clinic visit form and the Treatment Progress Table. Chart audits will be conducted throughout the study period to ensure adherence to the protocols and complete data sets per participant per measure. For this reason, the charts should always be up to date and locked in the designated filing area.

8.3 Blinding, Contamination, and Privacy Issues

Blinding

There can be no discussion of participants' names when interacting with research personnel. All research personnel are to remain blinded across the study period to all participants' treatment condition assignment. Research personnel include the following: (1) the Principal Investigator, (3) the Research Physical Therapist, and (3) the Research Assistant. All paperwork associated with the treatment of the participant must be coded with a participant ID number. The Research Coordinator is unblinded to the participant's treatment condition assignment, and can be contacted with regard to any participant issues.

Privacy Issues

Risks of confidentiality: All participants will be informed of HIPAA and will be given written information reiterating HIPAA regulations. All people at the clinic and laboratory sites have been trained in HIPAA regulations and procedures. All people interacting with the participant will be reminded of the HIPAA procedures across the study period so that confidentiality is maintained. The clinical and laboratory sites have also been set up to comply with HIPAA regulations. All data forms (hard copy or electronic) will be immediately coded and personal identification information will be removed. All hard copy data forms are stored in a locked cabinet. Only research personnel authorized to access data for the proposed project will have access to the locked cabinet. Informed consent forms will be kept in the PI's office in a locked cabinet separate from the data forms. All data collected at the clinical site will be coded and identifying information removed immediately. Data will be stored in a locked file cabinet at the site. A member of the research personnel will pick up clinical data from the clinical site when the participant has completed the treatment phase and bring it back to the laboratory to be stored with the laboratory data. Only the participating therapists and the RC will have access to this file cabinet in the clinic. Therapists who participate in the study will also sign an agreement not to discuss the treatments or progress of any of the participants in the study with other therapists in the clinic, with other participants in the study, or with research personnel blinded to treatment condition assignment. In addition, the institution in which the clinic is housed has agreed to adhere to all guidelines for protection of human subjects outlined by the NIH.

8.4 Log of Adverse Events

All adverse events (AE) should be documented on the AE form. Serious adverse events (SAE) should be immediately reported to the research personnel. The research personnel will report the SAE directly to the IRB using the following protocol set forth by the Human Research Protection Office at Washington University School of Medicine: a) death within 24 hours, and b) all other internal events within 10 days. Should any adverse events deemed to increase risks to participants be identified, the study will stop immediately and an investigation will be conducted. A report indicating the findings will be produced prior to study resumption.

8.5 Data and Safety Monitoring Plan
In addition to ensuring the safety of research subjects, the PI will be responsible for ensuring data safety and confidentiality. This will be accomplished by keeping all identifying information about research data or health identification data in a locked cabinet that can be accessed only by authorized research personnel. In addition to the daily safety procedures outlined, a committee of 3 health professionals at Washington University School of Medicine who are independent of the study have agreed to act as an internal Data and Safety Monitoring Committee (DSMC) to monitor the progress of the study and the procedures for maintaining the integrity and safety of data collection, processing, and analysis procedures as well as the reporting of adverse events. A professor in the Program in Occupational Therapy and the Department of Neurological Surgery and a professor in the Program in Physical Therapy and the Department of Radiology will provide expertise in the area of the overall integrity of the research process, and in particular, maintenance of the integrity of the data collection and analysis process. A physician and associate professor in the Department of Orthopedic Surgery and the Department of Neurology with a specialty in spine disorders and board certification in pain management will provide expertise with regard to spinal conditions, function as the medical monitor, and guide the committee with regard to actions necessary for dealing with and reporting any adverse events. The PI, and other Co-Investigators as appropriate, will meet with the DSMC two times a year to review progress of the data collection process, evaluate any unanticipated or anticipated side effects of participation in the study, and monitor the integrity and the accuracy of the data generated from the study. The biostatistics team will be responsible for overseeing the presentation of data at the DSMC meetings. In particular, The PI, the biostatistics team and other members of the research team will present recruitment and data issues and analyses at each meeting. Reports will be kept of each meeting and kept on record in the PI's laboratory. The PI and the research personnel will be responsible for implementing any procedures recommended by the DSMC. In addition to data and safety monitoring by the DSMC, adverse events will be reported to the Washington University Human Research Protection Office (HRPO) by the Principal Investigator within 24 hours of the event. Serious adverse events will also be reported to the funding institute. The Washington University HRPO will also monitor the progress of the study, the safety and privacy compliance of the recruitment and data collection procedures, and the proper reporting of adverse events through required yearly progress reports. The yearly progress reports include cumulative reports of any unanticipated or adverse events, yearly renewals of our informed consent document, and approval of all recruitment materials used for the study. If a serious adverse event occurs, the Washington University HRPO will be contacted immediately and the forms accessible through the HRPO website will be accessed to document the circumstances of the event. Changes in the consent form or protocol will be made if indicated based on the nature of the unanticipated or adverse event.

9. Assessments and Outcome Measures

9.1 Outcome Measures Overview
There will be an array of measures to assess outcomes, examine potential treatment effect modifiers, prescribe and progress treatment, and describe the sample. The measures were chosen to capture the important dimensions recommended for the study of people with LBP. All self-report measures will be collected using the automated, web-based system. The Time Table for Administration includes information about the time points of administration. The total administration time for all study measures is ~3 hours. All participants will receive a subset of self-report measures monthly that require ~30 minutes to complete.

9.2 Key Self-Report Measures
Modified Oswestry Disability Questionnaire (MODQ): The primary outcome variable is the 10-item MODQ, a disease-specific measure that provides an index of a participant's perceived LBP-related functional limitation. The scale for the MODQ ranges from 0-100; 100 represents the highest level of limitation. The MODQ is reliable, valid, and sensitive to change.

Numeric Pain Rating Scale (NRS): An 11-point scale (0-10) will be used to measure current pain, as well as average and worst pain over the prior 7 days; larger numbers indicate more intense pain. NRS measurements are reliable, valid, can be treated as ratio scale data, and provide sufficient levels of discrimination to describe pain intensity at varying levels of acuity.

9.3 Adherence to Home Program
During both the initial treatment phase and the booster phase, a standardized self-report measure of daily treatment adherence will be used. Participants will use a Visual Analog Scale (VAS) to indicate the percentage of the treatment they were able to perform as prescribed within a specific time interval (e.g., daily). The scale ranges from 0-100% with higher values indicating higher adherence. This measure will also be used to quantify adherence as part of the monthly, web-based, self-reports.

9.4 Descriptions of Self-Report Measures
Modified Oswestry Disability Questionnaire (MODQ)
Background/Purpose: This questionnaire is designed to give the therapist information as to how LBP has affected a participant's ability to manage in everyday life. It is a 10-item, disease-specific measure that provides an index of the patient's perceived LBP-related functional limitation.
Time points of administration: Laboratory Visit 1, Clinic Visits 1-6, Laboratory Visit 2, follow-up months 1-12, Booster Sessions
Procedure: Participants will answer each of the 10 questions by placing a mark in the one box that best describes his current condition. Since a participant may feel that 2 of the statements describe his condition, he is instructed to mark only the box that most closely describes his current condition.
Scoring: Each item is given a value from 0-5. The total score is the sum of all questions divided by 50, multiplied by 100 to get a percent. 100 represents the highest level of limitation. The minimal clinically important difference (MCID) for the MODQ for people with chronic LBP is 4-6 points.[63]

Numeric Pain Rating Scale (NRS)
Background/Purpose: This questionnaire is designed to give the therapist information on the intensity of a participant's LBP symptoms.
Time points of administration: Laboratory Visit 1, Clinic Visits 1-6, Laboratory Visit 2, follow-up months 1-12, Booster Sessions
Procedure: Participants will rate their LBP symptoms on a numeric scale of 0-10 where 0 represents no symptoms and 10 represents symptoms as bad as can be. They will rate their average symptoms over the prior 7 days, and worst symptoms over the prior 7 days.
Scoring: The score for each item is the rating the participant provides for each symptom category (average and worst). The MCID for the NRS is 2[80,81] points or 30%[80] change from baseline.

Acute Flare-Ups of LBP in Past 6 Months
Background/Purpose: This questionnaire is designed to give the therapist information about the history of a participant's LBP flare-ups in the past 6 months.

Definition: A flare-up is an increase in symptoms of at least 2 points on the NRS above a person's typical low back pain and lasts for at least 2 consecutive days[82]
Time points of administration: Laboratory Visit 1, Laboratory Visit 3, follow-up month 12
Procedure: Participants will fill in information on how many acute flare-ups they have had over the past 6 months, how many days each flare-up averaged in length, and the average pain intensity during the flare-ups.
Scoring: The score for each item will be the number the participant provides for each flare-up category: (1) Number of acute flare-ups over the last 6 months, (2) Average length in days of acute flare-ups, (3) Average intensity of acute flare-ups.

LBP Recurrences
Background/Purpose: This questionnaire is designed to give the therapist information about the history of a participant's LBP recurrences in the past 6 months.
Definition: A recurrence is an increase in LBP symptoms of at least a 2/10 that lasts for at least 24 hours and is preceded and followed by at least 30 days of no symptoms[74]
Time points of administration: Laboratory Visit 1, Laboratory Visit 3, follow-up month 12
Procedure: Participants will fill in how many recurrences of LBP they have had over the past 6 months, how many days each recurrence averaged in length, and the average pain intensity during the recurrences.
Scoring: The score for each item will be the number the participant provides for each recurrence category: (1) Number of recurrences over the last 6 months, (2) Average length in days of recurrences, (3) Average intensity of recurrences.

36-Item Short Form Health Survey (SF-36)
Background/Purpose: This questionnaire is designed to assess information about a participant's mental and physical health, and how well he is able to do his usual activities.
Time points of administration: Laboratory Visit 1, Laboratory Visit 2, Laboratory Visit 3, follow-up month 12
Procedure: Participants will answer each question by marking the answer as indicated. If participants are unsure how to answer a question, they are instructed to choose the best answer they can.
Scoring: Using a scoring application, the SF-36 provides 8 scales: a Physical Functioning, Role-Physical, Bodily Pain, General Health, Vitality, Social Functioning, Role-Emotional, and a Mental Health score. Each score is transformed to a 0-100 scale. The scores also are combined to provide a Physical Component (PCS) and Mental Component (MCS) Summary score. For the PCS and the MCS, norm-based scores are scaled and normalized to have a mean of 50 and a standard deviation of 10 based on the 1998 population norms.[83]

Absenteeism from Usual Activities
Background/Purpose: This questionnaire is designed to assess the number of days a participant has been kept from his usual activities (work, school or housework) because of LBP.[84,85]
Time points of administration: Laboratory Visit 1, Laboratory Visit 2, follow-up months 1-12
Procedure: Participants will answer each question by marking the answer as indicated.
Scoring: Quantified as number of days the participant did not participate in usual activities over the past 4 weeks. Range is from 0-28 days. Higher numbers indicate more days kept from activity/more affected by LBP.

Stanford Presenteeism Scale

Background/Purpose: This questionnaire is designed to assess how a participant's LBP affected his participation in usual activities and the impact of LBP on his ability to do his job over the past 4 weeks.
Time points of administration: Laboratory Visit 1, Laboratory Visit 2, follow-up months 1-12
Procedure: Participants will answer each question by marking the answer as indicated.
Scoring: Presenteeism is indexed in 3 ways. First, a Work Impairment Score (WIS) is calculated as the sum of answers on the questions regarding how LBP has affected job ability over the past 4 weeks. Each of the WIS questions is ranked on a 5-point Likert scale. All scores are 1-5, with questions 2, 5, 6, 8, and 10 reverse scored (5-1). The score ranges from 10-50 with 50 indicating the highest degree of impairment. Second, a Work Output Score (WOS) is quantified using the participant's estimate of the percentage of his usual productivity level during work over the past 4 weeks (1-100%). Third, a Work Absenteeism Score (WAS) is quantified using the participant's response to the number of work hours (1-40+) he missed in the past 4 weeks.[86]

<u>Current Medication Use for LBP</u>
Background/Purpose: This questionnaire is designed to provide general information on the use of medication for a participant's LBP.
Time points of administration: Laboratory Visit 1, Laboratory Visit 2, follow-up months 1-12
Procedure: Participants will answer each question by marking if they are taking non-prescription medication and prescription medication for their LBP. If yes, participants will indicate which medications they are taking and how many pills per day they are taking. For Laboratory Visit 1 and Laboratory Visit 2, the participant will answer regarding medications he is currently taking. At follow-up months 1-12, the participant will answer regarding the past 4 weeks.

<u>Health Professional Care Seeking for LBP</u>
Background/Purpose: This questionnaire is designed to provide general information on the use of additional treatments for a participant's LBP.
Time points of administration: Laboratory Visit 1, Laboratory Visit 2, follow-up months 1-12
Procedure: Participants will answer each question by marking yes or no for a list of other healthcare professionals they are seeing for treatment of their LBP. At Laboratory Visit 2 and follow-up months 1-12, if participants mark yes for a healthcare professional, they are subsequently asked how many times they saw that healthcare professional in the past 4 weeks.

<u>Equipment Use for LBP</u>
Background/Purpose: This questionnaire is designed to provide general information on the use of equipment for a participant's LBP.
Time points of administration: Laboratory Visit 1, Laboratory Visit 2, follow-up months 1-12
Procedure: Participants will answer each question by marking yes or no for a list of equipment they are using to treat their LBP. At Laboratory Visit 1 and Laboratory Visit 2 participants will answer regarding equipment they are currently using. At follow-up months 1-12, participants will answer if they obtained additional equipment in the past 4 weeks.

<u>Satisfaction with Care</u>
Background/Purpose: This questionnaire assesses information about how satisfied a participant feels with the physical therapist and treatment he was provided.
Time points of administration: Laboratory Visit 2
Procedure: Participants will answer each question by marking the answer as indicated. They may only check off one answer per item.

Scoring: Each question is ranked on a 5-point Likert scale. All scores are 1-5, with questions 1, 3, 4, 8, 9, 10 and 13 reverse scored (5-1). The total score is the sum of all of the answers (15-75).

Adherence to Home Program

Background/Purpose: This questionnaire is designed to provide information about how often a participant performs his treatment as it was prescribed. For the MST participants, the adherence is a combination of their prescribed exercises and the amount they applied the principles learned in treatment and performed the activities as prescribed across their day. For the SFE participants, the adherence is a combination of their prescribed strengthening and flexibility exercises.
Time points of administration: Clinic Visits 2-6, follow-up months 1-12, Booster Sessions 2-3
Clinic Visits (Daily Adherence)
Procedure: Participants will use a VAS to indicate for each day the percentage of the treatment they were able to perform as prescribed. At each clinic visit the therapist will ask the participant to provide an estimate of the average percentage of the treatment he was able to perform as prescribed in the interval of time between 2 clinic visits.
Scoring: Average adherence is calculated by averaging the participants' daily adherence. Scores range from 0-100%**.** Higher values indicate higher adherence to treatment.[64]
Follow-up months 1-12 (Monthly Adherence)
Procedure: Participants will use a VAS to indicate an average percent adherence to performance of the treatment as prescribed over the past month.
Scoring: Value (percentage of treatment performed) provided by the participant on each monthly survey. Scores range from 0-100%. Higher values indicate higher adherence to treatment.[64]

Demographics and LBP and Medical History

Background/Purpose: This questionnaire is designed to provide general information about the participant's demographics, LBP and medical history.
Time points of administration: Laboratory Visit 1
Procedure: Participants will provide information about each of the following items: age, handedness, gender, race, ethnicity, occupation, employment situation, marital status, education, LBP history, medical history.

Fear-Avoidance Beliefs Questionnaire (FABQ)

Background/Purpose: This questionnaire is designed to assess a participant's fear of pain and beliefs about how work and physical activity affect his LBP.
Time points of administration: Laboratory Visit 1, Laboratory Visit 2, Laboratory Visit 3
Procedure: Participants will answer each question by marking the answer as indicated.
Scoring: Each question is ranked on a 7-point Likert scale (0-6). Higher scores indicate higher fear-avoidance**.** Two subscale scores are calculated. The physical activity subscale score (FABQ-PA) is the sum of questions 2, 3, 4, and 5 and ranges from 0-24. The work subscale score (FABQ-W) is the sum of items 6, 7, 9, 10, 11, 12, and 15 and ranges from 0-42**.**

Treatment Preference Assessment Measure (TPA)

Background/Purpose: This questionnaire is designed to provide information about a participant's treatment preferences and the participant's perceptions of four attributes of each treatment: effectiveness, acceptability, suitability/appropriateness, and convenience. The questionnaire is modified from a preference questionnaire designed by Sidani et al[87] to reflect the treatments provided in the trial.
Time points of administration: Laboratory Visit 1, before randomization

Procedure: The two treatment descriptions will be given in a random order to each participant. First, the RA will inform the participant that she is interested in learning about his perception of each treatment. Second, the RA will read the description of the first treatment option. The RA will read the description slowly, clearly, and in an unbiased manner, in order to facilitate understanding. Third, the RA will ask the participant to rate the attributes of the treatment option just described. Fourth, the RA will repeat the three steps as they relate to the second treatment option. Fifth, the RA will ask the participant if he has a preference for either of the two treatment options, and if so, which one.
Scoring: The four treatment attributes (effectiveness, acceptability, suitability/appropriateness, and convenience) will be rated on a 5-point Likert scale (0-4)**.** A total scale score for each treatment will be computed as the mean of the four attribute ratings. Whether the participant prefers one of the two treatments will be recorded as yes/no. If the participant has a preference, then his preferred treatment will be recorded as MST or SFE.

9.4a Time Table for Administration

Measures	Laboratory Visit 1 (Baseline)	Clinic Visits 1-6	Laboratory Visit 2 (Post-Treatment)	Follow-up months 1-12 (Web-Based)	Laboratory Visit 3 (Follow-Up Month 6)	Booster Session (At Month 6)
MODQ	X	X (paper)	X	X		X (paper)
NRS (average & worst in prior 7 days)	X	X (paper)	X	X		X (paper)
Acute Flare-Ups of LBP	X			X (12 month only)	X	
LBP Recurrences	X			X (12 month only)	X	
SF-36	X		X	X (12 month only)	X	
Absenteeism from Usual Activities	X		X	X		
Stanford Presenteeism Scale	X		X	X		
Use of LBP-related medications & Additional treatment for LBP	X		X	X		
Satisfaction with care			X			
Adherence to Home Program		X (paper)		X (electronic)		X (paper)
Demographics/LBP & medical history	X					
FABQ	X		X		X	
TPA	X					
Contact Information	X					
Email Confirmation			X		X	
Clinical examination to classify LBP	X		X		X	

Clinic visit documentation		X				X

10. Data Management

10.1 Data Management Overview

The biostatistics team will oversee all activities. The PI and the Research Biostatistician will supervise data management. The research biostatistician will prepare web-based forms for data entry and editing, oversee data entry, write programs for data validation, manage the security and privacy of the electronic forms and data sets, prepare data sets for analysis, create progress reports, and provide for regular, secure back up of data. We will use a secure database created using Research Electronic Data Capture (REDCap) tools hosted by Washington University Biostatistics.

10.2 Sample Size Estimate

A power analysis[88] was conducted to determine adequate sample size for detecting a minimally important clinical difference of 6 on the MODQ.[89] Because power procedures for HLM models are not yet well developed, we used a multiple regression approach,[90] a reasonable alternative given both HLM and multiple regression are similar in form and intent. Furthermore, we took the conservative approach of powering the study for detection of a difference within a given time point. A study adequately powered to detect within-time differences will then have equal or greater power to detect differences over time to the extent that measures over time are correlated. This is a reasonable assumption, and in fact, analyses of data from our prior trial indicate correlations among the MODQ scores collected at different times averaged .49. The calculations based on a single time period thus provide a conservative estimate and “worst case” approach to determining sample size. [90] The power analysis assumed 1) the basic 4 group (Treatment x Booster) design (3 df), 2) inclusion of 3 additional predictors (e.g., age, adherence, classification; 3 df), 3) an interaction component reflecting how the effect of treatment could depend on other predictors (9 df), 4) attrition of 20%, 5) a .05 level of significance (two-tailed), and 6) minimum power of .80. The chosen power value is the common standard used in most power analyses. Using the target effect of 6 on the MODQ and variability estimates from the prior trial, the power analysis indicated that 154 participants would need to be enrolled. Note that although examining moderation of treatment impact by LBP classification is not a specific aim, we will be able to explore such moderation by including dummy codes representing classification as additional predictors in the model. Note also that there are a large number of potential moderators that could be included in the statistical model, but we made provisions for including 3 additional predictors in any given analysis because of the likely intercorrelations that will exist among candidate predictors. Preliminary analyses will identify relative independent predictors or create composite predictors from highly correlated measures.

10.3 Analyses

Primary Outcome Analyses

For MODQ, our primary outcome measure, mixed random effect repeated measures analyses will be made separately on each phase (treatment and follow-up) with participant as a random effect and a first order autoregressive covariance structure to account for correlation between time points. Treatment, time, and time by treatment interaction will be included as fixed effects. The baseline MODQ score will be a covariate in both models to control for baseline participant differences. If booster treatments after follow-up month 6 do not affect subsequent MODQ scores, treatment estimates from data after month 6 will be created from combined booster and no booster groups within treatment. These analyses will be intent-to-treat and use all data

available. Mean estimates for single time points will be model based (unless otherwise noted). No missing data imputation will be done.

Secondary Outcome Analyses
For average and worst NRS pain scores, SF-36 PCS and MCS scores, Stanford Presenteeism Scale WIS, and the FABQ-PA and FABQ-W Physical and Work scores, treatment and follow-up phase analyses will use mixed random effect repeated measures analysis (SAS mixed procedure) with baseline outcome values included as covariates and with participant within treatment as a random effect. Treatment contrasts will be reported with 95% confidence intervals without adjustment for experiment-wise error.

Variables will be transformed to stabilize variance. Contrasts of SFE – MST for transformed variables will be back-transformed to their original scale and confidence intervals simulated from Least Square means then back-transformed.

Variables that contain many 0 records will be transformed to a dichotomous scale and analyzed using a mixed generalized linear model (SAS glimmix procedure) with a logit link and binomial distribution. Contrasts for these variables will be reported as odds ratios.

Within treatment contrasts for the effect of booster on change between pre-booster (follow up months 1 through 6) and post-booster (follow up months 7 through 12) will be reported on the transformed scale. Back-transformed least square means will be reported on the original scale of each variable.

11. References

1. Frymoyer JW. Back pain and sciatica. *N Engl J Med.* 1988;318(5):291-300.
2. Papageorgiou AC, Croft PR, Thomas E, Ferry S, Jayson MI, Silman AJ. Influence of previous pain experience on the episode incidence of low back pain: results from the South Manchester Back Pain Study. *Pain.* 1996;66(2-3):181-185.
3. Pizzo PA, Clark NM, Carter-Pokras O, et al. *Relieving Pain in America: A Blueprint for Transforming Prevention, Care, Education, and Research.* Washington, DC June 2011 2011.
4. Knauer SR, Freburger JK, Carey TS. Chronic low back pain among older adults: a population-based perspective. *J Aging Health.* 2010;22(8):1213-1234.
5. Sinnott P, Wagner TH. Low back pain in VA users. *Arch Intern Med.* 2009;169(14):1338-1339.
6. Croft PR, Macfarlane GJ, Papageorgiou AC, Thomas E, Silman AJ. Outcome of low back pain in general practice: a prospective study. *BMJ.* 1998;316(7141):1356-1359.
7. Wahlgren DR, Atkinson JH, Epping-Jordan JE, et al. One-year follow-up of first onset low back pain. *Pain.* 1997;73(2):213-221.
8. Hestbaek L, Leboeuf-Yde C, Kyvik KO, Manniche C. The course of low back pain from adolescence to adulthood: eight-year follow-up of 9600 twins. *Spine (Phila Pa 1976).* 2006;31(4):468-472.
9. Hestbaek L, Leboeuf-Yde C, Manniche C. Low back pain: what is the long-term course? A review of studies of general patient populations. *Eur Spine J.* 2003;12(2):149-165.
10. Hestbaek L, Leboeuf-Yde C, Engberg M, Lauritzen T, Bruun NH, Manniche C. The course of low back pain in a general population. Results from a 5-year prospective study. *J Manipulative Physiol Ther.* 2003;26(4):213-219.
11. Von Korff M. Studying the natural history of back pain. *Spine (Phila Pa 1976).* 1994;19(18 Suppl):2041S-2046S.

12. Martin BI, Deyo RA, Mirza SK, et al. Expenditures and health status among adults with back and neck problems. *J Am Med Assoc.* 2008;299(6):656-664.
13. van Tulder M, Malmivaara A, Esmail R, Koes B. Exercise therapy for low back pain: a systematic review within the framework of the Cochrane collaboration back review group. *Spine (Phila Pa 1976).* 2000;25(21):2784-2796.
14. Hayden JA, van Tulder MW, Malmivaara A, Koes BW. Meta Analysis: Exercise therapy for treatment of non-specific low back pain. *Ann Intern Med.* 2005;142(9):765-775.
15. Van Middelkoop M, Rubinstein SM, Kuijpers T, et al. A systematic review on the effectiveness of physical and rehabilitation interventions for chronic non-specific low back pain. *Eur Spine J.* 2011;20(1):19-39.
16. Van Middelkoop M, Rubinstein SM, Verhagen AP, Ostelo RW, Koes BW, van Tulder MW. Exercise therapy for chronic nonspecific low-back pain. *Best Practice and Research in Clinical Rheumatology.* 2010;24:193-204.
17. Hayden JA, van Tulder MW, Malmivaara A, Koes BW. Exercise therapy for treatment of non-specific low back pain. *Cochrane Database Syst Rev.* 2005;3:CD000335.
18. Liddle SD, Baxter GD, Gracey JH. Exercise and chronic low back pain: what works? *Pain.* 2004;107(1-2):176-190.
19. Hayden JA, van Tulder M, Tomlinson G. Systematic review: Strategies for using exercise therapy to improve outcomes in chronic low back pain. *Ann Intern Med.* 2005;142(9):776-785.
20. Macedo LG, Maher CG, Latimer J, McAuley JH. Motor control exercise for persistent, nonspecific low back pain: a systematic review. *Phys Ther.* 2009;89(1):9-25.
21. Rackwitz B, de Bie R, Limm H, von Garnier K, Ewert T, Stucki G. Segmental stabilizing exercises and low back pain. What is the evidence? A systematic review of randomized controlled trials. *Clin Rehabil.* 2006;20(7):553-567.
22. Chou R, Huffman LH. Nonpharmacologic therapies for acute and chronic low back pain: a review of the evidence for an American Pain Society/American College of Physicians clinical practice guideline. *Ann Intern Med.* 2007;147(7):492-504.
23. Hayden JA, van Tulder MW, Tomlinson G. Systematic review: strategies for using exercise therapy to improve outcomes in chronic low back pain. *Ann Intern Med.* 2005;142(9):776-785.
24. Henchoz Y, Kai-Lik SA. Exercise and nonspecific low back pain: a literature review. *Joint Bone Spine.* 2008;75(5):533-539.
25. Ferreira PH, Ferreira ML, Maher CG, Herbert RD, Refshauge K. Specific stabilisation exercise for spinal and pelvic pain: a systematic review. *Aust J Physiother.* 2006;52(2):79-88.
26. Croft P, Papageorgiou A, McNally R. Low back pain. In: *Health Care Needs Assessment.* New York, NY: Radcliffe Medical Press Oxford; 1997:129-182.
27. Slade SC, Keating JL. Trunk-strengthening exercises for chronic low back pain: a systematic review. *J Manipulative Physiol Ther.* 2006;29(2):163-173.
28. Liddle SD, David BG, Gracey JH. Physiotherapists' use of advice and exercise for the management of chronic low back pain: A national survey. *Man Ther.* 2008;14(2):189-196.
29. Waddell G. *The Back Pain Revolution.* Edinburgh: Churchill Livingstone; 1998.
30. Jordan JL, Holden MA, Mason EE, Foster NE. Interventions to improve adherence to exercise for chronic musculoskeletal pain in adults. *Cochrane Database Systematic Review.* 2010(1):CD005956.
31. Lechner DE. Work hardening and work conditioning interventions: do they affect disability? *Phys Ther.* 1994;74(5):471-493.

32. Poiraudeau S, Rannou F, Revel M. Functional restoration programs for low back pain: a systematic review. *Annales de Readaptation et de Medecine Physique.* 2007;50(6):425-429.
33. Schonstein E, D K, J K, B K, RD H. Physical conditioning programs for workers with back and neck pain: A cochrane systematic review. *Spine (Phila Pa 1976).* 2003;19(28):E391-E395.
34. Guzman J, Esmail R, Karjalainen K, Malmivaara A, Irvin E, Bombardier C. Multidisciplinary rehabilitation for chronic low back pain: systematic review. *BMJ (Clinical research ed).* 2001;322(7301):1511-1516.
35. Schaafsma F, Schonstein E, Ojajarvi A, Verbeek J. Physical conditioning programs for improving work outcomes among workers with back pain. *Scand J Work Environ Health.* 2011;37(1):1-5.
36. Schonstein E, Kenny DT, Keating J, Koes BW. Work conditioning, work hardening and functional restoration for workers with back and neck pain. *Cochrane Database Syst Rev.* 2010;CD001822(1).
37. Teasell RW, Harth M. Functional restoration. Returning patients with chronic low back pain to work--revolution or fad? *Spine (Phila Pa 1976).* 1996;21(7):844-847.
38. Guzman J, Esmail R, Karjalainen K, Malmivaara A, Irvin E, Bombardier C. Multidisciplinary bio-psycho-social rehabilitation for chronic low back pain. *Cochrane Database Syst Rev.* 2002(1):CD000963.
39. Conner JM, Culberson A, Packowski C, Chiba AA, Tuszynski MH. Lesions of the Basal forebrain cholinergic system impair task acquisition and abolish cortical plasticity associated with motor skill learning. *Neuron.* 2003;38(5):819-829.
40. Graybiel AM. The basal ganglia: learning new tricks and loving it. *Curr Opin Neurobiol.* 2005;15(6):638-644.
41. De Zeeuw CI, Yeo CH. Time and tide in cerebellar memory formation. *Curr Opin Neurobiol.* 2005;15(6):667-674.
42. Kleim JA, Swain RA, Armstrong KA, Napper RM, Jones TA, Greenough WT. Selective synaptic plasticity within the cerebellar cortex following complex motor skill learning. *Neurobiol Learn Mem.* 1998;69(3):274-289.
43. Karni A, Meyer G, Jezzard P, Adams MM, Turner R, Ungerleider LG. Functional MRI evidence for adult motor cortex plasticity during motor skill learning. *Nature.* 1995;377(6545):155-158.
44. Adkins DL, Boychuk J, Remple MS, Kleim JA. Motor training induces experience-specific patterns of plasticity across motor cortex and spinal cord. *J Appl Physiol.* 2006;101(6):1776-1782.
45. Nudo RJ. Plasticity. *NeuroRx.* 2006;3(4):420-427.
46. Classen J, Knorr U, Werhahn KJ, et al. Multimodal output mapping of human central motor representation on different spatial scales. *J Physiol.* 1998;512(Pt 1):163-179.
47. Hund-Georgiadis M, von Cramon DY. Motor-learning-related changes in piano players and non-musicians revealed by functional magnetic-resonance signals. *Exp Brain Res.* 1999;125(4):417-425.
48. Pascual-Leone A, Wassermann EM, Sadato N, Hallett M. The role of reading activity on the modulation of motor cortical outputs to the reading hand in Braille readers. *Ann Neurol.* 1995;38(6):910-915.
49. Kleim JA, Cooper NR, VandenBerg PM. Exercise induces angiogenesis but does not alter movement representations within rat motor cortex. *Brain Res.* 2002;934(1):1-6.
50. Remple MS, Bruneau RM, VandenBerg PM, Goertzen C, Kleim JA. Sensitivity of cortical movement representations to motor experience: evidence that skill learning but not strength training induces cortical reorganization. *Behav Brain Res.* 2001;123(2):133-141.

51. Sahrmann SA. Movement impairment syndromes of the lumbar spine. In: *Diagnosis and Treatment of Movement Impairment Syndromes.* 1st ed. St. Louis, MO: Mosby; 2002:51-118.
52. Hoffman SL, Johnson MB, Zou D, Van Dillen LR. Sex differences in lumbopelvic movement patterns during hip medial rotation in people with chronic low back pain. *Arch Phys Med Rehabil.* 2011;92(7):1053-1059.
53. Gombatto SP, Collins DR, Sahrmann SA, Engsberg JR, Van Dillen LR. Gender differences in pattern of hip and lumbopelvic rotation in people with low back pain. *Clinical Biomechanics.* 2006;21(3):263-271.
54. Scholtes SA, Gombatto SP, Van Dillen LR. Gender differences in timing of lumbopelvic movement during the clinical test of active knee flexion. *J Orthop Sports Phys Ther.* 2008;38(1):A69.
55. Van Dillen LR, Sahrmann SA, Engsberg JR, Lenke LG. Trunk rotation-related impairments in people with low back pain who participate in rotational sports activities. Paper presented at: 5th Interdisciplinary World Congress on Low Back Pain & Pelvic Pain; November 10-13, 2004; Melbourne, Australia.
56. Van Dillen LR, Sahrmann SA, Caldwell CA, McDonnell MK, Bloom NJ, Norton BJ. Trunk rotation-related impairments in people with low back pain who participated in two different types of leisure activities: a secondary analysis. *J Orthop Sports Phys Ther.* 2006;36(2):58-71.
57. Scholtes SA, Gombatto SP, Van Dillen LR. Differences in lumbopelvic motion between people with and people without low back pain during two lower limb movement tests. *Clinical Biomechanics.* 2009;24(1):7-12.
58. Norton BJ, Sahrmann SA, Van Dillen LR. Differences in measurements of lumbar curvature related to gender and low back pain. *J Orthop Sports Phys Ther.* 2004;34(9):524-534.
59. Van Dillen LR, Gombatto SP, Collins DR, Engsberg JR, Sahrmann SA. Symmetry of timing of hip and lumbopelvic rotation motion in 2 different subgroups of people with low back pain. *Arch Phys Med Rehabil.* 2007;88(3):351-360.
60. Hoffman SL, Harris-Hayes M, Zou D, Baxter KR, Tateuchi H, Van Dillen LR. Spinal and hip flexion end-range of motion with forward bending in two low back pain subgroups based on the Movement System Impairment model. *J Orthop Sports Phys Ther.* 2010;40(1):A76.
61. Hoffman SL, Harris-Hayes M, Zou D, Baxter KR, Tateuchi H, Van Dillen LR. Pattern of movement during return from forward bending in two low back pain subgroups based on the Movement System Impairment model. *J Orthop Sports Phys Ther.* 2010;40(1):A77.
62. Adams M, Bogduk N, Burton K, Dolan P. Functional pathology. In: *The Biomechanics of Back Pain.* New York, NY: Churchill Livingstone; 2002:168-172.
63. Fritz JM, Irrgang JJ. A comparison of a modified Oswestry Low Back Pain Disability Questionnaire and the Quebec Back Pain Disability Scale. *Phys Ther.* 2001;81(2):776-788.
64. Van Dillen LR, Norton BJ, Sahrmann SA, et al. Effects of classification specific treatment versus non specific treatment on low back pain related functional disability in people with chronic low back pain over a one year period. Paper presented at: XIX Biennial ISEK Congress; July 19-21, 2012; Brisbane, Australia.
65. Jette DU, Jette AM. Physical therapy and health outcomes in patients with spinal impairments. *Phys Ther.* 1996;76(9):930-941.
66. Jette AM, Smith K, Haley SM, Davis KD. Physical therapy episodes of care for patients with low back pain. *Phys Ther.* 1994;74(2):101-110.
67. Jette AM, Delitto A. Physical therapy treatment choices for musculoskeletal impairments. *Phys Ther.* 1997;77(2):145-154.

68. Organization WH. *Adherence to Long-Term Therapies: Evidence for action.* Switzerland 2003 2003.
69. Pisters MF, Veenhof C, van Meeteren NL, et al. Long-term effectiveness of exercise therapy in patients with osteoarthritis of the hip or knee: a systematic review. *Arthritis Rheum.* 2007;57(7):1245-1253.
70. Pisters MF, Veenhof C, de Bakker DH, Schellevis FG, Dekker J. Behavioural graded activity results in better exercise adherence and more physical activity than usual care in people with osteoarthritis: a cluster-randomised trial. *J Physiother.* 2010;56(1):41-47.
71. Friedrich M, Gittler G, Arendasy M, Friedrich KM. Long-term effect of a combined exercise and motivational program on the level of disability of patients with chronic low back pain. *Spine (Phila Pa 1976).* 2005;30(9):995-1000.
72. Friedrich M, Gittler G, Halberstadt Y, Cermak T, Heiller I. Combined exercise and motivation program: effect on the compliance and level of disability of patients with chronic low back pain: a randomized controlled trial. *Arch Phys Med Rehabil.* 1998;79(5):475-487.
73. Harris-Hayes M, Holtzman GW, Early J, Van Dillen LR. Development and preliminary reliability testing of an assessment of patient independence in performing a treatment program: standardized scenarios. *J Rehabil Med.* 2010;42(3):221-227.
74. Stanton TR, Latimer J, Maher CG, Hancock MJ. A modified Delphi approach to standardize low back pain recurrence terminology. *Eur Spine J.* 2011;20(5):744-752.
75. Nordin M, Weiser S, van Doorn JW, Hiebert R. Nonspecific low back pain. In: Rom WN, ed. *Environmental and Occupational Medicine.* 3rd ed. Philadelphia, PA: Lippincott-Raven Publishers; 1998:947-956.
76. Harris-Hayes M, Van Dillen LR. The inter-tester reliability of physical therapists classifying low back pain problems based on the movement system impairment classification system. *Physical Medicine and Rehabilitation.* 2009;1(2):117-126.
77. Van Dillen LR, Sahrmann SA, Norton BJ, Caldwell CA, McDonnell MK, Bloom NJ. Movement system impairment-based categories for low back pain: stage 1 validation. *J Orthop Sports Phys Ther.* 2003;33(3):126-142.
78. Trudelle-Jackson E, Sarvaiya-Shah SA, Wang SS. Interrater reliability of a movement impairment-based classification system for lumbar spine syndromes in patients with chronic low back pain. *J Orthop Sports Phys Ther.* 2008;38(6):371-376.
79. Van Dillen LR, Maluf KS, Sahrmann SA. Further examination of modifying patient-preferred movement and alignment strategies in patients with low back pain during symptomatic tests. *Man Ther.* 2009;14(1):52-60.
80. Farrar JT, Young JP, Jr., LaMoreaux L, Werth JL, Poole RM. Clinical importance of changes in chronic pain intensity measured on an 11-point numerical pain rating scale. *Pain.* 2001;94(2):149-158.
81. Childs JD, Piva SR, Fritz JM. Responsiveness of the numeric pain rating scale in patients with low back pain. *Spine (Phila Pa 1976).* 2005;30(11):1331-1334.
82. Von Korff M. Studying the natural history of back pain. *Spine (Phila Pa 1976).* 1994;19(18 Suppl):2041S-2046S.
83. Ware JE, Jr. SF-36 health survey update. *Spine (Phila Pa 1976).* 2000;25(24):3130-3139.
84. Von Korff M, Ormel J, Keefe FJ, Dworkin SF. Grading the severity of chronic pain. *Pain.* 1992;50(2):133-149.
85. Patrick DL, Deyo RA, Atlas SJ, Singer DE, Chapin A, Keller RB. Assessing health-related quality of life in patients with sciatica. *Spine (Phila Pa 1976).* 1995;20(17):1899-1908.
86. Turpin RS, Ozminkowski RJ, Sharda CE, et al. Reliability and validity of the Stanford Presenteeism Scale. *J Occup Environ Med.* 2004;46(11):1123-1133.

87. Sidani S, Epstein DR, Bootzin RR, Moritz P, Miranda J. Assessment of preferences for treatment: validation of a measure. *Res Nurs Health.* 2009;32(4):419-431.
88. Cohen J. A power primer. *Psychol Bull.* 1992;112(1):155-159.
89. Fritz JM, Irrgang JJ. A comparison of a modified Oswestry Low Back Pain Disability Questionnaire and the Quebec Back Pain Disability Scale. *Phys Ther.* 2001;81(2):776-788.
90. Cohen J, Cohen P. *Applied Multiple Regression/Correlation Analysis for the Behavioral Sciences.* 2nd ed. Mahwah, NJ: Lawrence Erlbaum Associates; 1983.

Summary of Changes to Statistical Analysis Plan

The original protocol called for analysis with hierarchical linear modeling to model repeated measures without requiring time between samples to be constant. However, times between samples were sufficiently consistent to allow use of mixed random effects repeated measures models. The original protocol intended analysis of moderators, but moderators were not included due to strong treatment effects seen in the MODQ scores. Economic assessments were cancelled due to budget reductions.

Number and length of acute flare-ups of LBP in the past 6 months were transformed with natural log (x+1). The Stanford Presenteeism Scale WOS was transformed with natural log (101 – WOS). Adherence to the home program was transformed with a logit function, natural log [x/(100-x)].

Current medication use for LBP, absenteeism from usual activities, the Stanford Presenteeism Scale WAS, health professional care seeking for LBP, and equipment use for LBP contained many 0 records which required transformation to a dichotomous scale and analysis using a mixed generalized linear model (SAS glimmix procedure) with a logit link and binomial distribution. Current medication use for LBP had a first order autoregressive covariance structure, but the others had compound symmetry.

Summary of Changes to the Protocol

Major protocol changes are included.

November, 2013 People with fibromyalgia, Marfan syndrome, and Graves' disease are to be excluded.

January, 2014 Clinic Visit 1 can be scheduled within two weeks of Laboratory Visit 1. Laboratory Visit 2 can be scheduled within two weeks of Clinic Visit 6.

June, 2014 Laboratory Visit 3 can be scheduled within the 2 week period surrounding 6 months following Laboratory Visit 2.

September, 2014 Individuals who do not qualify at Laboratory Visit 1 because MODQ is <20 can be rescreened if LBP status has worsened.

April, 2015 The exploratory economic analyses we proposed to examine direct (medication and health services use and medical equipment) and indirect (absenteeism and presenteeism) health costs are not to be conducted. The decision was based on large budget cuts that limited our ability to financially support the personnel to conduct the analyses.

May, 2015 If a participant is in an acute flare-up when he or she comes in for a laboratory visit, the research physical therapist will determine if it is an actual acute flare-up. If it is determined to

be an actual acute flare-up, the laboratory visit will not be completed and the participant will be rescheduled.

July, 2015 Attendance scripts for Clinic Visit 1 and Clinic Visits 2-6 are to be implemented. These scripts will guide the clinicians to discuss with the participant the importance of scheduling visits and adhering to the schedule.

October, 2015 People with a history of disc herniation are excluded only if they have a current symptomatic disc herniation (current symptoms below the knee).

January, 2017 Preliminary analyses of the recurrence data indicated that only 40 of 77 individuals who reported having recurrences of LBP in the past 6 months had valid data. The data for 37 of 77 individuals who reported having recurrences of LBP did not meet the definition for recurrences (increase in LBP symptoms of at least a 2/10 that lasts for at least 24 hours and is preceded and followed by at least 30 days of no symptoms). Given the questionable validity of the responses, the recurrence data will not be reported.
