# Supplementary material for: Effect of Motor Skill Training in Functional Activities vs Strength and Flexibility Exercise on Function in People With Chronic Low Back Pain: A Randomized Clinical Trial
Source: JAMA Neurol. 2020 Dec 28;78(4):1–11. doi: 10.1001/jamaneurol.2020.4821 (PMC7770617; doi:10.1001/jamaneurol.2020.4821)
Supplement: Supplement 2. — eTable. Primary and Secondary Outcome Least square (LS) Mean Differences and Odds Ratios Pre-Booster (Months 1-6) and Post-Booster (Months 7-12) for People in the Booster Treatment Condition and People in the No Booster Treatment Condition eMethods. Detailed Descriptions of Motor Skill Training and Strength and Flexibility Exercise Treatment Conditions eReferences. [file jamaneurol-e204821-s002.pdf]

# Supplemental Online Content

van Dillen LR, Lanier VM, Steger-May K, et al. Effect of motor skill training in functional activities vs strength and flexibility exercise on function in people with chronic low back pain: a randomized clinical trial. *JAMA Neurol*. Published online December 28, 2020. doi:10.1001/jamaneurol.2020.4821

**eTable.** Primary and Secondary Outcome Least square (LS) Mean Differences and Odds Ratios Pre-Booster (Months 1-6) and Post-Booster (Months 7-12) for People in the Booster Treatment Condition and People in the No Booster Treatment Condition
**eMethods.** Detailed Descriptions of Motor Skill Training and Strength and Flexibility Exercise Treatment Conditions
**eReferences.**

This supplemental material has been provided by the authors to give readers additional information about their work.

© 2020 van Dillen LR et al. *JAMA Neurology*.

eTable. **Primary and Secondary Outcome Least square (LS) Mean Differences and Odds Ratios Pre-Booster (Months 1-6) and Post-Booster (Months 7-12) for People in the Booster Treatment Condition and People in the No Booster Treatment Condition**

| | | | Motor Skill Training | | | | Strength & Flexibility Exercise | | |
|---|---|---|---|---|---|---|---|---|---|
| | | | LS Means (95% CI)[a] | | Booster Change Difference (95% CI)[c] | | LS Means (95% CI)[a] | | Booster Change Difference (95% CI)[c] |
| Variable | | Time[b] | Booster | No Booster | | | Booster | No Booster | |
| modified Oswestry Disability Questionnaire[e] | | Pre-Booster | 11.6 (8.9, 14.2) | 11.3 (8.5, 14.0) | | | 20.4 (17.6, 23.3) | 16.4 (13.7, 19.1) | |
| | | Post-Booster | 11.0 (8.3, 13.6) | 11.4 (8.7, 14.2) | -0.7 (-5.0, 3.5) | | 19.6 (16.7, 22.4) | 14.6 (11.8, 17.3) | 0.9 (-3.5, 5.3) |
| Numeric Pain Rating Scale[f] | Average | Pre-Booster | 1.8 (1.4, 2.2) | 2.3 (1.9, 2.7) | | | 2.7 (2.3, 3.2) | 2.5 (2.1, 2.9) | |
| | | Post-Booster | 1.6 (1.2, 2.0) | 2.4 (2.0, 2.8) | -0.3 (-1.0, 0.4) | | 2.7 (2.3, 3.1) | 2.2 (1.8, 2.6) | 0.2 (-0.5, 0.9) |
| | Worst | Pre-Booster | 3.0 (2.4, 3.5) | 3.5 (3.0, 4.0) | | | 3.9 (3.3, 4.4) | 4.0 (3.3, 4.4) | |
| | | Post-Booster | 2.6 (2.1, 3.1) | 3.5 (3.0, 4.0) | -0.4 (-1.3, 0.5) | | 3.5 (3.0, 4.0) | 3.8 (3.3, 4.3) | 0.2 (-0.7, 1.1) |
| Acute Flare-Ups of LBP in Past 6 Months[g] | Number | Pre-Booster | 1.1 (0.6, 1.7) | 1.3 (0.7, 2.0) | | | 2.2 (1.4, 3.2) | 1.7 (1.0, 2.6) | |
| | | Post-Booster | 0.6 (0.3, 1.1) | 1.0 (0.6, 1.7) | -0.11 (-0.55, 0.32)[d] | | 1.1 (0.6, 1.8) | 1.1 (0.6, 1.8) | -0.15 (-0.60, 0.31)[d] |
| | Length | Pre-Booster | 0.7 (0.5, 1.0) | 0.8 (0.5, 1.1) | | | 1.2 (0.9, 1.5) | 1.0 (0.7, 1.3) | |
| | | Post-Booster | 0.5 (0.3,0.8) | 0.9 (0.6, 1.2) | -0.29 (-0.75, 0.17)[d] | | 0.9 (0.6, 1.2) | 0.8 (0.5, 1.1) | -0.14 (-0.62, 0.33)[d] |
| | Intensity | Pre-Booster | 3.0 (1.9, 4.0) | 3.3 (2.2, 4.4) | | | 4.0 (2.9, 5.2) | 3.7 (2.6, 4.8) | |
| | | Post-Booster | 2.2 (1.2, 3.2) | 3.1 (2.0, 4.2) | -0.6 (-2.2, 1.0) | | 3.3 (2.2, 4.4) | 3.4 (2.3, 4.4) | -0.4 (-2.1, 1.3) |
| SF-36 Component Summary Scores[h] | Physical | Pre-Booster | 48.7 (46.2, 51.1) | 52.0 (49.7, 54.3) | | | 50.2 (47.8, 52.7) | 46.9 (44.4, 49.4) | |
| | | Post-Booster | 48.7 (46.3, 51.1) | 52.5 (50.2, 54.8) | 0.5 ( -2.5, 3.4) | | 49.9 (47.4, 52.3) | 47.3 (44.8, 49.7) | 0.7 (-2.3, 3.8) |
| | Mental | Pre-Booster | 51.0 (47.7, 54.4) | 49.5 (46.3, 52.7) | | | 49.3 (45.9, 52.7) | 51.5 (48.1, 54.9) | |
| | | Post-Booster | 50.3 (47.0, 53.6) | 51.3 (48.1, 54.5) | 2.6 (-2.5, 7.6) | | 50.5 (47.2, 53.7) | 48.5 (45.1, 51.8) | -4.2 (-9.4, 1.0) |
| Stanford Presenteeism Scale[i] | Work Impairment Score | Pre-Booster | 15.5 (14.1, 16.8) | 16.2 (14.9, 17.5) | | | 18.3 (17.0, 19.6) | 17.7 (16.4, 19.1) | |
| | | Post-Booster | 15.6 (14.3, 17.0) | 16.0 (14.6, 17.3) | -0.4 (-2.6, 1.8) | | 17.6 (16.2, 18.9) | 16.8 (15.4, 18.2) | -0.3 (-2.5, 2.0) |
| | Work Output Score | Pre-Booster | 99 (98, 99) | 98 (96, 99) | | | 97 (96, 98) | 98 (97, 99) | |
| | | Post-Booster | 99 (98, 99) | 98 (97, 99) | 0.11 (-0.42, 0.64)[d] | | 98 (97. 99) | 99 (98, 99) | -0.05 (-0.59, 0.49)[d] |
| Adherence to Home Program[j] | | Pre-Booster | 79 (70, 86) | 73 (62, 81) | | | 41 (30, 54) | 62 (50, 72) | |
| | | Post-Booster | 84 (76, 89) | 72 (61, 81) | 0.31 (-0.39, 1.02)[d] | | 46 (34, 59) | 45 (34, 57) | 0.88 (0.16, 1.60)[d] |

© 2020 van Dillen LR et al. *JAMA Neurology*.

| | | | | | | | | | |
|---|---|---|---|---|---|---|---|---|---|
| | | | Percent (95% CI) | | Booster Change Odds Ratio (95% CI) | | Percent (95% CI) | | Booster Change Odds Ratio (95% CI) |
| Absenteeism from Usual Activities[k] — no. (%) | | Pre-Booster | 13 ( 8, 22) | 21 (13, 31) | | | 21 (13, 31) | 19 (12, 29) | |
| | | Post-Booster | 11 ( 6, 20) | 16 ( 9, 25) | 1.2 (0.4, 3.6) | | 18 (11, 29) | 11 ( 6, 20) | 1.7 (0.5, 5.0) |
| Stanford Presenteeism Scale[k] — no. (%) | Work Absenteeism | Pre-Booster | 23 (11, 43) | 22 (10, 42) | | | 30 (15, 51) | 28 (14, 49) | |
| | | Post-Booster | 13 ( 4, 33) | 19 ( 8, 38) | 0.6 (0.1, 3.3) | | 21 ( 9, 42) | 15 ( 5, 34) | 1.4 (0.3, 6.8) |
| Health Professional Care Seeking for LBP[k] — no. (%) | | Pre-Booster | 47 (30, 64) | 38 (22, 57) | | | 62 (44, 78) | 43 (26, 61) | |
| | | Post-Booster | 31 (17, 49) | 38 (22, 57) | 0.5 (0.1, 1.9) | | 60 (41, 77) | 47 (29, 65) | 0.8 (0.2, 2.8) |
| Equipment Use for LBP[k] — no. (%) | | Pre-Booster | 86 (70, 94) | 86 (70, 94) | | | 71 (53, 85) | 76 (58, 88) | |
| | | Post-Booster | 63 (45, 77) | 59 (41, 74) | 1.2 (0.3, 4.5) | | 61 (43, 77) | 63 (45, 78) | 1.2 (0.4, 3.8) |
| Current Medication Use for LBP[k] — no. (%) | | Pre-Booster | 27 (18, 39) | 45 (32, 58) | | | 49 (36, 63) | 39 (27, 52) | |
| | | Post-Booster | 22 (14, 33) | 46 (34, 60) | 0.7 (0.3, 1.8) | | 34 (23, 48) | 35 (24, 48) | 0.6 (0.2, 1.7) |

[a] Model based estimates. LS Means = least square means.
[b] Pre-Booster represents follow-up months 1 through 6; Post-Booster represents follow-up months 7 through 12.
[c] Model based change difference: Booster – No-Booster.
[d] Transformed scale.
[e] Scores on the modified Oswestry Disability Questionnaire range from 0%-100%; 100% represents the highest level of limitation.[1]
[f] Numeric Pain Rating Scale (NRS) ranges from 0-10 with higher scores indicating more pain. [2]
[g] A flare-up is an increase in symptoms of at least 2 points on the NRS above a participant's typical low back pain that lasts for at least 2 consecutive days. Participants provided the number of acute flare-ups in the past 6 months, the length of the flare-ups (days), and the average pain intensity (NRS) during the flare-ups. [3,4]
[h] 36-Item Short Form Health Survey (SF-36) Physical and Mental Component Summary scores range from 0-100 with higher scores indicating better physical or mental health. [5-7]
[i] Stanford Presenteeism Scale Work Impairment Score ranges from 10-50 with 50 indicating the highest degree of impairment. The Work Output Score is the participant's estimate of the percentage of his usual productivity level during work over the past 4 weeks (0-100%).[8,9]
[j] Adherence to home program ranges from 0-100% with higher values indicating higher adherence to treatment. Participants reported weekly adherence during the treatment phase and monthly adherence during the follow-up phase. [10,11]
[k] Absenteeism from usual activities, the Stanford Presenteeism Scale Work Absenteeism Score, health professional care seeking for LBP, and equipment use for LBP contained many 0 records which required transformation to a dichotomous scale.

© 2020 van Dillen LR et al. *JAMA Neurology*.

**eMethods. Detailed Descriptions of Motor Skill Training and Strength and Flexibility Exercise Treatment Conditions**

**Table of Contents**


Overview of the Trial (Both Conditions) .......... 5

Operational Definitions .......... 5

**Motor Skill Training Condition** .......... 6

- Overview and Goals of Motor Skill Training Treatment Condition .......... 6
- Overview of Motor Skill Training .......... 6
- Goals of the Motor Skill Training .......... 6
- Educational Principles .......... 7
- Guiding Principles of Motor Skill Treatment .......... 8
- General Principles of Treatment for all LBP Classifications .......... 10
- Principles of Treatment: LBP Classifications .......... 10
- Prescription of Motor Skills .......... 13
  - Initial Prescription of Motor Skills. .......... 13
  - Overall Prescription Guidelines for Motor Skills .......... 14
  - Grading Motor Skills to Challenge Motor Capabilities .......... 15
  - Progressing Motor Skills .......... 15
  - Guidelines for Assessment of Independence in Performance of Motor Skills during Treatment Sessions .......... 16
  - Providing Feedback during Practice .......... 17
  - General Comments .......... 17
  - Prescription at completion of the 6 week treatment phase and at completion of the booster phase: .......... 18
  - Procedures if participant reports a worsening of symptoms .......... 19
- Motor Skill Descriptions .......... 19

**Strength and Flexibility Exercise Treatment Condition** .......... 33

- Overview and Goals for Strength and Flexibility Exercise Treatment .......... 33
- Overview of Strength and Flexibility Exercise Treatment .......... 33
- Goals of the Strength and Flexibility Exercise Treatment .......... 33
- Educational Principles .......... 33
- Procedures for Administration of Strength and Flexibility Exercise Protocol .......... 34
  - Prescription Guidelines for Strength and Flexibility Exercise .......... 34
  - Progressing Strength and Flexibility Exercise .......... 35


© 2020 van Dillen LR et al. *JAMA Neurology*.

Guidelines for Assessment of Independence in Performance of Treatment Items during Treatment Sessions ....36
Specific procedures by treatment visit ....37
General comments ....39
Prescription at completion of the 6 week treatment phase and at completion of the booster phase: ....39
Procedures if participant reports a worsening of symptoms ....39
Strength and Flexibility Exercise Descriptions ....40
Suggestions for modifications ....43
Educational Principles Quiz Motor Skill Training Condition ....46
Educational Concepts Quiz Strength and Flexibility Exercise Condition ....47
**Motor Skill Training Skill Handouts** ....48
**Strength and Flexibility Exercise Handouts** ....157

Overview of the Trial (Both Conditions)

The therapist should review what will happen as the participant progresses through the study to reinforce what was described previously. The therapist should emphasize what will occur during the treatment phase and the possibility of a booster condition.

1. Six 1 hour treatment sessions will be provided on a weekly basis.
2. The participant will be progressed based on his response to treatment and ability to understand and perform the treatments prescribed.
3. At the completion of the 6 treatment sessions the participant will have a home program of exercises that he can perform on his own.
4. The participant will be expected to continue with the home program after discharge. The therapist should encourage continued participation in the home program.
5. When the participant returns to the lab at the 6 month time point, he will be randomized to either (1) receive additional treatment (booster condition), or (2) continue to be followed without additional treatment (no booster condition).
6. Participants randomized to the booster condition will be scheduled with the therapist who originally provided treatment. Additional treatments (up to 3) will be provided until the participant is independent in the home program he was discharged with at the end of the treatment phase of the study.

Operational Definitions

A. Area of symptoms (Sxs)
    1. Low back:
        a. Sxs in the area extending from T12 to the gluteal fold
    2. Proximal lower extremity (LE):
        a. Sxs in the area extending from the gluteal fold to the knee
    3. Distal LE:
        Sxs in the area extending from the knee to the foot
B. Symptom behavior and categorization
    1. Sxs:

© 2020 van Dillen LR et al. *JAMA Neurology*.

a. Any report of pain, paresthesias, weakness, or clumsiness

2. Paresthesia:
    a. Any subjective sensation experienced as numbness, tingling, a "pins and needles feeling", burning, pricking, tickling
3. Radiation:
    a. Term to describe sx behavior when sxs extend beyond the gluteal fold; can be unilateral or bilateral (this should be noted)

C. Symptom Behavior with Performance of Home Exercise Program
1. Muscle soreness
    a. Tenderness or pain in the muscle that is not consistent with the participant's reported LBP symptoms. Muscle soreness is expected with the performance of new exercises.
2. Worsening symptoms
    a. Report of an increase in the intensity of LBP symptoms or peripheralization of symptoms that lasts for greater than 4 hours and has not fully resolved after 24 hours.
        (1) Peripheralization: Symptoms located more laterally and distally from original location.

**Motor Skill Training Condition**

**Overview and Goals of Motor Skill Training Treatment Condition**

After providing a brief Overview of the Trial (see overview of the trial above), the therapist should verbally provide the Overview and Goals of the Motor Skill Training treatment condition to the participant and answer any questions the participant may have regarding this content. The goals for the 2 treatment conditions differ based on the nature of the treatments. Both treatment conditions are focused on increasing a participant's independence in performance of daily functional activities that have been restricted due to his LBP. The Motor Skill Training Condition does this through direct, challenging practice of modifications of skills that are difficult and/or painful to perform due to the person's LBP condition. Within the Motor Skill Training Condition, the goals are the same across the 5 potential LBP classification groups to be treated.

**Overview of Motor Skill Training**

Explain to the participant that:

1. he has been randomized into the treatment condition that focuses on improving his ability to perform everyday functional activities that are painful or difficult to perform because of his LBP by providing novel, challenging practice of those activities.
2. the specific directions of symptom-provoking movements and alignments identified during the examination are used to classify his LBP and that this information will be used to analyze and modify how he is performing everyday activities that are symptom provoking.
3. the practice will be matched to his capabilities and will simulate the challenges the participant is faced with in his everyday life.
4. he will get to assist in choosing the activities to be practiced.
5. progression of the practice will be based on his ability to 1) modify the skill, and 2) control his symptoms.

**Goals of the Motor Skill Training**

The participant will be:

1. independent in understanding how movements and alignments in a specific direction that are used during daily activities appear to contribute to the persistence or recurrence of his long-standing LBP problem.

© 2020 van Dillen LR et al. *JAMA Neurology*.

2. independent in problem solving and performing modifications of daily functional activities in which he uses the specific, stereotypic movements and alignments, particularly those that are symptom-provoking.
3. independent in controlling and relieving his symptoms during the day with modifications of movements or positions the participant has learned during treatment.

**Educational Principles**

At Visit 1, the therapist will provide the participant with Educational Principles (listed below) specific to the participant's LBP classification. At the beginning of Visit 2, the participant will take a quiz on the Educational Principles (See Educational Principles Quiz Motor Skill Training Condition). The quiz results should be reviewed to reinforce any principles with which the participant needs help. If the participant does not score 100% on the quiz, it should be given at each visit until a 100% score has been attained.

Rotation Classification

- The **primary** contributors to the participant's low back pain symptoms are:
  - **Repeated movements** of the low back into **rotation, side bending, or shifting.**
  - **Prolonged positions** of the low back in **rotation, side bending, or shifting.**
- The repetition of movements and positions of the low back in **rotation, side bending, or shifting** occurs **across the day** during all of the participant's daily activities.
- The **repetition** of movements and positions of the low back in **rotation, side bending, or shifting** keeps the low back region **irritated** and results in low back pain symptoms.
- The low level of irritation of tissues in the low back region that is the result of moving in the **same direction** all day makes it easy for the participant's low back pain symptoms to **persist** or **recur**.
- The **emphasis** of this treatment is on learning how to **modify** or **change** how the participant performs his daily activities so he **doesn't** rotate, side bend, or shift his low back repeatedly across the day.

Extension-Rotation Classification

- The **primary** contributors to the participant's low back pain symptoms are:
  - **Repeated movements** of the low back into **extension and rotation, side bending, or shifting.**
  - **Prolonged positions** of the low back in **extension and rotation, side bending, or shifting.**
- The repetition of movements and positions of the low back in **extension and rotation, side bending, or shifting** occurs **across the day** during all of the participant's daily activities.
- The **repetition** of movements and positions of the low back in **extension and rotation, side bending, or shifting** keeps the low back region **irritated** and results in low back pain symptoms.
- The low level of irritation of tissues in the low back region that is the result of moving in the **same direction** all day makes it easy for the participant's low back pain symptoms to **persist** or **recur**.
- The **emphasis** of this treatment is on learning how to **modify** or **change** how the participant performs his daily activities so he **doesn't** extend and rotate, side bend, or shift his low back repeatedly across the day.

Flexion-Rotation Classification

- The **primary** contributors to the participant's low back pain symptoms are:

© 2020 van Dillen LR et al. *JAMA Neurology*.

    - **Repeated movements** of the low back into **flexion and rotation, side bending, or shifting.**
    - **Prolonged positions** of the low back in **flexion and rotation, side bending, or shifting.**
- The repetition of movements and positions of the low back in **flexion and rotation, side bending, or shifting** occurs **across the day** during all of the participant's daily activities.
- The **repetition** of movements and positions of the low back in **flexion and rotation, side bending, or shifting** keeps the low back region **irritated** and results in low back pain symptoms.
- The low level of irritation of tissues in the low back region that is the result of moving in the **same direction** all day makes it easy for the participant's low back pain symptoms to **persist** or **recur**.
- The **emphasis** of this treatment is on learning how to **modify** or **change** how the participant performs his daily activities so he **doesn't** flex and rotate, side bend, or shift his low back repeatedly across the day.

Extension Classification

- The **primary** contributors to the participant's low back pain symptoms are:
    - **Repeated movements** of the low back into **extension.**
    - **Prolonged positions** of the low back in **extension.**
- The repetition of movements and positions of the low back in **extension** occurs **across the day** during all of the participant's daily activities.
- The **repetition** of movements and positions of the low back in **extension** keeps the low back region **irritated** and results in low back pain symptoms.
- The low level of irritation of tissues in the low back region that is the result of moving in the **same direction** all day makes it easy for the participant's low back pain symptoms to **persist** or **recur**.
- The **emphasis** of this treatment is on learning how to **modify** or **change** how the participant performs his daily activities so he **doesn't** extend his low back repeatedly across the day.

Flexion Classification

- The **primary** contributors to the participant's low back pain symptoms are:
    - **Repeated movements** of the low back into **flexion.**
    - **Prolonged positions** of the low back in **flexion.**
- The repetition of movements and positions of the low back in **flexion** occurs **across the day** during all of the participant's daily activities.
- The **repetition** of movements and positions of the low back in **flexion** keeps the low back region **irritated** and results in low back pain symptoms.
- The low level of irritation of tissues in the low back region that is the result of moving in the **same direction** all day makes it easy for the participant's low back pain symptoms to **persist** or **recur**.
- The **emphasis** of this treatment is on learning how to **modify** or **change** how the participant performs his daily activities so he **doesn't** flex his low back repeatedly across the day.

**Guiding Principles of Motor Skill Treatment**

The following principles will guide the prescription and practice of all motor skills.

© 2020 van Dillen LR et al. *JAMA Neurology*.

| Principle | Implementation in motor skill training treatment condition |
|---|---|
| Practice of a movement results in improvement. | Each skill incorporates the principles of 1) later and less movement of the lumbar region relative to other regions through contraction of groups of classification-specific trunk muscles, 2) earlier and greater movement of other joints (hip, knee, ankle, thoracic region) relative to the lumbar region, 3) avoidance of end-range trunk positions, and 4) no increase in LBP. |
| Large amounts of practice are required to truly master a motor skill. | Participants will practice each skill for ~15-20 minutes per treatment visit.<br>Participants will be given a home program of skills to practice daily.<br>Participants will be asked to incorporate the principles of training from the treatment visit into activities across the day. |
| Learning requires solving the motor problem and not rote repetition of over-learned tasks. | Skills have grades of increasing difficulty.<br>Standardized rules for progressing to more difficult grades are designed to continually challenge the participant's motor capacity. |
| Learning does not occur in the absence of feedback.<br><br>Intrinsic feedback is optimal for promoting self-learning and generalization. | Tasks have clear goals so participants can easily determine knowledge of results.<br><br>LBP behavior during practice will provide intrinsic feedback that can be used to make the appropriate adjustments to a participant's performance. |
| Optimal learning occurs with high levels of motivation and engagement. | Participants will help to select skills for practice to increase engagement and motivation.<br>Participants will practice 3 skills each visit to minimize boredom. |
| Variable practice conditions are optimal for learning and generalization. | Basic necessary movement principles stay the same but contexts change.<br>Variation is accomplished within skills (e.g. sit ↔ stand: vary seat height, seating materials, constraints of surroundings) and across tasks. |
| Massed practice promotes learning better than distributed practice. | The treating environment is set up to allow for continuous practice.<br>Participants are given encouragement by the therapist to continue practicing.<br>Rest breaks are provided at the request of the person or if the participant's LBP symptoms begin to increase. |
| Random practice of several tasks results in better learning than blocked practice of the same tasks in healthy adults. | Participants perform blocks of 10-15 trials of the 3 selected skills in random order at each visit. |

© 2020 van Dillen LR et al. *JAMA Neurology*.

| | |
|---|---|
| This principle is often tested as randomization of small blocks of trials set up to 3 tasks. | |
| Practice of a whole task results in better learning than practice of parts of the task, unless the task can be broken down into clearly separable parts. | Basic underlying movement principles of 1) muscle contraction and 2) timing and grading of movement excursion of the hip, knee, ankle and lumbar/thoracic region are as performed in the real world. |

*Information in column 1 is largely derived from summary by Kleim & Jones 2008[12]. Table adapted from Birkenmeier RL et al., 2010[13].

**General Principles of Treatment for all LBP Classifications**

For all LBP classifications there are general principles that will guide treatment. They include the following:

1. Contraction of groups of specific trunk muscles based on the participant's LBP classification.
2. Timing of movement of lumbar region versus other regions that can contribute to a movement
    a. Later and less movement of the lumbar region compared to movement of the hip, knee, ankle, and/or thoracic region.
3. Smaller angle of end range lumbar region postures; closer to 'neutral' alignment; avoiding postures associated with the participant's LBP classification.
    a. Note: In people who are flexible, end range is easy to determine. If the participant's lumbar region range is limited, then holding to optimal, i.e., neutral, is most important.
4. Movements and postures are performed without an increase in LBP symptoms.

Muscles to be activated and the directions of movement and alignment that need to be modified are specific to the participant's LBP classification.

**Principles of Treatment: LBP Classifications**

Below are the principles of treatment for each LBP classification. Also included are descriptions of proposed common contributing impairments for each classification. Although participants will not be performing any specific exercises to address the impairments, the therapist may want to consider these within the context of the skills a participant is practicing. For example, the therapist might want a participant who has short TFL/ITBs to stand in abduction when practicing turning in the hips rather than the back. The abduction will allow for more hip medial rotation. This participant also may be someone who the therapist would recommend to stand in hip abduction when performing activities in standing.

<u>Rotation Classification</u>

1. Activate all of the trunk muscles to allow trunk posture to be held constant, i.e., as a unit.
    a. Use equipment as need to facilitate support and/or muscle contraction
2. Emphasize getting rotation or side bending in the **thoracic** region and **not** moving in the lumbar region.
    a. Practice turning in the thoracic spine while staying "on axis"
        i. Hypertrophied abdominal muscles can limit rotation and result in the participant rotating "off axis" and beginning to shift rather than rotate
    b. If this is really problematic for the participant to perform, use a lumbosacral support for training purposes
    c. Move in the hips to perform rotation and do not rotate in the trunk

© 2020 van Dillen LR et al. *JAMA Neurology*.

    d. Perform stepping motions in place of hip or trunk rotation if the person has some structural hip variation, e.g., retroversion, anteversion, hip OA
        i. Men with **femoral retroversion** that are active often have TFL/ITB shortness and so lateral hip rotation is limited by **muscle** and medial hip rotation is limited by **structure.** Start practicing the turning motions with the hips abducted so that the hips can move more readily.
3. Move the extremities independent of the trunk.
    a. Use all LE joint movements (in particular, hip movements) and thoracic spine movement more to attain movement goal
4. Avoid repeated movements of (1) lumbar rotation, side bending, or shifting to one side and then the other, or (2) lateral pelvic tilt to one side and then the other during everyday activities.
5. Align the lumbar region in a slight curve with all assumed positions.
    a. Use equipment as needed to attain and maintain alignment
    b. Men who have a tall pelvis should not be forced into a lumbar curve. They often present with an upper lumbar curve that is flexed and a lower thoracic spine that is swayed back, creating what looks like a lumbar curve. They often have an overdeveloped rectus abdominus resulting in a thoracic kyphosis. They then sway back over the lumbar region.
6. Avoid repeated asymmetric position of the trunk or extremities to one side and then the other. Some examples include:
    a. Leaning on one arm and then the other when sitting
    b. Alternating standing on one leg and letting the opposite hip drop
7. Do not allow sitting or standing in a lumbar side bend, rotation, or shift.
    a. In sitting, it is particularly important to practice turning in the thoracic spine while staying “on axis”
        i. Hypertrophied abdominal muscles can limit rotation and result in the participant rotating “off axis” and beginning to shift rather than rotate
8. Common contributing impairments
    a. Imbalance in activation of trunk muscles and limb muscles
        i. Particularly trunk muscles that control rotation
    b. Decreased hip rotation, possibly due to ITB/TFL shortness
    c. Imbalance between hip medial and lateral rotation range of motion, possibly hip retroversion or hip anteversion
    d. Large hip to waist ratio
    e. Body weight that contributes to compression
    f. Hypertrophied abdominals and paraspinals that contribute to compression
    g. Potential excessive abdominal muscle activity **or** excessive paraspinal muscle activity that needs to be considered when cueing to use the trunk muscles, i.e., cue for less abdominal activity and more paraspinal activity or vice versa. It will be indicated on the clinical examination form if a participant presents with excessive abdominal or excessive paraspinal muscle activity so that the therapist can adjust his cueing during practice to accommodate these muscle activity findings.

<u>Extension-Rotation Classification</u>

1. Activate the abdominal muscles and relax the paraspinal muscles (back extensors).
    a. Particularly in upright positions
    b. Flatten the back actively
    c. Sway posture: Sway of the upper trunk backward may be the issue rather than too much activity of the back muscles. In this situation, changing a participant’s

© 2020 van Dillen LR et al. *JAMA Neurology*.

sway posture would be the emphasis rather than reducing paraspinal muscle activity.
    d. Practice activation as frequently possible, i.e., when driving, standing, sitting, etc. The lack of abdominal muscle definition and, therefore, activation, is likely due to repeated activation of the paraspinals and lower extremity muscles to control the pelvis. Turning off muscles (paraspinal and lower extremity muscles) and turning on muscles (abdominals) are **both** important.
    e. Use equipment as needed to facilitate support and/or muscle contraction
2. Move the extremities without anterior pelvic tilt, lumbar extension, rotation, or side bending.
3. Avoid repeated movements of (1) extension with rotation, side bending, or shifting the trunk, or (2) extension with lateral pelvic tilt during everyday activities. Some examples include:
    a. Twisting to put dishes in the dishwasher
    b. Sitting rotated at the dinner table
    c. Twisting the upper body when getting out of the car or when reaching for an item on the opposite car seat
4. Unload the trunk in extension, particularly in upright positions.
    a. Particularly if there is a compression component contributing to a participant's LBP
5. Avoid positioning of the trunk or the extremities asymmetrically. Some examples include:
    a. Leaning on one arm when sitting
    b. Crossing the legs or sitting on one foot
    c. Sitting twisted in a chair
    d. Standing on one leg and letting the other hip drop
6. Common contributing impairments
    a. Imbalance in activation of abdominal muscles and paraspinal muscles with the paraspinal muscle activation being greater than the abdominal muscle activation
    b. Imbalance in activation of lateral abdominal muscles and paraspinal muscles
        i. One side is more frequently activated or activated to a greater magnitude than the other
    c. Imbalance in length of lateral abdominal muscles
    d. Shortness of anterior and lateral hip muscles
    e. Shortness of trunk extensor muscles

<u>Flexion-Rotation Classification</u>

1. Activate the paraspinal muscles.
    a. Particularly in upright positions
    b. Use equipment as needed to facilitate support and/or muscle contraction
2. Move the extremities without lumbar flexion.
    a. In particular, the participant should flex in the hips and not in the lower back
    b. Lack of hip flexion finding: Some people, men in particular, may not have full hip flexion range of motion and the end feel into flexion feels bony. In these people attention must be paid to **adjusting** for the lack of hip flexion motion instead of attempting to gain more hip flexion range, because attempts at increasing hip flexion will result in lumbar flexion.
3. Avoid repeated movements of (1) flexion with rotation, side bending, or shifting the trunk to one side, or (2) flexion with lateral pelvic tilt during everyday activities. Some examples include:
    a. Bending and twisting to put dishes in the dishwasher
    b. Twisting your body to get out of the car

© 2020 van Dillen LR et al. *JAMA Neurology*.

4. Avoid **positioning of the trunk in flexion and positioning of the trunk or the extremities asymmetrically**.
    a. A flexed alignment of the low back will make it easier for the person to assume an asymmetric alignment of the lower back; this is particularly true in men
5. In sitting, in particular, support the lower back in a slightly extended alignment with head and shoulders back over hips. (This will be the alignment we will be testing for when the participant returns for testing at Lab Visit 2.)
    a. More extension may be required to relieve the person's symptoms and to keep the person from assuming an asymmetric alignment of his lower back
    b. Examples of asymmetric positioning include leaning on one arm when sitting, crossing legs or sitting on one foot, sitting twisted in a chair, and standing on one leg and letting the other hip drop
    c. The person should learn to **flex in his hips** and not in his lower back
        i. During practice of the skill "sit to stand to sit," the participant can repeatedly practice flexing in the hips rather than the lower back as part of the Sit to Stand component of the skill
        ii. Some people, men in particular, may not have full hip flexion range of motion and the end feel into flexion feels bony. In these people attention must be paid to **adjusting** for the lack of hip flexion motion instead of attempting to gain more hip flexion range, because attempts at increasing hip flexion will result in lumbar flexion.
6. Common contributing impairments
    a. Imbalance in activation of abdominal muscles and paraspinal muscles with the abdominal muscle activation being greater than the paraspinal muscle activation
    b. Imbalance in activation of lateral abdominal muscles and paraspinal muscles
        i. One side is more frequently activated or activated to a greater magnitude than the other
    c. Imbalance in length of lateral abdominal muscles
    d. Shortness of posterior and lateral hip muscles
    e. Shortness of abdominal muscles

**Prescription of Motor Skills**

**Initial Prescription of Motor Skills.** The skills that initially will be addressed in treatment will be prescribed based on the following criteria:

1. *Required skills.* There are 3 skills in which the participant is **required** to be trained. These include (1) sit to stand to sit, (2) picking up an object and placing it back in the original location, and (3) assumption of a neutral sitting alignment.
    a. These skills have been targeted because we know based on data we have collected in prior studies that these skills typically are problematic for people with long-standing LBP.
    b. The required skills **must** be practiced across the treatment phase because they are the skills that will be measured during Lab Visit 1, Lab Visit 2, and Lab Visit 3 with our laboratory instrumentation.

2. *Matching participant goals with specific skills, i.e., participant-specific skills.* The therapist will discuss with the participant the skills that have been identified during Lab Visit 1 as symptom-provoking or difficult based on the (1) modified Oswestry Disability Index, (2) history portion of the physical examination, and (3) findings from the clinical examination. The participant should identify any other skills that were not noted during Lab Visit 1. Together the therapist and participant will decide on the **priority** skills to

© 2020 van Dillen LR et al. *JAMA Neurology*.

begin to practice and the **level of independence (goal level of function)** the participant wants to attain given his everyday challenges. The participant should practice the specific skill he is having difficulty with. If it is not possible to work on the specific skill due to clinical constraints, then a skill that has the same movement or posture requirements should be practiced instead. The therapist should explain to the participant how this skill relates to the participant's goal. Skills that are symptom-provoking should be prioritized since there is a direct relationship between the participant's ability to perform a skill and his symptoms. If a skill, however, is *very* symptom-provoking, it may be difficult to work on the skill as a whole and the participant may need to practice components of the skill.

a. For example, because of the participant's pain level for the skill of picking up an object and placing the object, a participant may only be able to practice the component of picking up an object. The discussion of skills to practice should include the equipment the participant might need to be independent or the steps to use to attain independence without the use of equipment. *Inclusion of the participant in the decision-making is key to treatment success and adherence, so involving the participant in the process is very important.* Discussion and feedback about treatment progress is allowed. The participant should understand that he is improving in his ability to perform the skills he is practicing.

3. *Clinic Visit 1.* Practice of at *least one* of the *required* skills should be initiated at Clinic Visit 1. The participant also can practice one or two of the participant-specific skills he has identified as symptom-provoking and/or difficult due to his LBP condition. The expectation is that many of the required skills will overlap with participant-specific skills. In this case it would be beneficial to start practice of as many of the required skills as possible before working on other participant-specific skills. The participant should understand how these required skills relate to his specific needs regarding the skills he is interested in working on so that he is motivated to work on the required skills.

**Overall Prescription Guidelines for Motor Skills**

There are 2 aspects to prescription of motor skills for practice at home:

1. *Formal practice of home program*. The therapist determines the number of repetitions of practice of each motor skill when prescribing the home program. The prescription will vary based on the participant's presentation and performance during the treatment visit. In general, for each skill the participant is sent home to practice, the minimum number of repetitions is five, progressing to a maximum of ten repetitions and the minimum number of sets is one, progressing to a maximum of two. Performance of the skills without an increase in symptoms and demonstration of the appropriate method are the criteria for (1) determination of the number of repetitions to perform, and (2) progression (in both numbers of repetitions/sets and in grading). The priority is performance of the skills without an increase in symptoms, followed by demonstration of the appropriate method.
   a. It also is recommended that the participant perform the repetitions of *different* skills at different points during the day rather than practice *all* repetitions for *all* skills at *one* time. The therapist will also want to suggest that the participant initially practice *new* skills at a time when he can focus on his performance. As the participant progresses in the skill he can practice in more difficult circumstances. For example, a participant may find that a skill is more difficult at the end of the day because of fatigue or decreased ability to attend, so the participant initially would practice early in the day. As the participant's performance improves, he could attempt to practice in the more challenging circumstance, i.e., later in the day.

© 2020 van Dillen LR et al. *JAMA Neurology*.

2. *Application of principles taught in treatment to daily functional activities performed across the day.* For each LBP classification, there are principles specific to the classification that guide how the movements and postures the participant uses during daily functional activities should be performed. For example, for the rotation classification, the key principle for a "movement-related" skill is to move the trunk *as a unit* paying close attention to avoiding trunk rotation, side bending, or shifting. This is attained by (1) activating the trunk muscles (anterior, lateral, posterior), and (2) using other joints to attain the movement. This specific principle is taught to the participant during a treatment visit and implemented during the practice of skills during the treatment visit. The participant should also be instructed to implement the same principle(s) during **all** of his functional activities across the day.

**Grading Motor Skills to Challenge Motor Capabilities**

Using information from the baseline assessments, skills will be graded to match the motor capabilities of the participant. The job of the therapist will be to grade tasks such that they challenge, but do not over- or underwhelm, the motor capabilities of the participant. A participant should not be simply repeating tasks that he is already skilled at performing, nor should he repeatedly fail at a task. The goal of the treatment visit is to provide practice of novel, challenging ways to perform skills that are of importance to the participant. It is up to the therapist and participant to determine the level of challenge for practice of individual skills. For many of the skills, a standard set of circumstances are provided that the therapist can use to start practice of a skill.

1. For example, the standard circumstances for initiation of practice of the skill of sit to stand to sit is one in which the participant practices the skill from a stiff surface chair seat, e.g., wood, that is a standard height of 18 inches. If the participant is functioning at a higher level, however, the therapist may initiate practice of the skill at a higher level. If there is doubt about the exact level to start the participant, however, the therapist should start with the standard set of circumstances. If the participant requires more challenge he will move to the next level quickly by performing the skill 5 times without an increase in symptoms while demonstrating the appropriate method.

**Progressing Motor Skills**

The participant can progress in his treatment in three different ways.

1. *Within the home program and application of principles*. For the home program, the participant can progress within a skill by increasing the number of repetitions to perform within a practice session at home. For example, the participant could progress from 5 repetitions to 10 repetitions of practice of sit to stand to sit. The participant also could increase the frequency of application of the principles of treatment to daily activities performed across his day. For example, the participant may progress from applying the principles 50% of the time during a day to applying the principles 80% of the time during a day. Ideally, however, the participant should be applying the principles 100% of the time during the day and this should be encouraged regularly. The goal is for the participant to take responsibility for maintaining his newly acquired skills at the level that keeps him independent and out of an acute flare-up or LBP recurrence.

2. *Grading up a skill within a visit and across visits*. Within a visit, a participant can progress if he is able to perform a skill five times without an increase in symptoms and demonstrating the appropriate method. At this point the therapist can grade the skill up

© 2020 van Dillen LR et al. *JAMA Neurology*.

see (*Guidelines for Assessment of Independence in Performance of Motor Skills during Treatment Sessions*) below.

   a. For example, a participant may be working on sit to stand to sit from a wooden chair of a standard height. If he demonstrates the ability to perform the skill as described above, the skill could be graded up to practice sit to stand to sit from a chair with a soft surface. A participant may be able to grade a skill up more than one time within a visit and across visits. If a participant is unable to verbalize the key principle of a skill, but is able to perform five repetitions of the skill, he can be upgraded within the skill during the session, but cannot be progressed in this skill for his home program as he is not independent in one of the components for independence. The participant must be able to demonstrate appropriate performance AND verbalize the key principle to be upgraded in his home program or “discharged” from a skill.

3. *Changing to a new, different skill*. Skills will be practiced to the level of independence the participant wants to attain given his everyday challenges (goal level of function). For example, a participant may practice sit to stand to sit beginning with a chair with a stiff surface until he is able to perform the skill five times without an increase in symptoms and demonstrating the appropriate method. The skill then would be graded up to practice sit to stand to sit from a chair that has a soft surface until he can perform the skill as described above. Because the participant does not regularly sit in other kinds of chairs the participant can switch to a *new* skill rather than *grading the skill up again*. The new skill should be related to the skills discussed between the therapist and the participant at Clinic Visit 1. If the participant decides he wants to work on a skill different from the original plan, this should be discussed with the therapist.

*Each visit*. At each visit, with each skill practiced, the therapist should check at the beginning of the practice to make sure that the participant’s documented level of independence to be achieved (goal level of function) is the same as previously documented. Occasionally, a participant realizes when practicing at home that a different level of independence is needed during his daily life than what he originally stated.

**Guidelines for Assessment of Independence in Performance of Motor Skills during Treatment Sessions**

1. Assessment of independence in performing the motor skills will be performed at each visit by the treating therapist.
2. Overall procedures for assessing independence with motor skills performance
   a. Assessment includes 3 components
      i. Knowledge of the key concept
      ii. Correct performance without cues and without an increase in symptoms
      iii. Performance of the at least 5 repetitions
   b. The participant must be independent in all 3 components to be able to progress.
   c. The participant will *first* be asked to *identify* the key concept for the skill.
      i. If the participant is able to verbalize the key concept, then the person is *independent* in his knowledge.
      ii. If the participant is not able to verbalize the key concept, the person is not independent in his knowledge.
         1. The key concept is then reviewed.
         2. The participant cannot be progressed in this skill for his home program as he is not independent in one of the components for

© 2020 van Dillen LR et al. *JAMA Neurology*.

independence. However, he can practice more challenging levels of the skill during the treatment session.

d. The participant will then be asked to *perform* the skill.
    i. If the participant is able to perform the skill correctly without any verbal cues or increase in symptoms, the therapist should have the participant continue to perform the skill for five repetitions.
    ii. If the participant is able to perform five repetitions correctly and without an increase in symptoms, then the skill can be progressed as appropriate.
        1. If the participant has reached the goal level of function for that skill, then he can begin practicing a new skill.
    iii. If the participant's performance is *not* independent, the therapist will provide feedback as described below.
        1. The participant cannot be progressed in this skill for his home program as he is not independent in one of the components for independence. However, he can practice more challenging levels of the skill during the treatment session.

**Providing Feedback during Practice**

The following is the suggested sequence for providing feedback during practice:

1. Decide with the participant on the first skill be practiced.
2. Have the participant demonstrate how he performs the skill.
3. Provide the participant with the guiding principles based on his LBP classification and how these apply to the specific skill.
4. Instruct the participant in how to perform the skill applying the principles. The instruction can include visual, tactile, and/or verbal cues – whatever seems appropriate to use to teach the participant a new skill.
5. Have the participant try the skill a few times. Use symptoms to help provide intrinsic feedback to the participant about his performance as you are instructing him in performance.
6. Ask the participant how it felt when he tried to perform the skill in a new way. What was different in terms of (1) pain and (2) movement or posture?
7. If performance was painful or less than optimal, ask the participant what are the principles he needs to attend to with this skill. Focus the participant on the "primary" principle to which he should attend. In most instances, this should be the position of the lumbar spine to be attained and maintained. Other details can be added, such as using the hips and knees more to attain the movement or posture once performance begins to improve.
8. Attempt the skill again and follow the same process.
9. If the performance was successful, increase the repetitions of practice.
10. Move to a new skill when the skill can be performed without an increase in symptoms and is performed with the appropriate methods for the criterion number of repetitions (i.e. 5 reps).
11. If the attempt is not successful, you can:
    a. provide additional cues, for example, visual, tactile, etc.
    b. downgrade the skill to meet the motor capabilities of the participant.
    c. work on separate components of an overall skill.
12. If the participant is continually not successful you may need to find a less difficult skill.

**General Comments**

- Treatment sessions will be 1 hour each

© 2020 van Dillen LR et al. *JAMA Neurology*.

- It is okay for a participant to continue to perform other exercises for back pain that he has received prior to enrollment in this study. However, the participant is asked to refrain from obtaining treatment outside of that provided through the study throughout the duration of the treatment phase of the study (i.e. initial 6 weeks) and during the booster phase of the study.
- It is okay to review previous skills, even if the participant has been deemed independent in that skill.

**Prescription at completion of the 6 week treatment phase and at completion of the booster phase:**

1. *Discharge from treatment phase*. There should be no *new* skills initiated at the time of discharge from the 6-week treatment phase. During this visit, the therapist should practice the skills the participant is still working on from the prior visit. The participant could be *graded up* on a skill during the last visit. To prescribe a skill that has been graded up as a part of the home program on the 6$^{th}$ visit, the participant must be able to meet the target performance for that specific skill.
    a. For example, for the skill Performing Activities in Standing, the participant may be working on the *component* of Performing Activities While Standing in One Place. The participant can be graded up within this component if he can verbalize the key concept and demonstrate the appropriate method for 5 repetitions. The participant will be sent home with a home program of the skills he currently is working on at the grade that he is able to perform without increasing symptoms and that he can perform with the appropriate method.
    b. The participant is to continue *specific practice* of these skills practiced in the 6$^{th}$ visit until he is able to perform the skills without an increase in symptoms and with the appropriate method for the maximum number of repetitions and sets.
    c. In addition, the emphasis at discharge from the treatment phase is on the participant implementing the specific principle(s) he learned in all of his functional activities across the day. In particular, the participant should be encouraged not only to adhere to implementing the principles across his day, but also to use the principles to problem solve *new* situations that he is challenged with. For example, the participant may have a period where he is experiencing an increase in his LBP symptoms, i.e., an acute flare-up, or the participant may start a new job where he is faced with performing new movements and assuming new postures. In these situations, the participant should try to problem solve how to deal with the change in his situation using the principles he learned during the treatment phase of the study.
    d. The participant also should be informed that he can contact his therapist at any time to check in, to ask questions, to report any unusual circumstance regarding his LBP or his general health, or to problem solve a situation. The goal of the treatment phase is for the participant to (1) take responsibility for maintaining his newly acquired skills at the level that keeps him independent and out of an acute flare-up or LBP recurrence, and (2) be able to problem solve new situations using the principles he learned in treatment.

2. *Discharge from booster phase*. The same instructions for discharge from the treatment phase apply to discharge from the booster phase

© 2020 van Dillen LR et al. *JAMA Neurology*.

**Procedures if participant reports a worsening of symptoms**

If the participant reports a worsening of symptoms, the following procedures should be followed. These procedures should be followed if the participant calls to report a worsening of symptoms between visits or if the participant reports, during his visit, that his symptoms were worsened during the previous week.

1. Discuss possible causes of worsening
    a. Rule out change in activity level, specific activities, work conditions, or sleeping habits.
2. If worsening appears to be caused by the home program, the participant should be instructed to stop the specific practice of skills for 24 hours and encouraged to use pain relieving techniques. After the 24 hours have passed, the participant should begin the specific practice of skills, starting with 1 skill only. The participant should add 1 skill per day and report to the therapist if any specific skill increases his symptoms.
3. If a particular skill appears to be the cause of the worsening, the participant will practice the skill with the therapist to assure demonstration of the appropriate method and ability to control symptoms during performance.

**Motor Skill Descriptions**

Listed are the motor skills that that can be worked on during treatment sessions. Each skill is organized into components that the therapist can string together for a given skill. Thus, if the participant cannot perform the whole skill, he can practice components of the skill with the goal of putting all of the components together if appropriate for the participant's desired level of independence (goal level of function). There is information provided regarding options for grading the skill – these are ways to make the skill more or less challenging for the participant.

There are three required skills that every participant must practice sometime during the 6 week treatment phase – sit to stand to sit, picking up an object and placing it back in the original location, and assumption of a neutral sitting alignment.

**General Instructions for Motor Skill Descriptions**

1. The statement "Using appropriate alignment of trunk and extremities with positions and appropriate mechanics with movements" in the Methods section of each motor skill description refers to the details of movement or alignment specific to the participant's LBP classification. These details are provided previously in this manual.

2. An abdominal brace or tape can be used with any skill as indicated based on the participant's LBP classification and severity level. Performing the skill without the brace or tape would make the skill more difficult. The decision to use or not use a brace or tape could be used in grading the skill. Some participants may need to always use a brace or tape for performance of specific skills.

3. During a treatment visit positioning materials should be available for the participant. This gives the participant the opportunity to choose whether or not to use the materials as part of the decision-making process for performing a skill. These can include items such as a towel roll, a lumbar roll, a lumbar support, pillows, step stools for support, etc.

**Skills**

1. Sit to stand to sit (**required skill**)
2. Picking up an object and placing object in same or different location (**required skill**)
3. Donning a sock

© 2020 van Dillen LR et al. *JAMA Neurology*.

4. Performing activities from a seated position (includes appropriate sitting posture) (**required skill**)
5. Getting into and out of a car
6. Performing activities from a standing position (includes appropriate standing posture)
7. Bed mobility
8. Getting in and out of bed
9. Walking
10. Ascending and descending stairs
11. Getting up and down from the floor and performing activities while on the floor
12. Golf
13. Lunge
14. Plank position
15. Bending over

**Skill:** Sit to stand to sit (**required skill**)
Materials:
1. Chairs with and without arm rests that vary in height and depth
2. Seating materials that vary in firmness
3. Positioning materials (lumbar support, etc.)
4. Abdominal brace or tape if needed

All Methods:
Using appropriate alignment of trunk and extremities with positions and appropriate mechanics with movements, participant:

*Component*: Sit to stand
1. Assumes sitting with appropriate alignment of lower extremities and trunk using materials as needed
2. Moves chair to appropriate position to stand
3. Moves forward in chair
4. Moves from sit to stand

*Component*: Stand to sit
1. Moves chair to appropriate position to sit
2. Positions himself appropriately in front of chair
3. Moves from stand to sit
4. Moves back in chair
5. Assumes sitting with appropriate alignment of lower extremities and trunk using materials as needed

Grading:

1. Chair height can vary; standard chair height = 18 inches
    a. Lower chairs would be more challenging for taller people
    b. Higher chairs would be more challenging for shorter people
2. Chair seating can vary; standard seating = stiff
    a. Participant's LBP classification will determine what would be more challenging
3. Chair depth can vary; standard chair depth = 18 inches
    a. Shallower chairs would be more challenging for taller people
    b. Deeper chairs would be more challenging for shorter people
4. Arm rests can vary in height and presence
    a. Lower arm rest heights would be more challenging
    b. Performing without arm rests would be more challenging

© 2020 van Dillen LR et al. *JAMA Neurology*.

5. Chair backing can vary; standard = chair with back
    a. No back rest would be more challenging
6. Mobility between sit to stand and stand to sit can vary
    a. Standing, walking, and sitting in another chair would be more challenging
    b. Varying the types of chairs participant is moving out of and into as described above (items 1-5) would be more challenging
7. Space constraints around chair can vary
    a. Less space around chair would be more challenging

8. Speed of performance can be increased/decreased
    a. Faster would be more challenging
9. Presence of visual feedback can vary
    a. Performance without a mirror would be more challenging
10. Use of abdominal brace or tape
    a. Performing without the brace or tape would be more challenging

**<u>Skill</u>:** Picking up an object and placing an object in same or different location
(This includes lifting up an object and placing it back in its original location; **required skill**)
<u>Materials</u>:
1. Objects of different sizes, e.g., crates, grocery bags, suitcases
2. Weights: 1-20 pounds
3. Foot stools of varying heights (to position objects on)
4. Desk or shelf at waist height
5. Abdominal brace or tape if needed

<u>All Methods</u>:
Using appropriate alignment of trunk and extremities with positions and appropriate mechanics with movements, participant:

*Component*: Picking up object
1. Stands the appropriate distance in front of object placed directly in front of him
2. Positions his feet shoulder width apart
3. Lifts object

*Component*: Placing object in same or different location
1. Positions himself the appropriate distance in front of the object
2. Places object back in original location or moves object to a different location

<u>Grading</u>:
1. Weight of the object can be increased/decreased
    a. Heavier object would be more challenging
2. Original positioning of object and placement of object can vary
    a. Original positioning of object off of midline or farther away is more challenging
    b. Original positioning of object at varying heights could also be more challenging
        i. Participant’s LBP classification will determine what would be more challenging
    c. Placement of object off of midline or farther away is more challenging
        i. Placing object back in original location would be easier than placing in another location where the participant has to be aware of trunk mechanics, e.g., twisting
3. Speed of performance can be decreased/increased
    a. Faster would be more challenging

© 2020 van Dillen LR et al. *JAMA Neurology*.

4. Presence of visual feedback can vary
    a. Performance without a mirror would be more challenging
5. Use of abdominal brace or tape
    a. Performing without the brace or tape would be more challenging

**<u>Skill</u>:** Donning a sock
<u>Materials</u>:
1. Chairs that vary in height
2. Seating materials that vary in firmness
3. Positioning materials (lumbar support, etc.)
4. Typical sock and variety of sock choices
5. Abdominal brace or tape if needed

<u>All Methods</u>:
Using appropriate alignment of trunk and extremities with positions and appropriate mechanics with movements, participant:

*Component*: Initiation phase of donning sock
1. Assumes sitting using materials as needed
2. Moves forward in chair
3. Brings foot to be donned up toward trunk

*Component*: Completion phase of donning sock
1. Dons sock
2. Returns donned foot to starting position
3. Moves back in chair
4. Assumes sitting in chair using materials as needed

<u>Grading</u>:
1. Chair height can vary; standard chair height = 18 inches
    a. Lower chairs would be more challenging for taller people
    b. Higher chairs would be more challenging for shorter people
2. Chair seating can vary; standard seating = stiff
    a. Participant's LBP classification will determine what would be more challenging
3. Amount of movement of leg to be donned and movement of back can vary
    a. More leg movement without back movement would be more challenging
4. Chair depth can vary; standard chair depth = 18 inches
    a. Shallower chairs would be more challenging for taller people
    b. Deeper chairs would be more challenging for shorter people
5. Type of sock can vary
    a. Tighter sock would be more challenging
6. Chair backing can vary; standard = chair with back
    a. No back rest would be more challenging
7. Arm rests can vary in height and presence
    a. Lower arm rest heights would be more challenging
    b. Performing without arm rests would be more challenging
8. Don one or two feet
    a. Donning both feet would be more challenging
9. Speed of performance can be increased/decreased
    a. Faster would be more challenging
10. Presence of visual feedback can vary
    a. Performance without a mirror would be more challenging

© 2020 van Dillen LR et al. *JAMA Neurology*.

11. Use of abdominal brace or tape
    a. Performing without the brace or tape would be more challenging

**<u>Skill</u>:** Performing activities from a seated position (includes appropriate sitting posture; **required skill**)

<u>Materials</u>:

1. Chairs with and without arm rests and with and without chair backs that vary in height and depth
2. Seating materials that vary in firmness
3. Foot stools of varying heights (to position feet)
4. Objects of varying shapes and weights (e.g., file folders, books, etc.)
5. Positioning materials (lumbar support, etc.)
6. Abdominal brace or tape if needed

<u>All Methods</u>:
Using appropriate alignment of trunk and extremities with positions and appropriate mechanics with movements:

*Component*: Assume sitting

1. Participant assumes sitting using materials as needed
2. Participant positions himself relative to work surface for a given task appropriately

*Component:* Manipulate objects or reach for objects on work surface

1. Therapist places objects on work surface directly in front of participant
2. Participant performs manipulation or reaching from the seated position

*Component*: Manipulate objects or reach for objects outside of work surface (e.g. on floor, in file cabinet, etc.)

1. Therapist places objects outside of work surface
2. Participant performs manipulation or reaching from the seated position

<u>Grading</u>:

1. Chair height can vary; standard chair height = 18 inches
    a. Lower chairs would be more challenging for taller people
    b. Higher chairs would be more challenging for shorter people
2. Chair seating can vary; standard seating = stiff
    a. Participant's LBP classification will determine what would be more challenging
3. Placement of objects to reach for and manipulate can vary
    a. Objects above or below waist height would be more challenging
    b. Objects to either side of midline would be more challenging
    c. Objects farther away from the participant would be more challenging
4. Size and weight of objects can vary
    a. Larger and heavier objects would be more challenging
5. Chair depth can vary; standard chair depth = 18 inches
    a. Shallower chairs would be more challenging for taller people
    b. Deeper chairs would be more challenging for shorter people
6. Chair backing can vary; standard = chair with back
    a. No back rest would be more challenging
7. Speed of performance can be increased/decreased
    a. Faster would be more challenging

8. Presence of visual feedback can vary

© 2020 van Dillen LR et al. *JAMA Neurology*.

a. Performance without a mirror would be more challenging

9. Use of abdominal brace or tape
   a. Performing without the brace or tape would be more challenging

**Skill:** Getting into and out of a car

Materials:

1. Car
2. Positioning materials (lumbar support, etc.)
3. Abdominal brace or tape if necessary

All Methods:

Using appropriate alignment of trunk and extremities with positions and appropriate mechanics with movements, participant:

*Component*: Approaching car and sitting on side of car seat

1. Opens door and adjusts seat and steering wheel
2. Positions himself appropriately at side of car
3. Lowers himself into seat so his feet are on the ground and he is facing out of car
4. Moves himself further back into the seat (while still facing out of car)

*Component*: Moving into seated position to face forward in car

1. Moves trunk and legs to position himself facing forward in the seat
2. Assumes position behind the wheel (if driver's side) or facing forward in passenger seat using materials as needed
3. Adjusts seat position and steering wheel (if driver's side)

*Component*: Moving out of seated position to face out of car

1. Adjusts seat and steering wheel (if driver's side) to exit the car
2. Removes any materials used for seating if needed to exit car
3. Moves to side of seat (while still facing forward in car)
4. Moves trunk and legs so that he is facing out of the car with feet on the ground

*Component*: Moving from sitting on side of car seat to standing

1. Moves forward to edge of seat
2. Moves from sit to stand

Grading:

1. Side of car to sit in can vary
   a. Getting into driver's seat would be more challenging
2. Height of car can vary
   a. Participant's LBP classification will determine what would be more challenging
3. Types of seating can vary
   a. Participant's LBP classification will determine what would be more challenging
4. Speed of performance can be increased/decreased
   a. Faster would be more challenging
5. Presence of visual feedback can vary
   a. Performance without a mirror would be more challenging
6. Use of abdominal brace or tape
   a. Performing without the brace or tape would be more challenging

**Skill:** Performing activities in standing (Includes performing activities in standing in one place or performing activities in one place and then moving to another place)

© 2020 van Dillen LR et al. *JAMA Neurology*.

Materials:
1. Counter with overhead shelves or stacking shelves to simulate overhead counter
2. Height-adjustable table
3. Crate and dish rack to simulate dishwasher
4. Stacking steps
5. Clothes/towels/sheets of varying sizes
6. Box with lid to simulate clothes dryer
7. Objects of varying shapes and weights including objects to simulate manual work-related activities
8. Positioning materials (lumbar support, etc.)
9. Abdominal brace or tape if needed

All Methods:
Using appropriate alignment of trunk and extremities with positions and appropriate mechanics with movements:

*Component*: Activities while standing in one place
1. Therapist places object(s) on counter of standard height directly in front of participant
2. Participant assumes standing using materials as needed
3. Participant performs activities in standing
    a. Nature of the activities participant practices will vary depending on the participant-specific needs

*Component*: Activities requiring standing and walking
1. Therapist places object(s) on counter of standard height directly in front of participant
2. Participant assumes standing using materials as needed
3. Participant performs activities in standing that requires participant to walk to another location

Grading:
1. Height of work surface can vary, standard = 34-37 inches
    a. Participant's LBP classification will determine what would be more challenging
2. Height of surface to move objects to can vary
    a. Higher and lower surfaces would be more challenging
3. Size and weight of items to move can vary
    a. Larger and heavier objects would be more challenging
4. Placement of objects to reach for and move/manipulate can vary
    a. Objects above or below waist height would be more challenging
    b. Objects to either side of midline would be more challenging
    c. Objects farther away from the participant would be more challenging
    d. Objects to be placed in another location where the participant has to walk would be more challenging
5. Length of time to perform can vary
    a. Longer time would be more challenging
6. Presence of visual feedback can vary
    a. Performance without a mirror would be more challenging
7. Use of abdominal brace or tape
    a. Performing without the brace or tape would be more challenging

**Skill:** Bed mobility

Materials:
1. Plinth/bed
2. Pillows

© 2020 van Dillen LR et al. *JAMA Neurology*.

3. Covers
4. Surface materials for plinth/bed that vary in firmness
5. Positioning materials (lumbar support, etc.)
6. Abdominal brace or tape if needed

<u>All Methods</u>:
Using appropriate alignment of trunk and extremities with positions and appropriate mechanics with movements, participant:

*Component*: Assume supine, move from supine to side lying, assume side lying, move from side lying to supine
1. Lies on his back with legs extended using materials as needed
2. Removes any materials used for positioning in supine if indicated
3. Adjusts covers
4. Slides each heel up the bed one at a time to a position of ~ 50° of hip flexion and 90° of knee flexion
5. Log rolls to side lying side leading with his upper arm and pushing his feet into the plinth/bed
6. Assumes side lying using materials as needed
7. Removes any materials used for positioning in side lying if indicated
8. Log rolls from side lying to supine
9. Assumes supine position as in #1

*Component*: Assume side lying, move in bed while side lying
1. Assumes side lying with hips and knees flexed to ~ 60° of hip flexion and 90° of knee flexion using materials as needed
2. Removes any materials for positioning in side lying if indicated
3. Adjusts covers
4. Grasps the side of the plinth/bed with his upper hand, pulls with his hand, and pushes into the plinth/bed with the lower leg **moving forward** towards the edge of the plinth/bed
5. Pushes with his hand and pushes into the plinth with his lower leg **moving back away** from the edge of the plinth/bed
6. Assumes side lying as in #1

*Component*: Assume side lying, move from side lying to prone, assume prone, move from prone to side lying
1. Assumes side lying with hips and knees flexed to ~ 60° of hip flexion and 90° of knee flexion using materials as needed
2. Removes any materials used for positioning in side lying if indicated
3. Adjusts covers
4. Log rolls from side lying to prone
5. Positions any materials needed in prone
6. Removes any materials used for positioning in prone if indicated
7. Log rolls from prone to side lying
8. Assumes side lying as in #1

*Component*: Assume supine, move in bed while supine
1. Lies on his back with legs extended using materials as needed
2. Removes any materials used for supine if indicated
3. Adjusts covers
4. Slides each heel up the plinth/bed one at a time to a position of ~ 50° of hip flexion and 90° of knee flexion
5. Flexes both elbows to ~ 90°

© 2020 van Dillen LR et al. *JAMA Neurology*.

6. Pushes down with elbows and feet at the same time and moves body to side in small increments, keeping hips in line with shoulders

Grading: Participant will start with the surface that is closest to that which is difficult for him. If the participant cannot perform appropriately on this surface he can be downgraded to a firmer surface.
1. Plinth/bed surface can vary; standard surface = plinth surface
    a. Less stiff materials would be more challenging
2. Presence and weight of covers can vary; standard = sheet
    a. Heavier covers would be more challenging
3. Amount of leg movement can vary
    a. More leg movement without back movement would be more challenging
    b. Sliding both heels up or down together would be more challenging
4. Side (right or left) to roll towards can vary
    a. Rolling to atypical side would be more challenging
5. Speed of performance can be increased/decreased
    a. Faster would be more challenging
6. Presence of visual feedback can vary
    a. Performance without a mirror would be more challenging
7. Use of abdominal brace or tape
    a. Performing without the brace or tape would be more challenging

**Skill:** Getting in and out of a bed

Materials:
1. Plinth /bed
2. Pillows
3. Covers
4. Surface materials for plinth/bed that vary in firmness
5. Positioning materials (lumbar support, etc.)
6. Abdominal brace or tape if needed

All Methods:
Using appropriate alignment of trunk and extremities with positions and appropriate mechanics with movements, participant:

*Component*: Move from preferred sleeping position to side lying, move from side lying to sitting, assume sitting
1. Lies in preferred sleeping position using materials as needed
2. Removes any materials used for preferred sleeping position if indicated
3. Adjusts covers
4. Log rolls to side lying from preferred sleeping position
5. Grasps the side of the plinth/bed while side lying, as long as no rotation noted in low back
6. Pushes with his lower arm and the hand grasping the side of plinth/bed while letting the legs drop over the side of the plinth/bed, moving his trunk as a unit
7. Moves forward to edge of plinth/bed so his feet are positioned on the floor
8. Assumes sitting with his feet flat on the floor

*Component:* Move from sitting to side lying, move from side lying to preferred sleeping position, assume preferred sleeping position
1. Adjusts covers
2. Sits on the side of the plinth/bed with his feet flat on the floor

© 2020 van Dillen LR et al. *JAMA Neurology*.

3. Lowers himself on his elbow while moving his trunk as a unit and brings both legs up to the surface of the plinth/bed
4. Log rolls into preferred sleeping position
5. Assumes preferred sleeping position using materials as needed

Grading:
1. Plinth/bed surface can vary; standard surface = plinth surface
    a. Less stiff materials would be more challenging
2. Presence and weight of covers can vary; standard = sheet
    a. Heavier covers would be more challenging
3. Position on plinth/bed can vary
    a. Farther from the edge of the bed would be more challenging
4. Side (right or left) to move towards can vary
    a. Moving to atypical side would be more challenging
5. Amount of leg movement can vary
    a. More leg movement without back movement would be more challenging
    b. Sliding both heels up or down together would be more challenging
6. Speed of performance can be increased/decreased
    a. Faster would be more challenging
7. Presence of visual feedback can vary
    a. Performance without a mirror would be more challenging
8. Use of abdominal brace or tape
    a. Performing without the brace or tape would be more challenging

**Skill:** Walking
Materials:
1. Surfaces that vary in direction of path
2. Surfaces that vary in consistency
3. Abdominal brace or tape if necessary

All Methods:
Using appropriate alignment of trunk and extremities and mechanics with movements, participant:
1. Places hands on pelvis &/or trunk to monitor alignment and motion
2. Walks a straight path at a self-selected pace and typical step length for the standard distance (50 feet; 15.24 meters)

Grading:
1. Direction of path can vary; standard = straight path
    a. More variation in direction, including making turns, would be more challenging
2. Attention of participant can vary
    a. Divided attention while walking would be more challenging
3. Distance can vary; standard = 50 feet; 15.24 meters
    a. Longer distances would be more challenging
3. Surface can vary; standard = tile or low pile carpeted surface
    a. Uneven surfaces would be more challenging
    b. Softer or more pliable surfaces (e.g. plush carpet, etc.) would be more challenging
4. Speed of performance can be increased/decreased (including progressing to running)
    a. Faster would be more challenging
5. Placement of hands on pelvis &/or trunk to monitor alignment or motion can vary
    a. Performing without cue of hands would be more challenging

© 2020 van Dillen LR et al. *JAMA Neurology*.

6. Step length can vary
    a. Longer step lengths would be more challenging
7. Presence of visual feedback can vary
    a. Performance without a mirror would be more challenging
8. Use of abdominal brace or tape
    a. Performing without the brace or tape would be more challenging\

**<u>Skill</u>:** Ascending and descending stairs

<u>Materials</u>:
1. Stairs of varying heights and depths
2. Available handrail
3. Abdominal brace or tape if necessary

<u>All Methods</u>:
Using appropriate alignment of trunk and extremities and mechanics with movements, participant:
1. Places a hand on pelvis &/or trunk to monitor alignment and motion while holding onto a handrail with the other hand
2. Ascends and descends 5 steps of standard height (7-8 inch rise) and depth (11-12 inch run) using appropriate mechanics

<u>Grading</u>:
1. Total number of stairs can vary; standard = 5 steps
    a. More stairs would be more challenging
2. Use of hand rail can vary
    a. Performing without the support of handrail would be more challenging
3. Attention of participant can vary
    a. Divided attention while ascending and descending stairs would be more challenging
4. Speed of performance can be increased/decreased
    a. Faster would be more challenging
5. Placement of hands on pelvis &/or trunk to monitor alignment or motion can vary
    a. Performing without cue of hands would be more challenging
6. Presence of visual feedback can vary
    a. Performance without a mirror would be more challenging
7. Use of abdominal brace or tape
    a. Performing without the brace or tape would be more challenging

**<u>Skill</u>:** Getting up and down from the floor; performing activities while on the floor

<u>Materials</u>:
1. Carpet pieces or padding that vary in firmness/depth
2. Stools of different heights (to be used to assist with changes of position)
3. Objects of varying shapes and weights
4. Abdominal brace or tape as necessary

<u>All Methods</u>:
Using appropriate alignment of trunk and extremities with positions and appropriate mechanics with movements, participant:

*Component*: Moving from standing to ½ kneeling to full kneeling to ½ kneeling to standing
1. Moves from standing to ½ kneeling on one leg using materials as needed

© 2020 van Dillen LR et al. *JAMA Neurology*.

2. Moves from ½ kneeling on one leg to full kneeling
3. Moves from full kneeling to ½ kneeling on opposite leg using materials as needed
4. Moves from ½ kneeling on one leg to standing using materials as needed

*Component*: Moving from kneeling to quadruped, moving materials in quadruped
1. Moves from kneeling to quadruped using materials as needed
2. Shifts weight onto one arm and sits back on heels if appropriate
3. Reaches for object placed at arm's length and directly in front of participant (standard position)
4. Places object directly in front of himself
5. Assumes quadruped with weight on both arms

*Component*: Moving from quadruped to sitting on heels to full kneeling to ½ kneeling to standing
1. Moves from quadruped to sitting on heels
2. Moves from sitting on heels to full kneeling
3. Moves from full kneeling to ½ kneeling
4. Moves from ½ kneeling to standing

Grading:
1. Number of repetitions of moving from ½ kneeling to full kneeling to ½ kneeling before standing can vary
    a. More repetitions would be more challenging
2. Size and weight of items to move can vary
    a. Larger and heavier objects would be more challenging
3. Original position of object and placement of object can vary
    a. Original position of object farther away or off of midline would be more challenging
    b. Placement of objects farther away or off of midline would be more challenging
        i. Placing object back in original location would be easier than placing object in another location where the participant has to be aware of mechanics
4. Length of time to perform can vary
    a. Longer time would be more challenging
5. Speed of performance can be increased/decreased
    a. Faster would be more challenging
6. Presence of visual feedback can vary
    a. Performance without a mirror would be more challenging
7. Use of abdominal brace or tape
    a. Performing without the brace or tape would be more challenging

**Skill:** Golf
Materials:
1. Golf clubs of various heights and types
2. Abdominal brace or tape as necessary

All Methods:
Using appropriate alignment of trunk and extremities with positions and appropriate mechanics with movements, participant:

*Component*: Swinging golf club
1. Stands next to ball with golf club in hands

© 2020 van Dillen LR et al. *JAMA Neurology*.

2. Pulls golf club backwards
3. Swings golf club

*Component*: Picking up golf ball
1. Stands next to golf ball with golf club in hand
2. Squats down to pick up golf ball --OR--
3. Bends over with one leg up and back behind, using golf club for support
4. Picks up golf ball

Grading:
1. Grip on golf club can vary
2. Type of golf club can vary
3. Speed of performance can be increased/decreased
    a. Faster would be more challenging
4. Presence of visual feedback can vary
    a. Performance without a mirror would be more challenging
5. Use of abdominal brace or tape
    a. Performing without the brace or tape would be more challenging

**Skill:** Lunge
Materials:
1. Abdominal brace or tape as necessary

All Methods:
Using appropriate alignment of trunk and extremities and mechanics with movements, participant:
1. Places hands on pelvis &/or trunk to monitor alignment and motion
2. Steps forward with one leg and shifts weight onto the front leg (optional hold in this position)
3. Shifts weight towards back leg and steps back with the front leg

Grading:
1. Length of time to hold can be increased/decreased
    a. Longer time would be more challenging
2. Length of step forward can be increased/decreased
    a. Bigger step would be more challenging
3. Type of repetition can vary
    a. Alternating putting right and left leg forward would be more challenging (as compared to repeated repetitions of stepping forward with the same leg)
4. Speed of performance can be increased/decreased
    a. Faster would be more challenging
5. Presence of visual feedback can vary
    a. Performance without a mirror would be more challenging
6. Use of abdominal brace or tape
    a. Performing without the brace or tape would be more challenging

**Skill:** Plank position
Materials:
1. Abdominal brace or tape as necessary

All Methods:
Using appropriate alignment of trunk and extremities with positions and appropriate mechanics with movements, participant:

© 2020 van Dillen LR et al. *JAMA Neurology*.

1. Starts to assume the position by sitting on heels
2. Leans forward and places hands on surface in front of participant
3. Tucks toes under and straightens legs so that knees are no longer on the surface
4. After holding in the plank position, participant lowers knees to ground and pushes back with hands until s/he is sitting on heels

Grading:
1. Hand position can vary
    a. On hands (vs. on elbows) would be more challenging
2. Leg position can vary
    a. On feet (vs. on knees) would be more challenging
3. Length of time of hold can be increased/decreased
    a. Longer would be more challenging
4. Presence of visual feedback can vary
    a. Performance without a mirror would be more challenging
5. Use of abdominal brace or tape
    a. Performing without the brace or tape would be more challenging

**Skill:** Bending over
Materials:
1. Surface to hold onto for support as necessary
2. Abdominal brace or tape as necessary

All Methods:
Using appropriate alignment of trunk and extremities with positions and appropriate mechanics with movements, participant:
1. Stands with feet about hip width apart with weight even between both legs
2. Bends forward at the hips, keeping the back straight, letting arms hang comfortably as participant bends forward
3. Stands back up by squeezing buttock muscles and bringing buttocks underneath him/her

Grading:
1. Distance moved can vary
    a. Bending over farther (closer to 90 degrees at hip) would be more challenging
2. Number of repetitions can vary
    a. Repeated repetitions would be more challenging
3. Speed of performance can be increased/decreased
    a. Faster would be more challenging
4. Presence of visual feedback can vary
    a. Performance without a mirror would be more challenging
5. Use of abdominal brace or tape
    a. Performing without the brace or tape would be more challenging

© 2020 van Dillen LR et al. *JAMA Neurology*.

## Strength and Flexibility Exercise Treatment Condition

### Overview and Goals for Strength and Flexibility Exercise Treatment

After providing a brief Overview of the Trial, the therapist should verbally provide the Overview and Goals of the Strength and Flexibility Exercise treatment condition to the participant and answer any questions the participant may have regarding this content. The goals for the 2 treatment conditions differ based on the nature of the treatments. Both treatment conditions are focused on increasing a participant's independence in performance of daily functional activities that have been restricted due to his LBP. The Strength and Flexibility Exercise Treatment does this through increasing the participant's trunk strength and trunk and lower limb flexibility using exercises for trunk strength and trunk and lower extremity flexibility commonly cited in the literature as being appropriate for people with chronic LBP.

### Overview of Strength and Flexibility Exercise Treatment

Explain to the participant that:

1. he has been randomized into the treatment condition that focuses on improving his ability to perform everyday functional activities that are painful or difficult to perform because of his LBP through exercise
2. contributors to his LBP are:
    a. Poor posture
    b. Stressful living and working habits
    c. Loss of flexibility
    d. Loss of strength
    e. General decline of physical fitness
3. benefits of exercise and activity are:
    a. Makes bones stronger
    b. Develops fit muscles
    c. Keeps participant flexible
    d. Makes participant fit
    e. Makes participant feel good
    f. Releases natural chemicals which reduce pain
    g. Data suggests people who are active have less LBP episodes and less severe episodes

### Goals of the Strength and Flexibility Exercise Treatment

The participant will:

1. Reduce symptoms
2. Increase activity to prior level of function
3. Increase ability to manage back pain
4. Increase strength and flexibility
5. Increase independence in performance of daily functional activities that have been restricted due to LBP though exercise.

### Educational Principles

At Visit 1, the therapist will provide the participant with Educational Principles (listed below). At the beginning of Visit 2, the participant will take a quiz on the Educational Principles (See Educational Principles Quiz Strength and Flexibility Exercise Condition). The quiz results should be reviewed to reinforce any principles with which the participant needs help. If the participant

© 2020 van Dillen LR et al. *JAMA Neurology*.

does not score 100% on the quiz, it should be given at each visit until a 100% score has been attained.

1. Contributors to Back Pain
    a. Poor posture
    b. Stressful living and working habits
    c. Loss of flexibility
    d. Loss of strength
    e. General decline of physical fitness

2. Pain Control
    a. Use medications as prescribed by your physician.
    b. Use cold within the first 48 hours of onset to reduce symptoms.
    c. After 48 hours, heat may be used.

3. Coping with Back Pain
    a. It is important to participate in exercise/activity to help cope with your low back pain.

4. Exercise/Activity
    a. Makes your bones stronger
    b. Helps you develop fit muscles
    c. Keeps you flexible
    d. Makes you fit
    e. Makes you feel good
    f. Releases natural chemicals in your body which help reduce pain symptoms
    g. You should gradually build up exercise over a few days or weeks, then continue to exercise regularly.

5. Strength and Flexibility Exercise
    a. Performance of strength and flexibility exercises will improve your performance in the daily functional activities that are limited by your back pain.

6. Muscle Soreness
    a. Muscle soreness is tenderness or pain in your muscles that is not associated with your low back pain symptoms.
    b. Muscle soreness is expected with the performance of new exercises, and can occur anytime within 1-2 days after performing a new exercise.

**Procedures for Administration of Strength and Flexibility Exercise Protocol**

**Prescription Guidelines for Strength and Flexibility Exercise**

1. Treatment is divided into 3 phases with each phase being progressively more difficult.
2. All participants will start with Phase 1 exercises regardless of strength or previous experience with back pain exercises.
3. At each treatment session, the therapist will assess the participant’s independence in performance of the exercises. Specifically, the therapist will determine if the participant has met the criteria to advance to the next phase or if he should remain in the current phase (See Guidelines for Assessment of Independence in Performance of Treatment Items during Treatment Sessions)
4. In order for the HEP to be manageable for the participant, he will perform stretching every other day, then strengthening on the other days.

© 2020 van Dillen LR et al. *JAMA Neurology*.

    a. If the participant would prefer to perform the exercises only 2-3x/week (i.e. the participant chooses to perform both flexibility and strengthening exercises on the same day), this can be done at the discretion of the clinician, particularly if the clinician believes this would improve the participant's adherence. Ideally, the participant would alternate flexibility and strengthening exercises so that he is performing some type of exercise every day.

5. Flexibility – Participant is to perform each stretch with the following parameters: hold 10-30 seconds, 2-4 reps, at least 2-3 times/week.
    a. The maximum number of repetitions for flexibility (for purposes of classifying independence) would be 4 repetitions of 30 second holds for each exercise.
6. Strength – Participant is to perform each exercise with the following parameters: 2-3 sets of 8-10 repetitions; 2-3 times/week.
    a. The maximum number of repetitions for strength (for purposes of classifying independence) would be 3 sets of 10 repetitions, or 30 repetitions, for each exercise.
7. The therapist will provide a total of 2-3 flexibility exercises and 2-3 strength exercises within each session (i.e. a max of 4-6 total exercises for the HEP).
8. The therapist should attempt to incorporate a variety of types of exercise within the exercises prescribed. For example, the participant should not be doing 4 exercises that all work on flexion flexibility and strength. Instead, the participant should do 1 flexibility – flexion, 1 flexibility – rotation, 1 flexibility – quads, 1 strength – extensors.
9. On Visit 1, the participant can only be sent home with Phase 1 exercises, even if he is able to perform the maximum number of repetitions. He is still not independent with the exercise because he has not demonstrated Knowledge of the Key Concept. However, if on visit 2, the participant is able to perform the maximum number of repetitions of a Phase 2 exercise, he is able to be progressed to a Phase 3 exercise.
10. The therapist should provide 2 verbal cues before providing physical assistance with an exercise. If the therapist demonstrates the exercise for the participant, this is considered a verbal cue.
    a. Physical assistance is the therapist placing his hands on the participant to (a) provide tactile cues *to improve performance*, or (b) to *actually physically assist* the participant to perform the exercise correctly.
    b. A participant is rated as requiring physical assistance only if placing "hands on" is necessary to *improve the performance* of the exercise.
        i. For example, the therapist may place his hands on the participant's lumbar region to determine what the alignment is or the movement that is occurring. This would not be considered physical assistance. On the other hand, the therapist may place his hands on the lateral aspects of the participant's abdomen to facilitate a contraction of the lateral abdominals while simultaneously telling the participant to make his abdomen go concave. This would be considered physical assistance.
11. Participants may have mild discomfort with performance of exercise, but this is expected to improve as they progress in their program.
12. If the participant completes all 4-6 exercises within a session and the 60 minute treatment time has not been reached, the therapist can either prescribe more exercises or increase the number of repetitions for the participant to complete.

**Progressing Strength and Flexibility Exercise**

1. Progression of exercise will be based on independence in: knowledge of the key concept, correct performance, and performance of the maximum number of repetitions of each of the exercises.

© 2020 van Dillen LR et al. *JAMA Neurology*.

2. The treatment includes 2 components – strengthening and flexibility. The participant may progress to the next phase in either component or within a treatment item. It is not required that the participant meet the criteria in both components in order to progress to the next phase in one component.
    a. Example: The participant may progress to Phase 2 in his strengthening exercise but remain in Phase 1 for his flexibility exercise.
    b. Example: The participant may progress to Phase 2 for back strengthening but remain in Phase 1 for abdominal strengthening.
3. The participant may complain about muscle soreness if he has completed the maximum number of repetitions of a strength exercise in the last 1-2 days. If that is the case, the therapist can observe a few repetitions of the exercise and use his clinical judgment to decide if the participant is independent in the exercise and safe to progress to the next phase.
4. The participant should only be assessed for the performance of the maximum number of repetitions if that has been prescribed to him as an HEP. If the participant was given less than the maximum as an HEP, he should be progressed to the maximum as an HEP prior to being assessed for performance of the maximum number of repetitions (see Guidelines for Assessment of Independence in Performance of Treatment Items during Treatment Sessions for more information).
5. If a participant has achieved complete independence in an exercise and there is no further progression for that exercise, the participant should continue to perform the exercise at the maximum level. He can do this on his own and the exercise should be considered as ongoing and independent. The participant should not be further progressed by increasing sets, reps, or duration of the exercise.
6. It is okay to review previous exercises, even if the participant has been deemed independent in that exercise.

**Guidelines for Assessment of Independence in Performance of Treatment Items during Treatment Sessions**

1. Assessment of independence in performing the treatment program will be performed at each visit by the treating therapist.
2. Overall procedures for assessing independence to exercises
    a. Assessment includes 3 components
        i. Knowledge of the key concept
        ii. Correct performance without cues
        iii. Performance of the maximum number of repetitions
    b. The participant must be independent in all 3 components to be able to progress.
    c. The participant will *first* be asked to *identify* the key concept for his exercise.
        i. If the participant is able to verbalize the key concept, then the person is *independent* in his knowledge.
        ii. If the participant is not able to verbalize the key concept, the person is not independent in his knowledge.
            1. The key concept is then reviewed.
            2. The participant cannot be progressed in this exercise as he is not independent in one of the components for independence.
    d. The participant will then be asked to *perform* the exercise.
        i. If the participant is able to perform the exercise correctly without any verbal cues, the therapist should have the participant continue to perform the exercise for the *maximum number* of repetitions.

© 2020 van Dillen LR et al. *JAMA Neurology*.

        1. For flexibility exercises, the maximum number of repetitions for independence is 4 repetitions of 30 second holds.
        2. For strengthening exercises, the maximum number of repetitions for independence is 3 sets of 10, or 30 repetitions.
    ii. If the participant is able to perform the maximum number of repetitions, he is considered independent in that exercise and can be progressed as appropriate.
        1. If the participant has reached the maximum number of repetitions and there is no further progression for that exercise, the participant should continue to perform that exercise at the maximum level. Do not try to further progress the participant by increasing sets, reps, or duration of the exercise.
    iii. If the participant's performance is *not* independent, the therapist will stop the participant and provide a verbal cue for correct performance.
        1. The therapist should provide a maximum of 2 verbal cues.
        2. If the participant is able to correctly perform the exercise, his rating for *performance* is at the *verbal cues* level.
        3. If the participant is still unable to correctly perform the exercise, the therapist should provide physical assistance.
            a. If the participant is now able to correctly perform the exercise, his rating for *performance* is at the *verbal cues with physical assistance* level.
        4. The participant continues with this level of exercise until the next visit.
    iv. If the participant is still unable to correctly complete the exercise with verbal cues and physical assistance, the therapist should reassess the exercise and modify as necessary.
        1. Modification will typically be directed at decreasing the demand of the exercise, e.g. decreased ROM, decreased load, change in position.

**Specific procedures by treatment visit**

1. Visit #1 Exercise prescription and HEP instruction
    a. The therapist will instruct the participant in the appropriate method using demonstration, verbal instruction, and/or physical assistance.
    b. The participant should then demonstrate the exercise and the therapist will determine if the participant is able to perform it correctly.
    c. For the HEP, the participant must be able to perform the exercise independently or with verbal cues and report no worsening of symptoms as operationally defined.
        i. If the participant is unable to perform the activity because of discomfort of other joints or difficulty with balance, the activity may be modified. Exercises can also be modified based on the participant's specific needs.
            1. In general, for Phase 1 exercises, if the participant reports a worsening of symptoms as operationally defined, the exercise will be modified by limiting the range of motion of the exercise, decreasing the number of repetitions, or prescribing one of the alternative exercises suggested.
            2. In general, for Phase 2 or 3 exercises, if the participant reports a worsening of symptoms as operationally defined, the exercise will

© 2020 van Dillen LR et al. *JAMA Neurology*.

be modified by limiting the range of motion of the exercise, decreasing the number of repetitions, or returning to the previous phase of the exercise (as appropriate).

ii. Flexibility – Trunk
   1. Criteria for correct performance for HEP
      a. After initial instruction, the participant can perform the exercise independently or with verbal cues.
      b. The participant reports no worsening of symptoms as operationally defined.

iii. Flexibility – Extremity
   1. Criteria for correct performance for HEP
      a. After initial instruction, the participant can perform the exercise independently or with verbal cues.
      b. The participant reports no worsening of symptoms as operationally defined.

iv. Strength – Trunk
   1. Criteria for correct performance for HEP
      a. After initial instruction, the participant can perform a minimum of 5 repetitions independently or with verbal cues. The participant should be able to achieve the same amount of range of motion across all 5 repetitions.
      b. The participant reports no worsening of symptoms as operationally defined.

2. Visits #2-6
   a. The therapist will assess if the participant meets the criteria to progress to Phase 2 or 3 in each of the components.
      i. Education
         1. Participant education will continue as needed.
      ii. Exercise and HEP
         1. The therapist will ask the participant to perform his HEP. For each exercise, the therapist will assess the participant's knowledge of key concepts and the participant's performance. If the participant is independent in those two components, the therapist will assess the participant's performance of the maximum number of repetitions. The therapist will determine if the participant has met the criteria to progress to the next phase (Guidelines for Assessment of Independence in Performance of Treatment Items during Treatment Sessions).
            a. Each exercise will be continued, progressed, or terminated based on performance
            b. Progression of exercise may vary across individual exercises.
               i. Example (Trunk Strengthening): The participant may progress from Posterior Pelvic Tilt of Phase 1 to Trunk Curl of Phase 2, however continue with Single Arm Lift in Prone of Phase 1.
            c. Progression to Phase 2
               i. If the participant meets the criteria to progress to Phase 2, the therapist will instruct in the Phase 2 exercise. See Appendices A and J.

© 2020 van Dillen LR et al. *JAMA Neurology*.

        ii. If the participant does not meet the criteria to progress to Phase 2, the therapist will reinstruct the participant in Phase 1 exercise.

    d. Progression to Phase 3
        i. If the participant meets the criteria to progress to Phase 3, the therapist will instruct in the Phase 3 exercise. See Appendices A and J.
        ii. If the participant does not meet the criteria to progress to Phase 3, the therapist will reinstruct the participant in Phase 2 exercise.

**General comments**

1. Treatment sessions will be 1 hour each.
2. It is okay for a participant to continue to perform other exercises for back pain that he has received prior to enrollment in this study. However, the participant is asked to refrain from obtaining treatment outside of that provided through the study throughout the duration of the treatment phase of the study (i.e. initial 6 weeks) and during the booster phase of the study.

**Prescription at completion of the 6 week treatment phase and at completion of the booster phase:**

1. The therapist should not add any new exercises on the final visit.
2. In addition to the activities listed previously for visits #2-6, the therapist will do the following:
    a. Review the therapist's contact information for the participant to be able to contact the therapist with any questions he may have and remind the participant to call if he develops any symptoms that are unusual compared to his typical symptoms.
    b. Explain what is expected of the participant for follow up (i.e. additional laboratory visits, monthly e-mail questionnaires, possibility of additional treatment.)
    c. Encourage the participant to continue with HEP and with activity.

**Procedures if participant reports a worsening of symptoms**

1. If the participant reports a worsening of symptoms, the following procedures should be followed. These procedures should be followed if the participant calls to report a worsening of symptoms between visits or if the participant reports, during his visit, that his symptoms were worsened during the previous week.
2. Discuss possible causes of worsening
    a. Rule out change in activity level, specific activities, work conditions, or sleeping habits.
3. If worsening appears to be caused by the home exercise program, the participant should be instructed to stop exercises for 24 hours and encouraged to use pain relieving techniques. After the 24 hours have passed, the participant should begin the exercise program, starting with 1 exercise only. The participant should add 1 exercise per day and report to the therapist if any specific exercise increases his symptoms.
4. If a particular exercise appears to be the cause of the worsening, the participant will be instructed in the following:
    a. Phase 1 exercise – limit the range of motion that is performed, decrease the number of repetitions, or attempt an alternative exercise if there is one available.
    b. Phase 2 or 3 exercise – limit the range of motion that is performed, decrease the number of repetitions, or perform the appropriate exercise from the previous phase.

© 2020 van Dillen LR et al. *JAMA Neurology*.

**Strength and Flexibility Exercise Descriptions**

| Treatment Component | Phase 1 | Criteria for progression to Phase 2 |
|---|---|---|
| **Patient education** | | |
| General education | Anatomy and contributors to pain<br>Pain control<br>Benefits of exercise<br>Performance of exercises will improve performance in functional activities<br>Definition of worsening symptoms versus increased muscle soreness | Patient must pass the Educational Concepts quiz with a score of 100%<br><br>Continue education as necessary (e.g. participant needs reminders about appropriate pain control, etc.) |
| **Exercise Prescription** | | |
| Flexibility – Trunk | 1. Lower back flexion in supine<br>2. Trunk rotation in supine hook lying<br>3. Trunk side bend in standing<br>4. Trunk flexion/extension in quadruped<br>5. Trunk extension in prone<br>6. Trunk side stretch<br><br>Hold 10-30 seconds; 2-4 reps; at least 2-3x/week | Patient demonstrates independent performance of the exercise (see Appendix B).<br><br>Patient reports no worsening* of symptoms. |
| Flexibility – Extremity | 1. Hamstring/calf muscle stretch in supine<br>a. Alternative #1: doorway<br>b. Alternative #2: sitting forward lean<br>2. Calf muscle stretch in standing<br>a. Alternative: long sitting with towel<br>3. Gluteal muscle stretch in supine<br>a. Alternative: sitting<br>4. Piriformis muscle stretch in supine | Patient demonstrates independent performance of the exercise (see Appendix B).<br><br>Patient reports no worsening* of symptoms. |

© 2020 van Dillen LR et al. *JAMA Neurology*.

| | | |
|---|---|---|
| | a. Alternative: sitting<br>5. Quadriceps muscle stretch in standing<br>a. Alternative #1: prone<br>b. Alternative #2: standing lean<br><br>Hold 10-30 seconds; 2-4 reps; at least 2-3x/week | |
| Strength – Trunk | 1. Single arm lift in prone<br>2. Bridging<br>3. Posterior pelvic tilt<br>4. Trunk curl with rotation – arms by side<br>5. Side plank on elbow and knee<br>6. Opposite arm and leg lift in hands and knees<br>7. Double knees to chest with legs bent<br>8. Single leg lowering<br><br>2-3 sets of 8-10 repetitions; 2-3x/week | Patient demonstrates independent performance of the exercise (see Appendix B).<br><br>Patient reports no worsening* of symptoms with exercises. |

| **Treatment Component** | **Phase 2** | **Criteria for patient progression to Phase 3** |
|---|---|---|
| **Patient education** | | |
| General education | Continue patient education as needed | |
| **Exercise Prescription** | | |
| Flexibility – Trunk | 1. Trunk side bend in standing with arms overhead<br>2. Trunk flexion/extension in standing with arm support<br>3. Continue to increase ROM with other exercises described in Phase 1 as appropriate<br><br>Hold 10-30 seconds; 2-4 reps; at least 2-3x/week | Patient demonstrates independent performance of the exercise (see Appendix B).<br><br>Patient reports no worsening* of symptoms. |

© 2020 van Dillen LR et al. *JAMA Neurology*.

| | | |
|---|---|---|
| Flexibility – Extremity | 1. Continue to increase ROM with Phase 1 exercises as appropriate<br><br>Hold 10-30 seconds; 2-4 reps; at least 2-3x/week | Patient demonstrates independent performance of the exercise (see Appendix B).<br><br>Patient reports no worsening* of symptoms. |
| Strength – Trunk | 1. Opposite arm and leg lift in prone<br>2. Bridging with resistance<br>3. Trunk curl – arms crossed<br>4. Trunk curl with rotation – arms crossed<br>5. Side plank on elbow and foot<br>6. Arm and leg lift in face lying with arms bent<br>7. Double knees to chest progression<br>8. Bilateral leg lowering<br><br>2-3 sets of 8-10 repetitions; 2-3x/week | Patient demonstrates independent performance of the exercise (see Appendix B).<br><br>Patient reports no worsening* of symptoms with exercises. |

| **Treatment Component** | **Phase 3** | **Criteria for patient to terminate exercise** |
|---|---|---|
| **Patient education** | | |
| General education | Continue patient education as needed | |
| **Exercise Prescription** | | |
| Flexibility – Trunk | 1. Continue to increase ROM with Phase 1 or Phase 2 exercises as appropriate<br><br>Hold 10-30 seconds; 2-4 reps; at least 2-3x/week | Patient demonstrates independent performance of the exercise (see Appendix B).<br><br>Patient reports no worsening* of symptoms. |
| Flexibility – Extremity | 1. Continue to increase ROM with Phase 1 exercises as appropriate<br><br>Hold 10-30 seconds; 2-4 reps; at least 2-3x/week | Patient demonstrates independent performance of the exercise (see Appendix B).<br><br>Patient reports no worsening* of symptoms. |

© 2020 van Dillen LR et al. *JAMA Neurology*.

| | | |
|---|---|---|
| Strength – Trunk | 1. Active trunk extension in prone<br>2. Bridging with increased resistance<br>3. Trunk curl – hands next to head<br>4. Trunk curl with rotation – hands next to head<br>5. Side plank on hand and foot<br>6. Arm and leg lift in face lying with arms straight<br>7. Pelvic/leg lift<br>8. Bilateral leg raising<br><br>2-3 sets of 8-10 repetitions; 2-3x/week | Patient demonstrates independent performance of the exercise (see Appendix B).<br><br>Patient reports no worsening* of symptoms with exercises. |

**Suggestions for modifications**

1. Lower back flexion (double knee to chest in supine)
    a. Possible modifications:
        i. If participant reports knee pain, he may place his hands behind the posterior thigh.
        ii. If the participant's symptoms worsen as operationally defined, he may limit hip flexion range of motion.
2. Trunk rotation in supine hook lying
    a. Possible modifications:
        i. If the participant's symptoms worsen as operationally defined, he may limit rotation range of motion.
3. Trunk side bending in standing
    a. Possible modifications:
        i. If the participant has difficulty with balance, he may place his hand upon a stable surface for safety.
        ii. If the participant's symptoms worsen as operationally defined, he may limit the amount of side bending range of motion.
4. Trunk flexion/extension in quadruped
    a. Possible modifications:
        i. If participant reports wrist discomfort, he may place his hands in a fist position to keep the wrist neutral.
        ii. If the participant reports knee pain, pillows may be placed under the knees.
        iii. If the participant's symptoms worsen as operationally defined, he may limit the amount of motion.
5. Trunk extension in prone (prone on elbows)
    a. Possible modifications:
        i. If the participant's symptoms worsen as operationally defined, he may lie in prone rather than prone on elbows.
6. Hamstring/calf stretch (supine 90/90 position)
    a. Possible modifications:

© 2020 van Dillen LR et al. *JAMA Neurology*.

            i. If the participant is unable to maintain the exercise position due to quadriceps endurance or upper extremity problems, he could perform one of the following:
                1. Use a doorway to support the lower extremity. See Hamstring/calf muscle stretch alternative #1.
                2. Sit with leg supported on floor and lean trunk forward. See Hamstring/calf muscle stretch alternative #2.

7. Calf stretch (standing position with knee extended)
    a. Possible modifications:
        i. If the participant has lower extremity pain that prevents him from maintaining this position, he can perform the stretch in long sitting using a towel. See Calf muscle stretch alternative.
8. Gluteal stretch (single knee to chest)
    a. Possible modifications:
        i. If the participant reports knee pain, he may place his hands behind the posterior thigh.
        ii. If the participant's symptoms worsen as operationally defined, he may:
            1. limit the amount of hip flexion.
            2. perform the exercise in a seated position. See Gluteal muscle stretch alternative.
9. Piriformis stretch (supine hip lateral rotation)
    a. Possible modifications:
        i. If the participant is unable to perform or if his symptoms worsen as operationally defined, he may:
            1. limit the amount of hip flexion.
            2. perform the exercise in a seated position. See Piriformis muscle stretch alternative.
10. Quadriceps stretch (knee flexion in standing)
    a. Possible modifications:
        i. If the participant has difficulty with balance, he may perform this stretch in prone. See Quadriceps muscle stretch alternative #1.
        ii. If the participant lacks sufficient knee flexion or is unable reach the foot with his hand, he can use a chair/bench/stool for assistance. See Quadriceps muscle stretch alternative #2.
11. Single arm lift in prone
    a. Possible modifications:
        i. If the participant demonstrates a limitation in shoulder flexion range of motion, a pillow may be placed under his chest.
        ii. If the participant's symptoms worsen as operationally defined, he may limit the amount of arm movement.
12. Bridging
    a. Possible modifications:
        i. If the participant's symptoms worsen as operationally defined, he may limit the amount of hip extension.
13. Posterior pelvic tilt
    a. Possible modifications:
        i. If the participants symptoms worsen as operationally defined, he may:
            1. increase the amount of hip and knee flexion.
            2. not flatten his back as much.
14. Trunk curl with rotation, arms by side
    a. Possible modifications:

© 2020 van Dillen LR et al. *JAMA Neurology*.

i. If the participant's symptoms worsen as operationally defined, he may limit the motion.

© 2020 van Dillen LR et al. *JAMA Neurology*.

Educational Principles Quiz Motor Skill Training Condition

**KEY**

(answers **bolded**)

Please circle the **one best answer** for each question.

1. The movements and alignments in my low back that contribute to my low back pain are:
   **This answer will depend on the participant's LBP subgrouping**
   a. Twisting (rotation, side bending, or shifting)
   b. Bending (flexion)
   c. Arching (extension)
   d. Bending (flexion) **<u>and</u>** twisting (rotation, side bending, or shifting)
   e. Arching (extension) **<u>and</u>** twisting (rotation, side bending, or shifting)

2. One of the reasons my LBP continues is because I tend to **repeat** these specific movements and alignments across my day during many of my daily activities.
   **a. True**
   b. False

3. The repetition of movements and positions of my low back in **the same direction** across my day may contribute to keeping my low back region **irritated**.
   **a. True**
   b. False

4. My low back pain symptoms should **improve** if I **modify/change** how I move and align my low back with my daily activities.
   **a. True**
   b. False

5. My treatment is focused on learning how to change the movements and postures performed during my daily activities so that I don't move in the **specific directions** that cause my low back pain symptoms. This should increase my independence in performance of daily activities that have been restricted due to my low back pain.
   **a. True**
   b. False

© 2020 van Dillen LR et al. *JAMA Neurology*.

Educational Concepts Quiz Strength and Flexibility Exercise Condition

**KEY**

(answers **bolded**)

1. Low back pain symptoms can be the result of several **contributors** that include: (Please circle **all** that apply)
   a. **Poor posture**
   b. **Stressful living and working habits**
   c. **Loss of flexibility**
   d. **Loss of strength**
   e. **Decline of general fitness**

2. Exercise and maintaining my activity level are both important for coping with my low back pain symptoms. (Please circle the **best** answer)
   a. **True**
   b. False

3. Exercise is beneficial because it: (Please circle **all** that apply)
   a. **Makes my bones stronger**
   b. **Helps develop fit muscles**
   c. **Keeps me flexible**
   d. **Makes me feel good**
   e. **Contributes to release of natural chemicals that reduce symptoms**

4. **Muscle soreness** after exercise is expected with the performance of new exercises. (Please circle the **best** answer)
   a. **True**
   b. False

5. Performance of strength and flexibility exercises will improve my ability to perform my daily functional activities that are limited due to my LBP symptoms. (Please circle the **best** answer)
   a. **True**
   b. False

© 2020 van Dillen LR et al. *JAMA Neurology*.

**Motor Skill Training Skill Handouts**

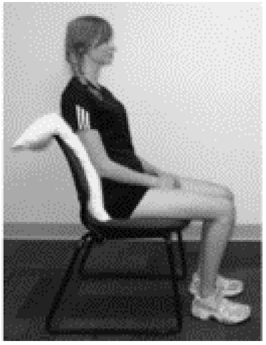
Correct

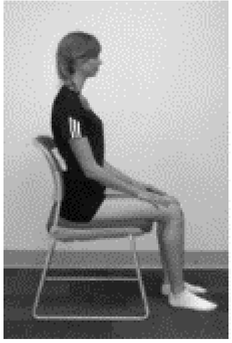
Correct

**Lumbar Rotation Skill: Sitting position – correct**

**Do:**

- Keep back well supported without any bending, twisting, or shifting
- A pillow or small folded towel may be placed behind low back to keep the curve in your low back (use whatever your therapist recommends)
- Keep feet supported on the floor (if feet do not reach the floor, place something underneath feet to support them)

**Key Principle:** Do not bend, twist, or shift low back

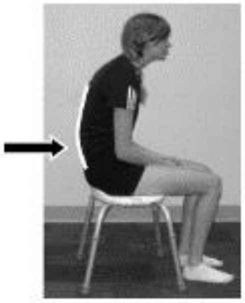
Incorrect – bending in low back

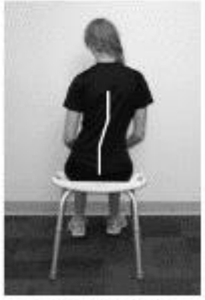
Incorrect – shift in low back

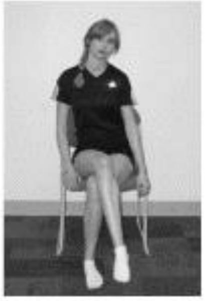
Incorrect – legs crossed

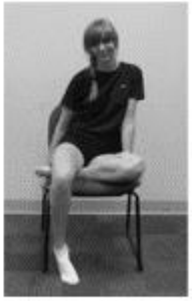
Incorrect – sitting on one leg

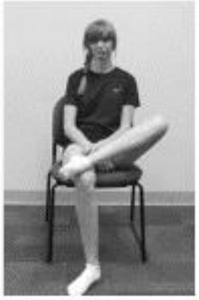
Incorrect – resting one ankle on the opposite knee

**Lumbar Rotation Skill: Sitting position – incorrect**

**Do NOT:**

- Sit with low back bent, twisted, or shifted
- Cross legs
- Sit on one leg

Rest one ankle on the opposite knee

© 2020 van Dillen LR et al. *JAMA Neurology*.

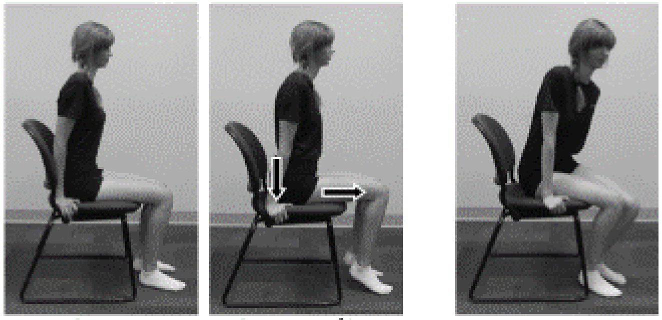

Correct — Correct – pushing up to move forward in chair — Incorrect – twisting in low back/hips

**Lumbar Rotation Skill: Sit to stand – step 1**

**Do:**

- If seated at a desk or another surface, move chair back so there is room to move forward and stand up
- Move forward in chair by pushing on the chair or armrests with both hands (left and middle pictures)

**Do NOT:** Twist low back/hips from side to side to move to edge of chair (right picture)
**Key Principle:** Do not twist low back/hips

---

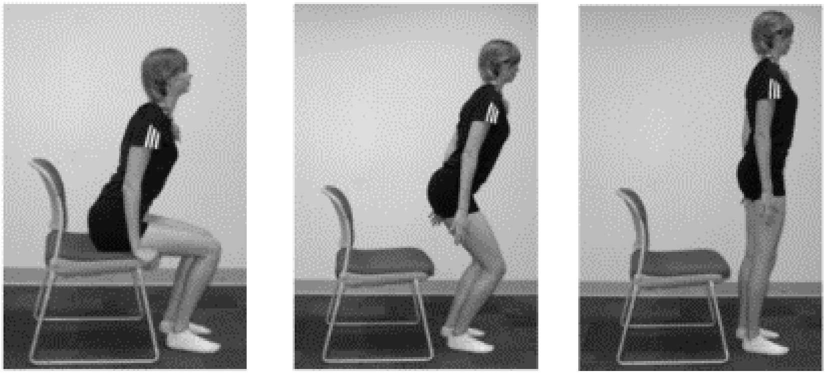

**Lumbar Rotation Skill: Sit to stand – step 2**

**Do:**

- Once at the edge of the chair, place your feet behind your knees (left picture)
- Lean forward at hips keeping back straight as you stand up from the chair (middle and right pictures)

**Do NOT:** Bend or twist your low back as you come to standing
**Key Principle:** Do not bend or twist low back

---

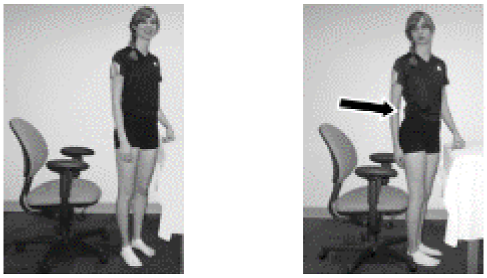

Correct — Incorrect – turning trunk with feet fixed; note twisting in low back

© 2020 van Dillen LR et al. *JAMA Neurology*.

**Lumbar Rotation Skill: Turning**

**Do:** Once in standing, turn away from the surface by stepping with each foot (left picture)
**Do NOT:** Turn your trunk with your feet fixed (right picture)
**Key Principle:** Do not twist low back

---

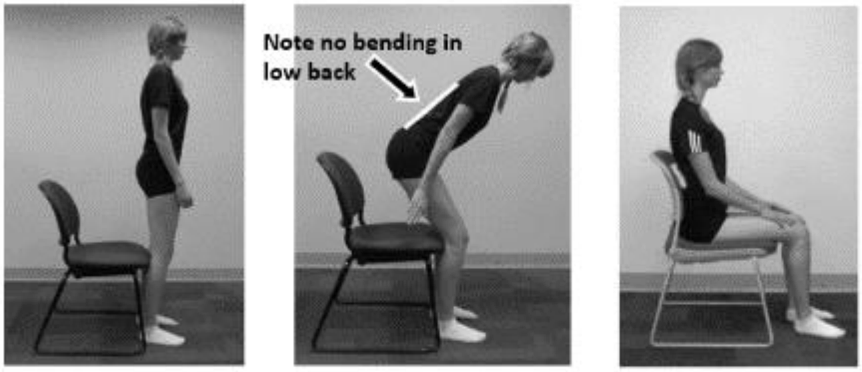


**Lumbar Rotation Skill: Stand to sit**

**Do:**

- Position chair so you are able to walk up to it and turn all the way around so the back of your legs are against the chair (left picture)
- Bend hips and knees to lower yourself into the chair without bending low back (middle picture)
- Move back in the chair and adjust position as necessary (right picture)

**Do NOT:** Bend or twist your low back as you sit down
**Key Principle:** Do not bend or twist low back

---

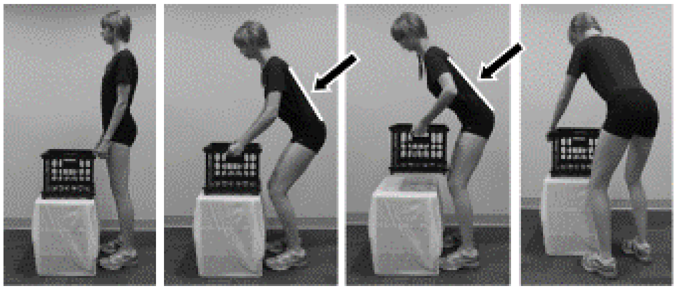

Correct distance | Correct positioning | Correct lifting | Correct positioning with item off to the side (no twisting in low back)

**Lumbar Rotation Skill: Picking up an object – correct**

**Do:**

- Stand close to object with feet shoulder width apart and the object directly in front of you (left picture)
- Keeping back straight, bend hips and knees (middle left picture)
- Contract abdominals
- Pick up object and straighten your legs to lift, keeping object close to your body (middle right picture)
- If an object is located off to the side, position yourself directly behind it (right picture)

**Key Principle:** Bend in hips and knees, not in low back

---

© 2020 van Dillen LR et al. *JAMA Neurology*.

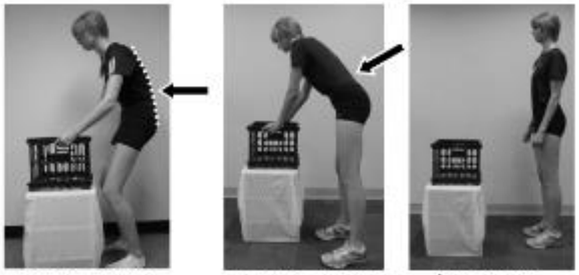

Incorrect – bending in low back | Incorrect – twisting in low back (feet facing forward, shoulders facing off to side) | Incorrect – too far from object

**Lumbar Rotation Skill: Picking up an object – incorrect**

**Do NOT:**

- Bend, twist, or shift your low back (left picture)
- Reach for an object off to the side without moving your whole body over to the object, as this would result in twisting of low back (middle picture)
- Stand too far away from the object (right picture)

---

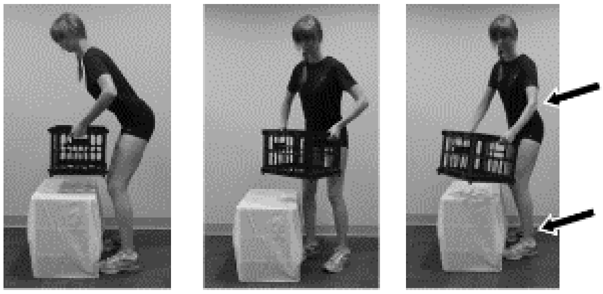

Correct | Correct | Incorrect – turning trunk with feet fixed; note twisting in low back

**Lumbar Rotation Skill: Placing object in same or different location**

**Do:**

- Keep object close to body as you lift
- Place object back in the same location by bending hips and knees (left picture)
- If object needs to be placed in a different location, move whole body by stepping with each foot (middle picture)

**Do NOT:** Turn your trunk while keeping your feet fixed (right picture)

**Key Principle:** Bend in hips and knees, not in low back; do not twist low back

---

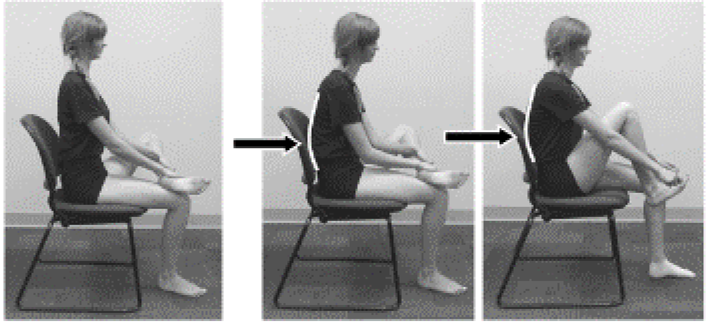

Correct | Incorrect – note bending in low back | Incorrect – note bending in low back

**Lumbar Rotation Skill: Donning a sock – initiation phase**

**Do:**

- Bend hip and knee to lift foot up off floor

© 2020 van Dillen LR et al. *JAMA Neurology*.

- Place foot on surface or rest it on opposite knee (left picture)

**Do NOT:** Bend or twist your low back (middle and right pictures)
**Key Principle:** Do not bend or twist low back

---

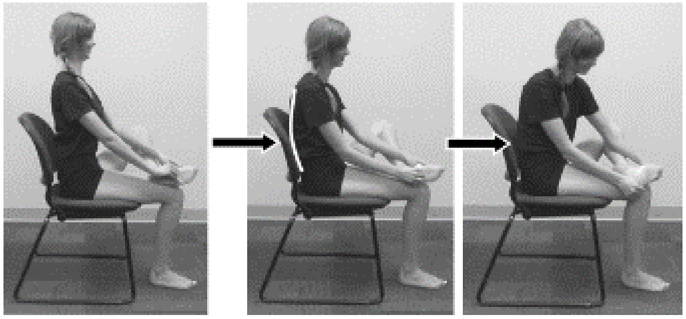

Correct | Incorrect – note bending in low back | Incorrect – note twisting in low back

**Lumbar Rotation Skill: Donning a sock – completion phase**

**Do:**

- Put sock on foot, keeping low back straight (left picture)
- Contract abdominals and lower foot back to the floor

**Do NOT:** Bend or twist your low back, especially when leaning forward to put the sock on your foot (middle and right pictures)
**Key Principle:** Do not bend or twist low back

---

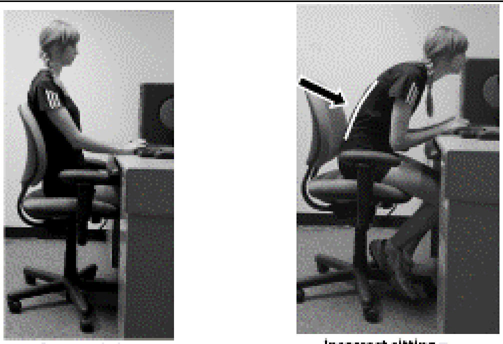

Correct sitting | Incorrect sitting – bending in low back

**Lumbar Rotation Skill: Sitting position at work surface**

**Do:**

- Sit comfortably in a chair with appropriate positioning materials
- Move chair close to work surface (left picture)
    - Keep head and shoulders over hips
    - Keep back supported

**Do NOT:** Sit away from the surface so you are leaning forward (right picture)
**Key Principle:** Do not bend low back

---

**Primary work zone**: The area in which you can work when performing a sweeping motion with your arm when your elbow is bent to 90 degrees.
**Secondary work zone**: The area in which you can work when performing a sweeping motion with your arm when it is fully extended.
**Tertiary work zone**: The area that is outside of your reach when your arm is fully extended.

**Lumbar Rotation Skill: Sitting position – work zones**

**Do:**

© 2020 van Dillen LR et al. *JAMA Neurology*.

- Position items you will use repetitively within the **primary** work zone
- Position items you will use occasionally within the **secondary** work zone
- Position items you rarely use within the **tertiary** work zone
- When reaching for items in the tertiary work zone, lean forward by bending at hips, keeping back straight

**Do NOT:**

- Bend, twist, or shift your low back when reaching for items
- Keep items you use frequently too far away from you

**Key Principle:** Keep items you use often close to you

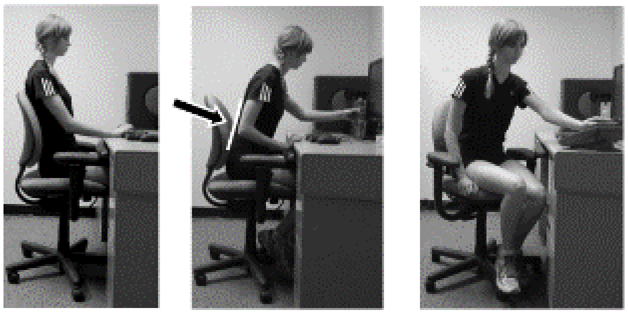

Correct sitting | Correct reaching – bending in hips and not low back | Correct reaching – turning whole body

**Lumbar Rotation Skill: Manipulate/reach for objects on work surface while sitting – correct**

**Do:**

- Sit comfortably in a chair with appropriate positioning materials
- Keep items you will be using often (e.g. keyboard, mouse, etc.) within the **primary** work zone (left picture)
- When reaching for an object on work surface, lean forward by bending at hips (middle picture)
- If you need to reach for something off to the side, turn whole body to face object (right picture)

**Key Principle:** Do not bend or twist low back; bend in hips when reaching

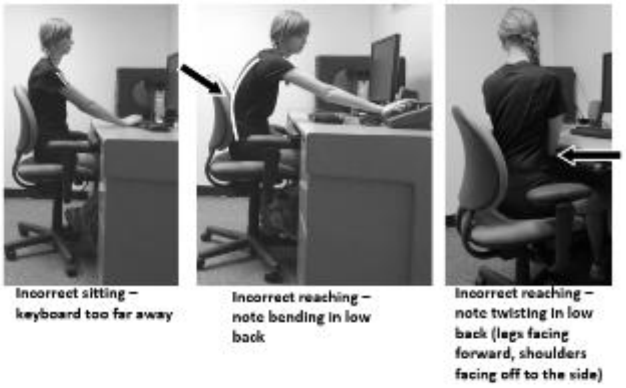

Incorrect sitting – keyboard too far away | Incorrect reaching – note bending in low back | Incorrect reaching – note twisting in low back (legs facing forward, shoulders facing off to the side)

**Lumbar Rotation Skill: Manipulate/reach for objects on work surface while sitting – incorrect**

**Do NOT:**

- Keep items you use frequently far away from you
- Bend, twist, or shift in low back when reaching for an object

© 2020 van Dillen LR et al. *JAMA Neurology*.

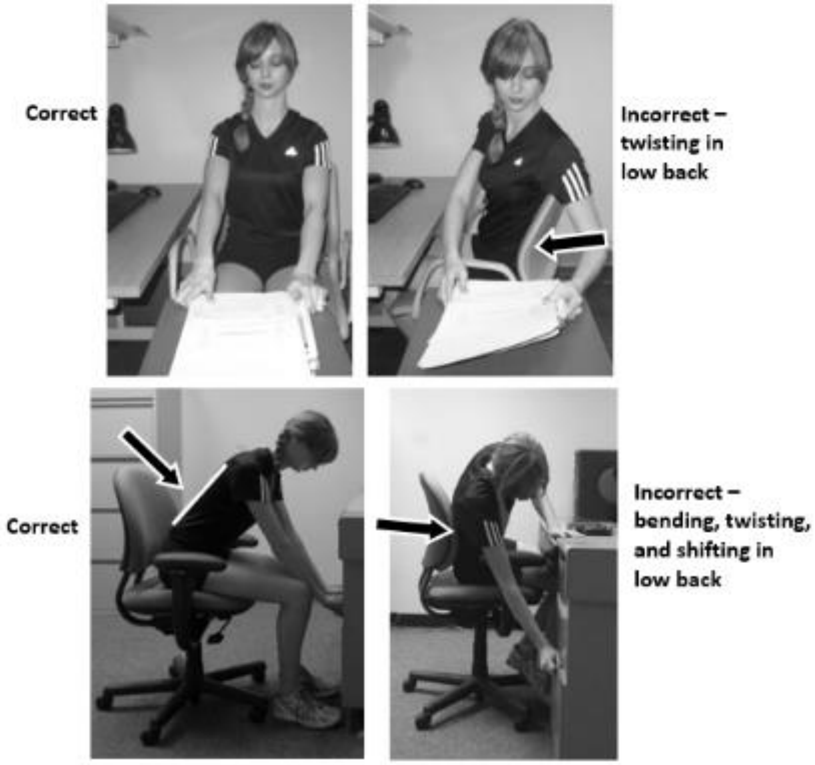


**Lumbar Rotation Skill: Manipulate/reach for objects outside of work surface while sitting**

**Do:**

- Sit comfortably in a chair with appropriate positioning materials
- When reaching for something lower than work surface, turn chair or move chair to reach for the object (left pictures)
    - If chair does not swivel, turn whole body

**Do NOT:** Bend, twist, or shift in your low back to reach for an object (right pictures)

**Key Principle:** Do not bend, twist, or shift low back

---

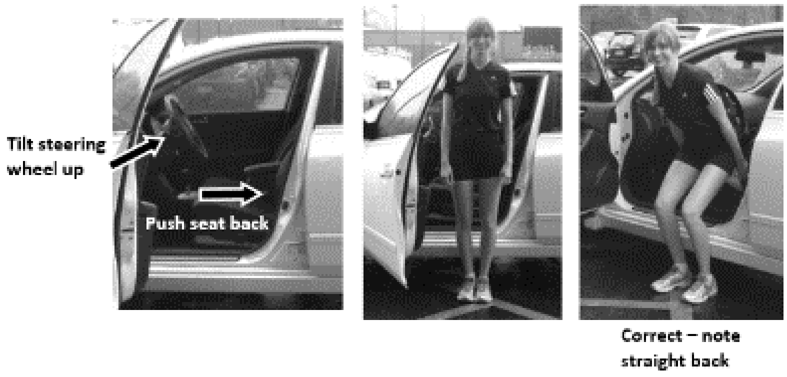


**Lumbar Rotation Skill: Getting into car – step 1**

**Do:**

- Open door, adjust steering wheel and seat position as necessary (left picture)
- Stand facing away from car (middle picture)
- Lower yourself down to sit on the edge of the seat, keeping back straight as you sit down (right picture)
- Scoot back into the car, keeping feet supported on the ground

**Do NOT:** Bend, twist, or shift your low back

**Key Principle:** Bend in hips and knees; do not bend, twist, or shift low back

---

© 2020 van Dillen LR et al. *JAMA Neurology*.

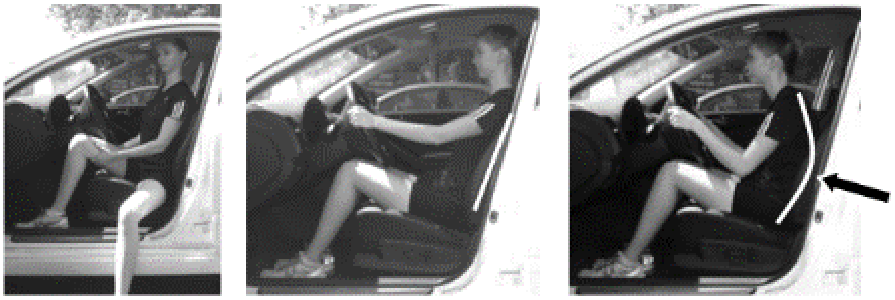

Correct

Incorrect – note bending in low back

**Lumbar Rotation Skill: Getting into car – step 2**

**Do:**

- Lift each leg into car, one at a time (left picture)
    - Use hands to help lift legs, if necessary
- Turn whole body to face forward in car
- Sit comfortably in seat using appropriate positioning materials
- Adjust position of seat and/or steering wheel so back is straight (middle picture)

**Do NOT:** Lean forward or to one side, resulting in bending, twisting, or shifting of your low back (right picture)

**Key Principle:** Do not bend, twist, or shift low back

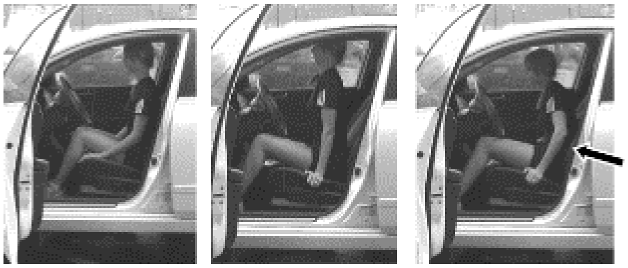

Wheel tilted up, seat pushed back

Correct – move to side by pushing up with both hands

Incorrect – move to side by scooting buttocks over; note twisting in low back (shoulders not aligned over hips)

**Lumbar Rotation Skill: Getting out of car – step 1**

**Do:**

- Adjust steering wheel and seat position as necessary (left picture)
- Move to side of seat while still facing forward in car
    - Move closer to side of seat by pushing on the seat with both hands equally (middle picture)

**Do NOT:** Bend, twist, or shift your low back, especially when moving to the side of the seat (right picture)

**Key Principle:** Do not bend, twist, or shift low back

© 2020 van Dillen LR et al. *JAMA Neurology*.

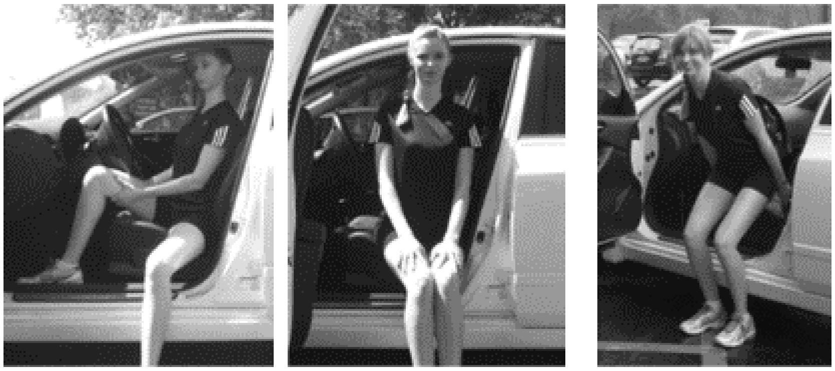

**Lumbar Rotation Skill: Getting out of car – step 2**
**Do:**

- Contract abdominal muscles as you lift each leg out of car, one at a time
    - Use hands to help lift legs, if necessary (left picture)
- Sit on the edge of the seat with feet on ground (middle picture)
- Lean forward by bending at hips and stand up, keeping back straight (right picture)

**Do NOT:** Bend, twist, or shift your low back
**Key Principle:** Do not bend, twist, or shift low back

---

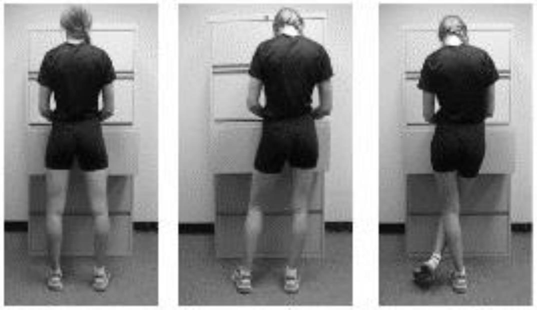

Correct standing | Incorrect standing – weight shifted onto one foot | Incorrect standing – legs crossed

**Lumbar Rotation Skill: Standing position**
**Do:**

- Stand with feet shoulder width apart, keeping weight evenly distributed over both feet (left picture)
- Stand close to the surface on which you will be working
- Surface should be at the height of your elbow for most tasks/activities

**Do NOT:**

- Stand with weight shifted onto one foot (middle picture)
- Stand with feet/legs crossed (right picture)
- Hold a load in one hand or on one side on a regular basis or for an extended period of time

**Key Principle:** Do not twist or shift low back

---

**Primary work zone**: The area in which you can work when performing a sweeping motion with your arm when your elbow is bent to 90 degrees.
**Secondary work zone**: The area in which you can work when performing a sweeping motion with your arm when it is fully extended.
**Tertiary work zone**: The area that is outside of your reach when your arm is fully extended.

**Skill: Standing position – work zones**

© 2020 van Dillen LR et al. *JAMA Neurology*.

**Do (see figure above):**

- Position items you will use repetitively within the **primary** work zone
- Position items you will use occasionally within the **secondary** work zone
- Position items you rarely use within the **tertiary** work zone
- When reaching for items in the tertiary work zone, lean forward by bending at hips, keeping back straight

**Do NOT:**

- Bend, twist, or shift your low back when reaching for items
- Keep items you use frequently too far away from you

**Key Principle:** Keep items you use often close to you

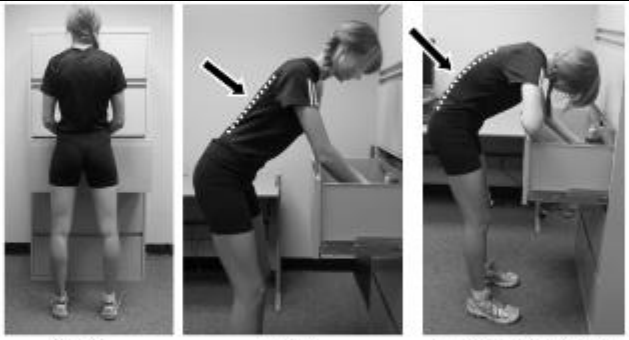

Correct standing | Correct reaching | Incorrect reaching – note bending in low back

**Lumbar Rotation Skill: Activities while standing in one place**

**Do:**

- Stand comfortably with the appropriate positioning materials (left picture)
- Keep items used often within the **primary** work zone
- When reaching for an object on the work surface, lean forward by bending at hips, keeping back straight (middle picture)
- If activity requires strength, surface should be slightly lower than elbow height
- If activity requires precision, surface should be slightly higher than elbow height

**Do NOT:** Bend, twist, or shift your low back when reaching for an object (right picture)
**Key Principle:** Bend in hips and knees; do not bend, twist, or shift low back

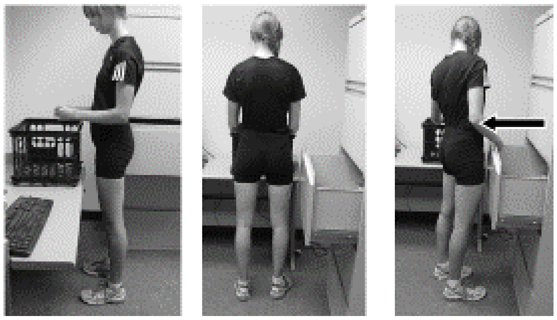

Correct reaching | Correct reaching | Incorrect reaching – note twisting in low back

**Lumbar Rotation Skill: Activities that require standing and walking**

**Do:**

- Stand comfortably with the appropriate positioning materials
- When reaching for something not directly in front of you, turn whole body to face the object (left and middle pictures)
    - Alternatively, you could side step so the object is directly in front of you

© 2020 van Dillen LR et al. *JAMA Neurology*.

- If you need to walk to another location, step away from surface, turn whole body in the direction you need to go, and then walk

**Do NOT:** Twist or shift in your low back when reaching for an object (right picture)
**Key Principle:** Do not twist or shift low back

---

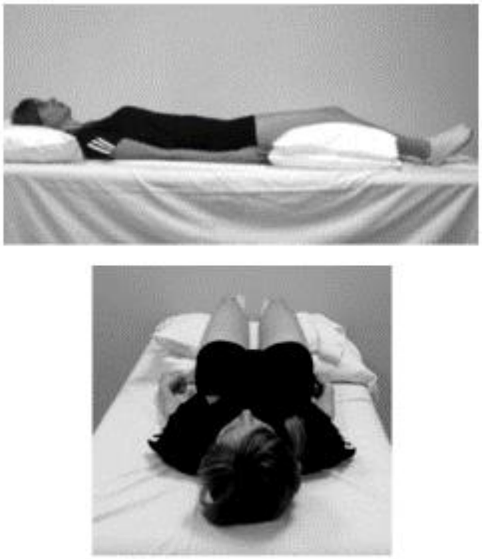

**Lumbar Rotation Skill: Positioning – back lying**
**Do:**

- Place pillows under knees to relieve pressure on low back
- Keep back straight
- Practice log rolling (i.e. moving body as a unit) before going to sleep

**Key Principle:** Do not lie with low back bent, twisted, or shifted

---

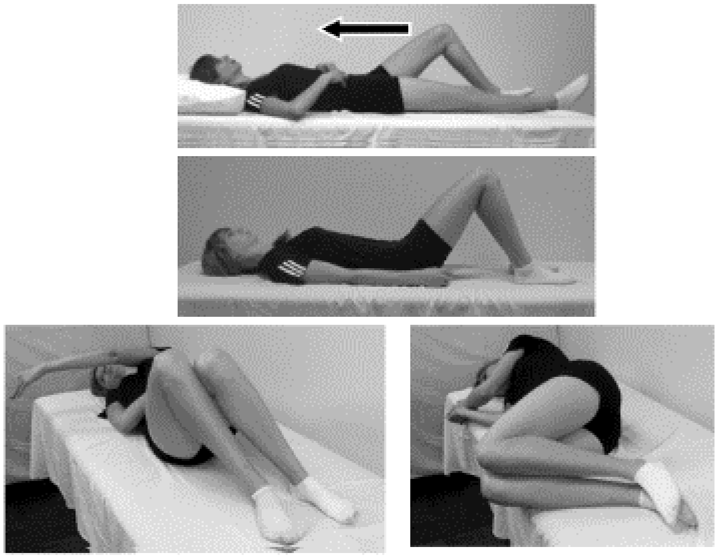

**Lumbar Rotation Skill: Rolling back lying to side lying**
**Do:**

- Remove covers and any positioning materials
- Contract abdominals and slide each heel up one at a time (top picture) so hips and knees are bent (middle picture)
- Reach with arm and push feet into surface to log roll onto side (bottom pictures)

© 2020 van Dillen LR et al. *JAMA Neurology*.

- Lead with arms (arms before legs; A before L), moving body as a unit

**Do NOT:** Twist upper body independently of lower body
**Key Principle:** Move body as a unit

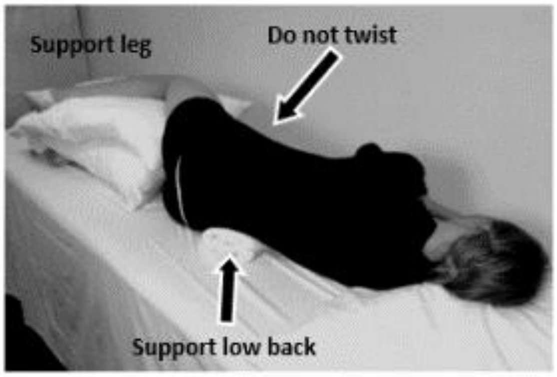


**Lumbar Rotation Skill: Positioning – side lying**
**Do:**
- Place as many pillows between knees as needed to keep pelvis in good alignment (i.e. no twisting or tilting)
- Legs should be aligned on top of each other
- Place a small folded towel at waist, just above hip
- Use a pillow to support head in a neutral position (i.e. no bending or twisting)

**Do NOT:**
- Lie with low back arched, twisted, or shifted
- Lie with pelvis twisted or tilted

**Key Principle:** Do not lie with low back bent, twisted, or shifted

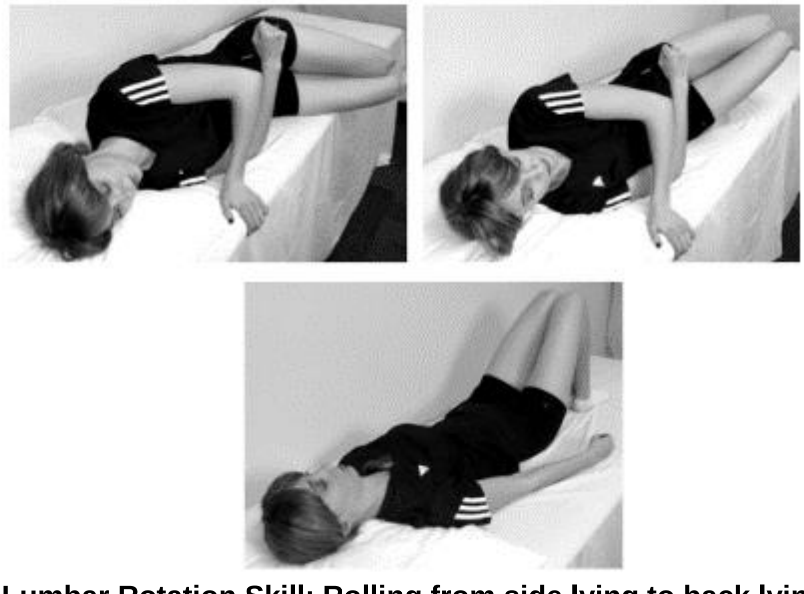

**Lumbar Rotation Skill: Rolling from side lying to back lying**
**Do:**
- Remove covers and any positioning materials
- Push hand of top arm, elbow of bottom arm, and lower leg into surface to log roll onto back, moving body as a unit (top pictures)
- Once on back (bottom picture), contract abdominals and slide one heel down at a time
- Position in back lying using materials as appropriate

© 2020 van Dillen LR et al. *JAMA Neurology*.

**Do NOT:** Twist your upper body independently of your lower body, especially when reaching to push from surface with top arm
**Key Principle:** Move body as a unit

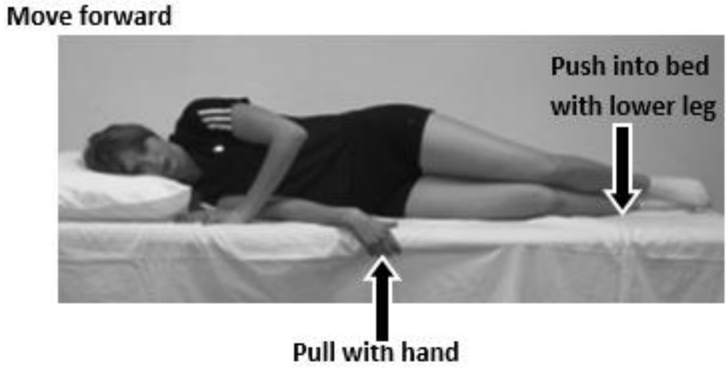


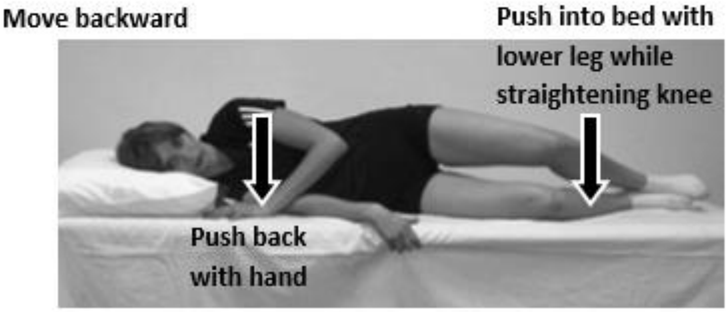


**Lumbar Rotation Skill: Moving in bed – side lying**
**Do:**

- Remove covers and any positioning materials
- To move forward (top picture)
    - Pull on the side of the bed with bottom hand and push into the bed with lower leg
- To move backward (bottom picture)
    - Push back with top hand and side of lower leg while straightening knee

**Do NOT:** Bend, twist, or shift your low back
**Key Principle:** Do not bend, twist, or shift low back

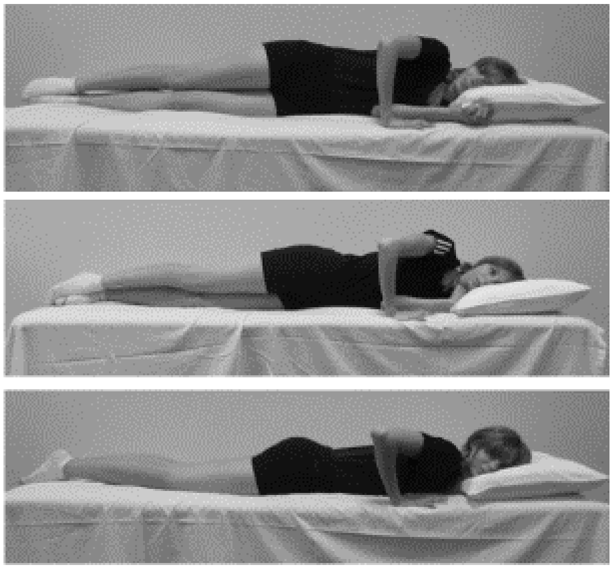

**Lumbar Rotation Skill: Rolling from side lying to face lying**

© 2020 van Dillen LR et al. *JAMA Neurology*.

**Do:**

- Remove covers and any positioning materials
- Straighten legs (top picture)
- Log roll onto stomach, moving body as a unit (middle and bottom pictures)

**Do NOT:** Twist upper body independently of lower body
**Key Principle:** Move body as a unit

---

Correct position

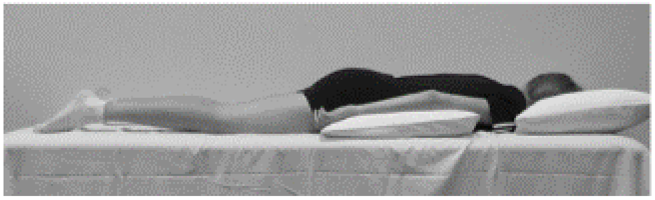

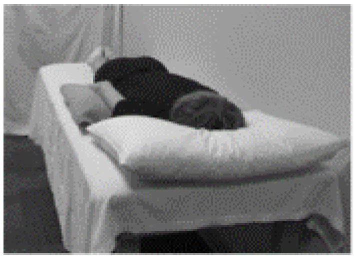

Correct position

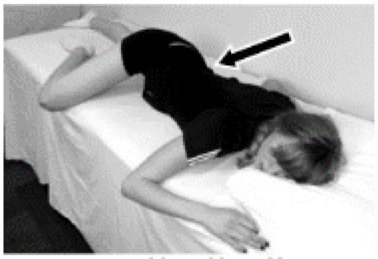

Incorrect position – hip and knee bent; note twisting in low back

**Lumbar Rotation Skill: Positioning – face lying**

**Do:** Use pillows as needed to help prevent twisting or shifting of your low back
**Do NOT:** Lie with one hip and knee bent resulting in twisting in your low back
**Key Principle:** Do not lie with low back twisted or shifted

---

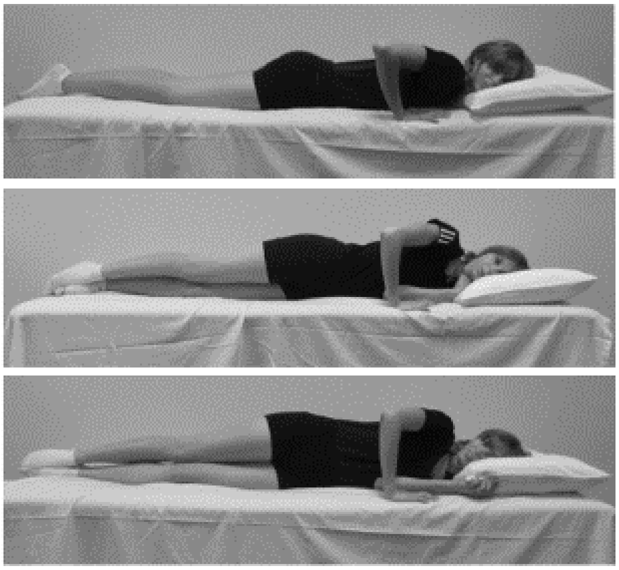

**Lumbar Rotation Skill: Rolling from face lying to side lying**

**Do:**

- Remove covers and any positioning materials
- If rolling onto left side, tuck left arm under body as much as possible
- Bend right arm up so hand is by chest (top picture)

© 2020 van Dillen LR et al. *JAMA Neurology*.

- Contract abdominals, and push right hand into surface to roll onto left side, moving body as a unit (middle and bottom pictures)
- Position in side lying with appropriate materials

**Do NOT:** Twist upper body independently of lower body
**Key Principle:** Move body as a unit

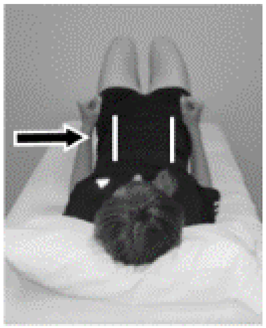
Correct

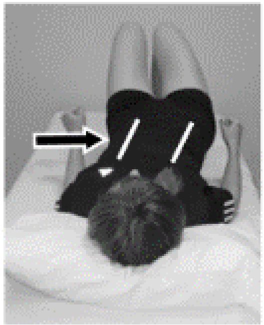
Incorrect – hips not in line with shoulders

**Lumbar Rotation Skill: Moving in bed – back lying**
**Do:**

- Remove covers and any positioning materials
- Contract abdominals and slide each heel up one at a time so hips and knees are bent
- Bend both arms so elbows are bent to 90 degrees
- Push down with elbows and feet at the same time
- Move body to the side in small increments
- Keep hips in line with shoulders, moving trunk as a unit (left picture)

**Do NOT:** Move hips while keeping shoulders fixed (right picture), as this will result in a shift in your low back
**Key Principle:** Move trunk as a unit

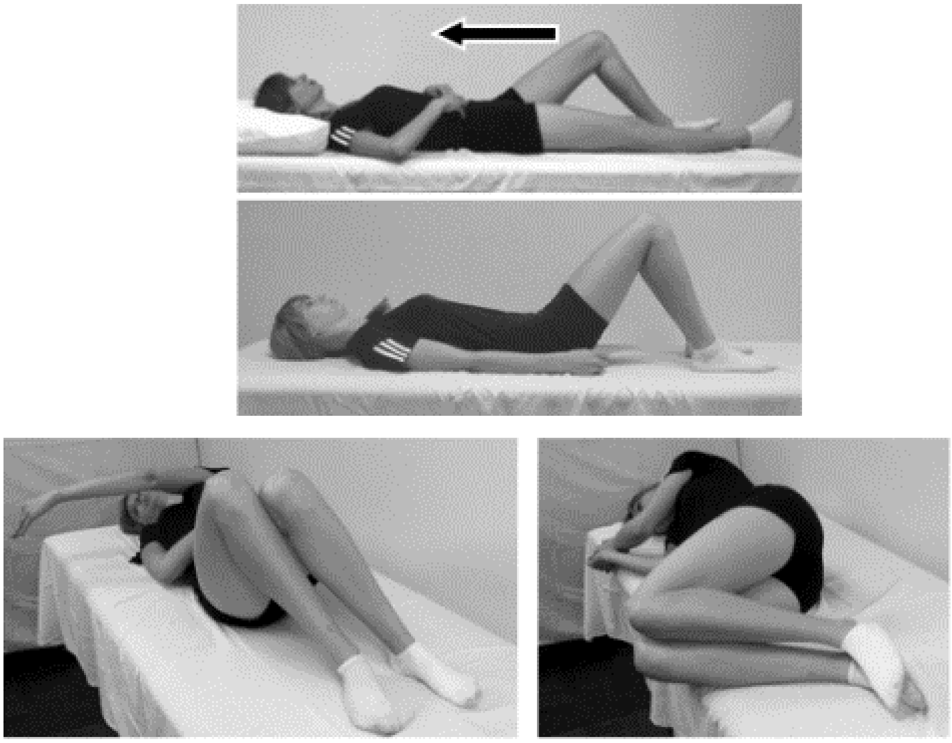

© 2020 van Dillen LR et al. *JAMA Neurology*.

**Lumbar Rotation Skill: Back lying to sit – step 1 (back lying to side lying)**
**Do:**

- Remove covers and any positioning materials
- Contract abdominals and slide each heel up one at a time (top picture) so hips and knees are bent (middle picture)
- Reach with arm and push feet into surface to log roll to side (bottom pictures)
- Lead with arms (arms before legs; A before L), moving body as a unit

**Do NOT:** Twist upper body independently of lower body
**Key Principle:** Move body as a unit

---

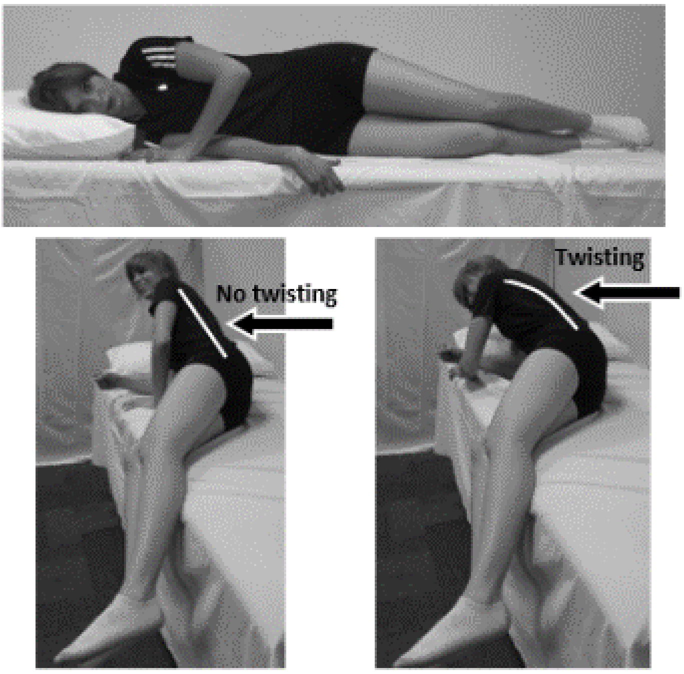


Correct

Incorrect – twisting in low back (shoulders ahead of hips)

**Lumbar Rotation Skill: Back lying to sit – step 2 (side lying to sit; using top arm)**
**Do:**

- From side lying (top picture), push into surface with top arm and elbow of the arm that is on the surface, while letting legs drop over edge of surface (bottom left picture)
- Keep low back straight as you come to sitting
- Move forward to edge of surface so feet are positioned on floor

**Do NOT:** Twist your low back, especially when reaching for the surface and pushing with your top hand (bottom right picture)
**Key Principle:** Move trunk as a unit

---

© 2020 van Dillen LR et al. *JAMA Neurology*.

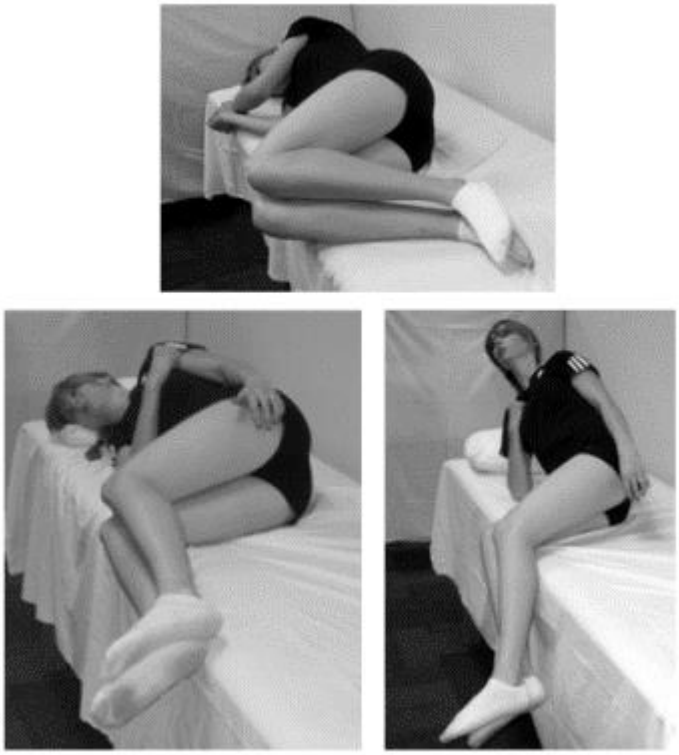

**Lumbar Rotation Skill: Back lying to sit – step 2 (side lying to sit; without using top arm)**

**Do:**

- From side lying (top picture), push into surface with elbow of the arm that is on the surface while letting legs drop over edge of surface (bottom pictures)
- Keep low back straight as you come to sitting
- Move forward to edge of surface so feet are positioned on floor

**Do NOT:**

- Twist low back
- Use top arm to help push up as this will result in twisting in low back

**Key Principle:** Move trunk as a unit

---

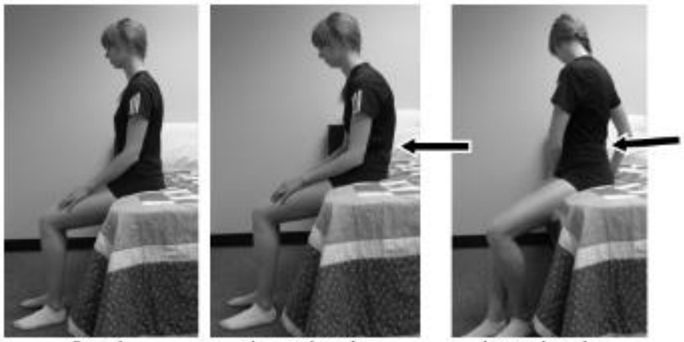

Correct | Incorrect – note bending in low back | Incorrect – note twisting in low back

**Lumbar Rotation Skill: Sitting position on edge of bed**

**Do:**

- Move forward to edge of bed so feet are supported on floor (left picture)
- If feet do not touch ground, place support under them (e.g. step stool), especially if you are going to be sitting on the edge of the bed for an extended period of time

**Do NOT:** Sit with your low back bent, twisted, or shifted (middle and right pictures)

© 2020 van Dillen LR et al. *JAMA Neurology*.

**Key Principle:** Do not bend, twist, or shift low back

Correct position

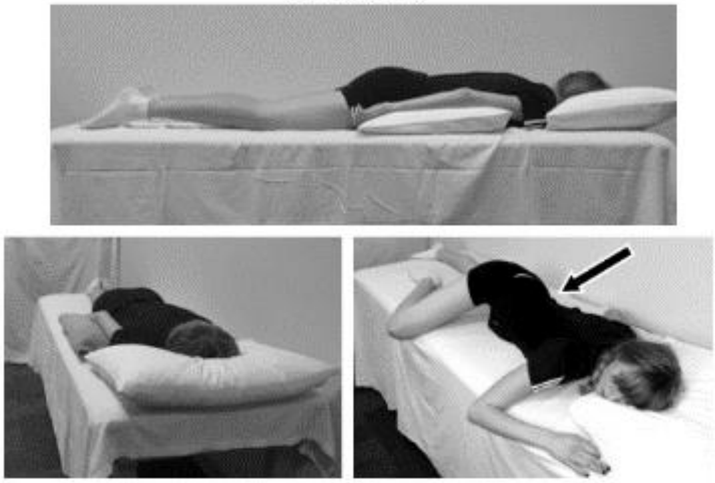

Correct position

Incorrect position – hip and knee bent; note twisting in low back

**Lumbar Rotation Skill: Positioning – face lying**

**Do:** Use pillows as needed to help prevent twisting or shifting of your low back
**Do NOT:** Lie with one hip and knee bent resulting in twisting in your low back
**Key Principle:** Do not lie with low back twisted or shifted

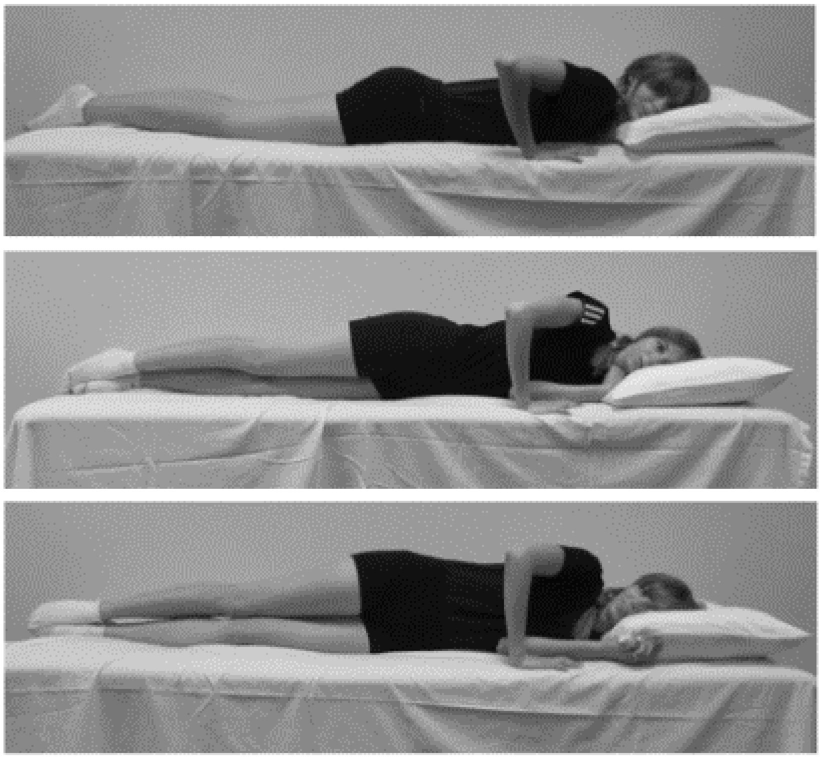

**Lumbar Rotation Skill: Face lying to sit – step 1 (face lying to side lying)**

**Do:**

- Remove covers and any positioning materials
- If rolling onto left side, tuck left arm under body as much as possible
- Bend right arm up so hand is by chest (top picture)

© 2020 van Dillen LR et al. *JAMA Neurology*.

- Contract abdominals and push right hand into surface to roll onto left side, moving body as a unit (middle and bottom pictures)

**Do NOT:** Twist upper body independently of lower body
**Key Principle:** Move body as a unit

---

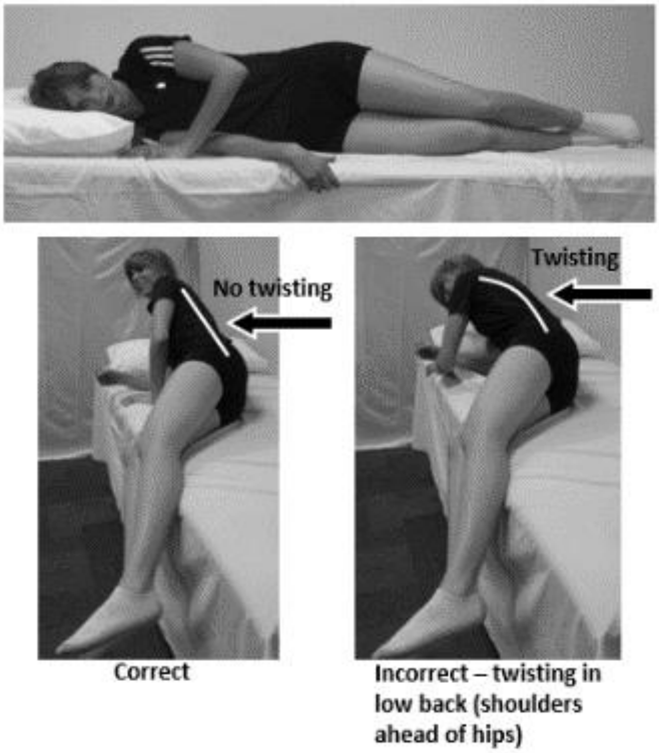


**Lumbar Rotation Skill: Face lying to sit – step 2 (side lying to sit; using top arm)**

Do:

- From side lying (top picture), push into surface with top arm and elbow of the arm on the surface, while letting legs drop over edge of surface (bottom left picture)
- Keep low back straight as you come to sitting
- Move forward to edge of surface so feet are positioned on floor

**Do NOT:** Twist your low back (bottom right picture)
**Key Principle:** Move trunk as a unit

---

© 2020 van Dillen LR et al. *JAMA Neurology*.

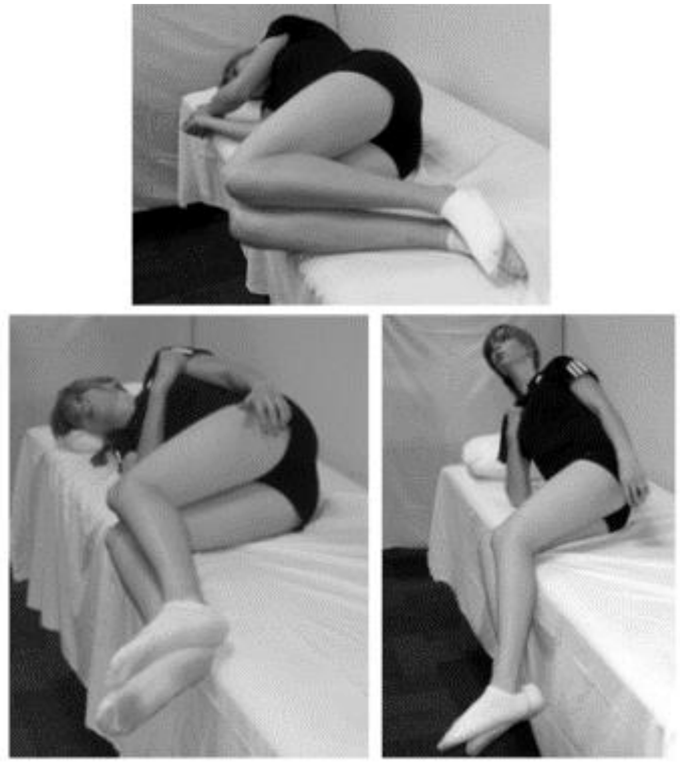

**Lumbar Rotation Skill: Face lying to sit – step 2 (side lying to sit; without using top arm)**

**Do:**

- From side lying (top picture), push into surface with elbow of the arm that is on the surface while letting legs drop over edge of surface (bottom pictures)
- Keep low back straight as you come to sitting
- Move forward to edge of surface so feet are positioned on floor

**Do NOT:**

- Twist low back
- Use top arm to help push as this will result in twisting in low back

**Key Principle:** Move trunk as a unit

---

© 2020 van Dillen LR et al. *JAMA Neurology*.

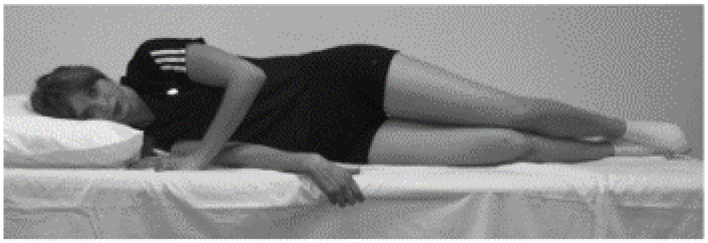

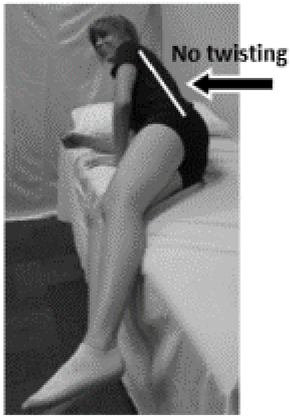


Correct

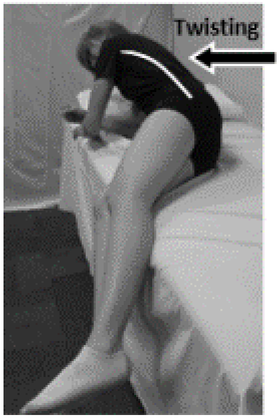


Incorrect – twisting in low back (shoulders ahead of hips)

**Lumbar Rotation Skill: Side lying to sit; using top arm**

**Do:**

- From side lying (top picture), push into surface with top arm and elbow of the arm on the surface, while letting legs drop over edge of surface (bottom left picture)
- Keep low back straight as you come to sitting
- Move forward to edge of surface so feet are positioned on floor

**Do NOT:** Twist your low back (bottom right picture)

**Key Principle:** Move trunk as a unit

---

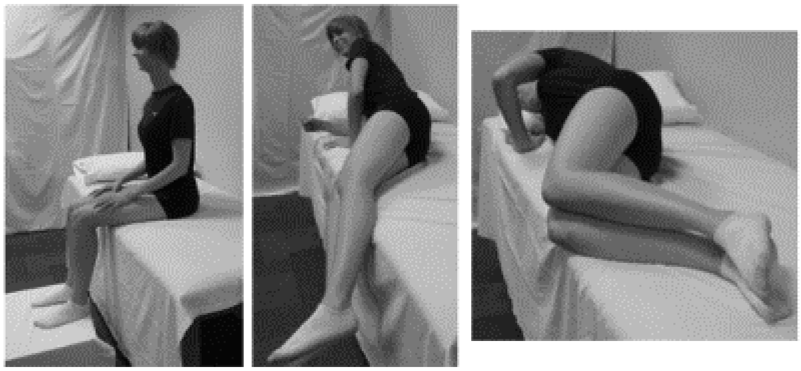

**Lumbar Rotation Skill: Sit to back lying – step 1 (sit to side lying)**

**Do:**

- From sitting (left picture), lower yourself down onto elbow closest to surface (i.e. right arm if going to right side, left arm if going to left side) as you move both feet up onto surface together (middle and right pictures)

**Do NOT:** Twist your low back

**Key Principle:** Move trunk as a unit

---

© 2020 van Dillen LR et al. *JAMA Neurology*.

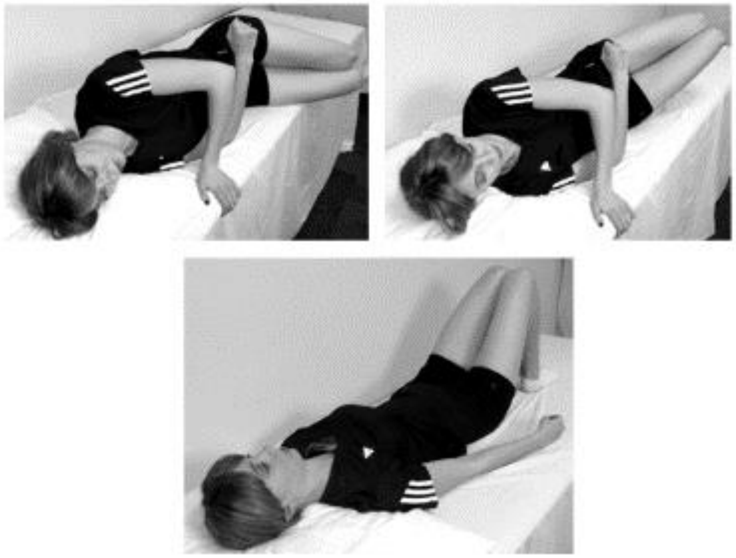

**Lumbar Rotation Skill: Sit to back lying – step 2 (rolling from side lying to back lying)**
**Do:**

- From side lying, push hand of top arm, elbow of bottom arm, and lower leg into surface to log roll onto back, moving body as a unit (top pictures)
- Once on your back (bottom picture), contract abdominals and slide one heel down at a time
- Position on your back using materials as appropriate

**Do NOT:** Twist your upper body independently of your lower body, especially when reaching over with your top arm to push from the surface
**Key Principle:** Move body as a unit

---

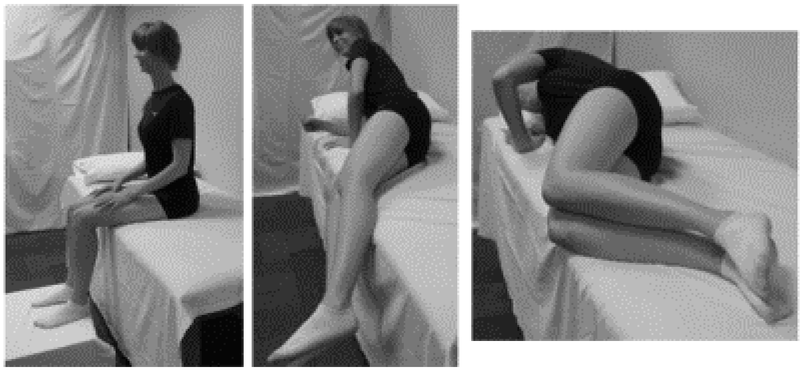

**Lumbar Rotation Skill: Sit to face lying – step 1 (sit to side lying)**
Do:

- From sitting (left picture), lower yourself down onto elbow closest to surface (i.e. right arm if going to right side, left arm if going to left side) as you move both feet up onto surface together (middle and right pictures)

**Do NOT:** Twist your low back
**Key Principle:** Move trunk as a unit

---

© 2020 van Dillen LR et al. *JAMA Neurology*.

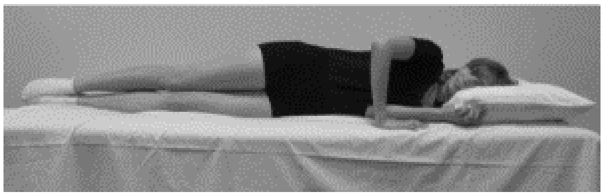
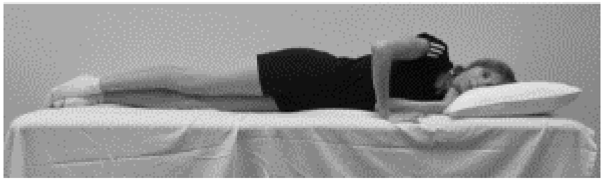
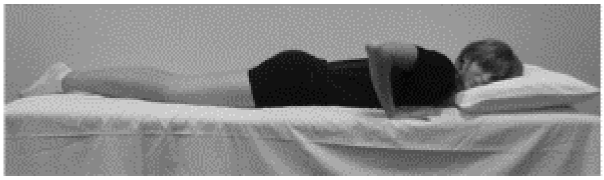

**Lumbar Rotation Skill: Sit to face lying – step 2 (rolling from side lying to face lying)**

**Do:**

- Move yourself back from edge of bed so you have room to roll onto stomach
- Straighten legs
- Log roll onto stomach, moving body as a unit

**Do NOT:** Twist upper body independently of lower body

**Key Principle:** Move body as a unit

---

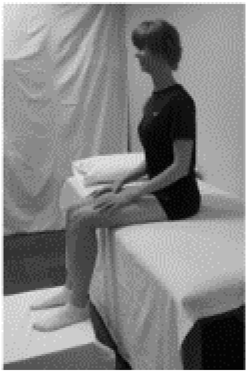
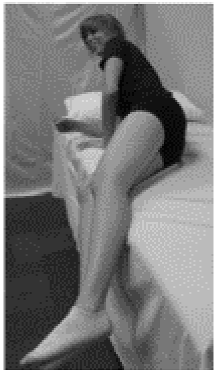
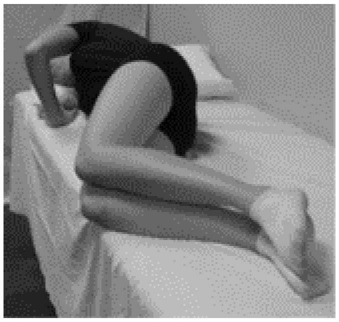

**Lumbar Rotation Skill: Sit to side lying**

**Do:**

- From sitting (left picture), lower yourself down onto elbow closest to surface (i.e. right arm if going to right side, left arm if going to left side) as you move both feet up onto surface together (middle and right pictures)

**Do NOT:** Twist your low back

**Key Principle:** Move trunk as a unit

**Key Principle:** Do not lie with low back bent, twisted, or shifted

---

© 2020 van Dillen LR et al. *JAMA Neurology*.

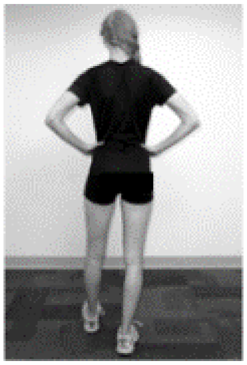

Correct

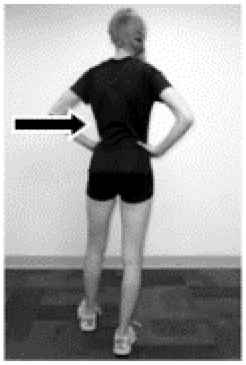

Incorrect – note twisting of pelvis and low back

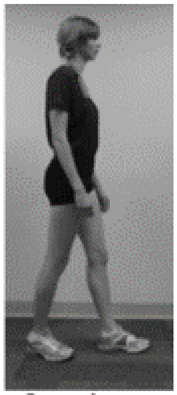

Correct – shorter step length with no twisting of pelvis or low back

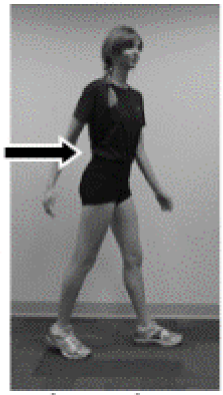

Incorrect – longer step length with twisting of pelvis and low back

**Lumbar Rotation Skill: Walking**

**Do:**

- Contract abdominals to control twisting of pelvis and low back
- Place your hands on your pelvis to monitor twisting or tilting of pelvis
- Take smaller steps or slow your walking speed to help control twisting or tilting if your therapist advised you to do so

**Do NOT:** Twist or tilt your pelvis or low back

**Key Principle:** Do not twist or tilt pelvis or low back

---

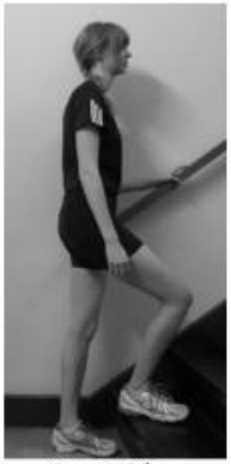

Correct stair climbing

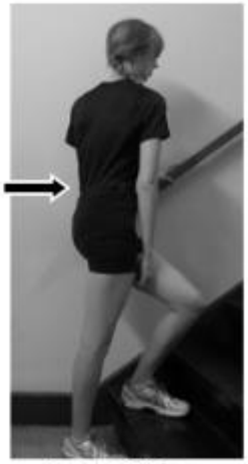

Incorrect stair climbing – note twisting in pelvis/ low back

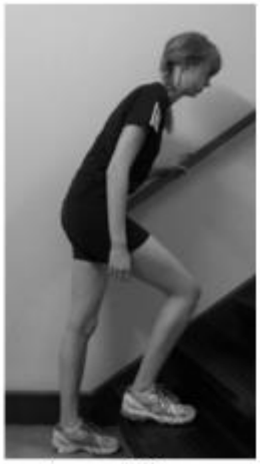

Incorrect stair climbing – note leaning forward

**Lumbar Rotation Skill: Stair climbing – upstairs**

**Do:**

- Contract abdominals when lifting leg onto next step
- You may monitor for pelvic twisting or tilting by placing hands on pelvis
- You may use hand rail as needed for balance or to help decrease the load on your trunk (left picture)

**Do NOT:**

- Twist or tilt pelvis/low back (middle picture)

© 2020 van Dillen LR et al. *JAMA Neurology*.

- Lean forward, as this results in bending in your back (right picture)

**Key Principle:** Do not twist or tilt pelvis/low back

---

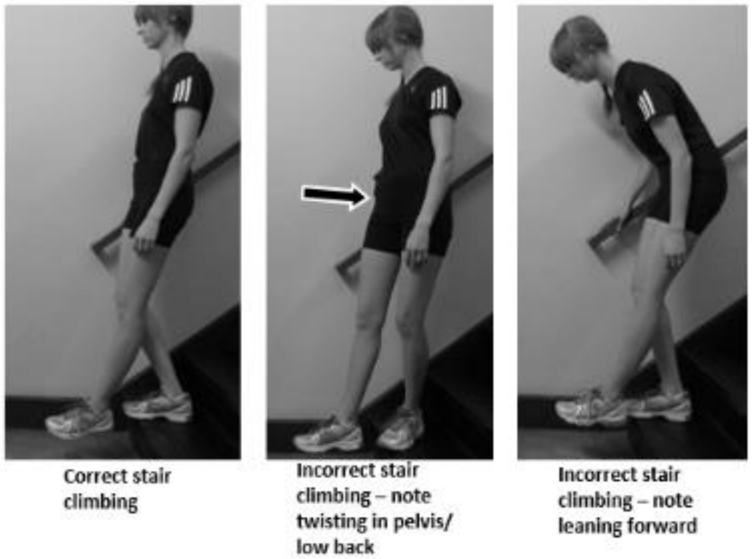

Correct stair climbing

Incorrect stair climbing – note twisting in pelvis/ low back

Incorrect stair climbing – note leaning forward

**Lumbar Rotation Skill: Stair climbing – downstairs**

**Do:**

- Contract abdominals when lowering leg onto next step
- You may monitor for pelvic twisting or tilting by placing hands on pelvis
- You may use hand rail as needed for balance or to help decrease the load on your trunk (left picture)

**Do NOT:**

- Twist or tilt pelvis/low back (middle picture)
- Lean forward, as this results in bending in your back (right picture)

**Key Principle:** Do not twist or tilt pelvis/low back

---

© 2020 van Dillen LR et al. *JAMA Neurology*.

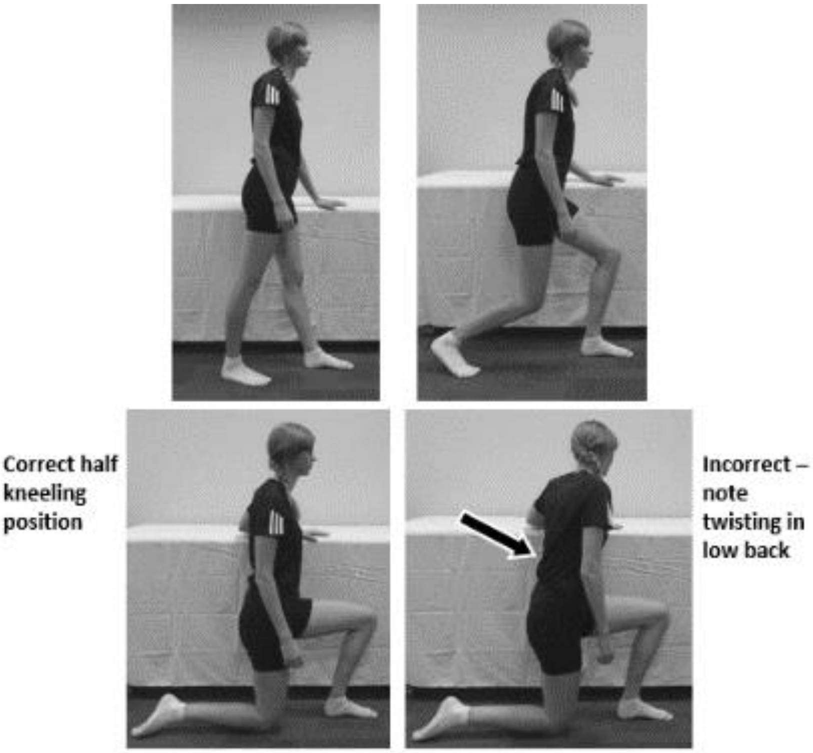


**Lumbar Rotation Skill: Standing to kneeling – step 1 (standing to half kneeling)**
**Do:**

- Stand next to a surface that can be used for support
- Stand with feet in a staggered position (top left picture)
- Bend hips and knees (top right picture) to lower yourself onto one knee in a half kneeling position (bottom left picture)

**Do NOT:** Bend, twist, or shift your low back (bottom right picture)
**Key Principle:** Do not bend, twist, or shift low back

---

© 2020 van Dillen LR et al. *JAMA Neurology*.

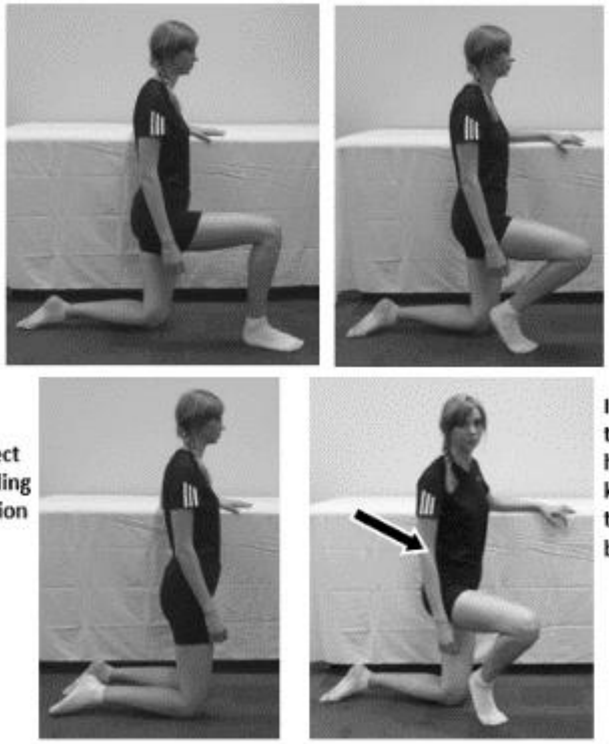


**Lumbar Rotation Skill: Standing to kneeling – step 2 (half kneeling to kneeling)**
**Do:** From half kneeling (top left picture), lower onto the knee of your other leg, using support as needed (top right and bottom left pictures)
**Do NOT:** Bend, twist, or shift your low back when moving into the kneeling position (bottom right picture)
**Key Principle:** Do not bend, twist, or shift low back

---

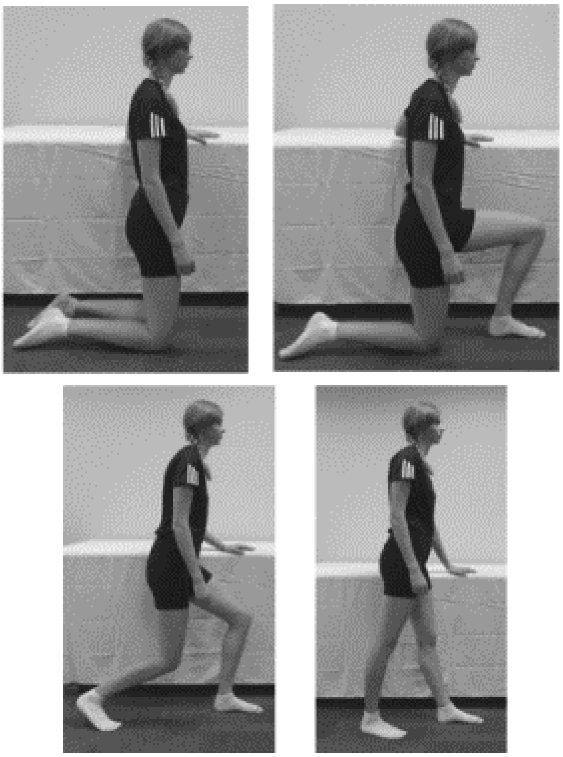

© 2020 van Dillen LR et al. *JAMA Neurology*.

**Lumbar Rotation Skill: Kneeling to standing**
**Do:**
- Hold onto something for support, if available (top left picture)
- Shift weight onto one knee while bringing other leg forward so your foot is on ground (top right picture)
- From this position, push up to standing (bottom pictures)

**Do NOT:** Bend, twist, or shift low back, especially when bringing your leg forward to put foot on the ground
**Key Principle:** Do not bend, twist, or shift low back

---

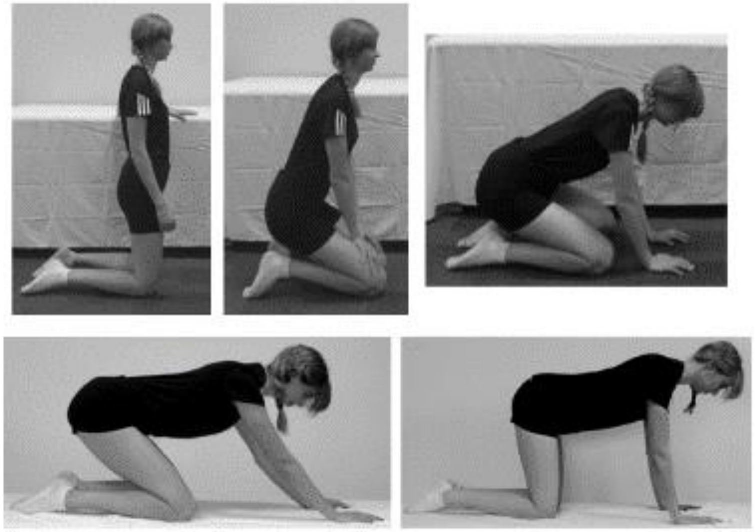

Correct sliding forward    Correct quadruped positioning

**Lumbar Rotation Skill: Kneeling to quadruped**
**Do:**
- From kneeling (top left picture), bend hips and knees so you are sitting back on your heels (top middle picture)
- Lean forward, by bending at hips, to place hands on floor (top right picture)
- Slide hands forward (bottom left picture) with weight evenly distributed between both hands and knees (bottom right picture)

**Do NOT:** Bend, twist, or shift low back, especially when sitting back on your heels and when leaning forward to place hands on floor and sliding forward into the quadruped position
**Key Principle:** Do not bend, twist, or shift low back

---

© 2020 van Dillen LR et al. *JAMA Neurology*.

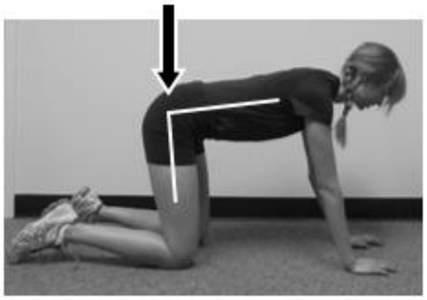

Correct positioning

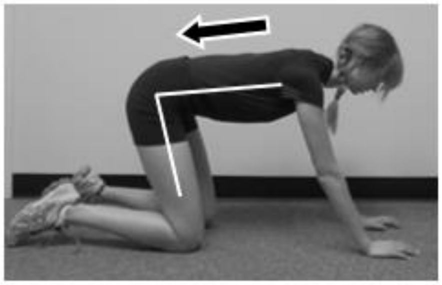

Correct positioning – weight shifted back towards heels

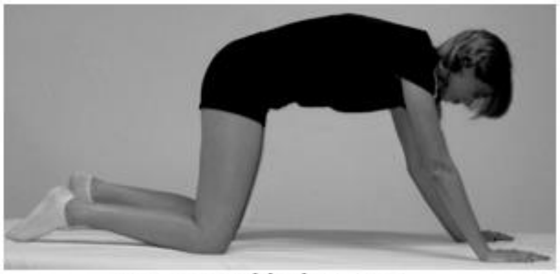

Incorrect positioning – note bending in low back

**Lumbar Rotation Skill: Positioning – quadruped**

**Do:**

- Position yourself so shoulders are directly over hands, and hips are directly over knees, keeping back straight (top left picture)
- Evenly distribute weight between both hands and knees
- If your therapist has advised you to do so, shift weight back towards your heels (top right picture)

**Do NOT:** Bend, twist, or shift your low back (bottom picture)

**Key Principle:** Do not bend, twist, or shift low back

© 2020 van Dillen LR et al. *JAMA Neurology*.

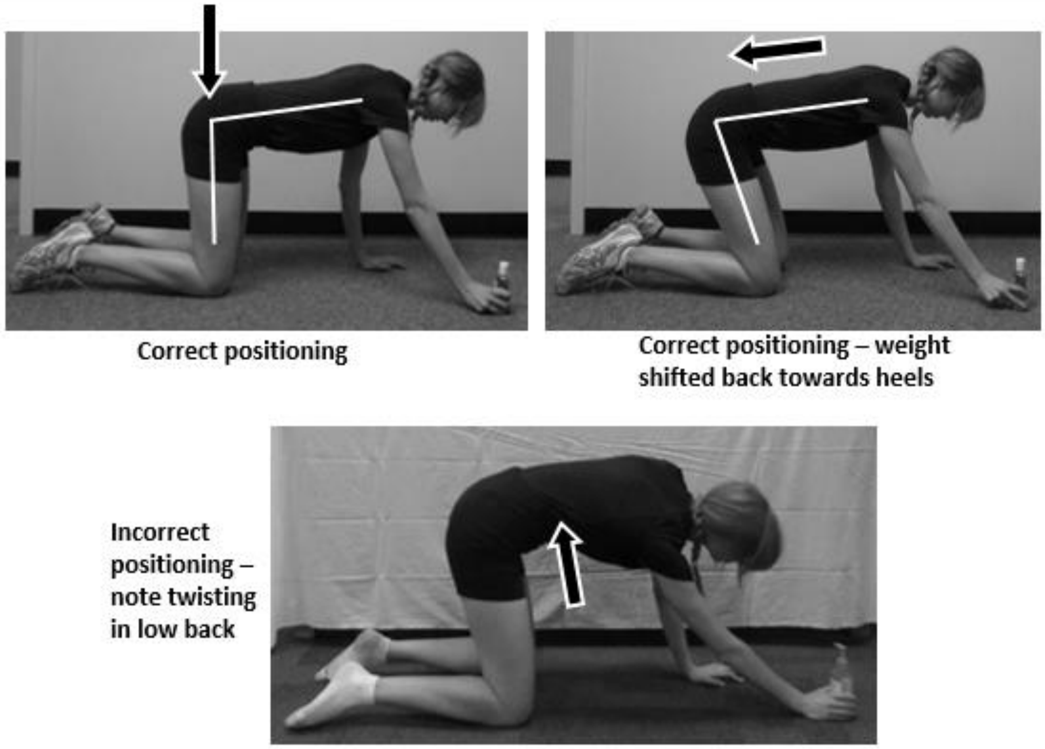


**Lumbar Rotation Skill: Moving object in quadruped**

**Do:**

- If your therapist has advised you to do so, shift weight back towards your heels (top right picture)
- Shift weight onto one arm and leg, moving body as a unit
- Contract abdominals, then lift unweighted arm off floor to reach for object (top left picture)
- Pick up object and move it as necessary
- After replacing object, return hand to its original position and shift weight evenly back onto both hands and knees

**Do NOT:** Bend, twist, or shift low back when reaching for object (bottom picture)

**Key Principle:** Do not bend, twist, or shift low back

© 2020 van Dillen LR et al. *JAMA Neurology*.

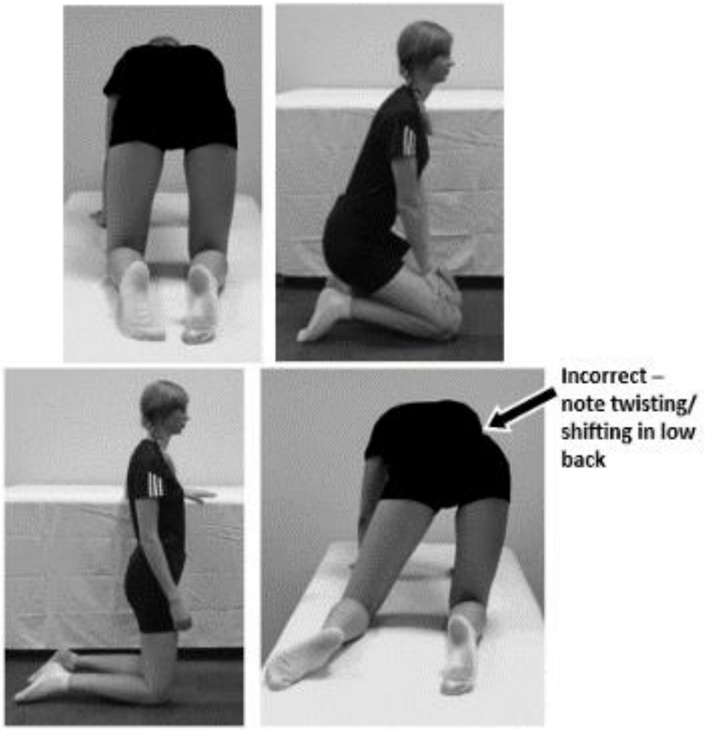


**Lumbar Rotation Skill: Quadruped to standing – step 1 (quadruped to kneeling)**

**Do:**

- Push back evenly with hands (top left picture), sliding hands back and bending hips and knees to sit back on your heels (top right picture)
- Straighten hips using leg muscles to get body into full kneeling (bottom left picture)

**Do NOT:** Push back unevenly with hands, as this would result in twisting/shifting in your low back (bottom right picture)

**Key Principle:** Do not bend, twist, or shift low back

© 2020 van Dillen LR et al. *JAMA Neurology*.

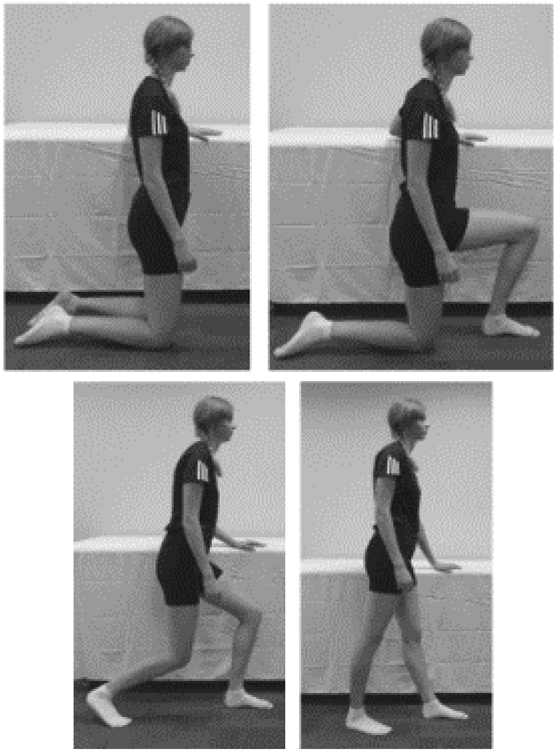

**Lumbar Rotation Skill: Quadruped to standing – step 2 (kneeling to standing)**

**Do:**

- Hold onto something for support, if available (top left picture)
- Bring one leg forward so foot is on ground (top right picture)
- From this position, push up to standing (bottom pictures)

**Do NOT:** Bend, twist, or shift low back, especially when bringing your leg forward to put your foot on the ground

**Key Principle:** Do not bend, twist, or shift low back

© 2020 van Dillen LR et al. *JAMA Neurology*.

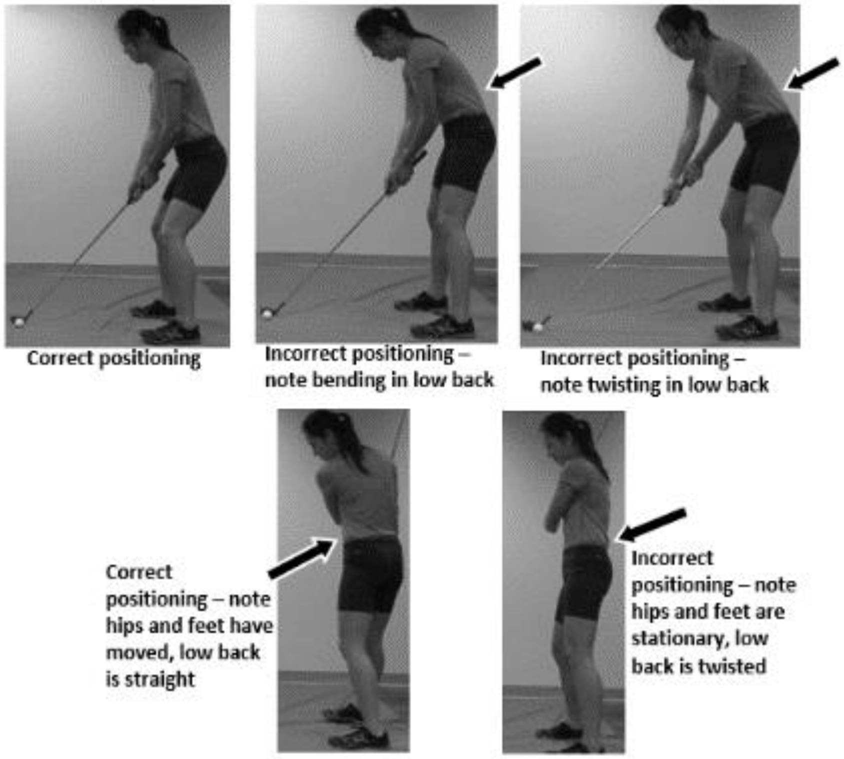


**Lumbar Rotation Skill: Golf – swinging club**

**Do:**

- Keep your back straight when standing next to the ball (top left picture)
- Allow your hips and feet to move (bottom left picture)

**Do NOT:**

- Bend, twist, or shift your low back (top middle and right pictures)
- Keep your feet stationary (bottom right picture)

**Key Principle:** Do not bend, twist, or shift low back

© 2020 van Dillen LR et al. *JAMA Neurology*.

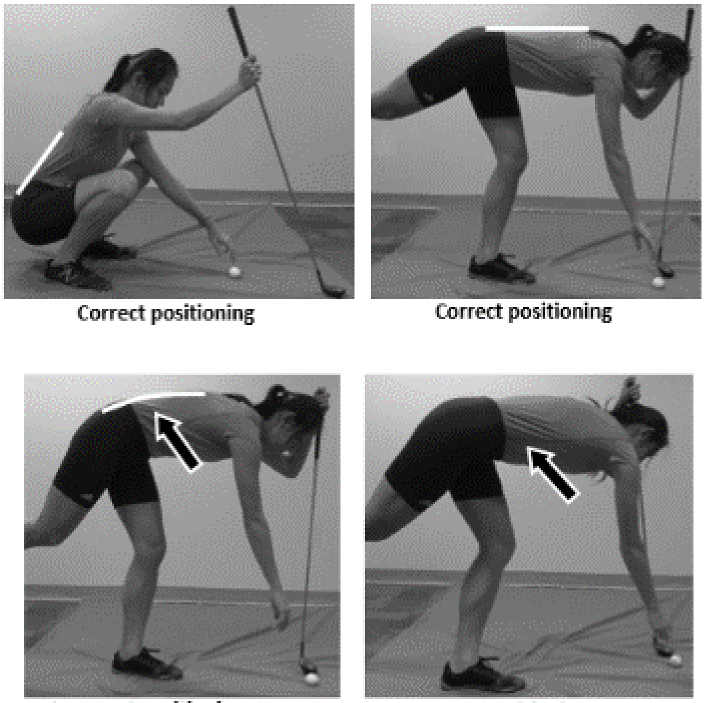

Correct positioning | Correct positioning

Incorrect positioning – note bending in low back | Incorrect positioning – note twisting in low back

**Lumbar Rotation Skill: Golf – picking up ball**

**Do:**

- Pick up ball by bending hips and knees (top left picture)
- You can also pick up the ball by lifting one leg up and back behind you, holding on to your golf club for support. Make sure to keep your back straight (top right picture)

**Do NOT:** Bend, twist, or shift low back (bottom pictures)

**Key Principle:** Do not bend, twist, or shift low back

© 2020 van Dillen LR et al. *JAMA Neurology*.

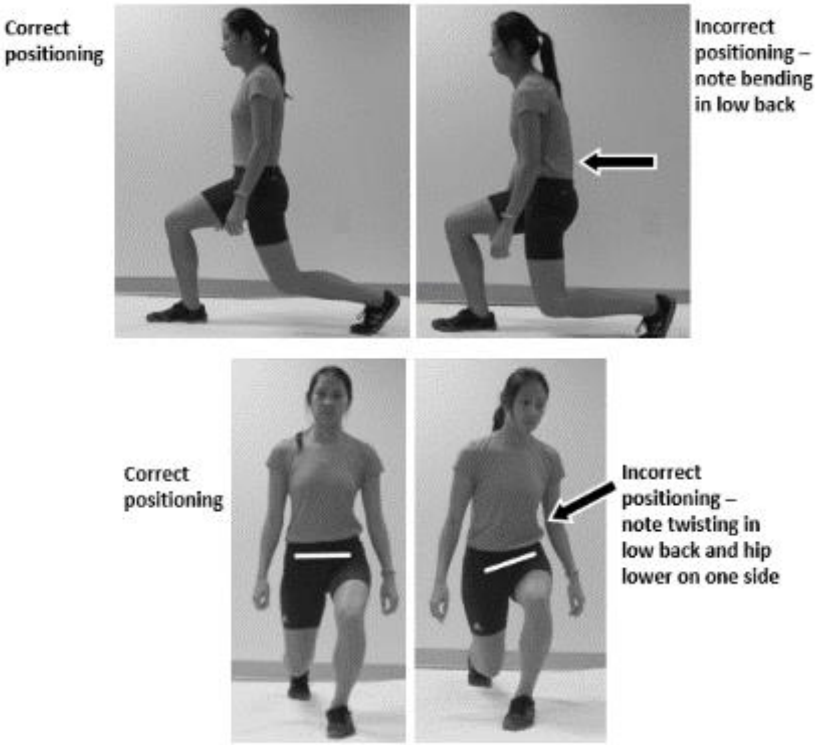


**Lumbar Rotation Skill: Lunge**

**Do:**

- Keep your chest and shoulders up and in line with your hips (left pictures)
- Keep your hips level (bottom left picture)

**Do NOT:**

- Bend, twist, or shift low back (right pictures)
- Let your hip drop on one side (bottom right picture)

**Key Principle:** Do not bend, twist, or shift low back

© 2020 van Dillen LR et al. *JAMA Neurology*.

Correct positioning

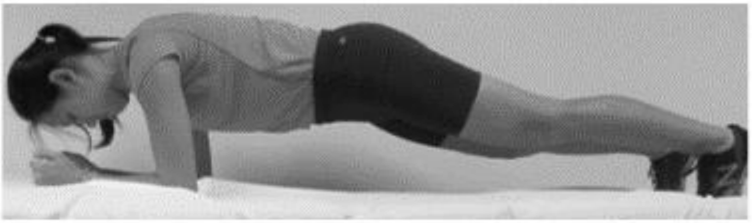

Incorrect positioning – note twisting in low back

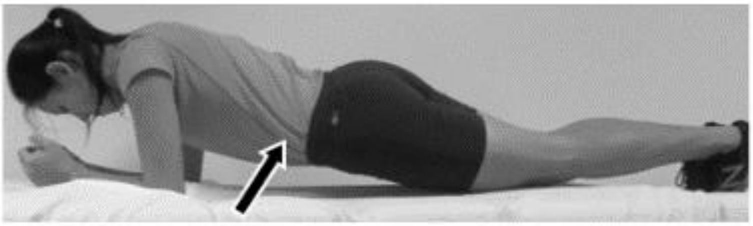

Incorrect positioning – note buttocks are up in the air

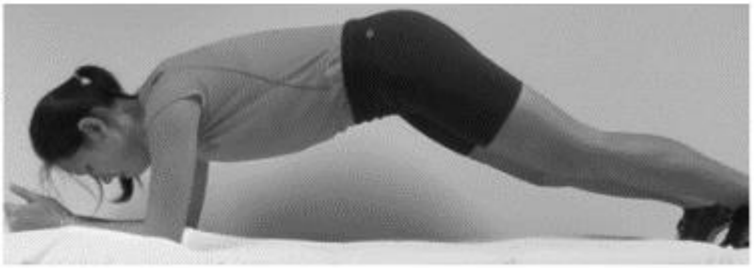

**Lumbar Rotation Skill: Plank position**

**Do:** Keep your back straight and your whole body in line (top picture)

**Do NOT:**

- Bend, twist, or shift low back (middle picture)
- Bend your hips so your buttocks are up in the air (bottom picture)

**Key Principle:** Do not bend, twist, or shift low back

---

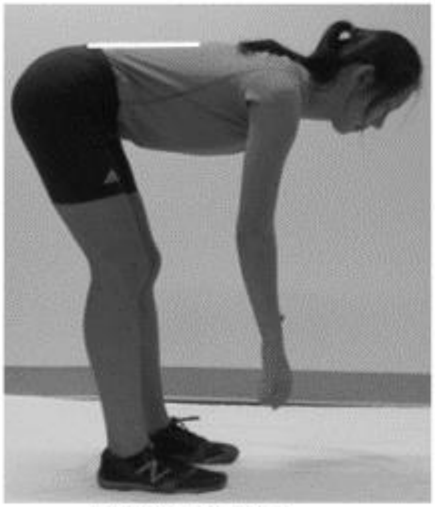

Correct positioning

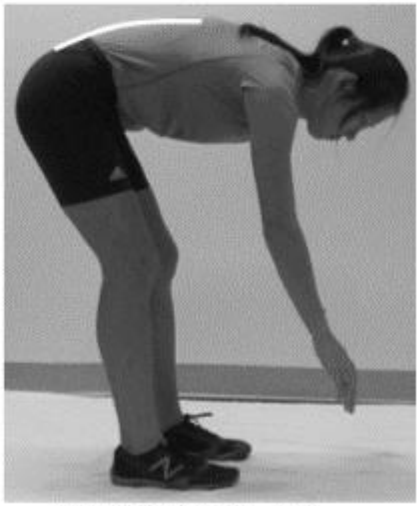

Incorrect positioning – note bending in low back

**Lumbar Rotation Skill: Bending over**

**Do:**

- Bend in your hips and keep your back straight (left picture)
- When you come back up from bending over, keep your back straight and use your buttock muscles to bring your hips/buttocks underneath you

© 2020 van Dillen LR et al. *JAMA Neurology*.

**Do NOT:** Bend, twist, or shift low back (right picture)
**Key Principle:** Do not bend, twist, or shift low back

---

**Motor Skills Training – Lumbar Extension Rotation Handouts**

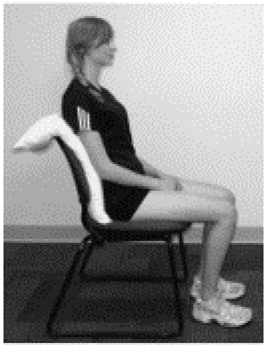

Correct

**Lumbar Extension Rotation Skill: Sitting position – correct**
**Do:**

- Keep back well supported without any arching, twisting, or shifting
- A pillow may be placed lengthwise behind back to help keep back relaxed
- Keep feet supported on floor (if feet do not reach floor, place something underneath feet to support them)
- Keep legs relaxed

**Key Principle:** Do not arch, twist, or shift low back

---

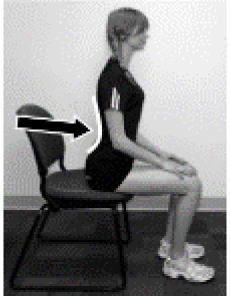

Incorrect – arching in low back; back unsupported

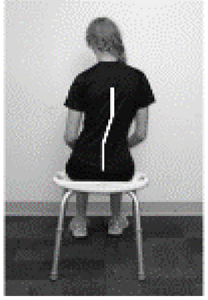

Incorrect – shift in low back

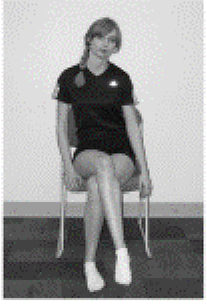

Incorrect – legs crossed

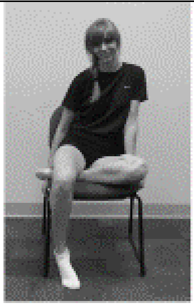

Incorrect – sitting on one leg

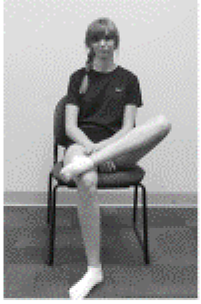

Incorrect – resting one ankle on the opposite knee

**Lumbar Extension Rotation Skill: Sitting position – incorrect**
**Do NOT:**

- Sit with low back arched, twisted, or shifted
- Sit on edge of chair with back unsupported
- Cross legs
- Sit on one leg
- Rest one ankle on the opposite knee

---

© 2020 van Dillen LR et al. *JAMA Neurology*.

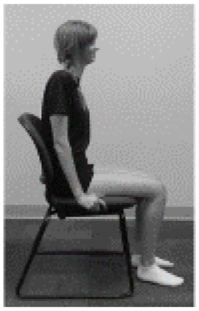

Correct hand placement for pushing up in chair

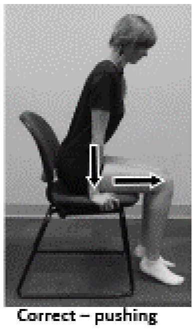

Correct – pushing up to move forward in chair

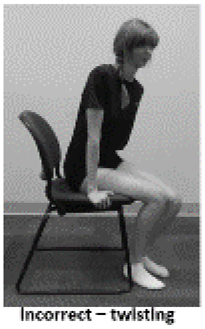

Incorrect – twisting in low back/hips

Incorrect – leaning against the back of the chair; arching in low back

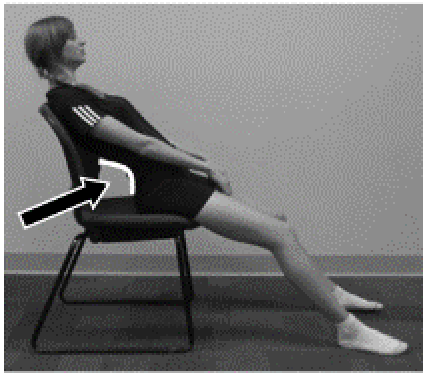

**Lumbar Extension Rotation Skill: Sit to stand – step 1**

**Do:**

- If seated at a desk or another surface, move chair back so there is room to lean forward and stand up
- Move forward in chair by pushing on the chair or armrests with both hands (top left and middle pictures)
    - Keep hands in front of hips

**Do NOT:**

- Twist low back/hips from side to side to move to edge of chair (top right picture)
- Lean against back of chair to move forward in chair (bottom picture)

**Key Principle:** Do not twist low back/hips

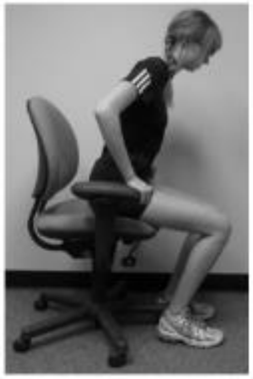

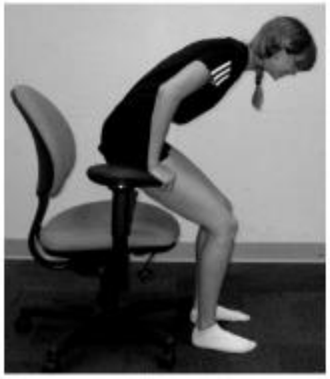

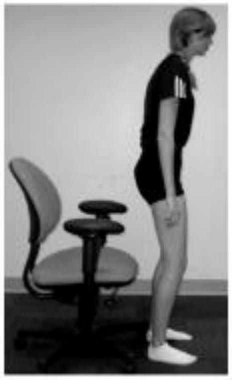

**Skill: Sit to stand – step 2**

**Do:**

- Once at the edge of the chair (left picture), place feet behind your knees
- Lean forward at hips keeping back flat or slightly bent as you stand up from chair, using armrests if available (middle and right pictures)

© 2020 van Dillen LR et al. *JAMA Neurology*.

**Do NOT:** Arch or twist your low back as you come to standing

**Key Principle:** Do not arch or twist low back

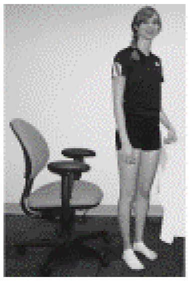

Correct

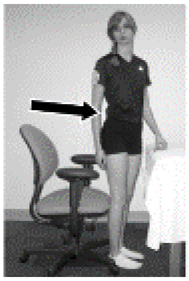

Incorrect – turning trunk with feet fixed; note twisting in low back

**Lumbar Extension Rotation Skill: Turning**

**Do:** Once in standing, turn away from surface by stepping with each foot (left picture)
**Do NOT:** Turn your trunk with your feet fixed (right picture)
**Key Principle:** Do not twist low back

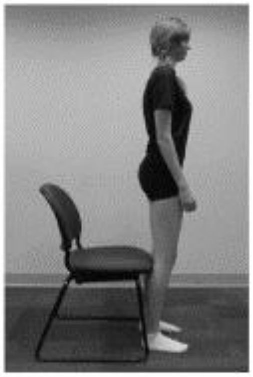

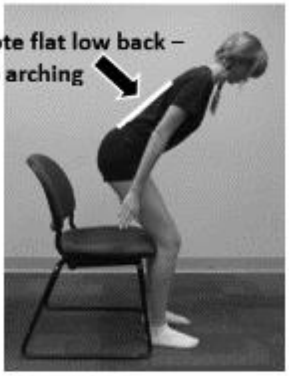


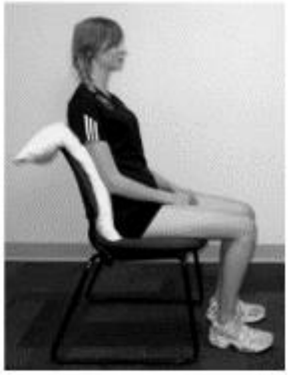

**Lumbar Extension Rotation Skill: Stand to sit**

**Do:**

- Position chair so you are able to walk up to it and turn all the way around so the back of your legs are against the chair (left picture)
- Bend hips and knees to lower yourself into the chair without arching low back (middle picture)
- Move back in chair and adjust position as necessary (right picture)

**Do NOT:** Arch or twist your low back as you sit down
**Key Principle:** Do not arch or twist low back

© 2020 van Dillen LR et al. *JAMA Neurology*.

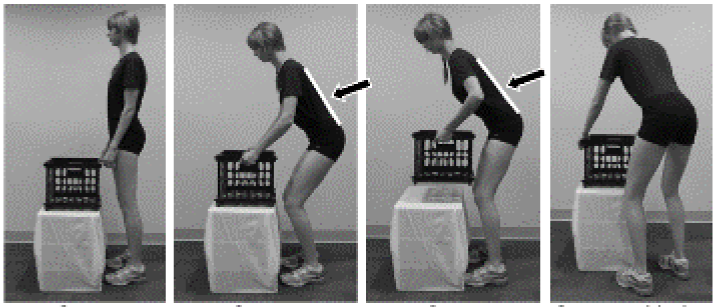

Correct distance | Correct bending | Correct lifting | Correct positioning when item is off to the side

**Lumbar Extension Rotation Skill: Picking up an object – correct**

**Do:**

- Stand close to object with feet shoulder width apart and object directly in front of you (left picture)
- Keeping back flat, bend hips and knees (middle left picture)
- Contract abdominals
- Pick up object and straighten your legs to lift, keeping the object close to your body (middle right picture)
- If an object is located off to the side, position yourself directly behind it (right picture)

**Key Principle:** Bend in hips and knees; do not arch low back

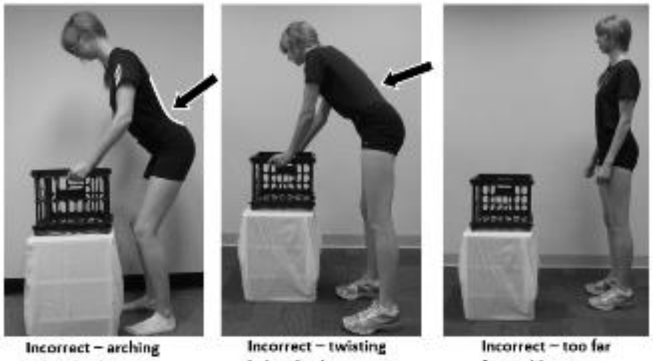

Incorrect – arching in low back | Incorrect – twisting in low back | Incorrect – too far from object

**Lumbar Extension Rotation Skill: Picking up an object – incorrect**

**Do NOT:**

- Arch, twist, or shift low back (left picture)
- Reach for an object off to the side, as this would result in twisting of low back (middle picture)
- Stand too far away from the object (right picture)

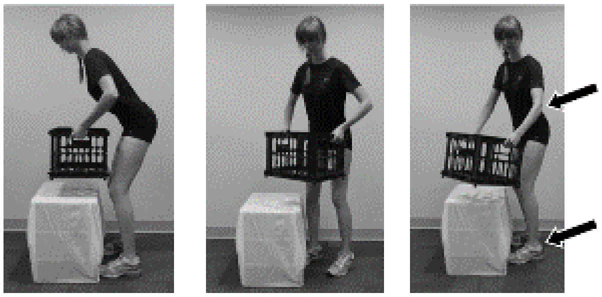

Correct | Correct | Incorrect – turning body with feet fixed; note twisting in low back

**Lumbar Extension Rotation Skill: Placing object in same or different location**

© 2020 van Dillen LR et al. *JAMA Neurology*.

**Do:**

- Keep object close to body as you lift
- Place object back in the same location by bending hips and knees (left picture)
- If object needs to be placed in a different location, move whole body by stepping with each foot (middle picture)

**Do NOT:** Turn your trunk while keeping your feet fixed (right picture)
**Key Principle:** Bend in hips and knees; do not arch or twist low back

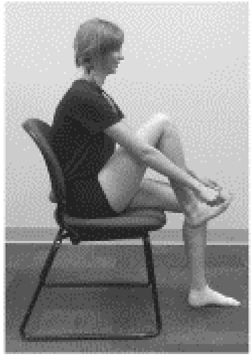
Correct

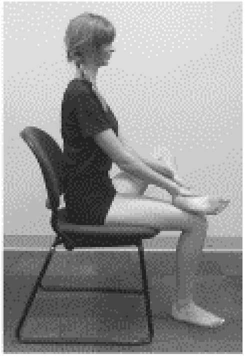
Correct

**Lumbar Extension Rotation Skill: Donning a sock – initiation phase**

**Do:**

- Contract abdominals
- Bend hip and knee to lift foot up off floor (left picture)
- Place foot on surface or rest it on opposite knee (right picture)

**Do NOT:** Arch, twist, or shift your low back
**Key Principle:** Do not arch, twist, or shift low back

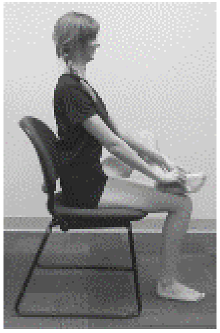
Correct

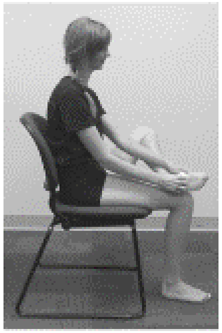
Correct

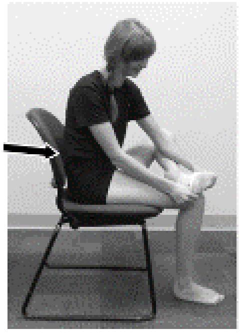
Incorrect – note twisting in low back

**Lumbar Extension/Rotation Skill: Donning a sock – completion phase**

**Do:**

- Put sock on foot, keeping low back flat (left picture) or slightly bent (middle picture)
- Contract abdominals and lower foot back to floor

**Do NOT:** Arch or twist your low back, especially when leaning forward to put the sock on your foot (right picture)
**Key Principle:** Do not arch or twist low back

© 2020 van Dillen LR et al. *JAMA Neurology*.

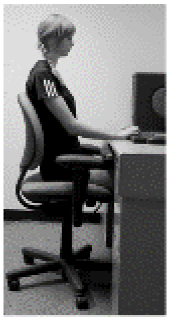

Correct sitting

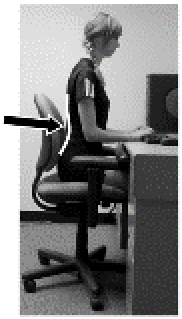

Incorrect sitting – arching in low back; back unsupported

**Lumbar Extension Rotation Skill: Sitting position at work surface**

**Do:**

- Sit comfortably in a chair with appropriate positioning materials
- Move chair close to work surface, making sure to keep back supported by, and relaxed into, the chair (left picture)

**Do NOT:** Sit away from the surface so your back is arched and/or not supported (right picture)

**Key Principle:** Do not arch low back

---

**Primary work zone**: The area in which you can work when performing a sweeping motion with your arm when your elbow is bent to 90 degrees.

**Secondary work zone**: The area in which you can work when performing a sweeping motion with your arm when it is fully extended.

**Tertiary work zone**: The area that is outside of your reach when your arm is fully extended.

**Lumbar Extension Rotation Skill: Sitting position – work zones**

**Do:**

- Position items you will use repetitively within the **primary** work zone
- Position items you will use occasionally within the **secondary** work zone
- Position items you rarely use within the **tertiary** work zone
- When reaching for items in the tertiary work zone, lean forward by bending at hips, keeping back flat

**Do NOT:**

- Arch, twist, or shift your low back when reaching for items
- Keep items you use frequently too far away from you

**Key Principle:** Keep items you use often close to you

---

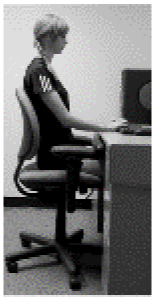

Correct sitting

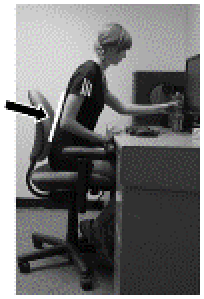

Correct reaching – bending in hips and not in low back

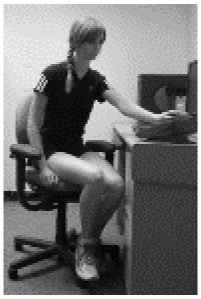

Correct reaching – turning whole body

© 2020 van Dillen LR et al. *JAMA Neurology*.

**Lumbar Extension Rotation Skill: Manipulate/reach for objects on work surface while sitting – correct**

**Do:**

- Sit comfortably in a chair with the appropriate positioning materials
- Keep items you will be using often (e.g. keyboard, mouse, etc.) within the **primary** work zone (left picture)
- When reaching for an object on work surface, contract abdominals, then lean forward by bending at hips (middle picture)
- If you need to reach for something off to the side, turn whole body to face object (right picture)

**Key Principle:** Do not arch or twist low back; bend in hips when reaching

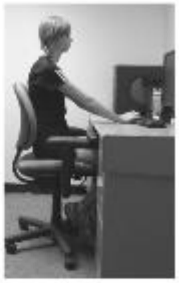

Incorrect sitting – keyboard too far away

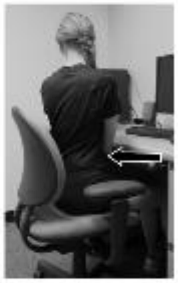

Incorrect reaching – note twisting in low back (legs facing forward, shoulders facing off to the side)

**Lumbar Extension Rotation Skill: Manipulate/reach for objects on work surface while sitting – incorrect**

**Do NOT:**

- Keep items you use frequently far away from you
- Arch, twist, or shift low back when reaching for an object

---

© 2020 van Dillen LR et al. *JAMA Neurology*.

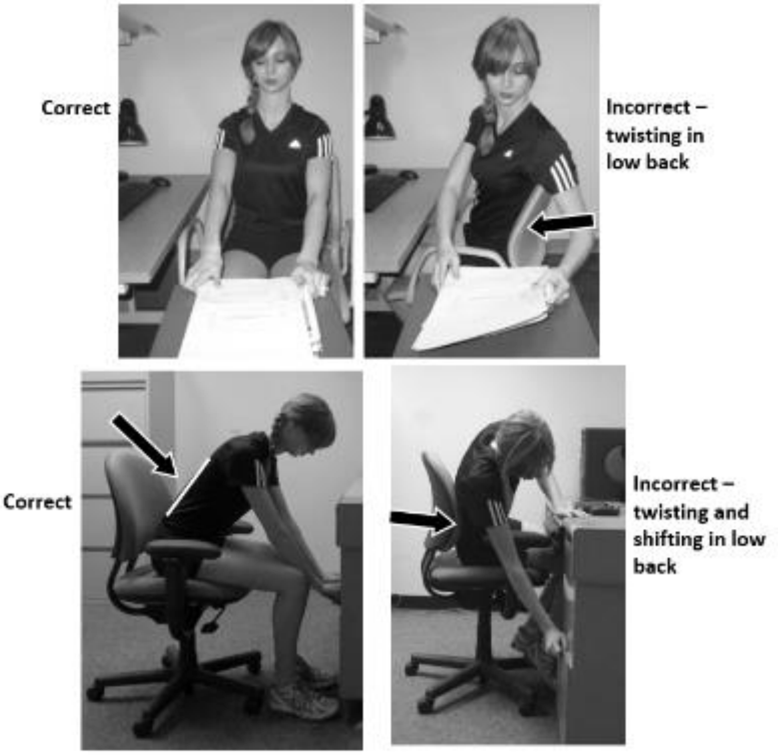


**Lumbar Extension Rotation Skill: Manipulate/reach for objects outside of work surface while sitting**

**Do:**

- Sit comfortably in a chair with the appropriate positioning materials
- When reaching for something lower than work surface, turn chair or move chair to reach for object (left pictures)
    - If chair does not swivel, turn whole body

**Do NOT:** Arch, twist, or shift in your low back to reach for an object (right pictures)

**Key Principle:** Do not arch, twist, or shift low back

---

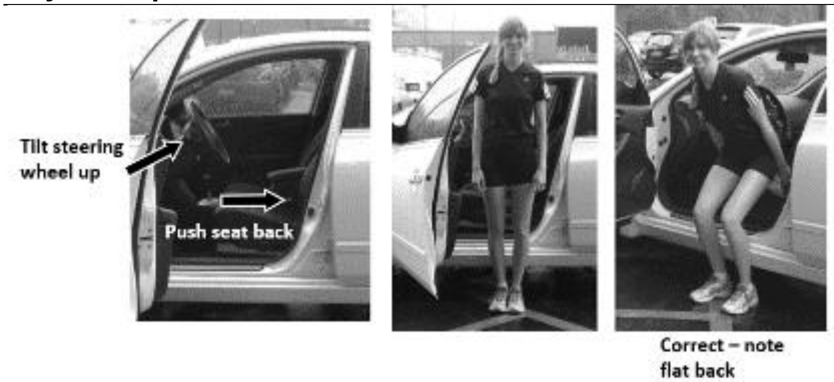


**Lumbar Extension Rotation Skill: Getting into car – step 1**

**Do:**

- Open door, adjust steering wheel and seat position as necessary (left picture)
- Stand facing away from car (middle picture)
- Lower yourself down to sit on the edge of the seat, keeping back flat or slightly bent as you sit down (right picture)

**Do NOT:** Arch, twist, or shift your low back

**Key Principle:** Bend in hips and knees; do not arch, twist, or shift low back

---

© 2020 van Dillen LR et al. *JAMA Neurology*.

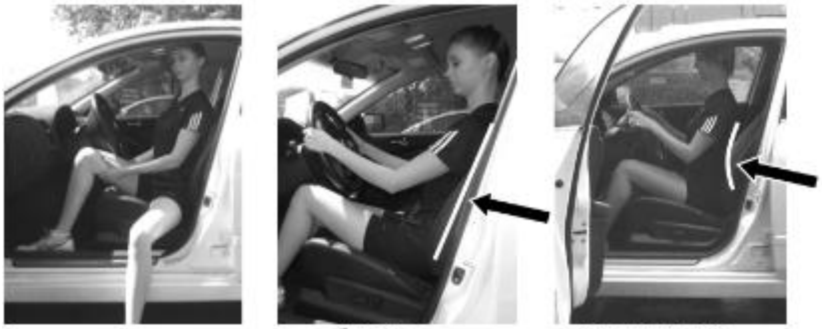

Correct

Incorrect – note arching in low back

**Lumbar Extension Rotation Skill: Getting into car – step 2**

**Do:**

- Contract abdominals, then lift each leg into car, one at a time
    - Use hands to help lift legs, if necessary (left picture)
- Turn whole body to face forward in car
- Sit comfortably in seat using appropriate positioning materials
- Adjust position of seat and/or steering wheel so back is flat (middle picture)
    - Get as close to the steering wheel as you can (elbows bent, back flat)
- *Pivot* on heel to move from pedal to pedal while driving

**Do NOT:**

- Lean forward or to one side, resulting in arching, twisting, or shifting of your low back (right picture)
- Move both legs at the same time
- *Lift* foot from pedal to pedal when driving

**Key Principle:** Do not arch, twist, or shift low back

---

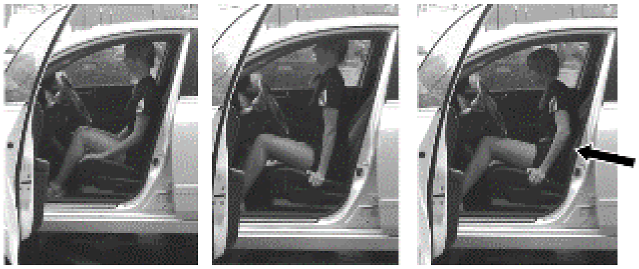

Wheel tilted up, seat pushed back

Correct – move to side by pushing up with both hands

Incorrect – move to side by scooting buttocks over; note twisting in low back (shoulders not aligned over hips)

**Lumbar Extension Rotation Skill: Getting out of car – step 1**

**Do:**

- Adjust steering wheel and seat position as necessary (left picture)
- Move to side of seat while still facing forward in car
    - Move closer to side of seat by pushing on the seat with both hands equally (middle picture)

**Do NOT:**

- Arch, twist, or shift your low back, especially when moving to the side of the seat (right picture)

**Key Principle:** Do not arch, twist, or shift low back

---

© 2020 van Dillen LR et al. *JAMA Neurology*.

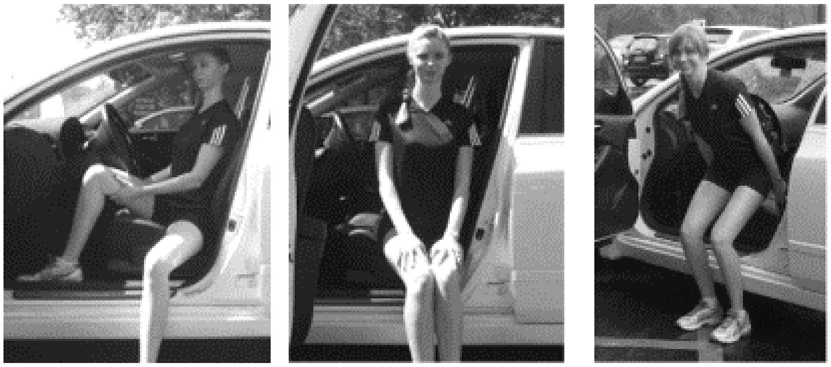

**Lumbar Extension Rotation Skill: Getting out of car – step 2**

**Do:**

- Contract abdominal muscles as you lift each leg out of car, one at a time
    - Use hands to help lift legs, if necessary (left picture)
- Sit on the edge of the seat with feet on ground (middle picture)
- Lean forward by bending at hips and stand up, keeping back flat (right picture)

**Do NOT:**

- Move both legs at the same time
- Arch, twist, or shift your low back

**Key Principle:** Do not arch, twist, or shift low back

---

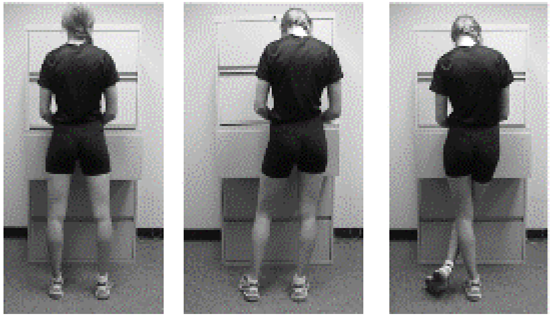

Correct standing | Incorrect standing – weight shifted onto one foot | Incorrect standing – legs crossed

**Lumbar Extension Rotation Skill: Standing position**

**Do:**

- Stand with feet shoulder width apart, keeping weight evenly distributed over both feet (left picture)
- Stand close to surface on which you will be working
- Surface should be at the height of your elbow for most tasks/activities

**Do NOT:**

- Stand with weight shifted onto one foot (middle picture)
- Stand with feet/legs crossed (right picture)
- Hold a load in one hand or on one side on a regular basis or for an extended period of time

**Key Principle:** Do not twist or shift low back

---

**Primary work zone**: The area in which you can work when performing a sweeping motion with your arm when your elbow is bent to 90 degrees.

© 2020 van Dillen LR et al. *JAMA Neurology*.

**Secondary work zone**: The area in which you can work when performing a sweeping motion with your arm when it is fully extended.

**Tertiary work zone**: The area that is outside of your reach when your arm is fully extended.

**Lumbar Extension Rotation Skill: Standing position – work zones**

**Do:**

- Position items you will use repetitively within the **primary** work zone
- Position items you will use occasionally within the **secondary** work zone
- Position items you rarely use within the **tertiary** work zone
- When reaching for items in tertiary work zone, lean forward by bending at hips, keeping back flat

**Do NOT:**

- Arch, twist, or shift your low back when reaching for items
- Keep items you use frequently too far away from you

**Key Principle:** Keep items you use often close to you

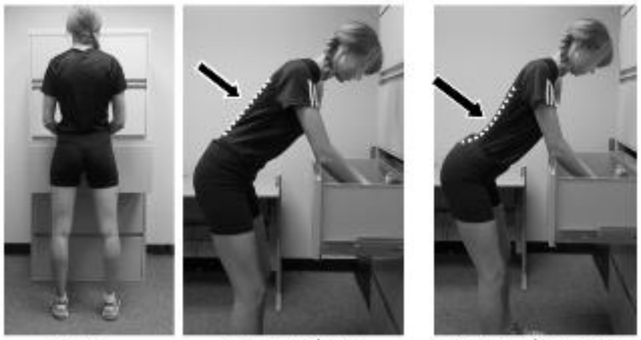

Correct standing | Correct reaching – note straight back | Incorrect reaching – note arching in low back

**Lumbar Extension Rotation Skill: Activities while standing in one place**

**Do:**

- Stand comfortably with the appropriate positioning materials (left picture)
- Keep items used often within the **primary** work zone
- When reaching for an object on work surface, contract abdominals, then lean forward by bending at hips
- If activity requires strength, surface should be slightly lower than elbow height
- If activity requires precision, surface should be slightly higher than elbow height

**Do NOT:** Arch, twist, or shift your low back when reaching for an object (middle and right pictures)

**Key Principle:** Bend in hips and knees; do not arch, twist, or shift low back

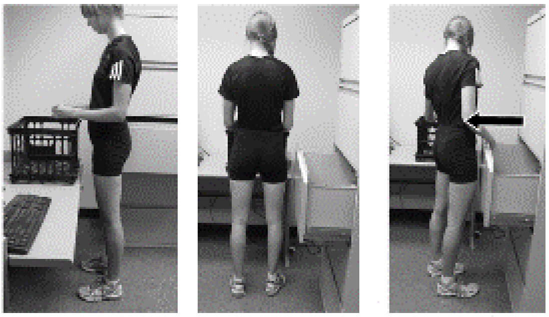

Correct reaching | Correct reaching | Incorrect reaching – note twisting in low back

© 2020 van Dillen LR et al. *JAMA Neurology*.

**Lumbar Extension Rotation Skill: Activities that require standing and walking**

**Do:**

- Stand comfortably with the appropriate positioning materials
- When reaching for an object that is not directly in front of you, turn whole body to face object (left and middle pictures)
    - Alternatively, you could side step so object is directly in front of you
- If you need to walk to another location, step away from surface, turn whole body in the direction you need to go, and then walk

**Do NOT:** Twist or shift in your low back when reaching for an object (right picture)

**Key Principle:** Bend in hips and knees; do not twist or shift low back

---

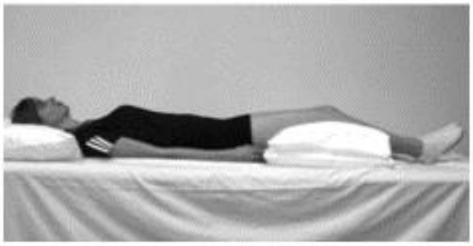

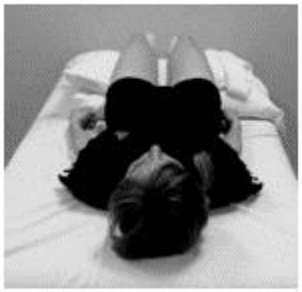

**Lumbar Extension Rotation Skill: Positioning – back lying**

**Do:**

- Place pillows under knees to relieve pressure on low back
- Keep back flat
- Relax legs
- Practice log rolling (i.e. moving body as a unit) before going to sleep

**Key Principle:** Do not lie with low back arched, twisted, or shifted

---

© 2020 van Dillen LR et al. *JAMA Neurology*.

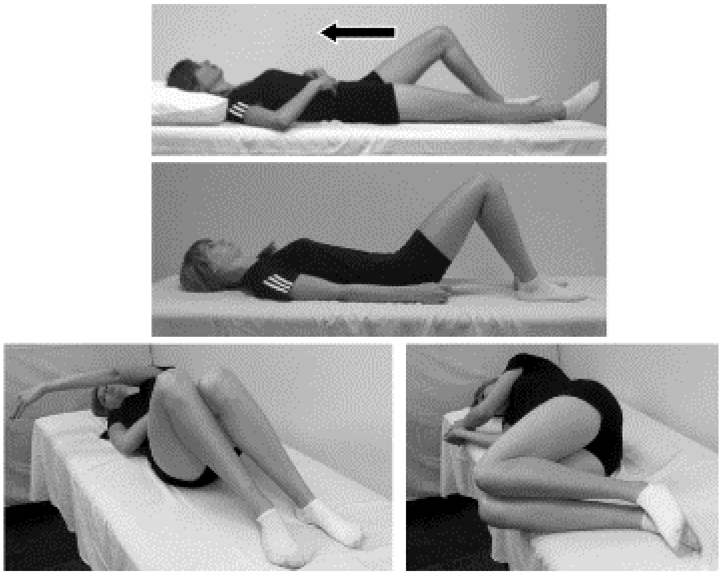

.

**Lumbar Extension Rotation Skill: Rolling back lying to side lying**

**Do:**

- Remove covers and any positioning materials
- Contract abdominals and *slide* each heel up one at a time (top picture) so hips and knees are bent (middle picture)
- Reach with arm to log roll to side (bottom pictures)
- Lead with arms (arms before legs; A before L), moving body as a unit

**Do NOT:**

- Twist upper body independently of lower body
- Push with feet into surface
- *Lift* legs off surface of bed

**Key Principle:** Move body as a unit

---

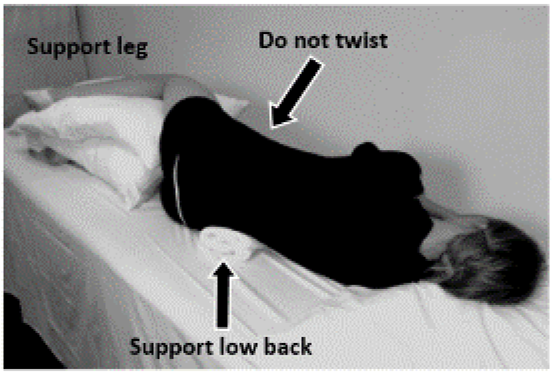


**Lumbar Extension Rotation Skill: Positioning – side lying**

**Do:**

- Place as many pillows between knees as needed to keep pelvis in good alignment (i.e. no twisting or tilting)
- Legs should be aligned on top of each other
- Hips and knees can be bent forward a little so back is slightly bent
- Place a small folded towel at waist, just above hip
- Use a pillow to support head in a neutral position (i.e. no bending or twisting)

**Do NOT:**

- Lie with low back arched, twisted, or shifted

© 2020 van Dillen LR et al. *JAMA Neurology*.

- Lie with pelvis twisted or tilted

**Key Principle:** Do not lie with low back arched, twisted, or shifted

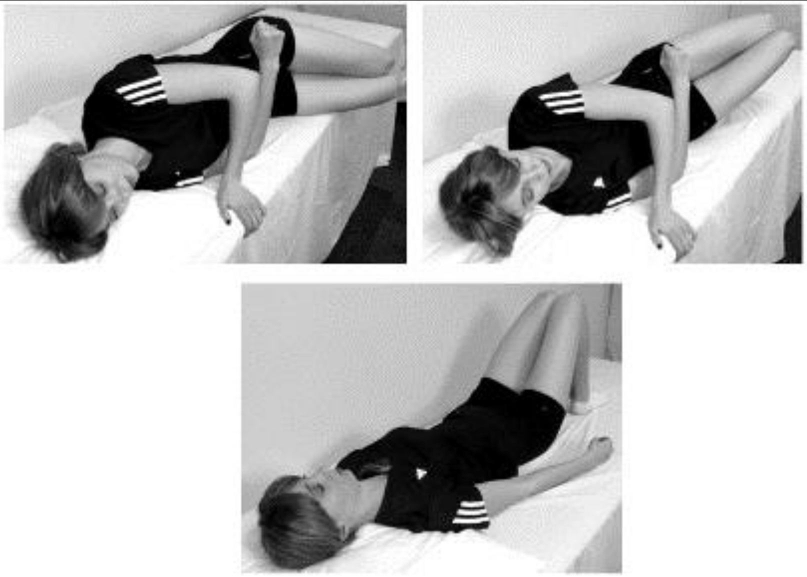

**Lumbar Extension Rotation Skill: Rolling from side lying to back lying**

**Do:**

- Remove covers and any positioning materials
- Push hand of top arm, elbow of bottom arm, and lower leg into surface and log roll onto back, moving body as a unit (top pictures)
- Once on back (bottom picture), contract abdominals as you *slide* one heel down at a time
- Position in back lying using materials as appropriate

**Do NOT:** Twist upper body independently of lower body, especially when reaching over with top arm to push from surface

**Key Principle:** Move body as a unit

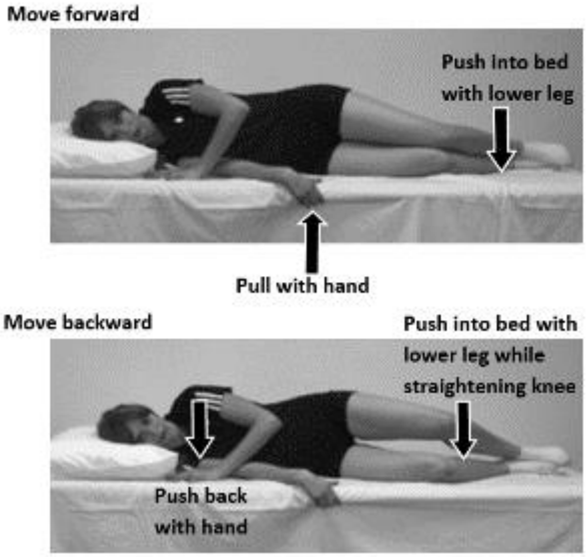


**Lumbar Extension Rotation Skill: Moving in bed – side lying**

**Do:**

- Remove covers and any positioning materials

© 2020 van Dillen LR et al. *JAMA Neurology*.

- To move forward (top picture)
  - Pull on side of bed with bottom hand and push into bed with lower leg
- To move backward (bottom picture)
  - Push back with top hand and side of lower leg while straightening knee

**Do NOT:** Arch, twist, or shift your low back
**Key Principle:** Do not arch, twist, or shift low back

---

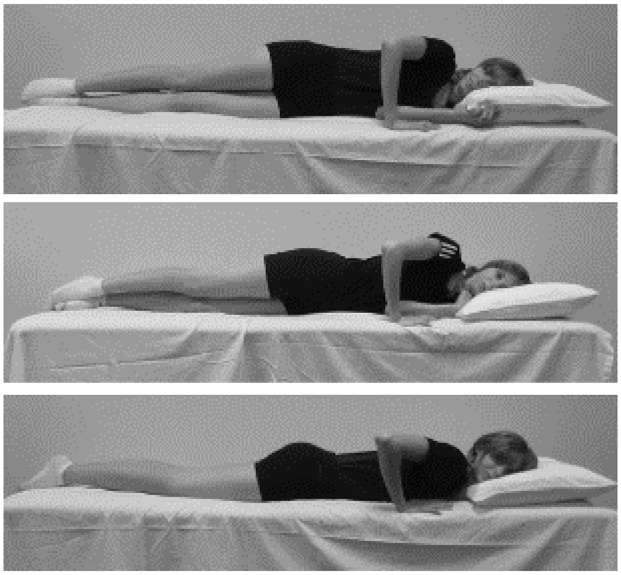

**Lumbar Extension Rotation Skill: Rolling from side lying to face lying**

**Do:**

- Remove covers and any positioning materials
- Straighten legs (top picture)
- Log roll onto stomach, moving body as a unit (middle and bottom pictures)

**Do NOT:** Twist upper body independently of lower body
**Key Principle:** Move body as a unit

---

Correct position

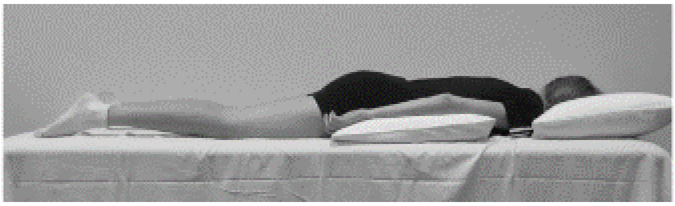

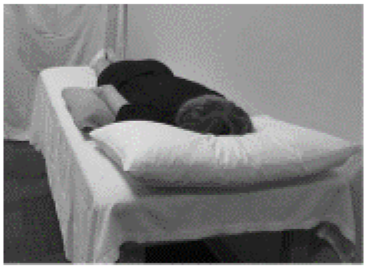

Correct position

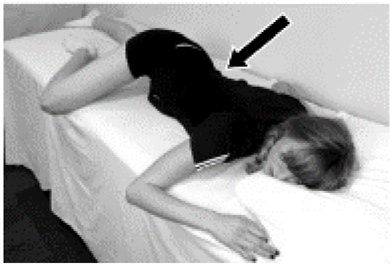

Incorrect position – hip and knee bent; note twisting in low back

**Lumbar Extension Rotation Skill: Positioning – face lying**

**Do:** Use pillows as needed to help prevent arching, twisting or shifting in your low back
**Do NOT:**

© 2020 van Dillen LR et al. *JAMA Neurology*.

- Lie with one hip and knee bent resulting in twisting in your low back
- Place arms above head

**Key Principle:** Do not lie with low back twisted or shifted

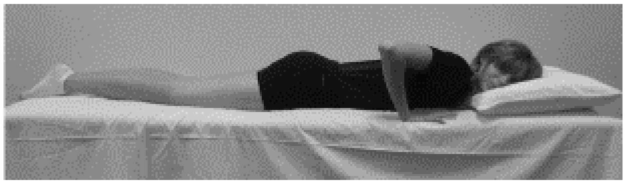

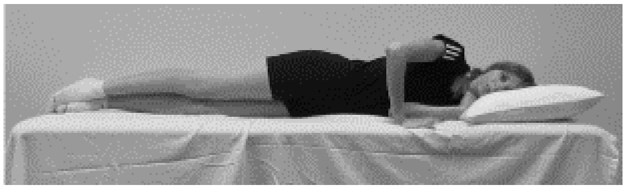

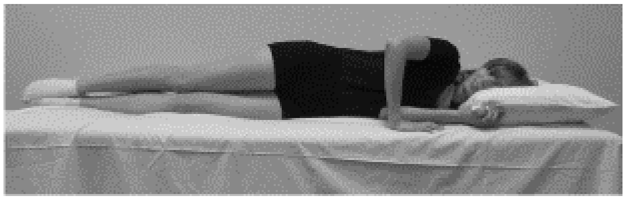

**Lumbar Extension Rotation Skill: Rolling from face lying to side lying**

**Do:**

- Remove covers and any positioning materials
- If rolling onto left side, tuck left arm under body as much as possible
- Bend right arm up so hand is by chest (top picture)
- Contract abdominals, and push right hand into surface to roll onto left side, moving body as a unit (middle and bottom pictures)
- Position in side lying with appropriate materials

**Do NOT:** Twist upper body independently of lower body

**Key Principle:** Move body as a unit

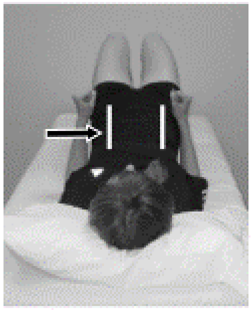

Correct

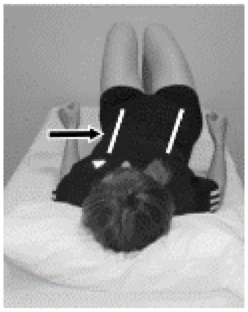

Incorrect – hips not in line with shoulders

**Lumbar Extension Rotation Skill: Moving in bed – back lying**

**Do:**

- Remove covers and any positioning materials
- Contract abdominals and *slide* each heel up one at a time so hips and knees are bent
- Bend both arms so elbows are bent to 90 degrees
- Push down with elbows and feet at same time
- Move body to side in small increments
- Keep hips in line with shoulders, moving trunk as a unit (left picture)

© 2020 van Dillen LR et al. *JAMA Neurology*.

**Do NOT:** Move hips while keeping shoulders fixed (right picture), as this will result in a shift in your low back
**Key Principle:** Move trunk as a unit

---

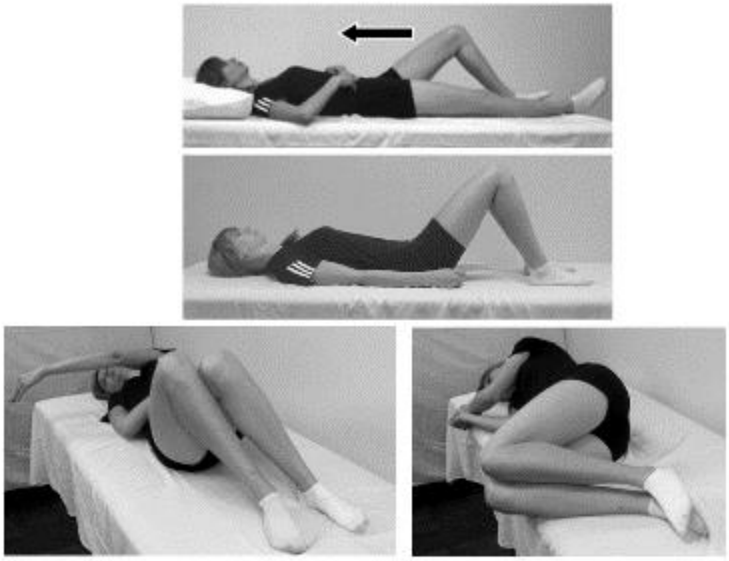

**Lumbar Extension Rotation Skill: Back lying to sit – step 1 (back lying to side lying)**

**Do:**

- Remove covers and any positioning materials
- Contract abdominals as you *slide* each heel up one at a time (top picture) so hips and knees are bent (middle picture)
- Reach with arm and push feet into surface to log roll to side (bottom pictures)
- Lead with arms (arms before legs; A before L), moving body as a unit

**Do NOT:**

- Twist upper body independently of lower body
- *Lift* legs off surface of bed

**Key Principle:** Move body as a unit

---

© 2020 van Dillen LR et al. *JAMA Neurology*.

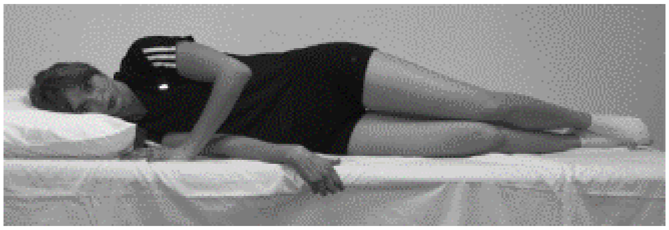

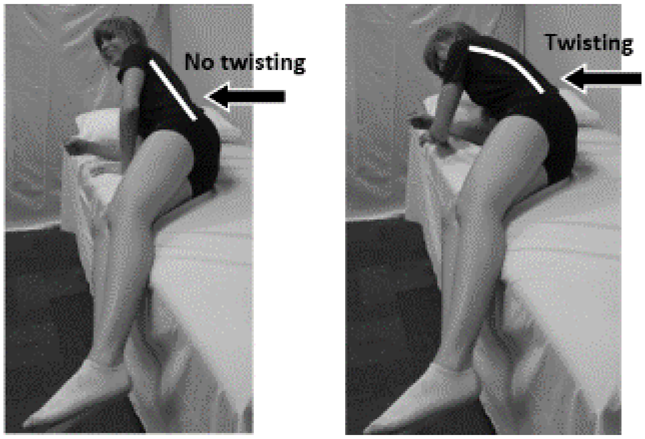


Correct

Incorrect – twisting in low back

**Lumbar Extension Rotation Skill: Back lying to sit – step 2 (side lying to sit; using top arm)**

**Do:**

- From side lying (top picture), push into surface with top arm and elbow of arm on surface, while letting legs drop over edge of surface (bottom left picture)
- Keep low back flat or slightly bent as you come to sitting
- Move forward to edge of surface so feet are positioned on floor

**Do NOT:** Twist your low back, especially when reaching for the surface and pushing into surface with your top hand (bottom right picture)

**Key Principle:** Move trunk as a unit

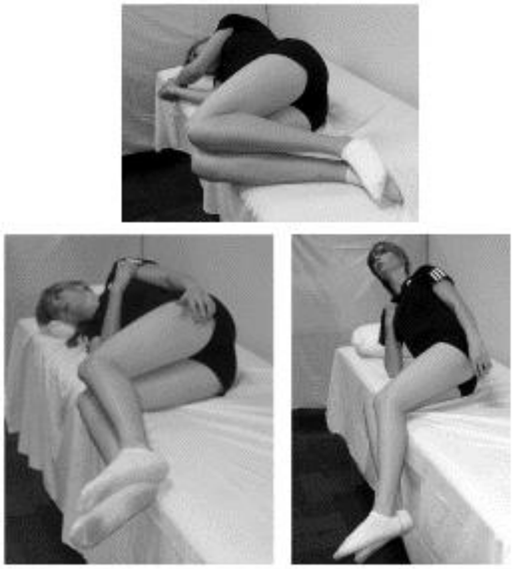

© 2020 van Dillen LR et al. *JAMA Neurology*.

**Lumbar Extension Rotation Skill: Back lying to sit – step 2 (side lying to sit; without using top arm)**

**Do:**

- From side lying (top picture), push into surface with elbow of arm on surface while letting legs drop over edge of surface (bottom pictures)
- Keep low back flat or slightly bent as you come to sitting
- Move forward to edge of surface so feet are positioned on floor

**Do NOT:**

- Twist low back
- Use top arm to help push as this will result in twisting in low back

**Key Principle:** Move trunk as a unit

---

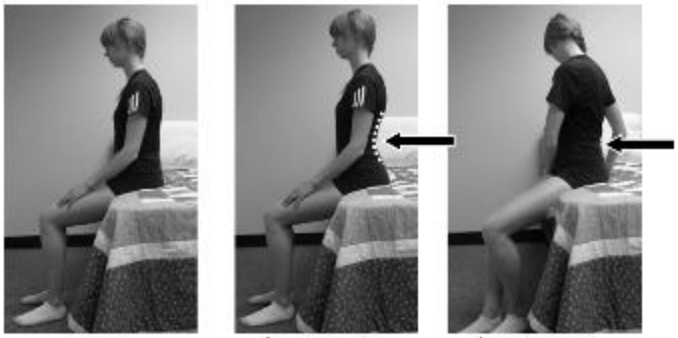

Correct | Incorrect – note arching in low back | Incorrect – note twisting in low back

**Lumbar Extension Rotation Skill: Sitting position on edge of bed**

**Do:**

- Move forward to edge of bed so feet are supported on floor
- If feet do not touch ground, place support under them (e.g. step stool), especially if you are going to be sitting on the edge of the bed for an extended period of time (left picture)

**Do NOT:** Sit with your low back arched, twisted, or shifted (right picture)

**Key Principle:** Do not let feet hang off edge of bed; do not arch, twist, or shift low back

---

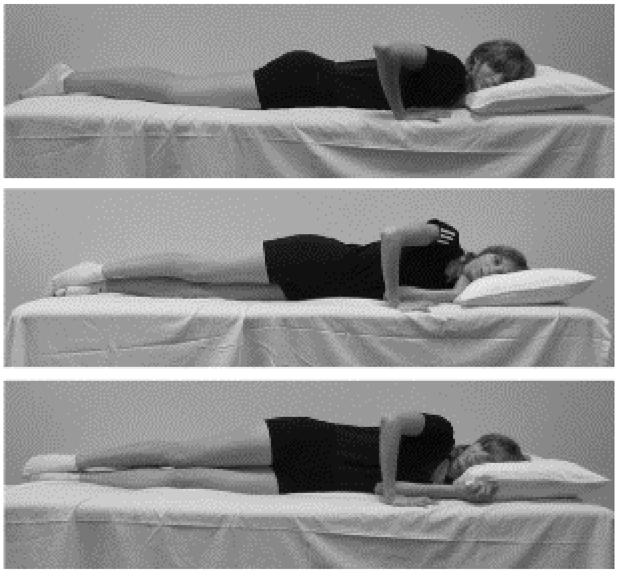

**Lumbar Extension Rotation Skill: Face lying to sit – step 1 (face lying to side lying)**

**Do:**

- Remove covers and any positioning materials
- If rolling onto left side, tuck left arm under body as much as possible

© 2020 van Dillen LR et al. *JAMA Neurology*.

- Bend right arm up so hand is by chest (top picture)
- Contract abdominals, and push right hand into surface to roll onto left side, moving body as a unit (middle and bottom pictures)

**Do NOT:** Twist upper body independently of lower body
**Key Principle:** Move body as a unit

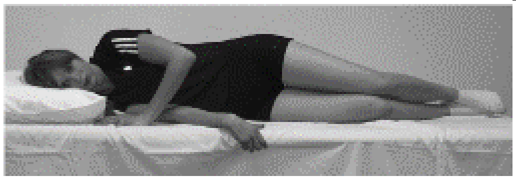

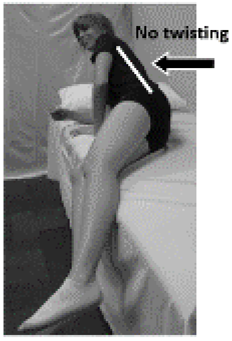

Correct

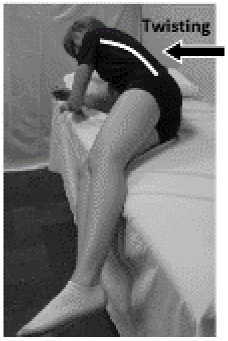

Incorrect – twisting in low back

**Lumbar Extension Rotation Skill: Face lying to sit – step 2 (side lying to sit; using top arm)**

**Do:**

- From side lying (top picture), push into surface with top arm and elbow of arm on surface, while letting legs drop over edge of surface (bottom left picture)
- Keep low back flat or slightly bent as you come to sitting
- Move forward to edge of surface so feet are positioned on floor

**Do NOT:** Twist your low back (bottom right picture)
**Key Principle:** Move trunk as a unit

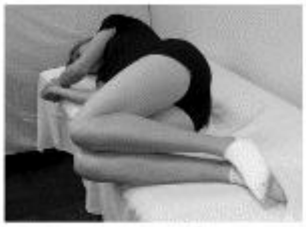

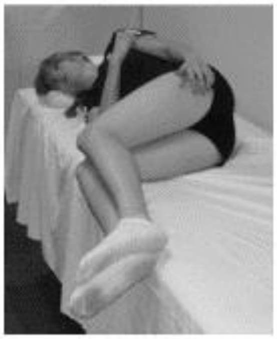

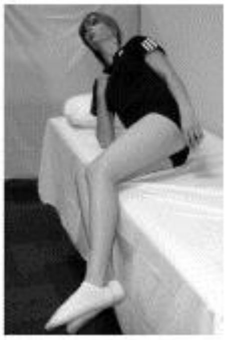

**Lumbar Extension Rotation Skill: Face lying to sit – step 2 (side lying to sit; without using top arm)**

**Do:**

© 2020 van Dillen LR et al. *JAMA Neurology*.

- From side lying (top picture), push into surface with elbow of arm on surface while letting legs drop over edge of surface (bottom pictures)
- Keep low back flat or slightly bent as you come to sitting
- Move forward to edge of surface so feet are positioned on floor

**Do NOT:**

- Twist low back
- Use top arm to help push as this will result in twisting in low back

**Key Principle:** Move trunk as a unit

---

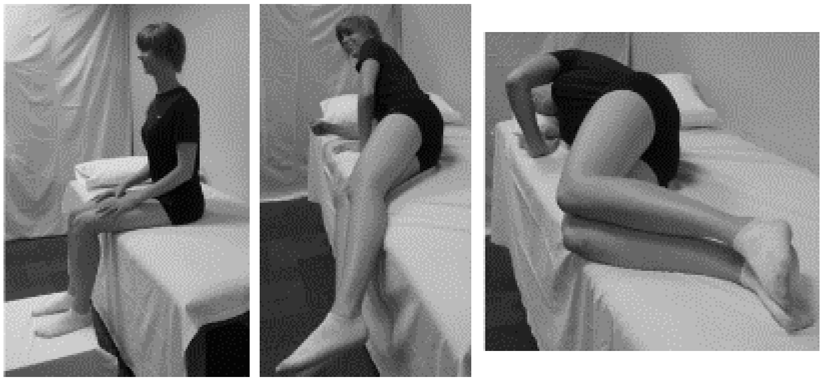

**Lumbar Extension Rotation Skill: Sit to back lying – step 1 (sit to side lying)**

**Do:**

- From sitting (left picture), lower yourself down onto elbow closest to surface (i.e. right arm if going to right side, left arm if going to left side) as you move both feet up onto the surface together (middle and right pictures)

**Do NOT:** Twist your low back

**Key Principle:** Move trunk as a unit

---

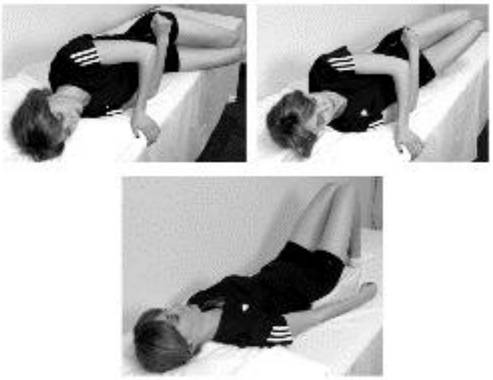

**Lumbar Extension Rotation Skill: Sit to back lying – step 2 (rolling from side lying to back lying)**

**Do:**

- From side lying, push hand of top arm, elbow of bottom arm, and lower leg into surface to log roll onto back, moving body as a unit (top pictures)
- Once on back (bottom picture), contract abdominals as you *slide* one heel down at a time
- Position on your back using materials as appropriate

**Do NOT:** Twist your upper body independently of your lower body, especially when reaching over with your top arm to push from the surface

**Key Principle:** Move body as a unit

---

© 2020 van Dillen LR et al. *JAMA Neurology*.

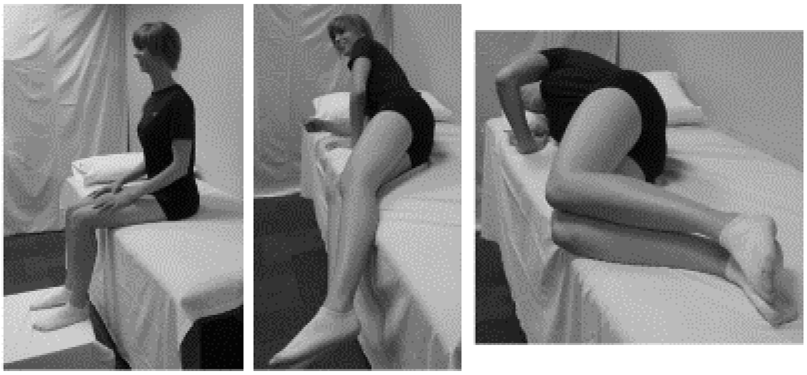

**Lumbar Extension Rotation Skill: Sit to face lying – step 1 (sit to side lying)**

**Do:**

- From sitting (left picture), lower yourself down onto elbow closest to surface (i.e. right arm if going to right side, left arm if going to left side) as you move both feet up onto surface together (middle and right pictures)

**Do NOT:** Twist your low back

**Key Principle:** Move trunk as a unit

---

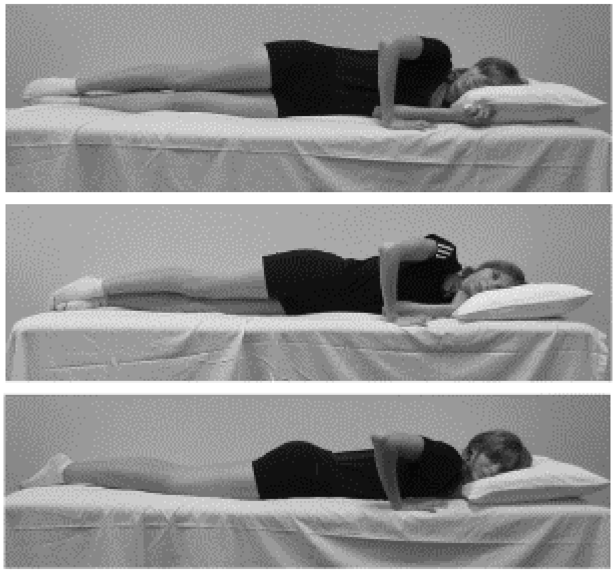

**Lumbar Extension Rotation Skill: Sit to face lying – step 2 (rolling from side lying to face lying)**

**Do:**

- Move back from edge of bed so you have room to roll onto stomach
    - Push back with top hand and side of lower leg while straightening knee
- Straighten legs
- Log roll onto stomach, moving body as a unit

**Do NOT:** Twist upper body independently of lower body

**Key Principle:** Move body as a unit

---

© 2020 van Dillen LR et al. *JAMA Neurology*.

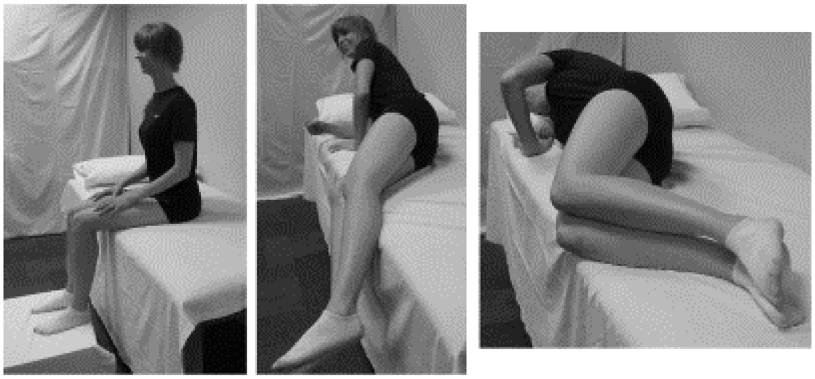

**Skill: Sit to side lying**
**Do:**
- From sitting (left picture), lower yourself down onto elbow closest to surface (i.e. right arm if going to right side, left arm if going to left side) as you move both feet up onto surface together (middle and right pictures)

**Do NOT:** Twist your low back
**Key Principle:** Move trunk as a unit

---

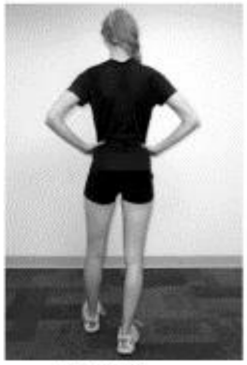

Correct

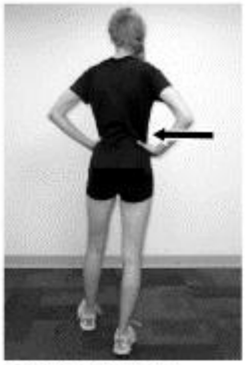

Incorrect – note twisting of the pelvis

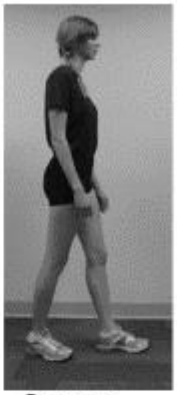

Correct – shorter step length with no twisting of pelvis or low back

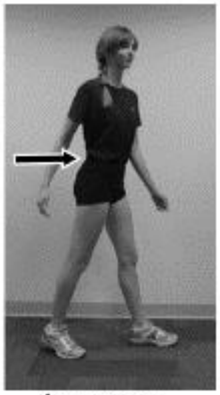

Incorrect – longer step length with twisting of pelvis and low back

**Lumbar Extension Rotation Skill: Walking**
**Do:**
- Contract abdominals to control twisting of pelvis and low back
- Place your hands on your pelvis to monitor twisting of pelvis
- Take smaller steps or slow your walking speed to help control twisting or tilting if your therapist advised you to do so

**Do NOT:** Twist or tilt your pelvis
**Key Principle:** Do not twist or tilt pelvis or low back

---

© 2020 van Dillen LR et al. *JAMA Neurology*.

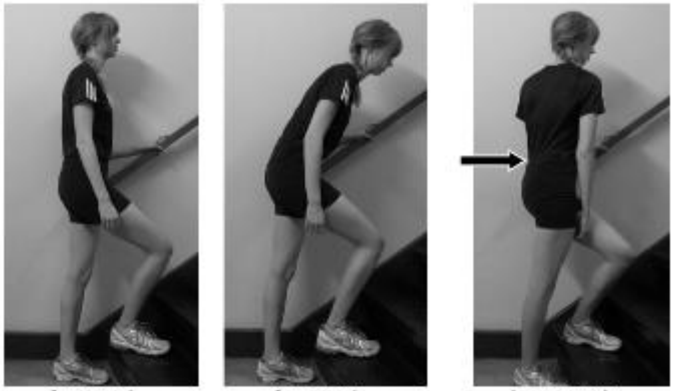

Correct stair climbing

Correct stair climbing – leaning forward

Incorrect stair climbing – note twisting in pelvis/low back

**Lumbar Extension Rotation Skill: Stair climbing – upstairs**

**Do:**

- Contract abdominals when lifting leg onto next step
- You may monitor for pelvic twisting or tilting by placing your hands on your pelvis
- You may use hand rail as needed for balance or to help decrease the load on your trunk (left picture)
- You may lean forward slightly to relieve symptoms (middle picture)

**Do NOT:** Twist or tilt your pelvis (right picture)

**Key Principle:** Do not twist or tilt pelvis/low back

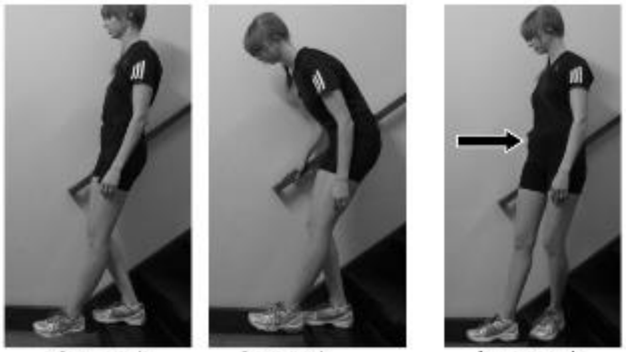

Correct stair climbing

Correct stair climbing – leaning forward slightly; shoulders in line with each other

Incorrect stair climbing – note twisting in low back; shoulders not in line with each other

**Lumbar Extension Rotation Skill: Stair climbing – downstairs**

**Do:**

- Contract abdominals when lowering leg onto next step
- You may monitor for pelvic twisting or tilting by placing your hands on your pelvis
- You may use hand rail as needed for balance or to help decrease the load on your trunk (left picture)
- You may lean forward slightly to relieve symptoms (middle picture)

**Do NOT:**

- Twist or tilt your pelvis (right picture)
- Twist low back (right picture)

**Key Principle:** Do not twist or tilt pelvis/low back

© 2020 van Dillen LR et al. *JAMA Neurology*.

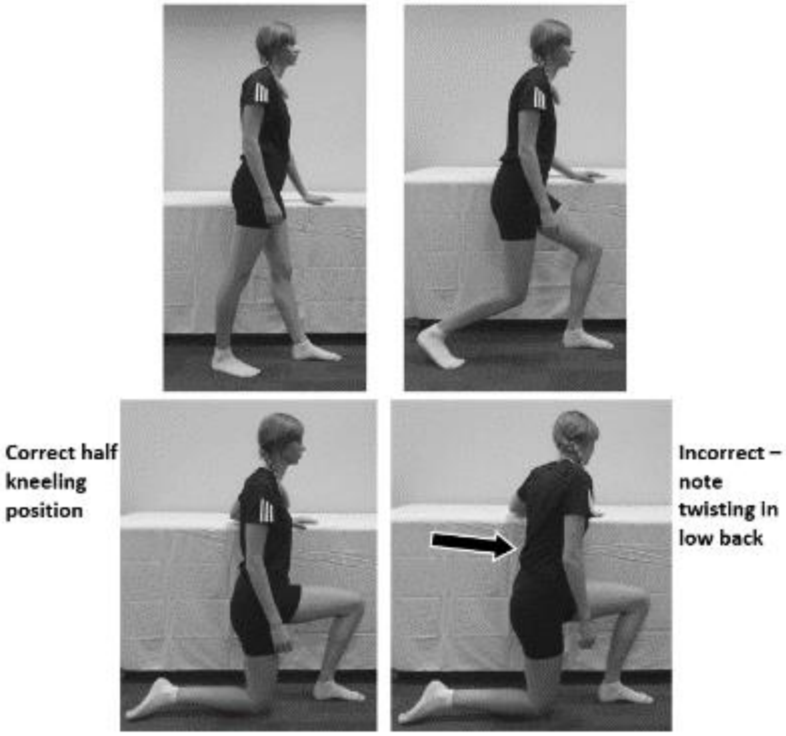


**Lumbar Extension Rotation Skill: Standing to kneeling – step 1 (standing to half kneeling)**

**Do:**

- Stand next to a surface that can be used for support
- Stand with feet in a staggered position (top left picture)
- Bend hips and knees (top right picture) to lower yourself onto one knee in a half kneeling position (bottom left picture)

**Do NOT:** Arch, twist, or shift your low back (bottom right picture)
**Key Principle:** Do not arch, twist, or shift low back

---

© 2020 van Dillen LR et al. *JAMA Neurology*.

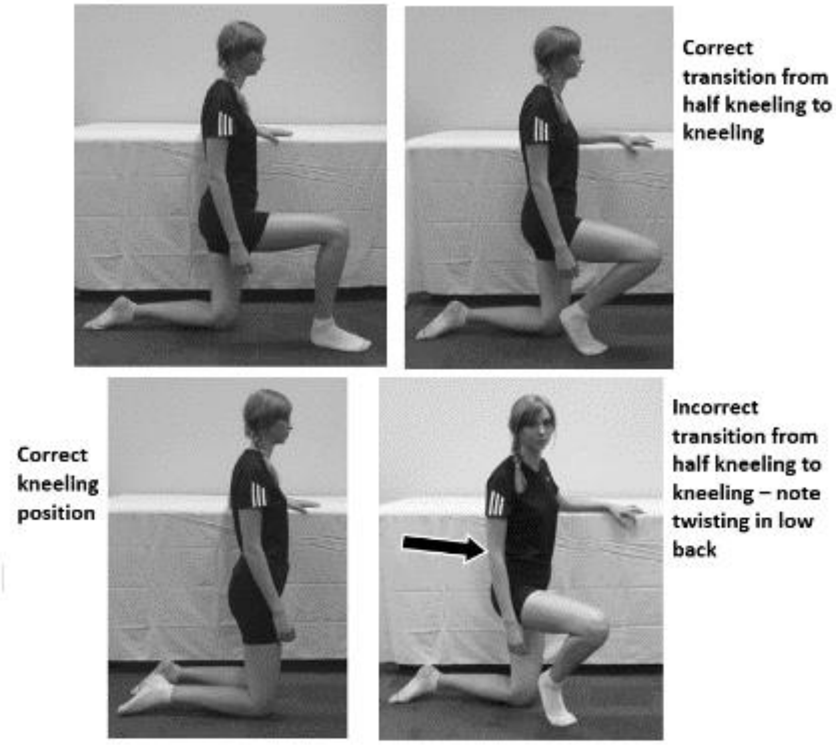


**Lumbar Extension Rotation Skill: Standing to kneeling – step 2 (half kneeling to kneeling)**

**Do:** From half kneeling (top left picture), lower onto the knee of your other leg, using support as needed (top right and bottom left pictures)

**Do NOT:** Arch, twist, or shift your low back when moving into the kneeling position (bottom right picture)

**Key Principle:** Do not arch, twist, or shift low back

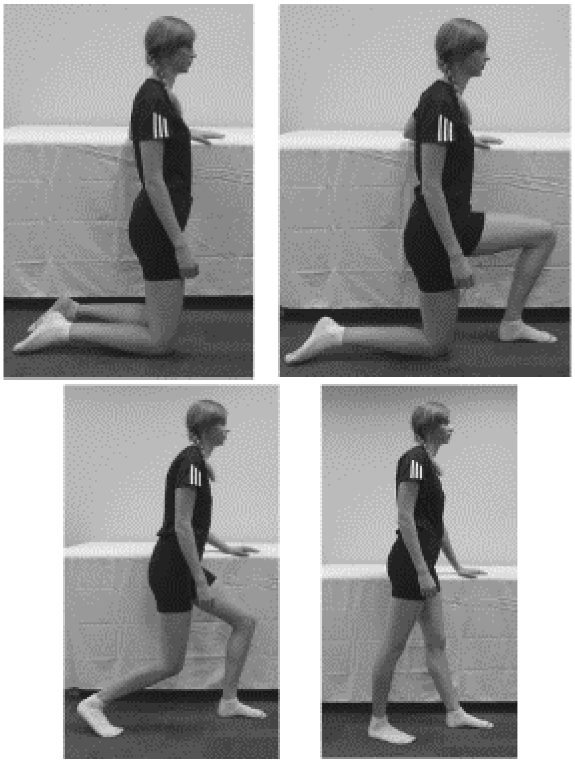

© 2020 van Dillen LR et al. *JAMA Neurology*.

**Lumbar Extension Rotation Skill: Kneeling to standing**

**Do:**

- Hold onto something for support, if available (top left picture)
- Shift weight onto one knee while bringing your other leg forward so your foot is on the ground (top right picture)
- From this position, push up to standing (bottom pictures)

**Do NOT:** Arch, twist, or shift low back, especially when bringing your leg forward to put foot on the ground

**Key Principle:** Do not arch, twist, or shift low back

---

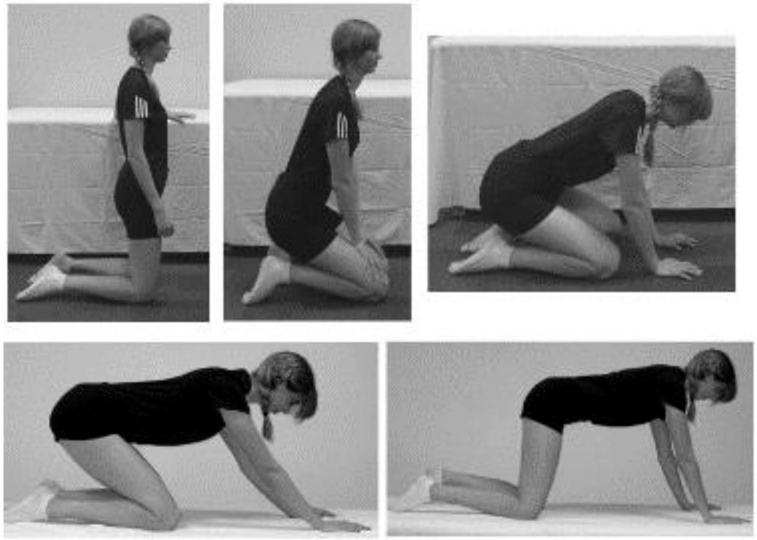

Correct sliding forward

Correct quadruped positioning

**Lumbar Extension Rotation Skill: Kneeling to quadruped**

**Do:**

- From kneeling (top left picture), bend hips and knees so you are sitting back on your heels (top middle picture)
- Lean forward, by bending at hips, to place hands on floor (top right picture)
- Slide hands forward (bottom left picture) with weight evenly distributed between both hands and knees (bottom right picture)

**Do NOT:** Arch, twist, or shift low back, especially when sitting back on your heels and when leaning forward to place hands on floor and sliding forward into the quadruped position

**Key Principle:** Do not arch, twist, or shift low back

---

© 2020 van Dillen LR et al. *JAMA Neurology*.

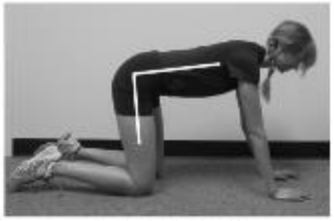

Correct positioning

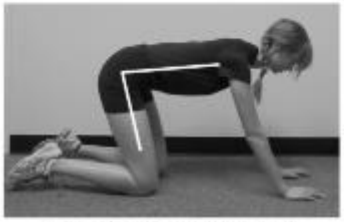

Correct positioning – weight shifted back towards heels

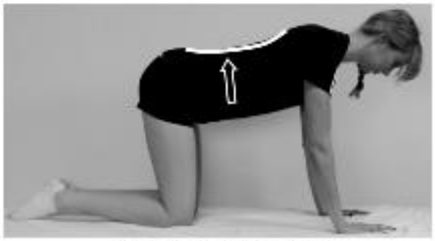

Incorrect positioning – note arching in low back

**Lumbar Extension Rotation Skill: Positioning – quadruped**

**Do:**

- Position yourself so shoulders are directly over hands, and hips are directly over knees, keeping back flat (top picture)
- Evenly distribute weight between both hands and knees
- If your therapist has advised you to do so, shift weight back towards your heels (top right picture)

**Do NOT:** Arch, twist, or shift your low back (bottom picture)

**Key Principle:** Do not arch, twist, or shift low back

---

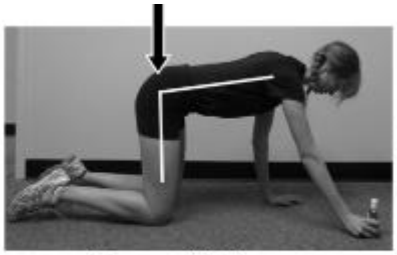

Correct positioning

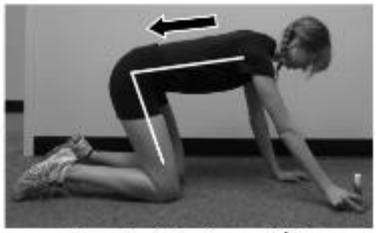

Correct positioning – weight shifted back towards heels

Incorrect positioning – note twisting in low back

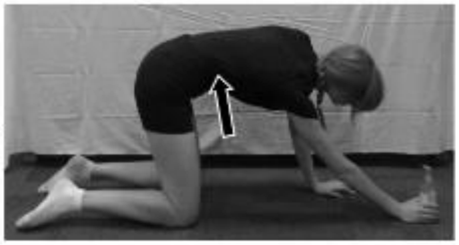

**Lumbar Extension Rotation Skill: Moving object in quadruped**

**Do:**

- If your therapist has advised you to do so, shift weight back towards your heels
- Shift weight onto one arm and leg, moving body as a unit
- Contract abdominals, then lift unweighted arm off floor to reach for object (top picture)
- Pick up object and move it as necessary

© 2020 van Dillen LR et al. *JAMA Neurology*.

- After replacing object, return hand to its original position and shift weight evenly back onto both hands and knees

**Do NOT:** Arch, twist, or shift low back when reaching for object (bottom picture)
**Key Principle:** Do not arch, twist, or shift low back

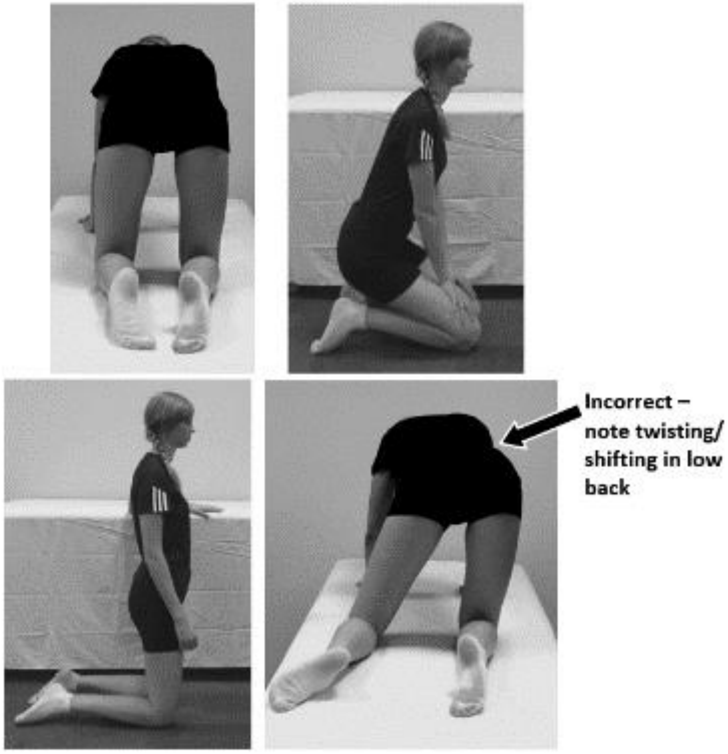


**Lumbar Extension Rotation Skill: Quadruped to standing – step 1 (quadruped to kneeling)**

**Do:**

- Push back evenly with hands (top left picture), sliding hands back and bending hips and knees to sit back on your heels (top right picture)
- Straighten hips using leg muscles to get body into full kneeling (bottom left picture)

**Do NOT:** Push back unevenly with hands, as this would result in twisting/shifting in your low back (bottom right picture)
**Key Principle:** Do not arch, twist, or shift low back

© 2020 van Dillen LR et al. *JAMA Neurology*.

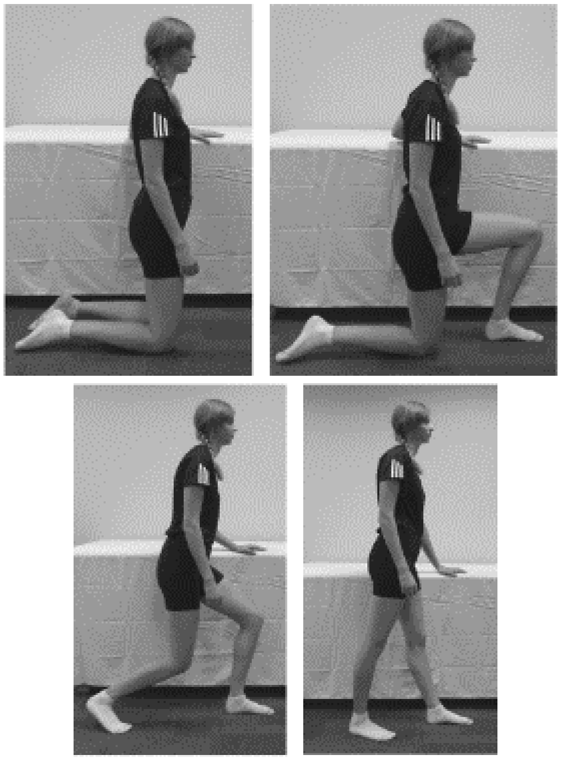

**Lumbar Extension Rotation Skill: Quadruped to standing – step 2 (kneeling to standing)**

**Do:**

- Hold onto something for support, if available (top left picture)
- Bring one leg forward so foot is on ground (top right picture)
- From this position, push up to standing, keeping low back flat (bottom pictures) or slightly bent

**Do NOT:** Arch, twist, or shift low back, especially when bringing your leg forward to put foot on the ground

**Key Principle:** Do not arch, twist, or shift low back

© 2020 van Dillen LR et al. *JAMA Neurology*.

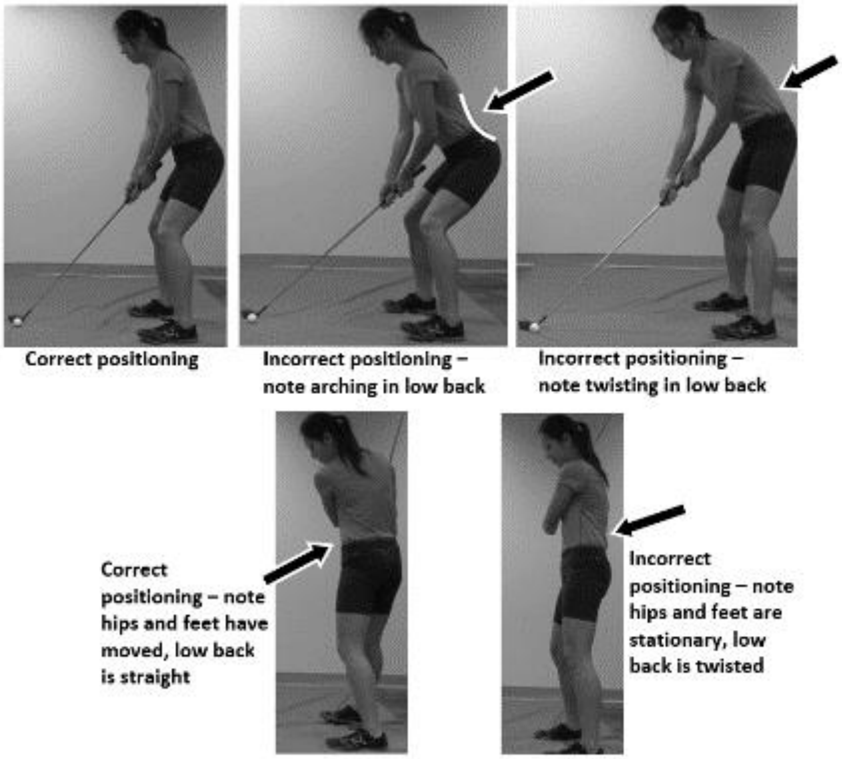


**Lumbar Extension Rotation Skill: Golf – swinging club**

**Do:**

- Keep your back straight when standing next to the ball (top left picture)
- Allow your hips and feet to move (bottom left picture)

**Do NOT:**

- Arch, twist, or shift your low back (top middle and right pictures)
- Keep your feet stationary (bottom right picture)

**Key Principle:** Do not arch, twist, or shift low back

© 2020 van Dillen LR et al. *JAMA Neurology*.

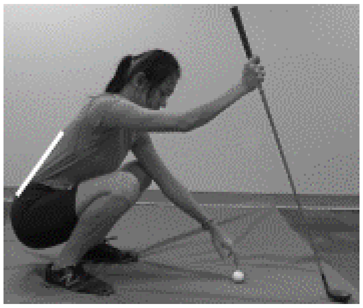
Correct positioning

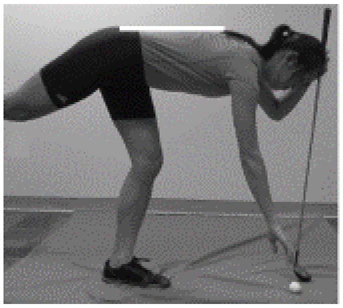
Correct positioning

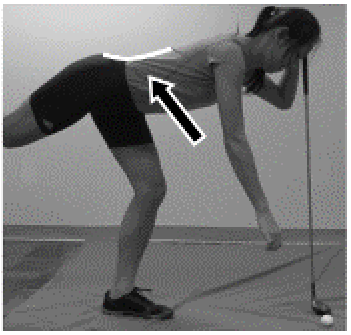
Incorrect positioning –
note arching in low back

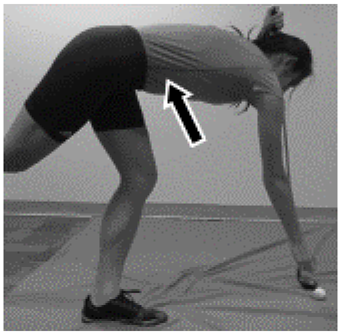
Incorrect positioning –
note twisting in low back

**Lumbar Extension Rotation Skill: Golf – picking up ball**

**Do:**

- Pick up ball by bending hips and knees (top left picture)
- You can also pick up the ball by lifting one leg up and back behind you, holding on to your golf club for support. Make sure to keep your back straight (top right picture)

**Do NOT:** Arch, twist, or shift low back (bottom pictures)

**Key Principle:** Do not arch, twist, or shift low back

© 2020 van Dillen LR et al. *JAMA Neurology*.

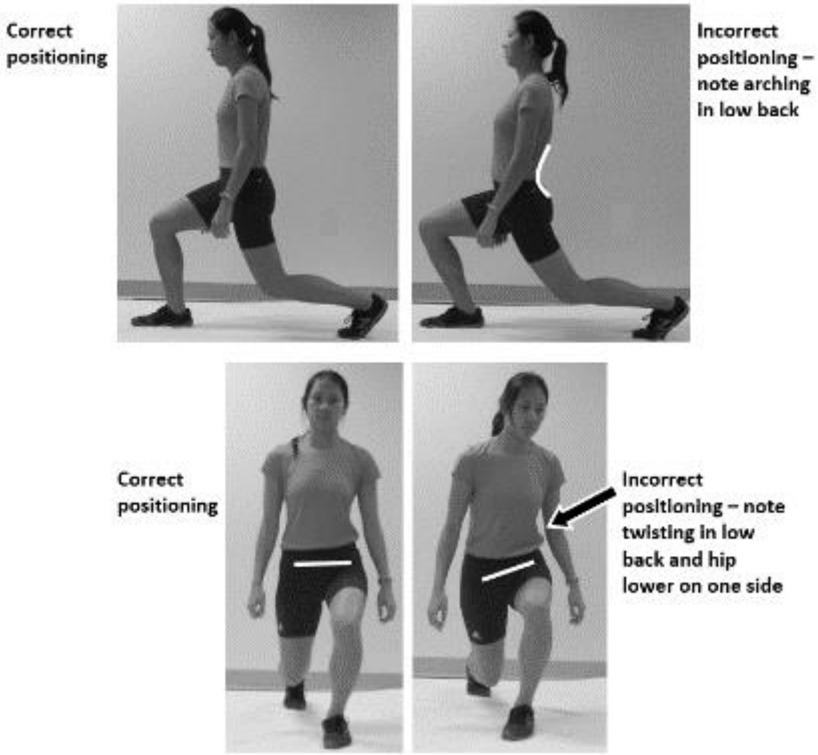


**Lumbar Extension Rotation Skill: Lunge**

**Do:**

- Keep your chest and shoulders up and in line with your hips (left pictures)
- Keep your hips level (bottom left picture)

**Do NOT:**

- Arch, twist, or shift low back (right pictures)
- Let your hip drop on one side (bottom right picture)

**Key Principle:** Do not arch, twist, or shift low back

---

© 2020 van Dillen LR et al. *JAMA Neurology*.

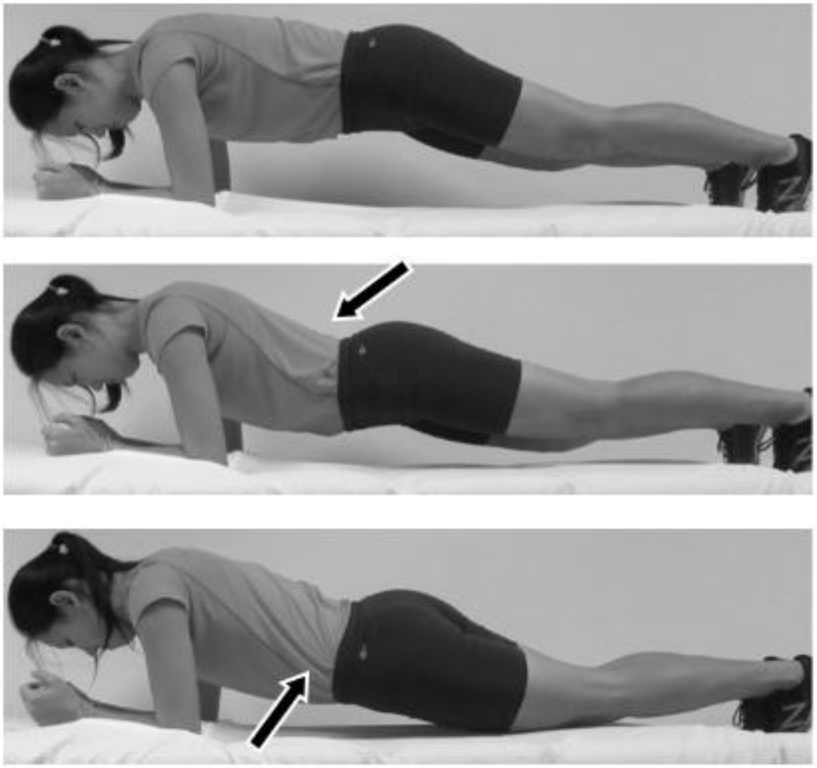

Correct positioning

Incorrect positioning – note arching in low back

Incorrect positioning – note twisting in low back

**Lumbar Extension Rotation Skill: Plank position**

**Do:** Keep your back straight and your whole body in line (top picture)

**Do NOT:**

- Arch, twist, or shift low back (middle and bottom pictures)
- Bend your hips so your buttocks are up in the air

**Key Principle:** Do not arch, twist, or shift low back

---

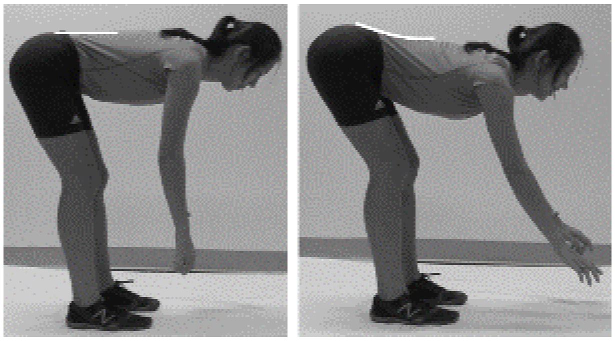

Correct positioning

Incorrect positioning – note arching in low back

**Lumbar Extension Rotation Skill: Bending over**

**Do:**

- Bend in your hips and keep your back straight (left picture)
- When you come back up from bending over, keep your back straight and use your buttock muscles to bring your hips/buttocks underneath you

**Do NOT:** Arch, twist, or shift low back (right picture)

**Key Principle:** Do not arch, twist, or shift low back

---

© 2020 van Dillen LR et al. *JAMA Neurology*.

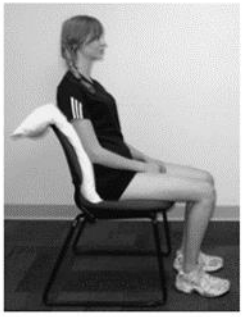
Correct

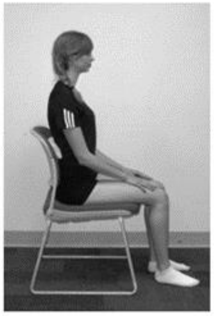
Correct

**Lumbar Flexion Rotation Skill: Sitting position – correct**

**Do:**

- Keep back well supported without any bending, twisting, or shifting
- A pillow or small folded towel may be placed behind low back to maintain a slight arch in your low back (use whatever your therapist recommends)
- Keep feet supported on the floor (if feet do not reach the floor, place something underneath feet to support them)
- Keep head and shoulders aligned over or slightly behind hips to maintain a slight arch in your low back

**Key Principle:** Do not bend, twist, or shift low back

---

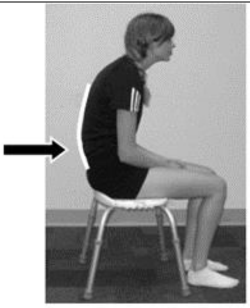
Incorrect – bending in low back

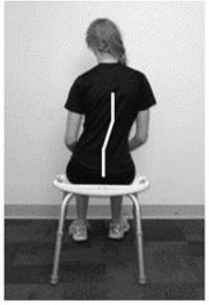
Incorrect – shift in low back

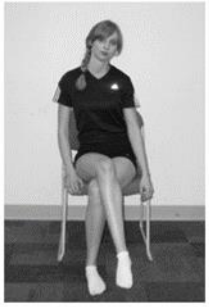
Incorrect – legs crossed

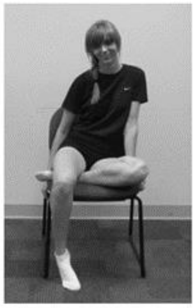
Incorrect – sitting on one leg

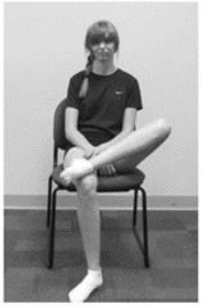
Incorrect – resting one ankle on the opposite knee

**Lumbar Flexion Rotation Skill: Sitting position – incorrect**

**Do NOT:**

- Sit with low back bent, twisted, or shifted
- Cross legs
- Sit on one leg
- Rest one ankle on the opposite knee
- Sit with knees higher than hips

---

© 2020 van Dillen LR et al. *JAMA Neurology*.

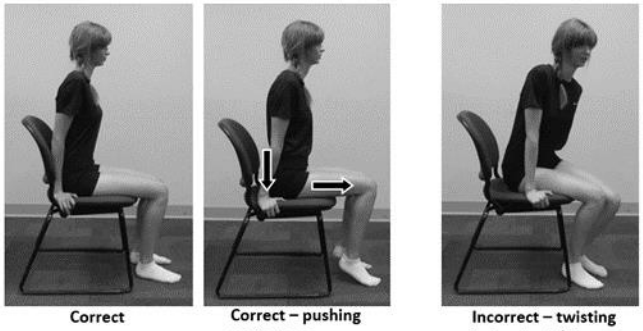

Correct | Correct – pushing up to move forward in chair | Incorrect – twisting in low back/hips

**Lumbar Flexion Rotation Skill: Sit to stand – step 1**

**Do:**

- If seated at a desk or another surface, move chair back so there is room to move forward and stand up
- Move forward in chair by pushing on the chair or armrests with both hands (left and middle pictures)
    - Keep hands behind hips

**Do NOT:** Twist low back/hips from side to side to move to edge of chair (right picture)

**Key Principle:** Do not bend or twist low back; do not twist hips

---

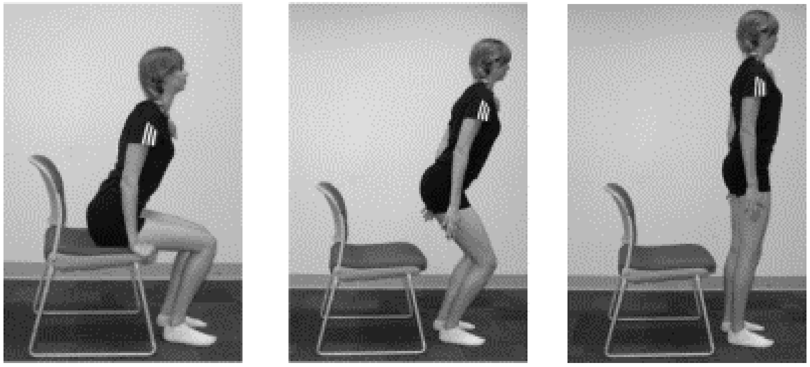

**Lumbar Flexion Rotation Skill: Sit to stand – step 2**

**Do:**

- Once at the edge of the chair, place your feet behind your knees (left picture)
- Lean forward at hips keeping back straight as you stand up from the chair (middle and right pictures)

**Do NOT:** Bend or twist your low back as you come to standing

**Key Principle:** Do not bend or twist low back

---

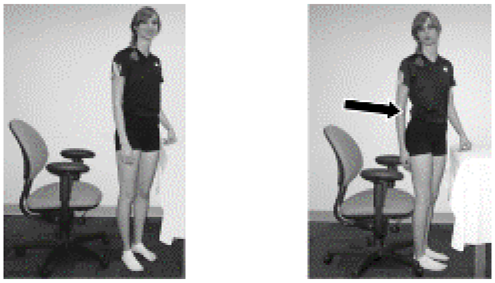

Correct | Incorrect – turning trunk with feet fixed; note twisting in low back

© 2020 van Dillen LR et al. *JAMA Neurology*.

**Lumbar Flexion Rotation Skill: Turning**

**Do:** Once in standing, turn away from the surface by stepping with each foot (left picture)
**Do NOT:** Turn your trunk with your feet fixed (right picture)
**Key Principle:** Do not twist low back

---

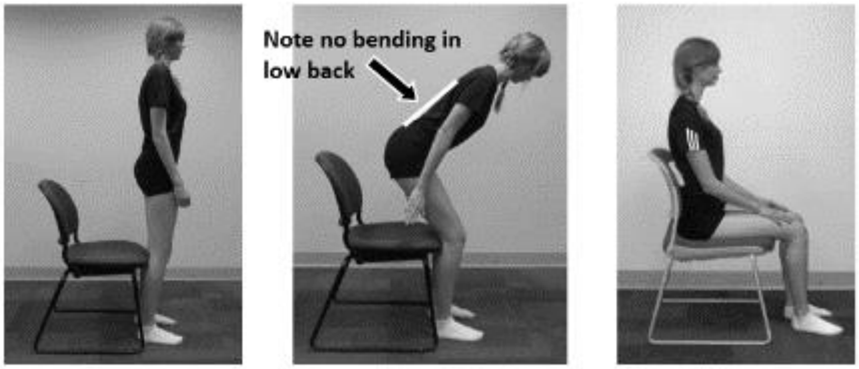


**Lumbar Flexion Rotation Skill: Stand to sit**

**Do:**

- Position chair so you are able to walk up to it and turn all the way around so the back of your legs are against the chair (left picture)
- Bend hips and knees to lower yourself into the chair without bending low back (middle picture)
- Move back in the chair and adjust position as necessary (right picture)

**Do NOT:** Bend or twist your low back as you sit down
**Key Principle:** Do not bend or twist low back

---

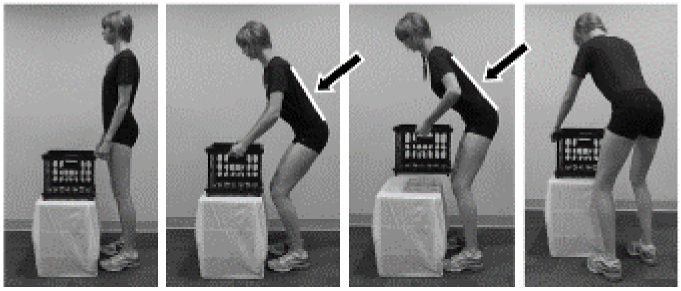

Correct distance | Correct positioning | Correct lifting | Correct positioning with item off to the side (no twisting in low back)

**Lumbar Flexion Rotation Skill: Picking up an object – correct**

**Do:**

- Stand close to object with feet shoulder width apart and the object directly in front of you (left picture)
- Keeping back straight, bend hips and knees (middle left picture)
- Easily contract abdominals without causing your low back to bend
- Pick up object and straighten your legs to lift, keeping object close to your body (middle right picture)
- If an object is located off to the side, position yourself directly behind it (right picture)

**Key Principle:** Bend in hips and knees, not in low back

---

© 2020 van Dillen LR et al. *JAMA Neurology*.

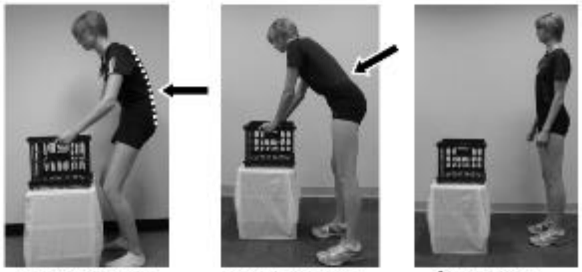

Incorrect – bending in low back | Incorrect – twisting in low back (feet facing forward, shoulders facing off to side) | Incorrect – too far from object

**Lumbar Flexion Rotation Skill: Picking up an object – incorrect**

**Do NOT:**

- Bend, twist, or shift your low back (left picture)
- Reach for an object off to the side without moving your whole body over to the object, as this would result in twisting of low back (middle picture)
- Stand too far away from the object (right picture)

---

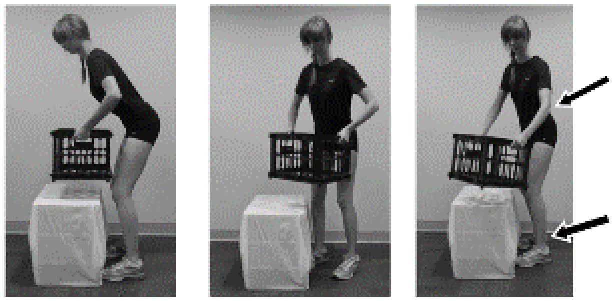

Correct | Correct | Incorrect – turning trunk with feet fixed; note twisting in low back

**Lumbar Flexion Rotation Skill: Placing object in same or different location**

**Do:**

- Keep object close to body as you lift
- Place object back in the same location by bending hips and knees (left picture)
- If object needs to be placed in a different location, move whole body by stepping with each foot (middle picture)

**Do NOT:** Turn your trunk while keeping your feet fixed (right picture)

**Key Principle:** Bend in hips and knees, not in low back; do not twist low back

---

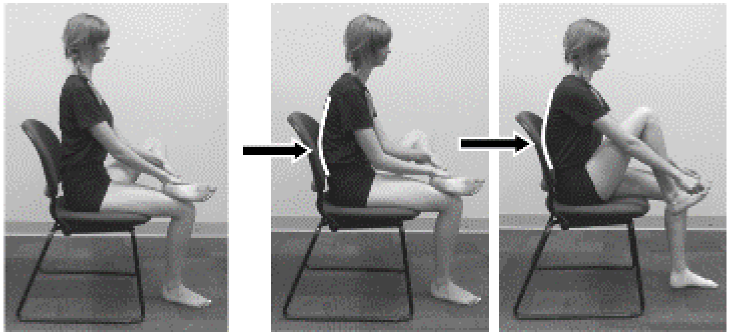

Correct | Incorrect – note bending in low back | Incorrect – note bending in low back

**Lumbar Flexion Rotation Skill: Donning a sock – initiation phase**

Do:

© 2020 van Dillen LR et al. *JAMA Neurology*.

- Bend hip and knee to lift foot up off floor
- Place foot on surface or rest it on opposite knee (left picture)

**Do NOT:** Bend or twist your low back (middle and right pictures)
**Key Principle:** Do not bend or twist low back

---

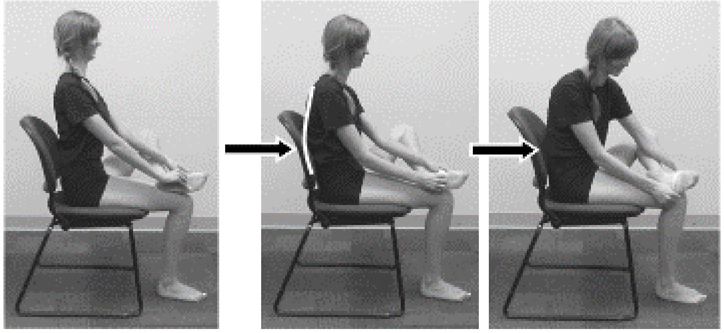

Correct | Incorrect – note bending in low back | Incorrect – note twisting in low back

**Lumbar Flexion Rotation Skill: Donning a sock – completion phase**

**Do:**

- Put sock on foot, keeping low back straight (left picture)
- Easily contract abdominals without causing your low back to bend and lower foot back to the floor

**Do NOT:** Bend or twist your low back, especially when leaning forward to put the sock on your foot (middle and right pictures)
**Key Principle:** Do not bend or twist low back

---

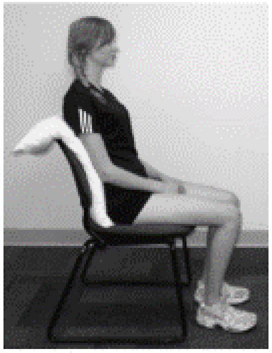

Correct

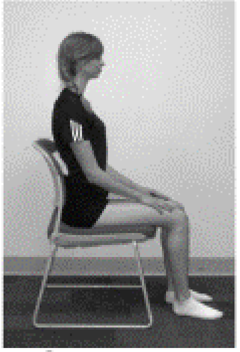

Correct

**Skill: Sitting position – correct**

**Do:**

- Keep back well supported without any bending, twisting, or shifting
- A pillow or small folded towel may be placed behind low back to maintain a slight arch in your low back (use whatever your therapist recommends)
- Keep feet supported on floor (if feet do not reach floor, place something underneath feet to support them)
- Keep head and shoulders aligned over hips to maintain a slight arch in your low back

**Key Principle:** Do not bend, twist, or shift low back

---

© 2020 van Dillen LR et al. *JAMA Neurology*.

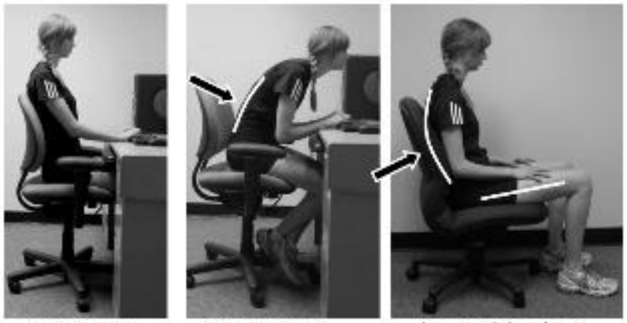

Correct sitting | Incorrect sitting – bending in low back | Incorrect sitting – knees higher than hips, resulting in bending in low back

**Lumbar Flexion Rotation Skill: Sitting position at work surface**

**Do:**

- Sit comfortably in a chair with appropriate positioning materials
- Move chair close to work surface (left picture)
    - Keep head and shoulders over hips
    - Keep back supported

**Do NOT:**

- Sit away from the surface so you are leaning forward (middle picture)
- Sit with knees higher than hips, as this will result in bending in your low back (right picture)

**Key Principle:** Do not bend, twist, or shift low back

---

**Primary work zone**: The area in which you can work when performing a sweeping motion with your arm when your elbow is bent to 90 degrees.
**Secondary work zone**: The area in which you can work when performing a sweeping motion with your arm when it is fully extended.
**Tertiary work zone**: The area that is outside of your reach when your arm is fully extended.

**Skill: Sitting position – work zones**

**Do:**

- Position items you will use repetitively within the **primary** work zone
- Position items you will use occasionally within the **secondary** work zone
- Position items you rarely use within the **tertiary** work zone
- When reaching for items in the tertiary work zone, lean forward by bending at hips, keeping back straight

**Do NOT:**

- Bend, twist, or shift your low back when reaching for items
- Keep items you use frequently too far away from you

**Key Principle:** Keep items you use often close to you

---

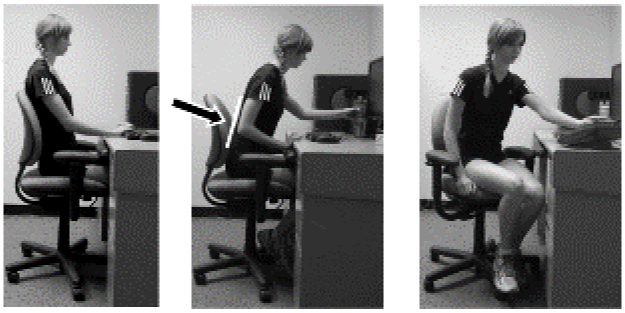

Correct sitting | Correct reaching – bending in hips and not low back | Correct reaching – turning whole body

© 2020 van Dillen LR et al. *JAMA Neurology*.

**Lumbar Flexion Rotation Skill: Manipulate/reach for objects on work surface while sitting – correct**

**Do:**

- Sit comfortably in a chair with appropriate positioning materials
- Keep items you will be using often (e.g. keyboard, mouse, etc.) within the **primary** work zone (left picture)
- When reaching for an object on work surface, lean forward by bending at hips (middle picture)
- If you need to reach for something off to the side, turn whole body to face object (right picture)

**Key Principle:** Do not bend, twist, or shift low back; bend in hips when reaching

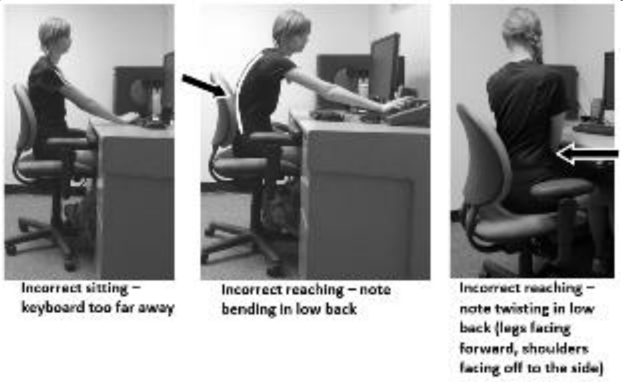


**Lumbar Flexion Rotation Skill: Manipulate/reach for objects on work surface while sitting – incorrect**

**Do NOT:**

- Keep items you use frequently far away from you
- Bend, twist, or shift in low back when reaching for an object

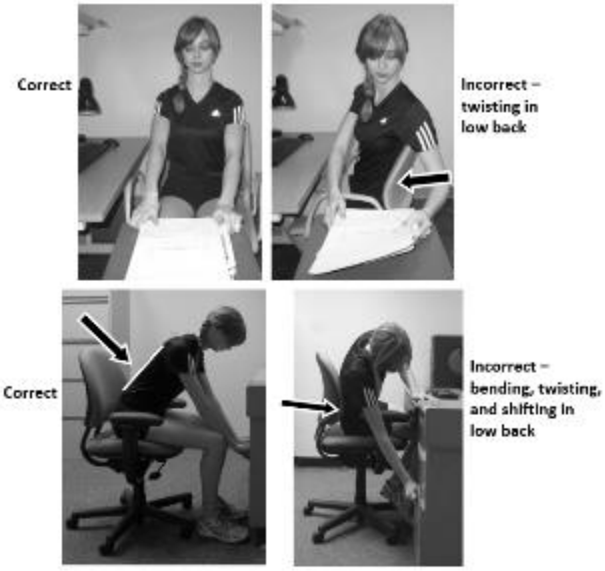


**Lumbar Flexion Rotation Skill: Manipulate/reach for objects outside of work surface while sitting**

**Do:**

- Sit comfortably in a chair with appropriate positioning materials

© 2020 van Dillen LR et al. *JAMA Neurology*.

- When reaching for something lower than work surface, turn chair or move chair to reach for the object (left pictures)
    - If chair does not swivel, turn whole body

**Do NOT:** Bend, twist, or shift in your low back to reach for an object (right pictures)
**Key Principle:** Do not bend, twist, or shift low back

---

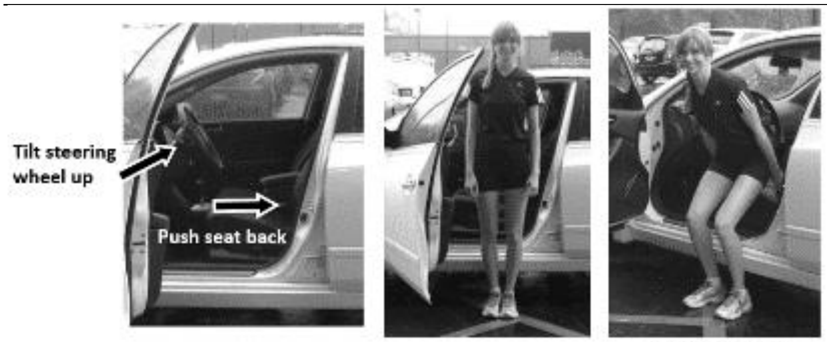


Correct – note straight back

**Lumbar Flexion Rotation Skill: Getting into car – step 1**

**Do:**

- Open door, adjust steering wheel and seat position as necessary (left picture)
- Stand facing away from car (middle picture)
- Lower yourself down to sit on the edge of the seat, keeping back straight as you sit down (right picture)
- Scoot back into the car, keeping feet supported on the ground

**Do NOT:** Bend, twist, or shift your low back
**Key Principle:** Bend in hips and knees; do not bend, twist, or shift low back

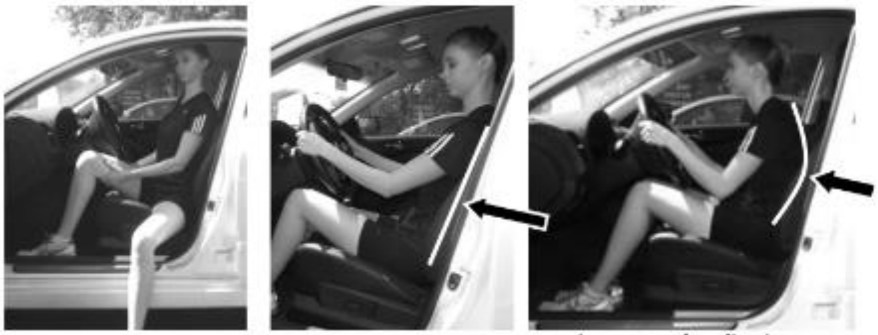

Correct

Incorrect – bending in low back

**Lumbar Flexion Rotation Skill: Getting into car – step 2**

**Do:**

- Lift each leg into car, one at a time
    - Use hands to help lift legs, if necessary (left picture)
- Turn whole body to face forward in car
- Sit comfortably in seat using appropriate positioning materials
- Adjust position of seat and/or steering wheel so back is straight (middle picture)
    - Get as close to the steering wheel as you can (elbows bent, back straight)

**Do NOT:** Lean forward or to one side, resulting in bending, twisting, or shifting of your low back (right picture)
**Key Principle:** Do not bend, twist, or shift low back

---

© 2020 van Dillen LR et al. *JAMA Neurology*.

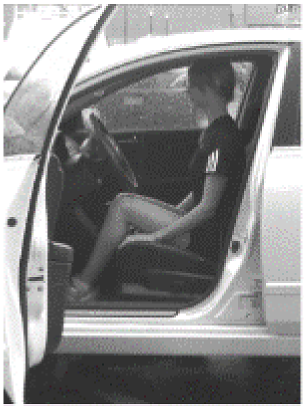

Wheel tilted up, seat pushed back

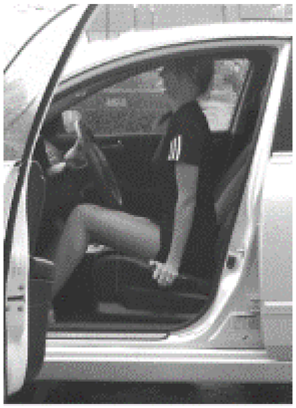

Correct – move to side by pushing up with both hands

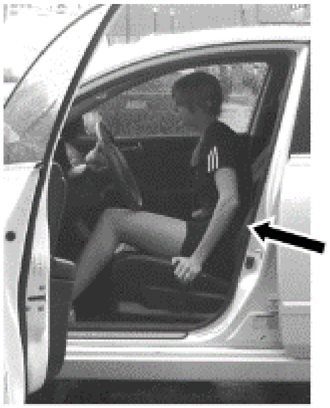

Incorrect – move to side by scooting buttocks over; note twisting in low back (shoulders not aligned over hips)

**Lumbar Flexion Rotation Skill: Getting out of car – step 1**

**Do:**

- Adjust steering wheel and seat position as necessary (left picture)
- Move to side of seat while still facing forward in car
    - Move closer to side of seat by pushing on the seat with both hands equally (middle picture)

**Do NOT:** Bend, twist, or shift your low back, especially when moving to the side of the seat (right picture)

**Key Principle:** Do not bend, twist, or shift low back

---

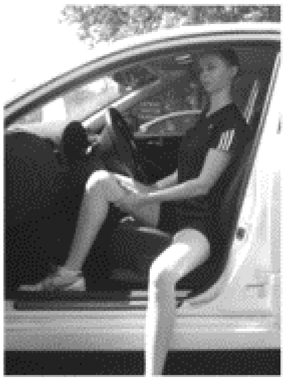

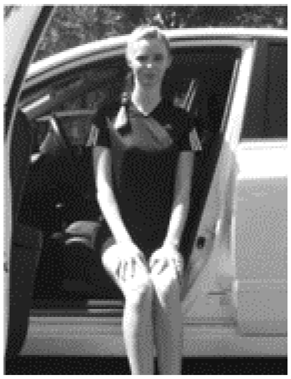

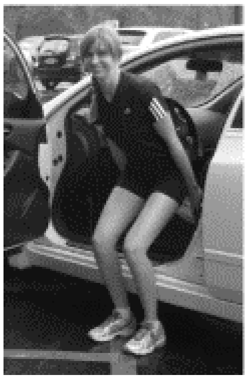

**Lumbar Flexion Rotation Skill: Getting out of car – step 2**

**Do:**

- Easily contract abdominal muscles as you lift each leg out of car, one at a time
    - Use hands to help lift legs, if necessary (left picture)
- Sit on the edge of the seat with feet on ground (middle picture)
- Lean forward by bending at hips and stand up, keeping back straight (right picture)

**Do NOT:** Bend, twist, or shift your low back

**Key Principle:** Do not bend, twist, or shift low back

---

© 2020 van Dillen LR et al. *JAMA Neurology*.

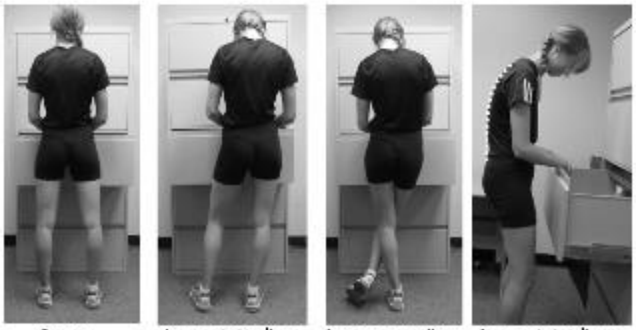

Correct standing | Incorrect standing – weight shifted onto one foot | Incorrect standing – legs crossed | Incorrect standing – slumped

**Lumbar Flexion Rotation Skill: Standing position**

**Do:**

- Stand with feet shoulder width apart, keeping weight evenly distributed over both feet (left picture)
- Stand close to the surface on which you will be working
- Surface should be at the height of your elbow for most tasks/activities

**Do NOT:**

- Stand with weight shifted onto one foot (middle left picture)
- Stand with feet/legs crossed (middle right picture)
- Hold a load in one hand or on one side on a regular basis or for an extended period of time
- Do not stand in a slumped posture (right picture)

**Key Principle:** Do not bend, twist, or shift low back

---

**Primary work zone**: The area in which you can work when performing a sweeping motion with your arm when your elbow is bent to 90 degrees.
**Secondary work zone**: The area in which you can work when performing a sweeping motion with your arm when it is fully extended.
**Tertiary work zone**: The area that is outside of your reach when your arm is fully extended.

**Lumbar Flexion Rotation Skill: Standing position – work zones**

**Do:**

- Position items you will use repetitively within the **primary** work zone
- Position items you will use occasionally within the **secondary** work zone
- Position items you rarely use within the **tertiary** work zone
- When reaching for items in the tertiary work zone, lean forward by bending at hips, keeping back straight

**Do NOT:**

- Bend, twist, or shift your low back when reaching for items
- Keep items you use frequently too far away from you

**Key Principle:** Keep items you use often close to you

---

© 2020 van Dillen LR et al. *JAMA Neurology*.

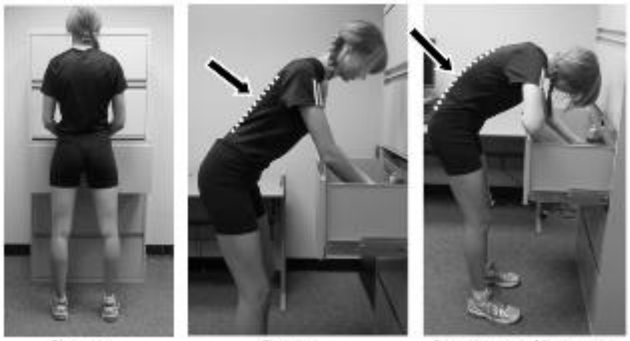

Correct standing | Correct reaching | Incorrect reaching – note bending in low back

**Lumbar Flexion Rotation Skill: Activities while standing in one place**

**Do:**

- Stand comfortably with the appropriate positioning materials (left picture)
- Keep items used often within the **primary** work zone
- When reaching for an object on the work surface, lean forward by bending at hips, keeping back straight (middle picture)
- If activity requires strength, surface should be slightly lower than elbow height
- If activity requires precision, surface should be slightly higher than elbow height

**Do NOT:** Bend, twist, or shift your low back when reaching for an object (right picture)

**Key Principle:** Bend in hips and knees; do not bend, twist, or shift low back

---

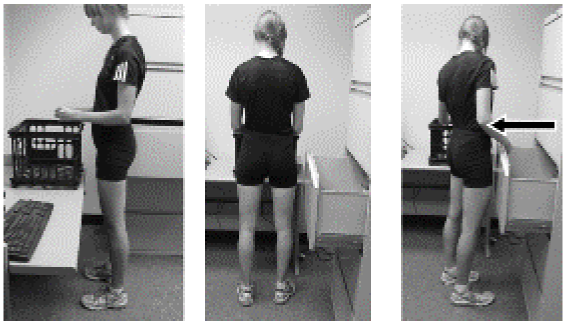

Correct reaching | Correct reaching | Incorrect reaching – note twisting in low back

**Lumbar Flexion Rotation Skill: Activities that require standing and walking**

**Do:**

- Stand comfortably with the appropriate positioning materials
- When reaching for something not directly in front of you, turn whole body to face the object (left and middle pictures)
    - Alternatively, you could side step so the object is directly in front of you
- If you need to walk to another location, step away from surface, turn whole body in the direction you need to go, and then walk

**Do NOT:** Bend, twist, or shift your low back when reaching for an object (right picture)

**Key Principle:** Do not bend, twist, or shift low back

---

© 2020 van Dillen LR et al. *JAMA Neurology*.

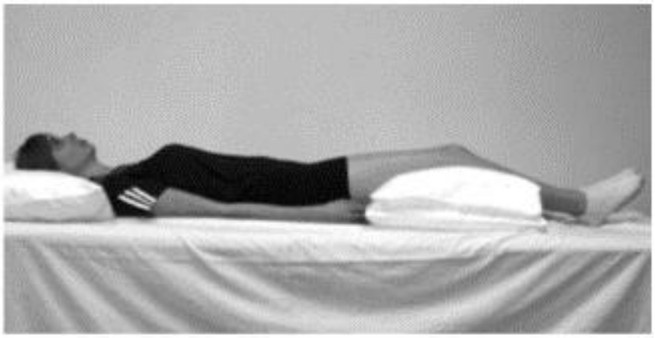

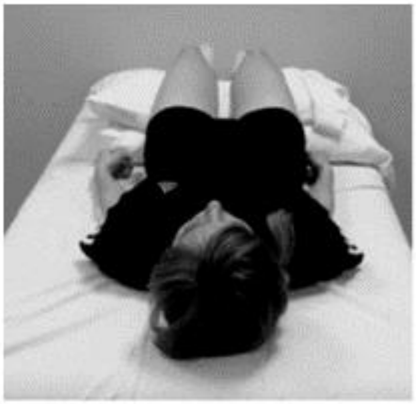

**Lumbar Flexion Rotation Skill: Positioning – back lying**

**Do:**

- Place pillows under knees to relieve pressure on low back
- Keep back straight (not twisted or shifted)
- Place a small towel under low back to keep your back slightly arched
- Practice log rolling (i.e. moving body as a unit) before going to sleep

**Key Principle:** Do not lie with low back bent, twisted, or shifted

© 2020 van Dillen LR et al. *JAMA Neurology*.

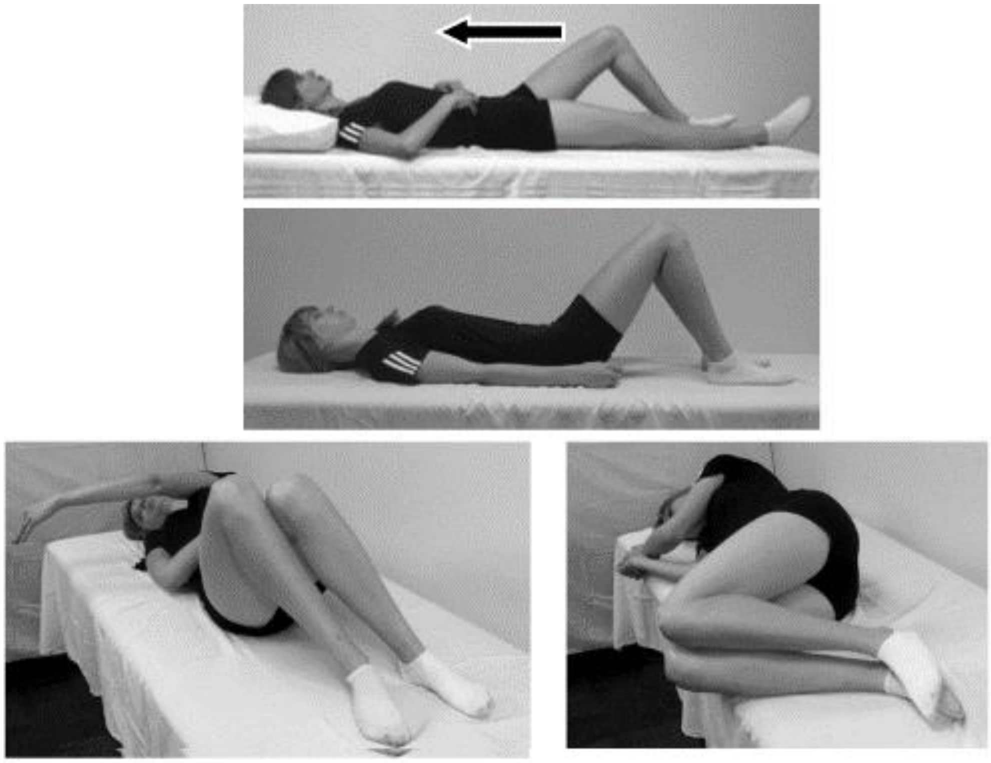

**Lumbar Flexion Rotation Skill: Rolling back lying to side lying**
**Do:**
- Remove covers and any positioning materials
- Easily contract abdominals without pushing your low back into the surface and slide each heel up one at a time (top picture) so hips and knees are bent (middle picture)
- Reach with arm and push feet into surface to log roll onto side (bottom pictures)
- Lead with arms (arms before legs; A before L), moving body as a unit

**Do NOT:** Twist upper body independently of lower body
**Key Principle:** Move body as a unit

---

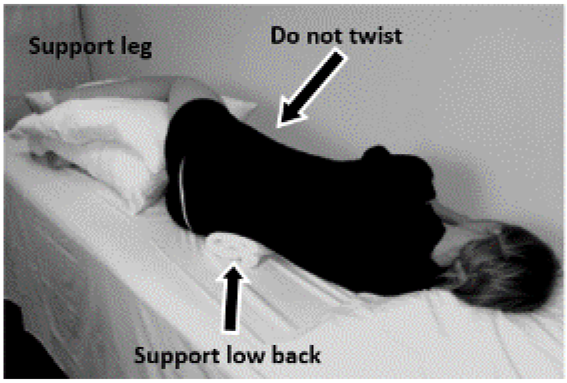


**Lumbar Flexion Rotation Skill: Positioning – side lying**
**Do:**
- Place as many pillows between knees as needed to keep pelvis in good alignment (i.e. no twisting or tilting)
- Legs should be aligned on top of each other
- Place a small folded towel at waist, just above hip
- Use a pillow to support head in a neutral position (i.e. no bending or twisting)

© 2020 van Dillen LR et al. *JAMA Neurology*.

**Do NOT:**

- Lie with low back bent, twisted, or shifted
- Lie with pelvis twisted or tilted
- Lie curled up (i.e. in fetal position), as this will result in bending in your low back

**Key Principle:** Do not lie with low back bent, twisted, or shifted

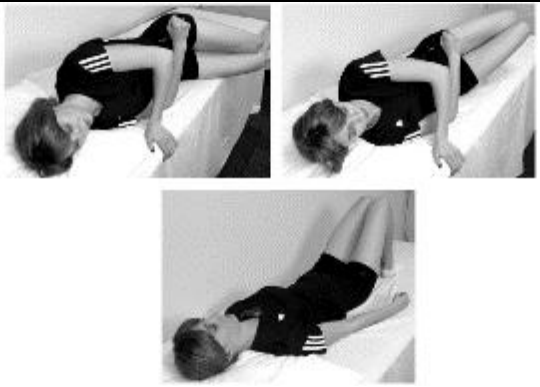

**Lumbar Flexion Rotation Skill: Rolling from side lying to back lying**

**Do:**

- Remove covers and any positioning materials
- Push hand of top arm, elbow of bottom arm, and lower leg into surface to log roll onto back, moving body as a unit (top pictures)
- Once on back (bottom picture), easily contract abdominals without pushing your low back into the surface, and slide one heel down at a time
- Position in back lying using materials as appropriate

**Do NOT:** Twist your upper body independently of your lower body, especially when reaching to push from surface with top arm

**Key Principle:** Move body as a unit

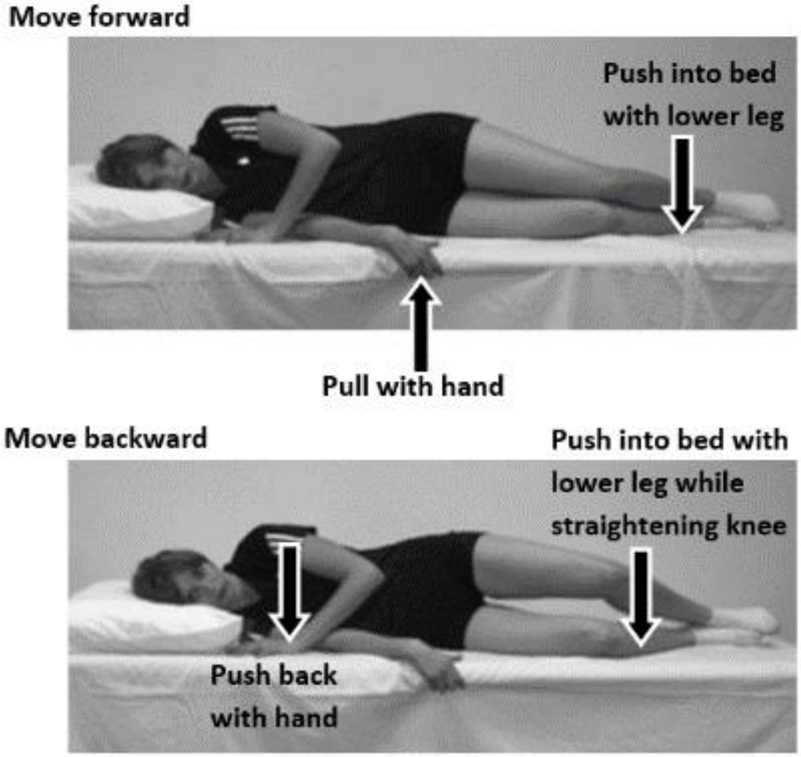


**Lumbar Flexion Rotation Skill: Moving in bed – side lying**

© 2020 van Dillen LR et al. *JAMA Neurology*.

**Do:**

- Remove covers and any positioning materials
- To move forward (top picture)
  - Pull on the side of the bed with bottom hand and push into the bed with lower leg
- To move backward (bottom picture)
  - Push back with top hand and side of lower leg while straightening knee

**Do NOT:** Bend, twist, or shift your low back
**Key Principle:** Do not bend, twist, or shift low back

---

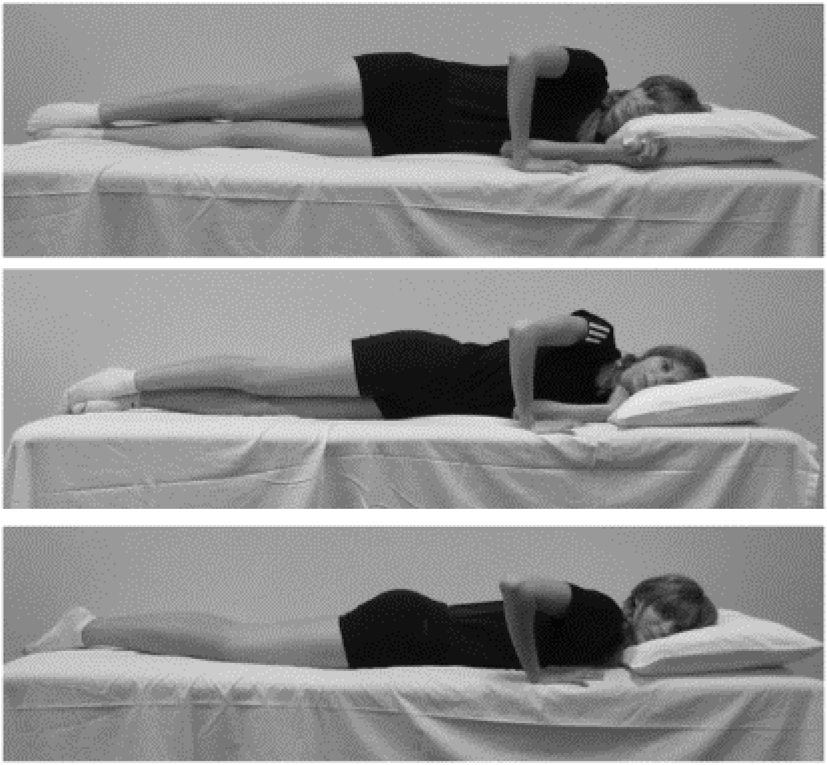

**Lumbar Flexion Rotation Skill: Rolling from side lying to face lying**

**Do:**

- Remove covers and any positioning materials
- Straighten legs (top picture)
- Log roll onto stomach, moving body as a unit (middle and bottom pictures)

**Do NOT:** Twist upper body independently of lower body
**Key Principle:** Move body as a unit

---

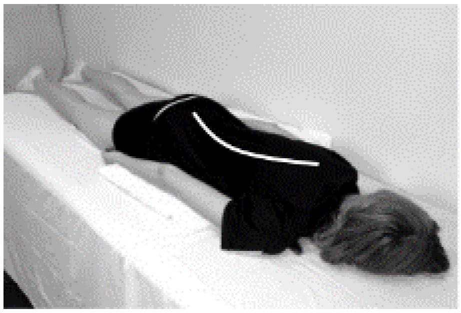

Correct position

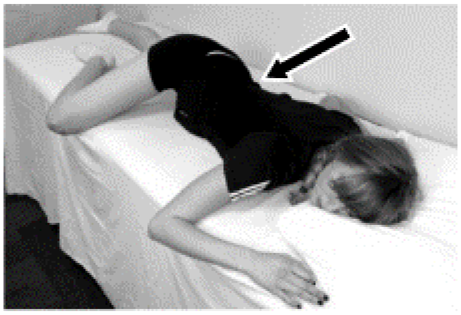

Incorrect position – hip and knee bent; note twisting in low back

**Lumbar Flexion Rotation Skill: Positioning – face lying**

© 2020 van Dillen LR et al. *JAMA Neurology*.

**Do:** Lie on your stomach while maintaining a slight arch in your back
**Do NOT:** Lie with one hip and knee bent resulting in twisting in your low back
**Key Principle:** Do not lie with low back twisted or shifted

---

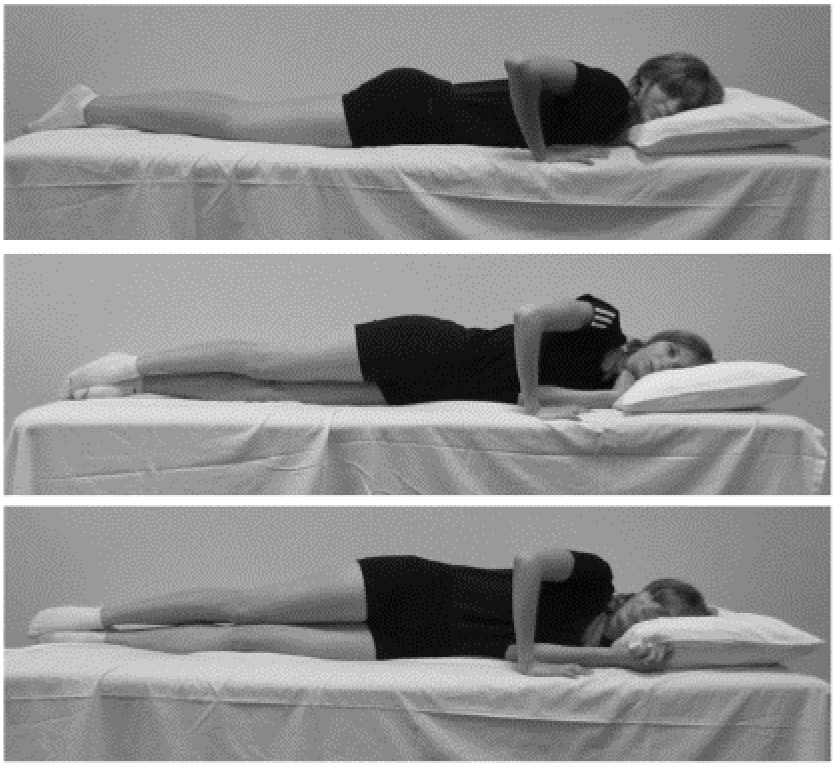

**Lumbar Flexion Rotation Skill: Rolling from face lying to side lying**
**Do:**

- Remove covers and any positioning materials
- If rolling onto left side, tuck left arm under body as much as possible
- Bend right arm up so hand is by chest (top picture)
- Easily contract abdominals without causing your low back to bend, and push right hand into surface to roll onto left side, moving body as a unit (middle and bottom pictures)
- Position in side lying with appropriate materials

**Do NOT:** Twist upper body independently of lower body
**Key Principle:** Move body as a unit

---

© 2020 van Dillen LR et al. *JAMA Neurology*.

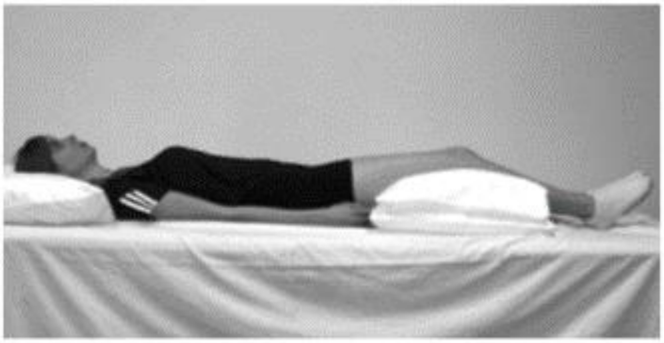

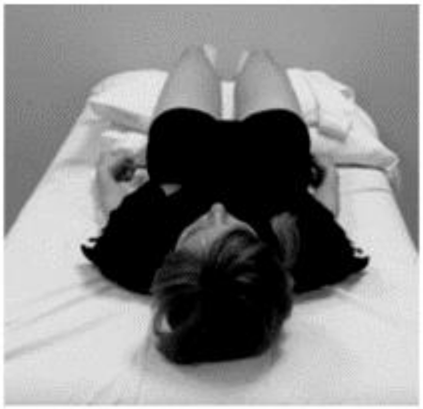

**Lumbar Flexion Rotation Skill: Positioning – back lying**

**Do:**

- Place pillows under knees to relieve pressure on low back
- Keep back straight (not twisted or shifted)
- Place a small towel under low back to keep your back slightly arched
- Practice log rolling (i.e. moving body as a unit) before going to sleep

**Key Principle:** Do not lie with low back bent, twisted, or shifted

© 2020 van Dillen LR et al. *JAMA Neurology*.

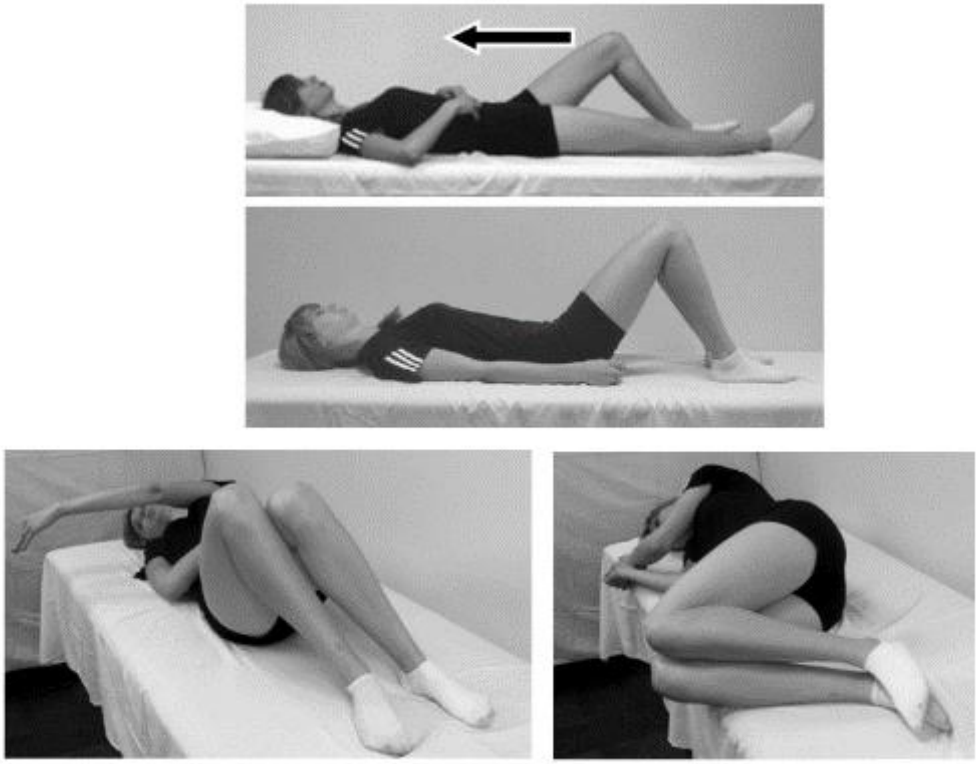

**Lumbar Flexion Rotation Skill: Back lying to sit – step 1 (back lying to side lying)**

**Do:**

- Remove covers and any positioning materials
- Easily contract abdominals without pushing your low back into the surface, and slide each heel up one at a time (top picture) so hips and knees are bent (middle picture)
- Reach with arm and push feet into surface to log roll to side (bottom pictures)
- Lead with arms (arms before legs; A before L), moving body as a unit

**Do NOT:** Twist upper body independently of lower body

**Key Principle:** Move body as a unit

© 2020 van Dillen LR et al. *JAMA Neurology*.

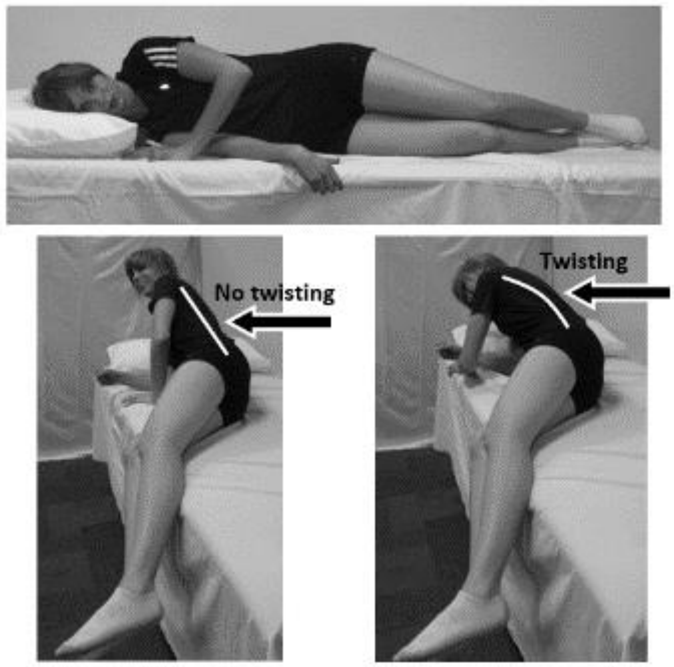


Correct

Incorrect – twisting in low back (shoulders ahead of hips)

**Lumbar Flexion Rotation Skill: Back lying to sit – step 2 (side lying to sit; using top arm)**

**Do:**

- From side lying (top picture), push into surface with top arm and elbow of the arm that is on the surface, while letting legs drop over edge of surface (bottom left picture)
- Keep low back straight as you come to sitting
- Move forward to edge of surface so feet are positioned on floor

**Do NOT:** Twist your low back, especially when reaching for the surface and pushing with your top hand (bottom right picture)

**Key Principle:** Move trunk as a unit

© 2020 van Dillen LR et al. *JAMA Neurology*.

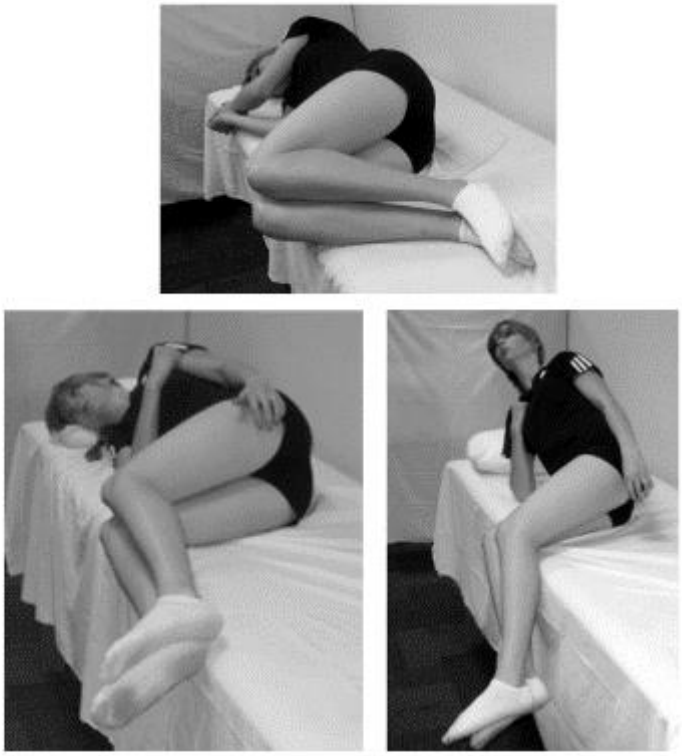

**Lumbar Flexion Rotation Skill: Back lying to sit – step 2 (side lying to sit; without using top arm)**

**Do:**

- From side lying (top picture), push into surface with elbow of the arm that is on the surface while letting legs drop over edge of surface (bottom pictures)
- Keep low back straight as you come to sitting
- Move forward to edge of surface so feet are positioned on floor

**Do NOT:**

- Bend or twist low back
- Use top arm to help push up as this will result in twisting in low back

**Key Principle:** Move trunk as a unit

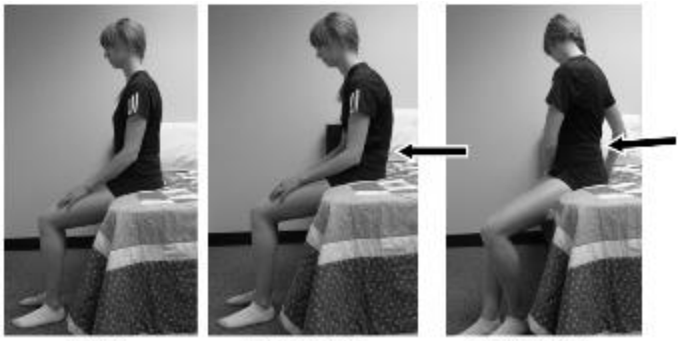

Correct | Incorrect – note bending in low back | Incorrect – note twisting in low back

**Lumbar Flexion Rotation Skill: Sitting position on edge of bed**

**Do:**

- Move forward to edge of bed so feet are supported on floor
- If feet do not touch ground, place support under them (e.g. step stool), especially if you are going to be sitting on the edge of the bed for an extended period of time (left picture)

© 2020 van Dillen LR et al. *JAMA Neurology*.

**Do NOT:** Sit with your low back bent, twisted, or shifted (middle and right pictures)
**Key Principle:** Do not bend, twist, or shift low back

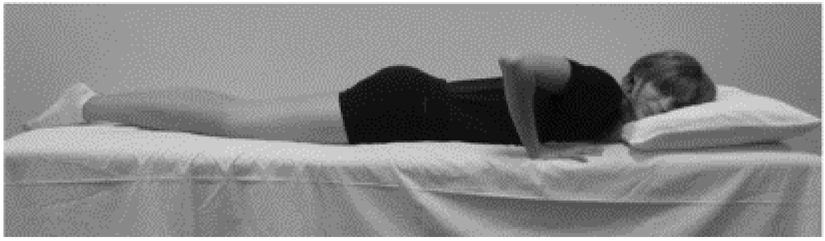

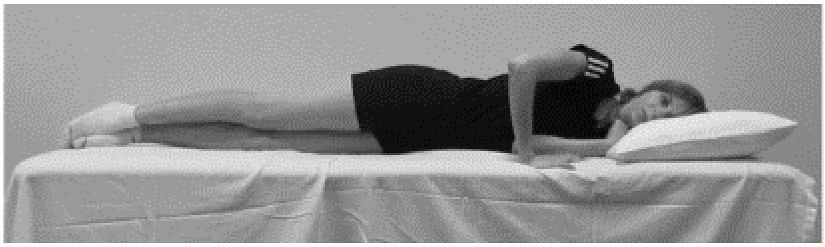

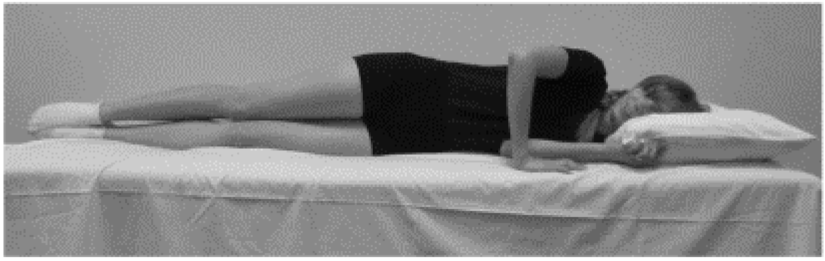

**Lumbar Flexion Rotation Skill: Face lying to sit – step 1 (face lying to side lying)**

**Do:**

- Remove covers and any positioning materials
- If rolling onto left side, tuck left arm under body as much as possible
- Bend right arm up so hand is by chest (top picture)
- Easily contract abdominals without causing your low back to bend and push right hand into surface to roll onto left side, moving body as a unit (middle and bottom pictures)

**Do NOT:** Twist upper body independently of lower body
**Key Principle:** Move body as a unit

© 2020 van Dillen LR et al. *JAMA Neurology*.

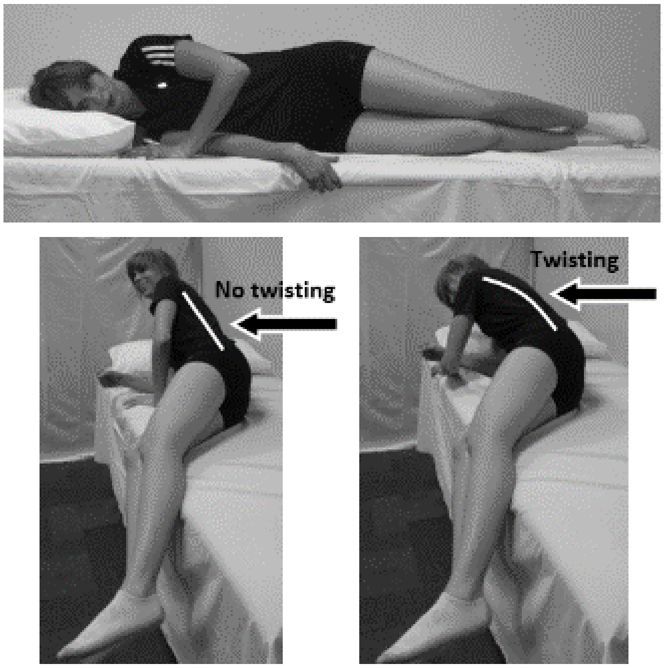


Correct

Incorrect – twisting in low back (shoulders ahead of hips)

**Lumbar Flexion Rotation Skill: Face lying to sit – step 2 (side lying to sit; using top arm)**

**Do:**

- From side lying (top picture), push into surface with top arm and elbow of the arm on the surface, while letting legs drop over edge of surface (bottom left picture)
- Keep low back straight as you come to sitting
- Move forward to edge of surface so feet are positioned on floor

**Do NOT:** Twist your low back (bottom right picture)

**Key Principle:** Move trunk as a unit

---

© 2020 van Dillen LR et al. *JAMA Neurology*.

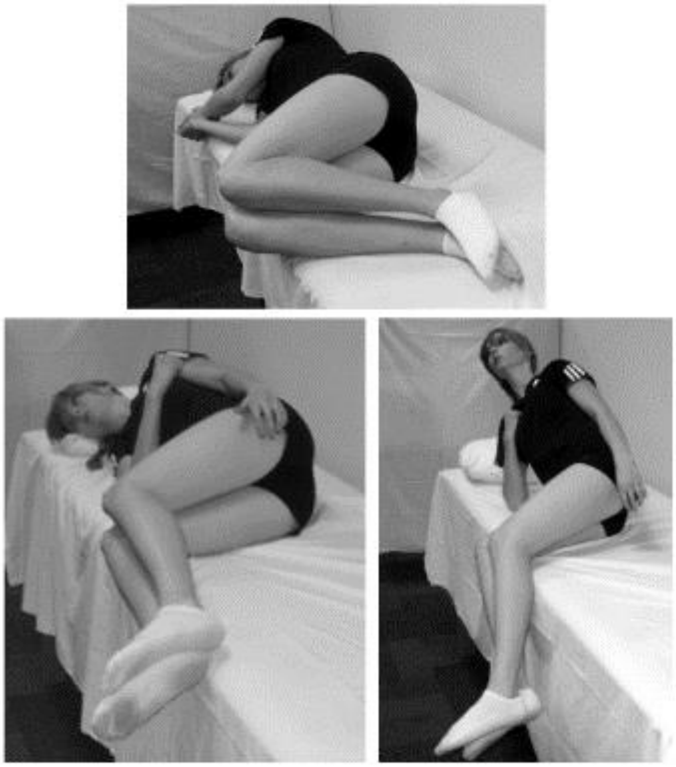

**Lumbar Flexion Rotation Skill: Face lying to sit – step 2 (side lying to sit; without using top arm)**

**Do:**

- From side lying (top picture), push into surface with elbow of the arm that is on the surface while letting legs drop over edge of surface (bottom pictures)
- Keep low back straight as you come to sitting
- Move forward to edge of surface so feet are positioned on floor

**Do NOT:**

- Twist low back
- Use top arm to help push as this will result in twisting in low back

**Key Principle:** Move trunk as a unit

© 2020 van Dillen LR et al. *JAMA Neurology*.

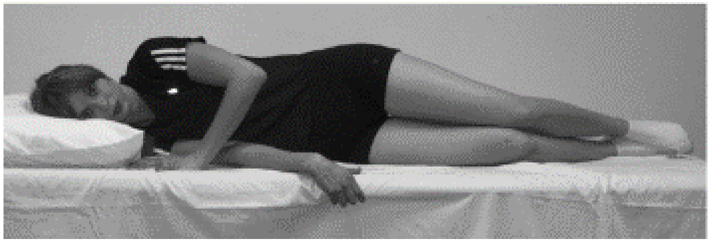

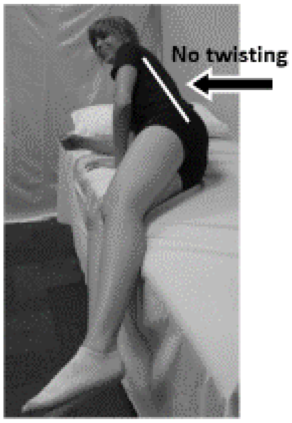

Correct

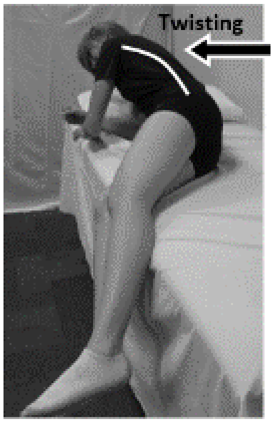

Incorrect – twisting in low back (shoulders ahead of hips)

**Lumbar Flexion Rotation Skill: Side lying to sit; using top arm**

**Do:**

- From side lying (top picture), push into surface with top arm and elbow of the arm on the surface, while letting legs drop over edge of surface (bottom left picture)
- Keep low back straight as you come to sitting
- Move forward to edge of surface so feet are positioned on floor

**Do NOT:** Bend or twist your low back (bottom right picture)

**Key Principle:** Move trunk as a unit

© 2020 van Dillen LR et al. *JAMA Neurology*.

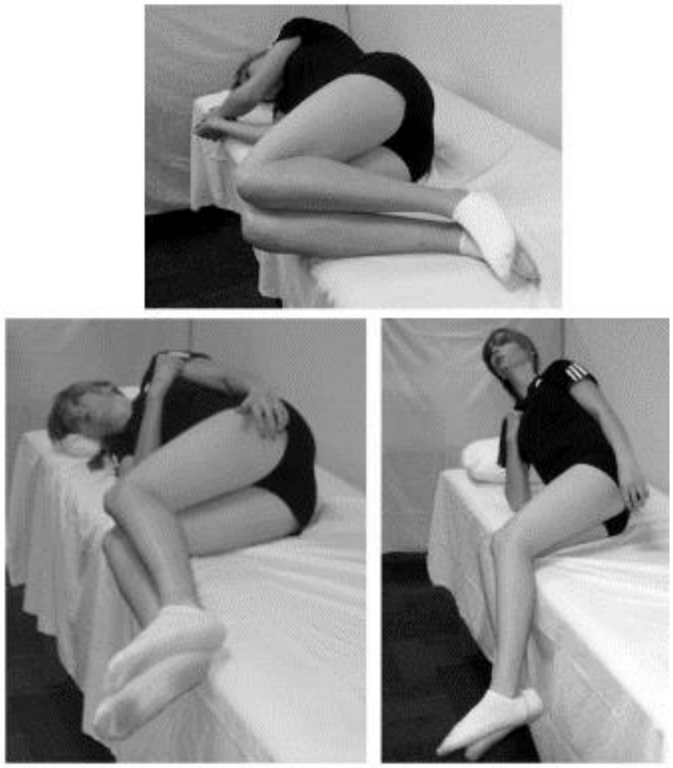

**Lumbar Flexion Rotation Skill: Side lying to sit; without using top arm**

**Do:**

- From side lying (top picture), push into surface with elbow of the arm that is on the surface while letting legs drop over edge of surface (bottom pictures)
- Keep low back straight as you come to sitting
- Move forward to edge of surface so feet are positioned on floor

**Do NOT:**

- Twist low back
- Use top arm to help push up as this will result in twisting in low back

**Key Principle:** Move trunk as a unit

---

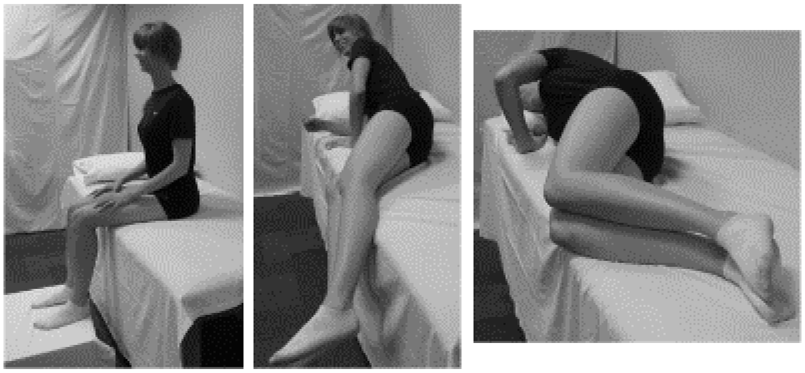

**Lumbar Flexion Rotation Skill: Sit to back lying – step 1 (sit to side lying)**

**Do:**

- From sitting (left picture), lower yourself down onto elbow closest to surface (i.e. right arm if going to right side, left arm if going to left side) as you move both feet up onto surface together (middle and right pictures)

© 2020 van Dillen LR et al. *JAMA Neurology*.

**Do NOT:** Twist your low back
**Key Principle:** Move trunk as a unit

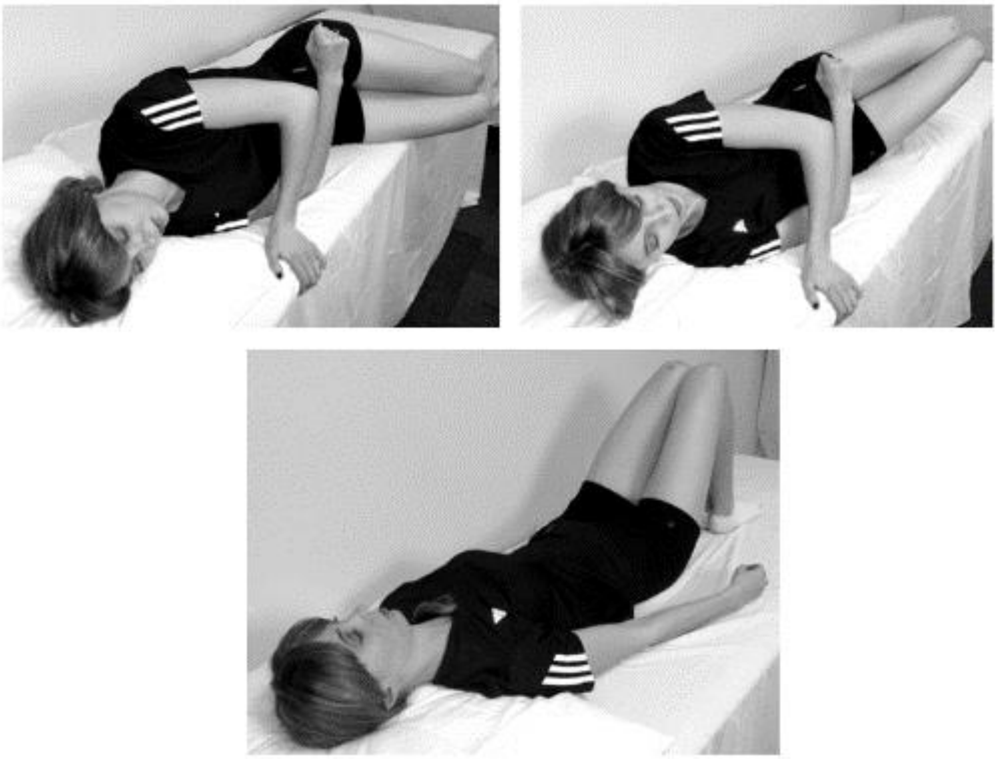

**Lumbar Flexion Rotation Skill: Sit to back lying – step 2 (rolling from side lying to back lying)**

**Do:**

- From side lying, push hand of top arm, elbow of bottom arm, and lower leg into surface to log roll onto back, moving body as a unit (top pictures)
- Once on your back (bottom picture), easily contract abdominals without pushing your low back into the surface and slide one heel down at a time
- Position on your back using materials as appropriate

**Do NOT:** Twist your upper body independently of your lower body, especially when reaching over with your top arm to push from the surface
**Key Principle:** Move body as a unit

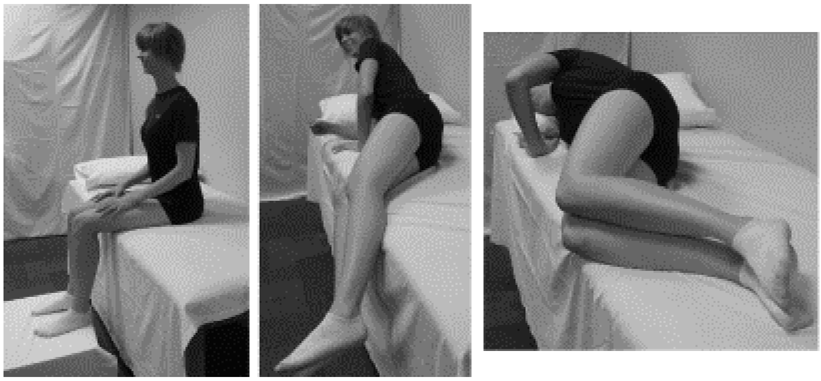

**Lumbar Flexion Rotation Skill: Sit to face lying – step 1 (sit to side lying)**

**Do:**

© 2020 van Dillen LR et al. *JAMA Neurology*.

- From sitting (left picture), lower yourself down onto elbow closest to surface (i.e. right arm if going to right side, left arm if going to left side) as you move both feet up onto surface together (middle and right pictures)

**Do NOT:** Twist your low back
**Key Principle:** Move trunk as a unit

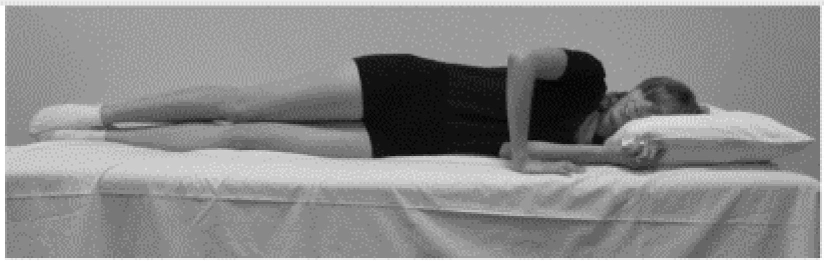

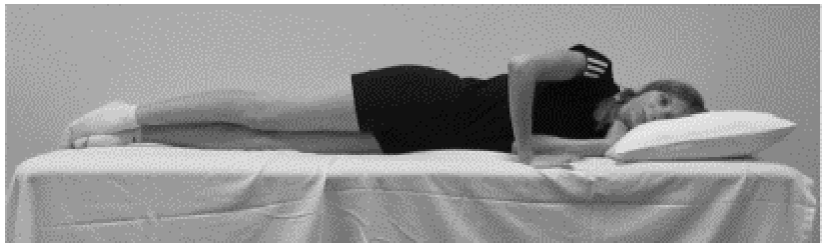

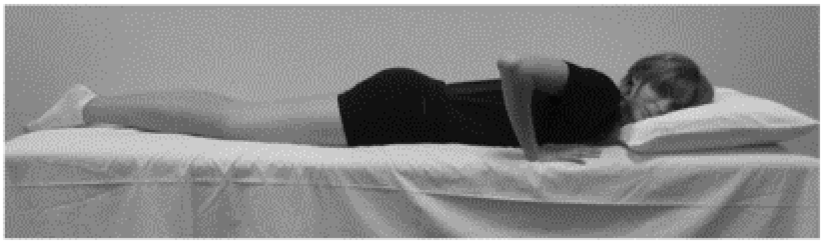

**Lumbar Flexion Rotation Skill: Sit to face lying – step 2 (rolling from side lying to face lying)**
**Do:**

- Move yourself back from edge of bed so you have room to roll onto stomach
- Straighten legs
- Log roll onto stomach, moving body as a unit

**Do NOT:** Twist upper body independently of lower body
**Key Principle:** Move body as a unit

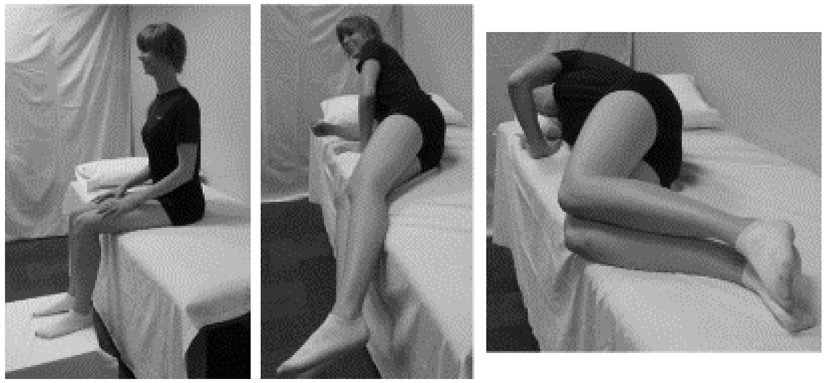

**Lumbar Flexion Rotation Skill: Sit to side lying**
**Do:**

© 2020 van Dillen LR et al. *JAMA Neurology*.

- From sitting (left picture), lower yourself down onto elbow closest to surface (i.e. right arm if going to right side, left arm if going to left side) as you move both feet up onto surface together (middle and right pictures)

**Do NOT:** Twist your low back
**Key Principle:** Move trunk as a unit

---

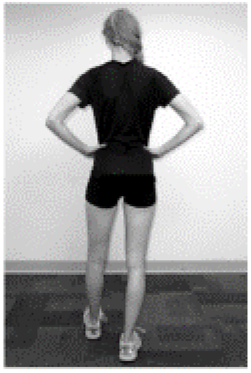
Correct

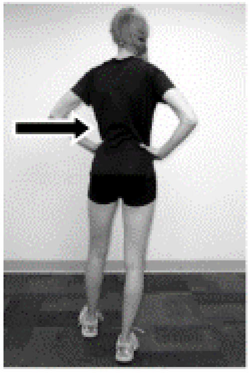
Incorrect – note twisting of the pelvis

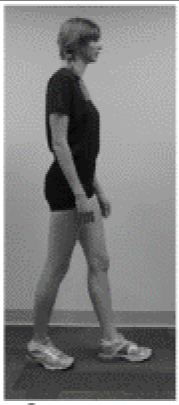
Correct – shorter step length with no twisting of pelvis or low back

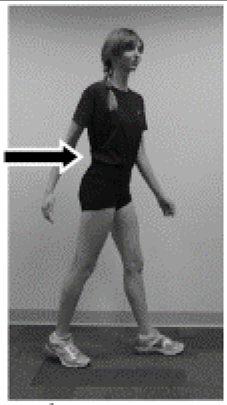
Incorrect – longer step length with twisting of pelvis and low back

**Lumbar Flexion Rotation Skill: Walking**
**Do:**

- Easily contract abdominals without causing your low back to bend to control twisting of pelvis and low back
- Place your hands on your pelvis to monitor twisting or tilting of pelvis
- Take smaller steps or slow your walking speed to help control twisting or tilting if your therapist advised you to do so

**Do NOT:**

- Twist or tilt your pelvis
- Walk with shoulders ahead of hips, i.e. bent forward
    - You can stand against a wall as a reminder of correct alignment

**Key Principle:** Do not bend forward or twist or tilt pelvis

---

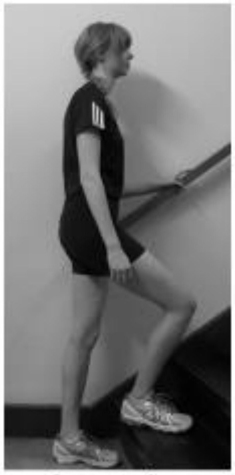
Correct stair climbing

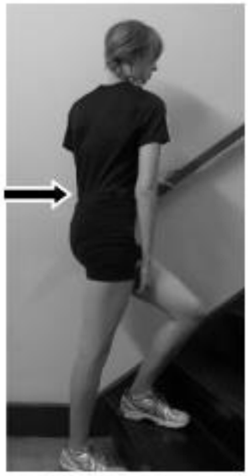
Incorrect stair climbing – note twisting in pelvis/ low back

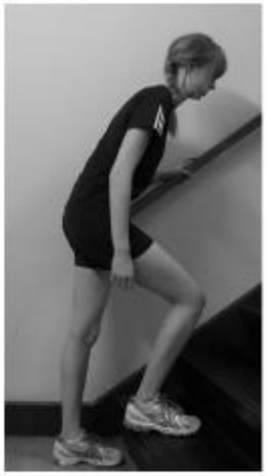
Incorrect stair climbing – note leaning forward

© 2020 van Dillen LR et al. *JAMA Neurology*.

**Lumbar Flexion Rotation Skill: Stair climbing – upstairs**

**Do:**

- Easily contract abdominals without causing your low back to bend when lifting leg onto next step
- You may monitor for pelvic twisting or tilting by placing hands on pelvis
- You may use hand rail as needed for balance or to help decrease the load on your trunk (left picture)

**Do NOT:**

- Twist or tilt pelvis (middle picture)
- Lean forward, resulting in bending in your low back (right picture)

**Key Principle:** Do not bend low back or twist or tilt pelvis

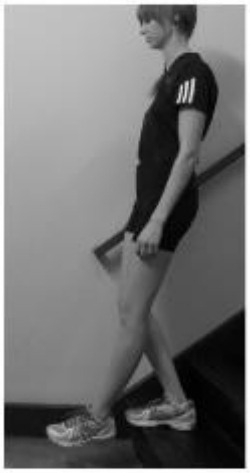

Correct stair climbing

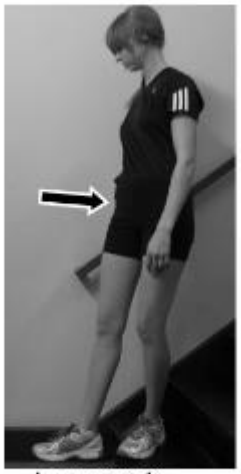

Incorrect stair climbing – note twisting in pelvis/ low back

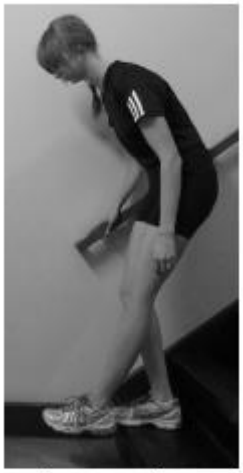

Incorrect stair climbing – note leaning forward

**Lumbar Flexion Rotation Skill: Stair climbing – downstairs**

**Do:**

- Easily contract abdominals without causing your low back to bend when lowering leg onto next step
- You may monitor for pelvic twisting or tilting by placing hands on pelvis
- You may use hand rail as needed for balance or to help decrease the load on your trunk (left picture)

**Do NOT:**

- Twist or tilt pelvis (middle picture)
- Lean forward, resulting in bending in your low back (right picture)

**Key Principle:** Do not bend low back or twist or tilt pelvis

© 2020 van Dillen LR et al. *JAMA Neurology*.

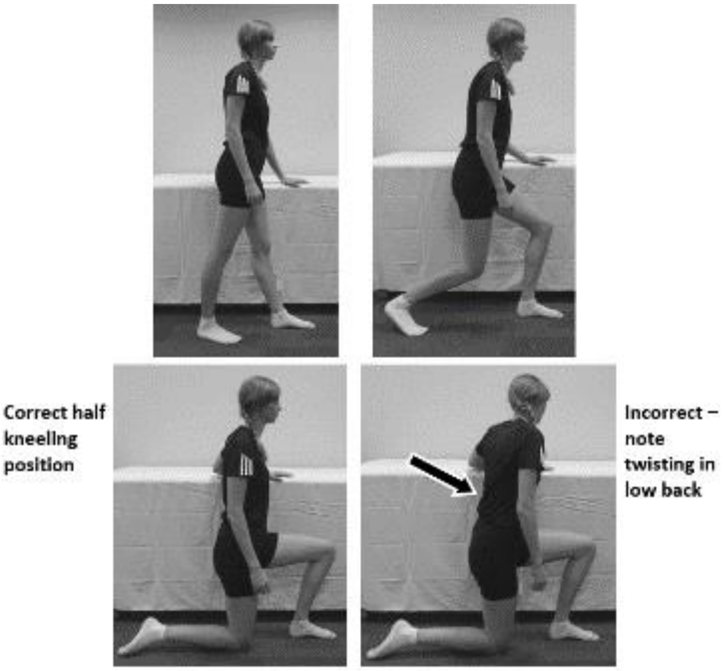


**Lumbar Flexion Rotation Skill: Standing to kneeling – step 1 (standing to half kneeling)**

**Do:**

- Stand next to a surface that can be used for support
- Stand with feet in a staggered position (top left picture)
- Bend hips and knees (top right picture) to lower yourself onto one knee in a half kneeling position (bottom left picture)

**Do NOT:** Bend, twist, or shift your low back (bottom right picture)

**Key Principle:** Do not bend, twist, or shift low back

---

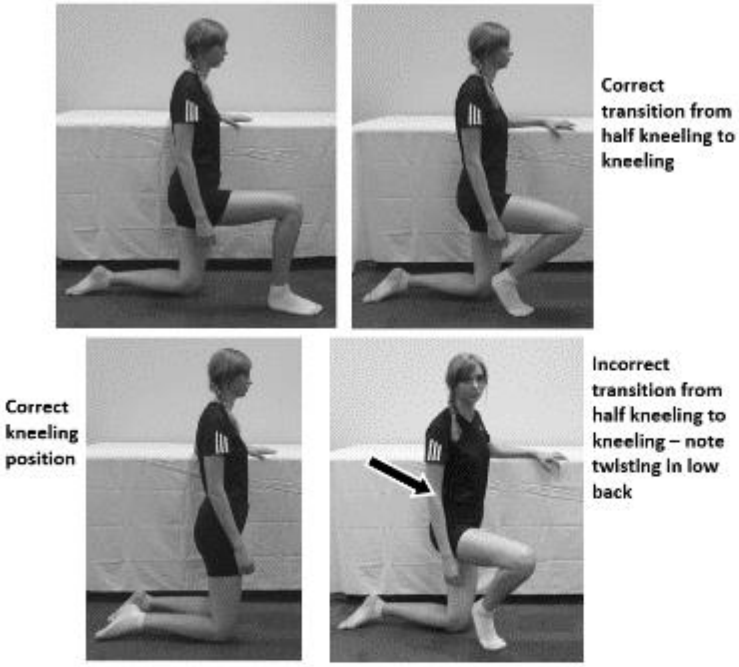


**Lumbar Flexion Rotation Skill: Standing to kneeling – step 2 (half kneeling to kneeling)**

© 2020 van Dillen LR et al. *JAMA Neurology*.

**Do:** From half kneeling (top left picture), lower onto the knee of your other leg, using support as needed (top right and bottom left pictures)
**Do NOT:** Bend, twist, or shift your low back when moving into the kneeling position (bottom right picture)
**Key Principle:** Do not bend, twist, or shift low back

---

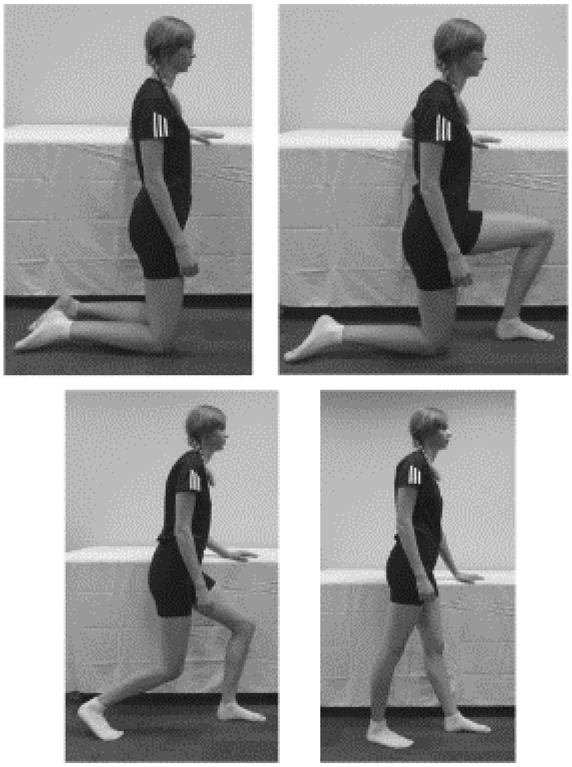

**Lumbar Flexion Rotation Skill: Kneeling to standing**

**Do:**

- Hold onto something for support, if available (top left picture)
- Shift weight onto one knee while bringing other leg forward so your foot is on ground (top right picture)
- From this position, push up to standing (bottom pictures)

**Do NOT:** Bend, twist, or shift low back, especially when bringing your leg forward to put foot on the ground
**Key Principle:** Do not bend, twist, or shift low back

---

© 2020 van Dillen LR et al. *JAMA Neurology*.

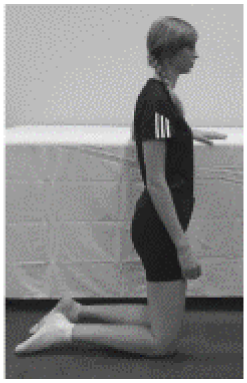

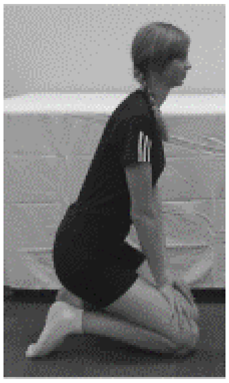

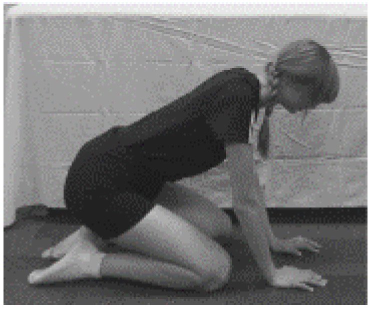

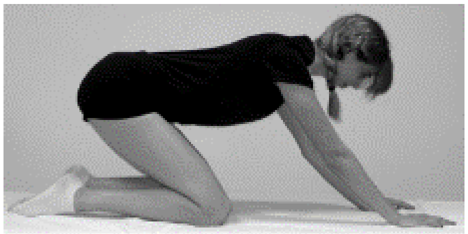

Correct sliding forward

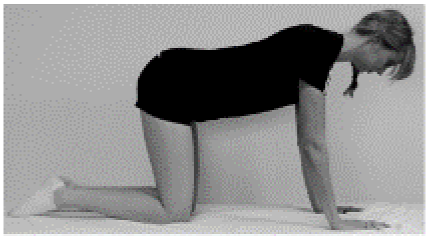

Correct quadruped positioning

**Lumbar Flexion Rotation Skill: Kneeling to quadruped**

**Do:**

- From kneeling (top left picture), bend hips and knees so you are sitting back on your heels (top middle picture)
- Lean forward, by bending at hips, to place hands on floor (top right picture)
- Slide hands forward (bottom left picture) with weight evenly distributed between both hands and knees (bottom right picture)

**Do NOT:** Bend, twist, or shift low back, especially when sitting back on heels and when leaning forward to place hands on floor and sliding forward into the quadruped position

**Key Principle:** Do not bend, twist, or shift low back

---

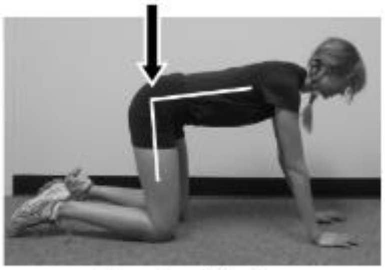

Correct positioning

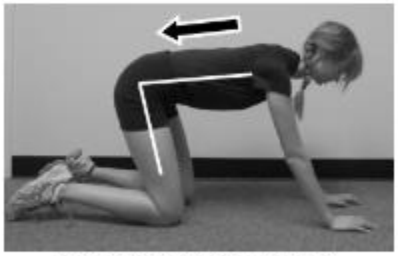

Correct positioning – weight shifted back towards heels

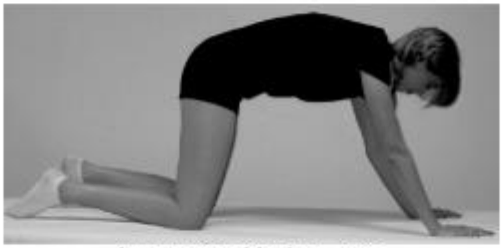

Incorrect positioning – note bending in low back

**Lumbar Flexion Rotation Skill: Positioning – quadruped**

© 2020 van Dillen LR et al. *JAMA Neurology*.

**Do:**

- Position yourself so shoulders are directly over hands, and hips are directly over knees, keeping back straight (top left picture)
- Evenly distribute weight between both hands and knees
- If your therapist has advised you to do so, shift weight back towards your heels (top right picture)

**Do NOT:** Bend, twist, or shift your low back (bottom picture)
**Key Principle:** Do not bend, twist, or shift low back

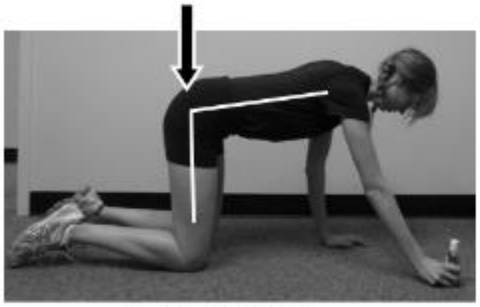

Correct positioning

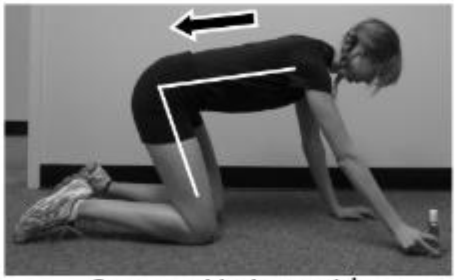

Correct positioning – weight shifted back towards heels

Incorrect positioning – note twisting in low back

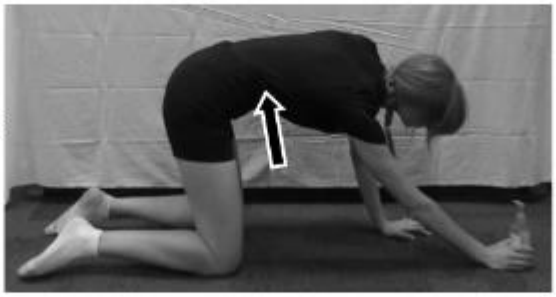

**Lumbar Flexion Rotation Skill: Moving object in quadruped**

**Do:**

- If your therapist has advised you to do so, shift weight back towards your heels (top right picture)
- Shift weight onto one arm and leg, moving body as a unit
- Easily contract abdominals without causing your low back to bend, then lift unweighted arm off floor to reach for object (top left picture)
- Pick up object and move it as necessary
- After replacing object, return hand to its original position and shift weight evenly back onto both hands and knees

**Do NOT:** Bend, twist, or shift low back when reaching for object (bottom picture)
**Key Principle:** Do not bend, twist, or shift low back

---

© 2020 van Dillen LR et al. *JAMA Neurology*.

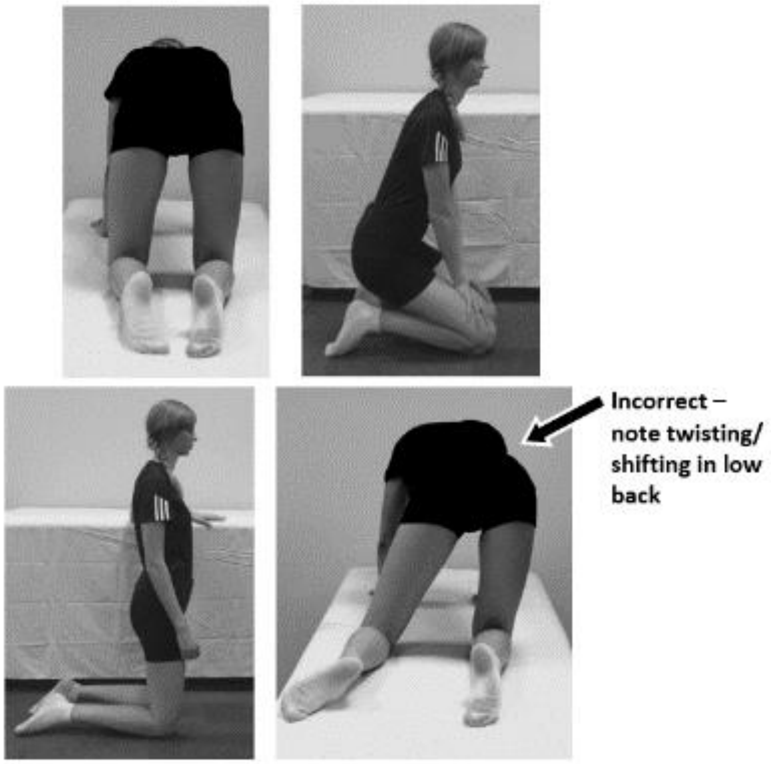


**Lumbar Flexion Rotation Skill: Quadruped to standing – step 1 (quadruped to kneeling)**

**Do:**

- Push back evenly with hands (top left picture), sliding hands back and bending hips and knees to sit back on your heels (top right picture)
- Straighten hips using leg muscles to get body into full kneeling (bottom left picture)

**Do NOT:**

- Push back unevenly with hands, as this would result in twisting/shifting in your low back (bottom right picture)
- Bend low back

**Key Principle:** Do not bend, twist, or shift low back

© 2020 van Dillen LR et al. *JAMA Neurology*.

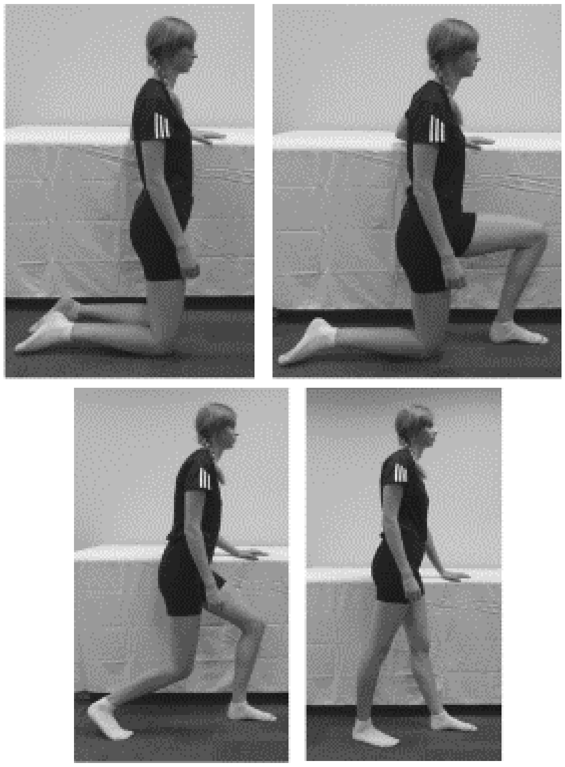

**Lumbar Flexion Rotation Skill: Quadruped to standing – step 2 (kneeling to standing)**

**Do:**

- Hold onto something for support, if available (top left picture)
- Bring one leg forward so foot is on ground (top right picture)
- From this position, push up to standing keeping low back straight (bottom pictures)

**Do NOT:** Bend, twist, or shift low back, especially when bringing your leg forward to put your foot on the ground

**Key Principle:** Do not bend, twist, or shift low back

© 2020 van Dillen LR et al. *JAMA Neurology*.

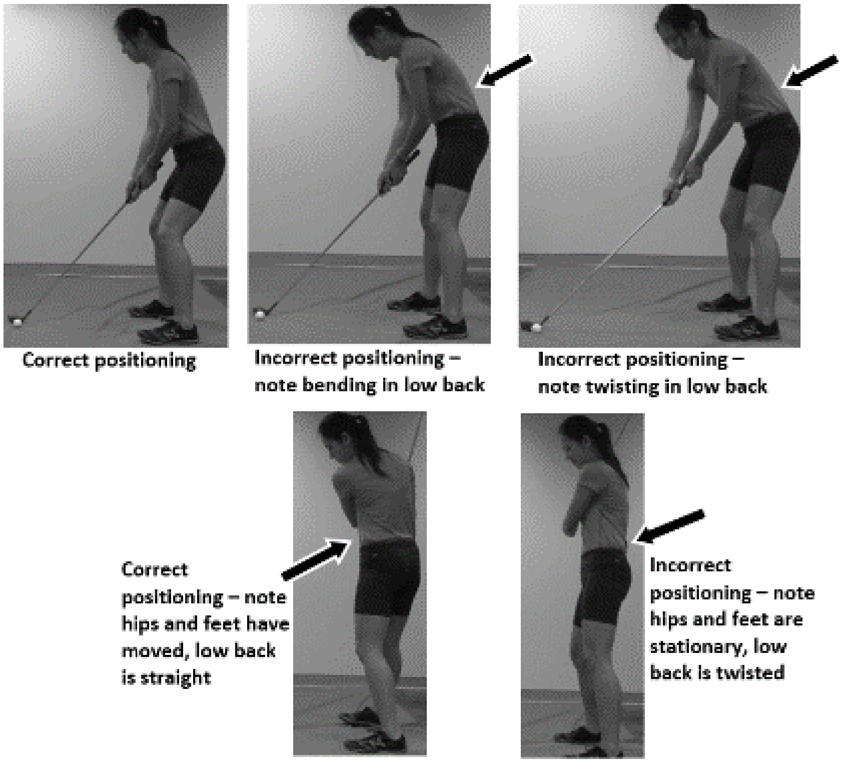


**Lumbar Flexion Rotation Skill: Golf – swinging club**

**Do:**

- Keep your back straight when standing next to the ball (top left picture)
- Allow your hips and feet to move (bottom left picture)

**Do NOT:**

- Bend, twist, or shift your low back (top middle and right pictures)
- Keep your feet stationary (bottom right picture)

**Key Principle:** Do not bend, twist, or shift low back

© 2020 van Dillen LR et al. *JAMA Neurology*.

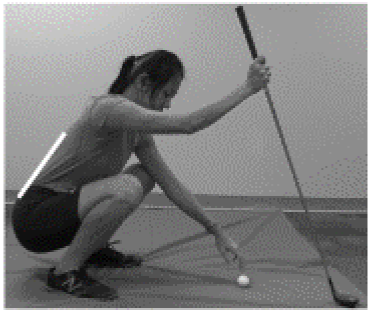

Correct positioning

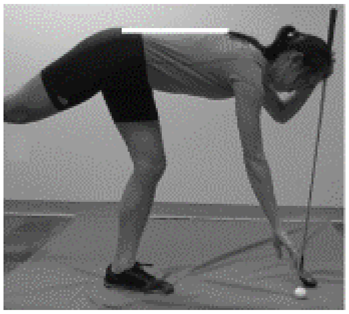

Correct positioning

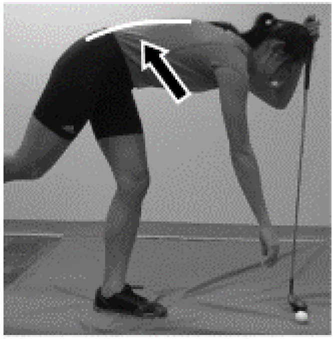

Incorrect positioning – note bending in low back

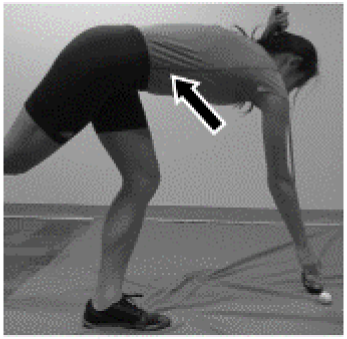

Incorrect positioning – note twisting in low back

**Lumbar Flexion Rotation Skill: Golf – picking up ball**

**Do:**

- Pick up ball by bending hips and knees (top left picture)
- You can also pick up the ball by lifting one leg up and back behind you, holding on to your golf club for support. Make sure to keep your back straight (top right picture)

**Do NOT:** Bend, twist, or shift low back (bottom pictures)

**Key Principle:** Do not bend, twist, or shift low back

---

© 2020 van Dillen LR et al. *JAMA Neurology*.

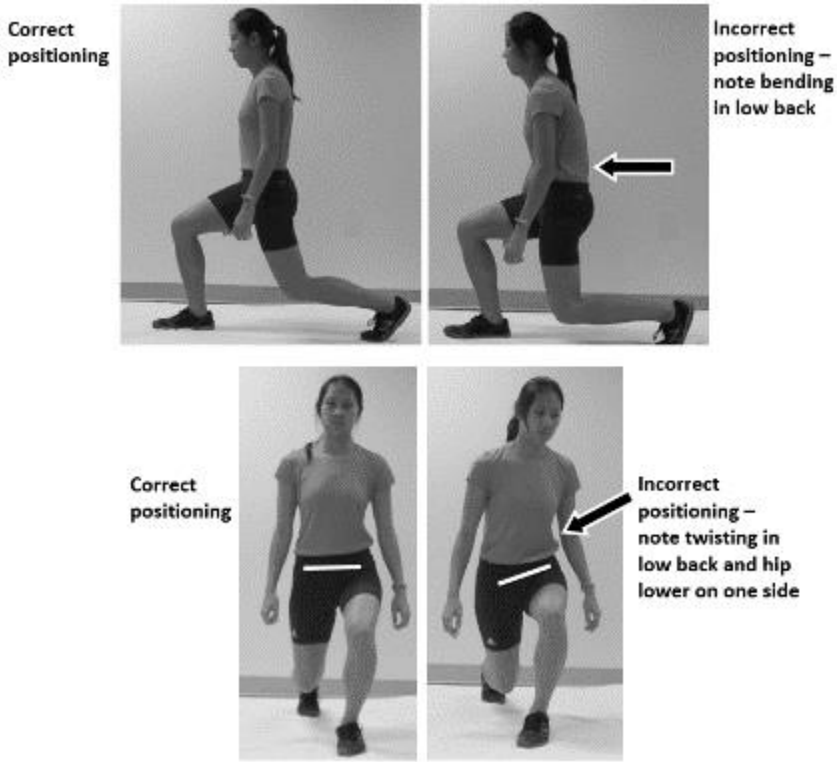


**Lumbar Flexion Rotation Skill: Lunge**

**Do:**

- Keep your chest and shoulders up and in line with your hips (left pictures)
- Keep your hips level (bottom left picture)

**Do NOT:**

- Bend, twist, or shift low back (right pictures)
- Let your hip drop on one side (bottom right picture)

**Key Principle:** Do not bend, twist, or shift low back

© 2020 van Dillen LR et al. *JAMA Neurology*.

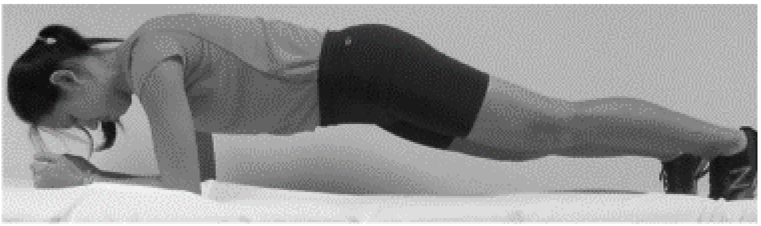

Correct positioning

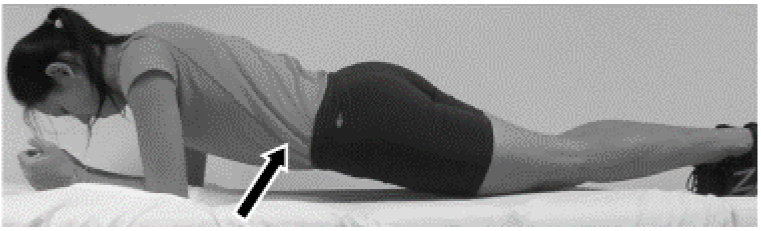

Incorrect positioning – note twisting in low back

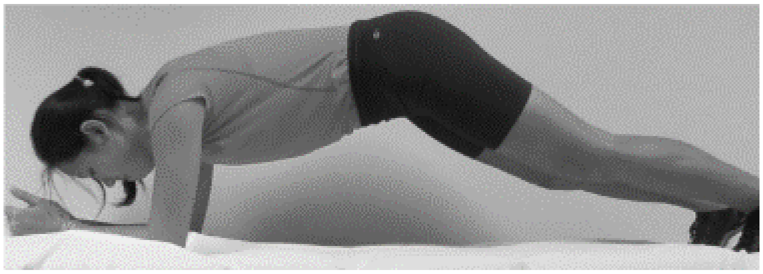

Incorrect positioning – note buttocks are up in the air

**Lumbar Flexion Rotation Skill: Plank position**

**Do:** Keep your back straight and your whole body in line (top picture)

**Do NOT:**

- Bend, twist, or shift low back (middle picture)
- Bend your hips so your buttocks are up in the air (bottom picture)

**Key Principle:** Do not bend, twist, or shift low back

---

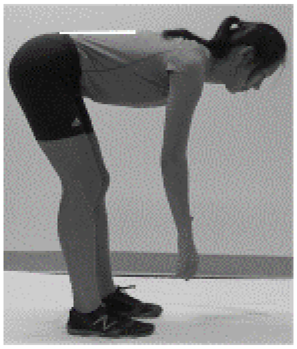

Correct positioning

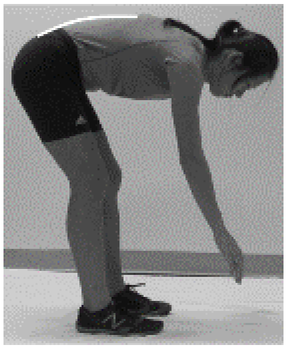

Incorrect positioning – note bending in low back

**Lumbar Flexion Rotation Skill: Bending over**

**Do:**

- Bend in your hips and keep your back straight (left picture)
- When you come back up from bending over, keep your back straight and use your buttock muscles to bring your hips/buttocks underneath you

**Do NOT:** Bend, twist, or shift low back (right picture)

**Key Principle:** Do not bend, twist, or shift low back

---

© 2020 van Dillen LR et al. *JAMA Neurology*.

**Strength and Flexibility Exercise Handouts**
**Trunk Flexibility Phase 1**

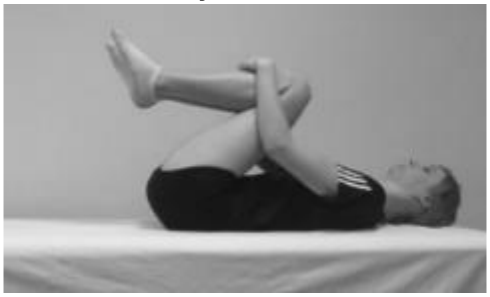

**Activity: Lower Back Flexion in Back Lying**

**Position:** Lie on your back on the floor or bed.

**Movement:** Pull your knees up to your chest.  Grip your knees with your hands and gently pull your knees further toward your chest.

**Key Concept:** Stretch low back.

---

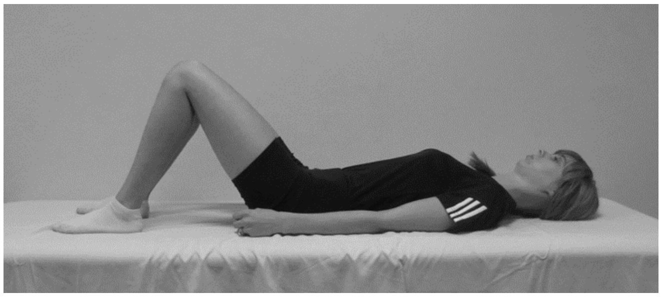

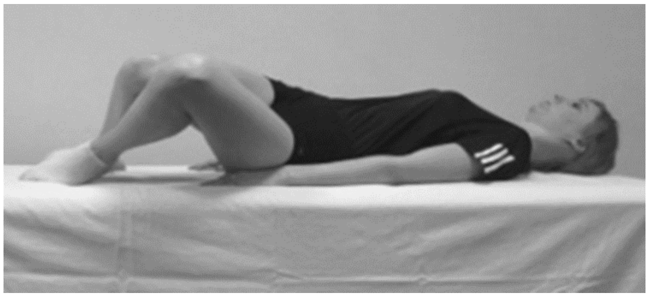

**Trunk Flexibility Phase 1**
**Activity: Trunk Rotation in Back Lying with Hips and Knees Flexed**
**Position:** Lie on your back with your hips and knees bent, your feet flat on the floor or bed, and your legs together (top picture).
**Movement:** Slowly roll your knees to one side keeping your upper trunk still (bottom picture). Return to the starting position.  Repeat, rolling your knees to the other side.
**Key Concept:** Stretch low back.

---

© 2020 van Dillen LR et al. *JAMA Neurology*.

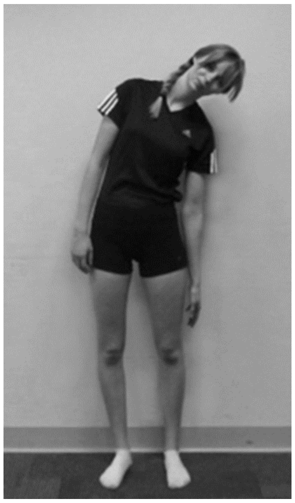

**Trunk Flexibility Phase 1**
**Activity: Trunk Side Bend in Standing**
**Position:** Stand with your feet shoulder width apart and your trunk against a wall.
**Movement:** Bend your trunk to one side moving from the waist. As you side bend, do not allow your trunk to bend forward, backward or twist. Repeat to the other side.
**Key Concept:** Stretch side of trunk.

---

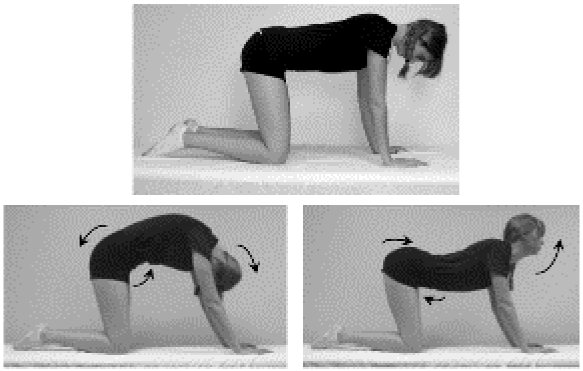

**Trunk Flexibility Phase 1**
**Activity: Trunk Flexion/Extension in Hands and Knees**
**Position:** Assume the hands and knees position with your hands positioned directly under your shoulders and your knees under your hips (top picture).
**Movement:** First, lower your head and round your back, tucking your tail bone under (bottom left picture). Second, let your low back sag toward the floor as you roll your tailbone and head upward (bottom right picture).
**Key Concept:** Stretch low back.

---

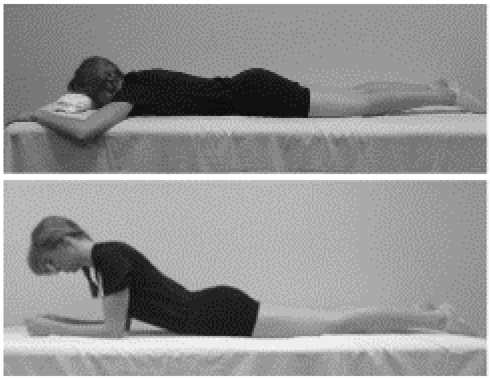

© 2020 van Dillen LR et al. *JAMA Neurology*.

**Trunk Flexibility Phase 1**
**Activity: Trunk Extension in Face Lying**
**Position:** Lie on your stomach with your elbows and forearms at a 90 degree angle (top picture).
**Movement:** Arch the small of your back by pressing your pelvis and stomach to the floor as you roll your head upward. Move yourself up onto your elbows (bottom picture).
**Key Concept:** Stretch low back.

---

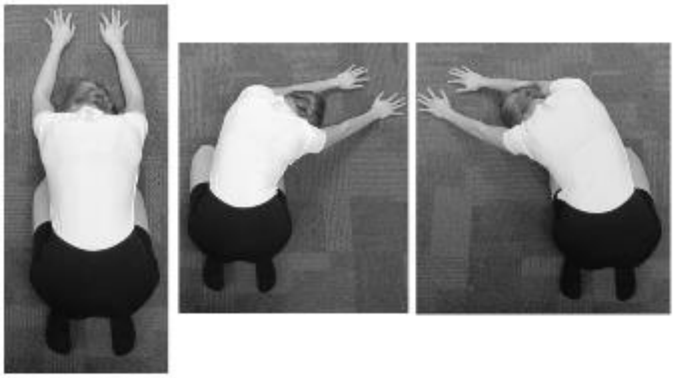

**Trunk Flexibility Phase 1**
**Activity: Trunk Side Stretch**
**Position:** Sit back on your heels with your arms out in front of you (left picture).
**Movement:** Move your hands slowly to the right side until you feel a stretch on your left side (middle picture). Return to the starting position. Repeat to the other side (right picture).
**Key Concept:** Stretch side of trunk.

---

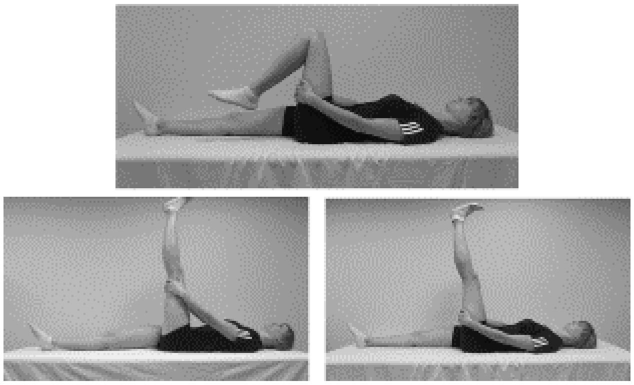

**Extremity Flexibility Phase 1**
**Activity: Hamstring/Calf Muscle Stretch**
**Position:** Lie on your back on the floor or bed. Bend your hip to 90 degrees with your knee remaining relaxed (top picture). Keep your other leg flat on the floor or bed.
**Movement:** Place your hands behind your leg in a position that is comfortable for you, trying to grab as close to your knee as you can. Actively straighten your knee using the muscles in the front of your thigh (bottom left picture). Next, bring your toes towards your trunk (bottom right picture). You can use a towel behind your knee to assist in holding the leg if you cannot reach your leg. Repeat with the opposite leg.
**Key Concept:** Stretch back of leg and calf.

---

© 2020 van Dillen LR et al. *JAMA Neurology*.

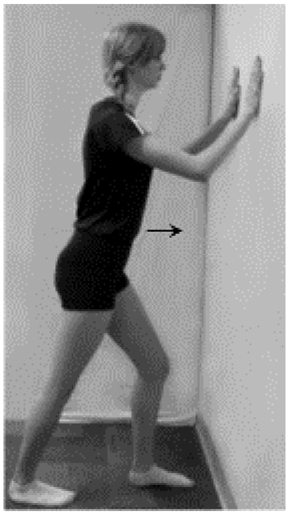

**Extremity Flexibility Phase 1**
**Activity: Calf Muscle Stretch in Standing**
**Position:** Position your body facing a wall. Place both hands on the wall to support you.
**Movement:** Point your toes directly towards the wall and hold your heels down. Lean into the wall by bending the knee of your front leg, keeping the knee of your back leg straight as shown above (see arrow). You should feel a stretch in your calf muscle, located at the back of your lower leg. Repeat on the other side.
**Key Concept:** Stretch back of calf.

---

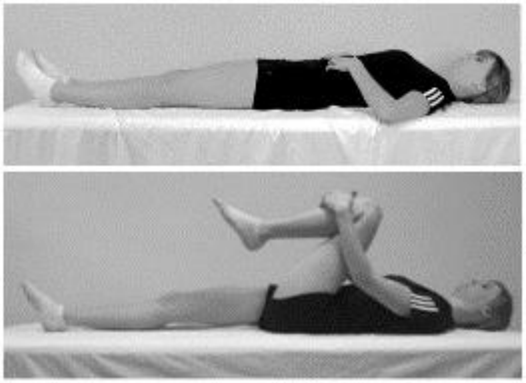

**Extremity Flexibility Phase 1**
**Activity: Gluteal Muscle Stretch in Back Lying**
**Position:** Lie on your back with your legs straight (top picture).
**Movement:** Bend the leg to be stretched towards your chest. Hold onto your knee with both hands. Gently pull your knee towards your chest with your hands, keeping your head on the floor or bed and keeping the opposite leg straight (bottom picture). You should feel a stretch in the back of your buttock of the bent leg. You may also feel a stretch in the front of your thigh on the opposite leg.
**Key Concept:** Stretch back of hip.

---

© 2020 van Dillen LR et al. *JAMA Neurology*.

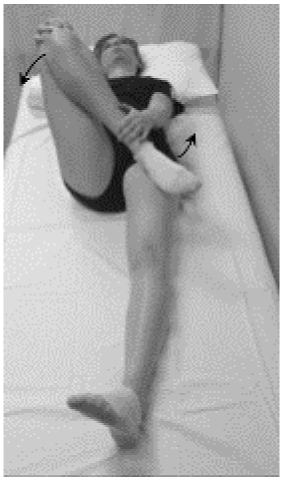

**Extremity Flexibility Phase 1**
**Activity: Piriformis Muscle Stretch in Back Lying**
**Position:** Lie on your back holding your knee and ankle as shown.
**Movement:** Pull your knee toward your chest. Then pull your ankle toward your chest so that you feel a stretch in your buttock. Keep the opposite leg straight.
**Key Concept:** Stretch back of hip.

---

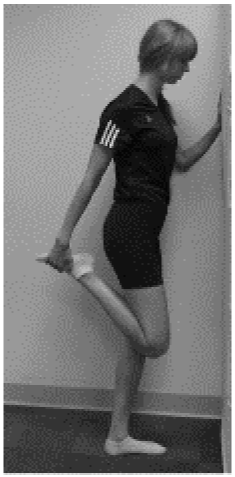 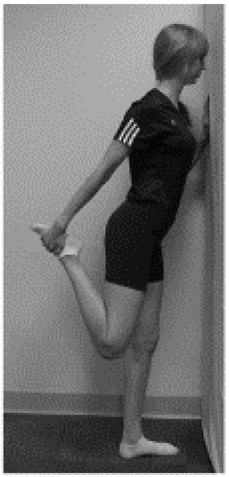

**Extremity Flexibility Phase 1**
**Activity: Quadriceps Muscle Stretch in Standing**
**Position:** Stand facing a wall with your left hand on the wall to support your body weight.
**Movement:** Bend your right knee, and grip your ankle (left picture). Make sure your knee is pointed forward and not toward or away from your other knee. Pull your right leg back (right picture) while maintaining your spinal alignment.
Repeat with the opposite leg.
**Key Concept:** Stretch front of thigh.

---

© 2020 van Dillen LR et al. *JAMA Neurology*.

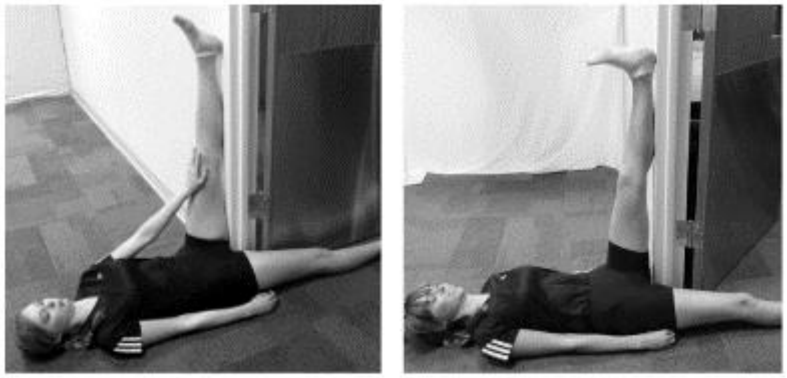

**Extremity Flexibility Phase 1**
**Activity: Hamstring/Calf Muscle Stretch – Alternative #1**
**Position:** Lie on your back with your leg supported on a wall next to a doorway (left picture). Lie as close to the base of the doorway as possible while keeping your legs straight.
**Movement:** Next, bring your toes towards your trunk (right picture). Keep the opposite leg straight on the floor.
Repeat with the opposite leg.
**Key Concept:** Stretch back of leg and calf.

---

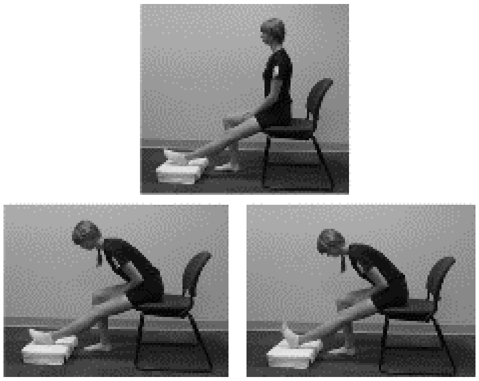

**Extremity Flexibility Phase 1**
**Activity: Hamstring/Calf Muscle Stretch – Alternative #2**
**Position:** Sit with your leg supported on the floor or on a low stool (top picture).
**Movement:** Relax, letting your knee straighten. Lean your trunk forward from your hips (bottom left picture) while keeping your back straight. Pull your toes toward your trunk (bottom right picture) while maintaining the knee and trunk position.
**Key Concept:** Stretch back of leg and calf.

---

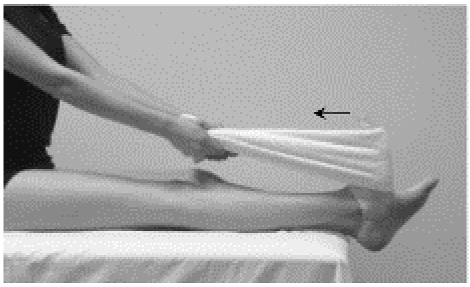

**Extremity Flexibility Phase 1**
**Activity: Calf Muscle Stretch – Alternative**
**Position:** Sit with one leg straight out in front of you. Wrap a towel or sheet around your foot.
**Movement:** Gently pull the towel/sheet toward you, keeping your knee straight. You should feel a stretch in your calf muscle, located at the back of your lower leg.

© 2020 van Dillen LR et al. *JAMA Neurology*.

**Key Concept:** Stretch back of calf.

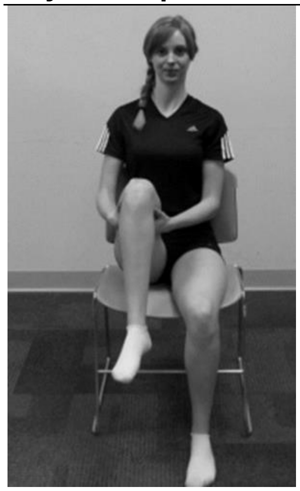

**Extremity Flexibility Phase 1**
**Activity: Gluteal Muscle Stretch – Alternative**
**Position:** Sit in a chair with your back supported.
**Movement:** Place your hands underneath your thigh and bring your knee towards your chest. Keep your back straight and your head up.
**Key Concept:** Stretch back of hip.

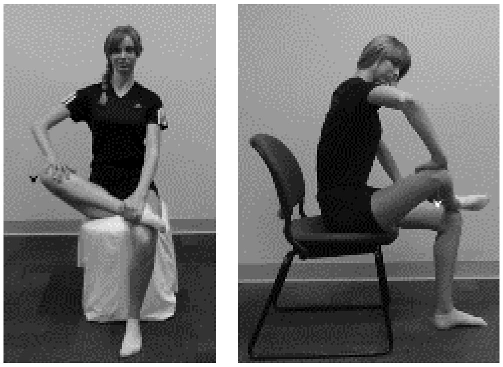

Front View    Side View

**Extremity Flexibility Phase 1**
**Activity: Piriformis Muscle Stretch – Alternative**
**Position:** Sit in a chair with your foot on the opposite knee.
**Movement:** Lean your trunk forward from your hips, keeping your back straight, and gently push your knee towards the floor.
**Key Concept:** Stretch back of hip.

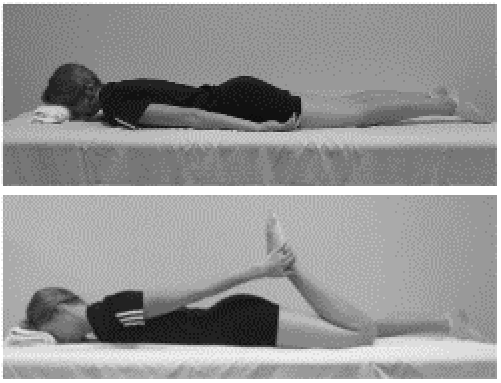

**Extremity Flexibility Phase 1**

© 2020 van Dillen LR et al. *JAMA Neurology*.

**Activity: Quadriceps Muscle Stretch – Alternative #1**
**Position:** Lie on your stomach as shown above (top picture). Place a 2 inch towel roll under your forehead or turn your head to either side, whatever direction is most comfortable for you.
**Movement:** Bend your knee and bring your foot toward your buttock (bottom picture). Grasp your ankle to bend the knee fully, but do not lift your thigh off the floor or bed. You should feel a stretch in the front of your thigh. If advised by your therapist, you may use a towel to assist with the knee bend motion.
**Key Concept:** Stretch front of thigh.

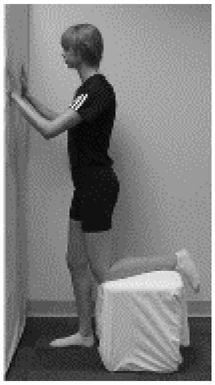

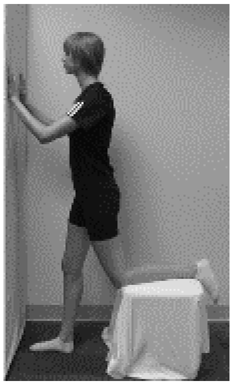

**Extremity Flexibility Phase 1**
**Activity: Quadriceps Muscle Stretch – Alternative #2**
**Position:** Stand facing a wall with one knee bent slightly and the other leg supported on a stationary stool (left picture). Extend your arms and place hands on wall for support.
**Movement:** Shift your weight forward (right picture), stretching the muscle in the front of your thigh. Maintain your spinal alignment.
**Key Concept:** Stretch front of thigh.

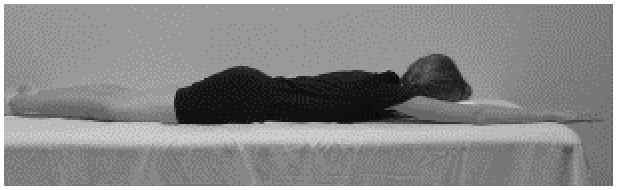

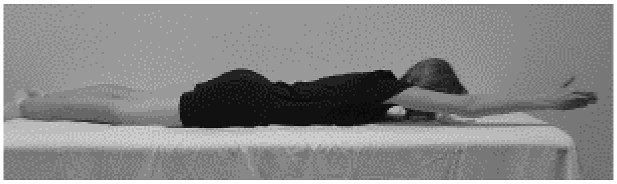

**Trunk Strength Phase 1**
**Activity: Single Arm Lift in Face Lying**
**Position:** Lie on your stomach with one arm overhead and your other arm by your side (top picture). Place a 2 inch towel roll under your forehead or turn your head to either side, whatever direction is most comfortable for you. ***If instructed by your therapist, place a pillow under your chest to allow room to move your arm.
**Movement:** Turn your thumb up and raise your arm upward (bottom picture). Lift your arm only as high as instructed by your therapist.
**Key Concept:** Strengthen low back.

© 2020 van Dillen LR et al. *JAMA Neurology*.

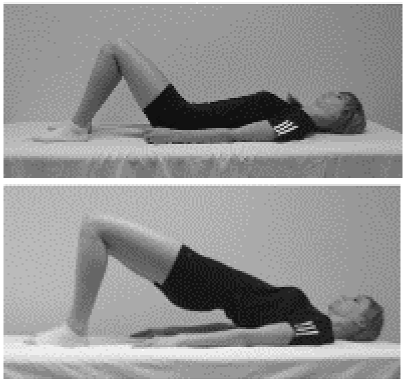

**Trunk Strength Phase 1**
**Activity: Bridging**
**Position:** Lie on your back with your hips and knees bent and your feet flat on the floor or bed (top picture).
**Movement:** Squeeze your buttocks together and lift your buttocks off the floor (bottom picture). Return to the starting position.
**Key Concept:** Strengthen low back.

---

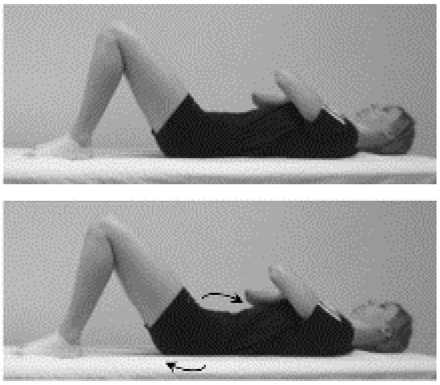

**Trunk Strength Phase 1**
**Activity: Posterior Pelvic Tilt**
**Position:** Lie on your back with your knees bent to about 50 degrees. You can put your hands on your stomach or across your chest (top picture).
**Movement:** Tighten your stomach muscles and flatten your back into the table (bottom picture).
**Key Concept:** Strengthen abdominals.

---

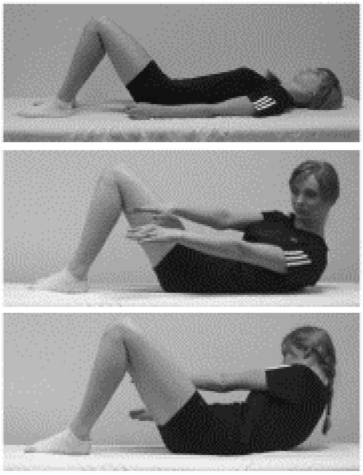

**Trunk Strength Phase 1**

© 2020 van Dillen LR et al. *JAMA Neurology*.

**Activity: Trunk Curl with Rotation – Arms by Side**
**Position:** Lie on your back with your hips and knees bent, your feet flat on the floor or bed, and your arms at your sides (top picture).
**Movement:** Raise your head and shoulders while curling your trunk upward and to the side (middle picture). Keep your chin tucked in. Only curl up as far as instructed by your therapist. Repeat to the other side (bottom picture).
**Key Concept:** Strengthen abdominals.

---

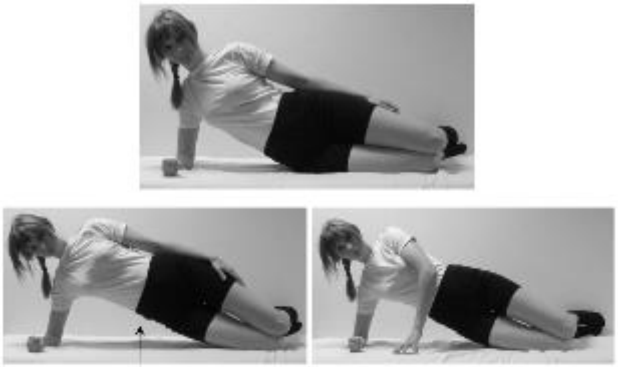

**Trunk Strength Phase 1**
**Activity: Side Plank on Elbow and Knee**
**Position:** Lie on your side, propped up on one elbow with your knees bent to 90 degrees (top picture). Make sure your shoulders and knees are in line with each other.
**Movement:** Lift your hips up off the surface so your body is in a straight line (bottom left picture). ***If instructed by your therapist, place your top hand on the surface in front of you for additional support (bottom right picture). Repeat on the other side.
**Key Concept:** Strengthen low back and abdominals.

---

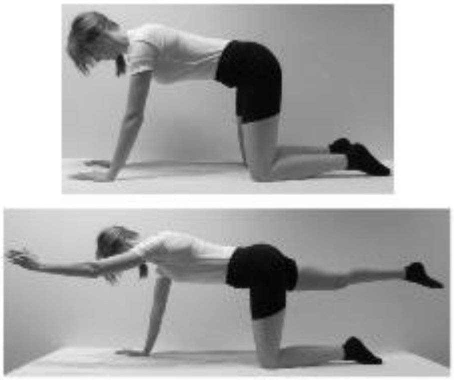

**Trunk Strength Phase 1**
**Activity: Opposite Arm and Leg Lift in Hands and Knees**
**Position:** Start in the hands and knees position (top picture).
**Movement:** Lift your left arm straight out in front of you while simultaneously straightening your right leg out behind you (bottom picture). Return to the starting position.
**Key Concept:** Strengthen low back.

---

© 2020 van Dillen LR et al. *JAMA Neurology*.

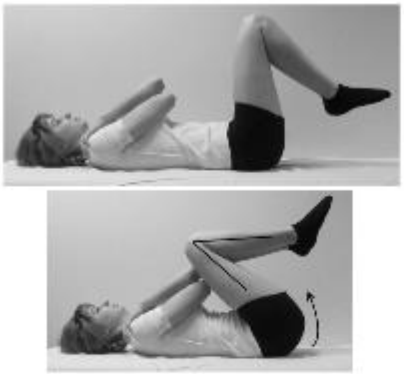

**Trunk Strength Phase 1**
**Activity: Double Knees to Chest with Legs Bent**
**Position:** Lie on your back with your legs bent (top picture).
**Movement:** Tighten your abdominal muscles and lift your buttocks off of the surface while keeping your knees bent more than 90 degrees (bottom picture). Do not use momentum to lift your buttocks off the surface.
**Key Concept:** Strengthen abdominals.

---

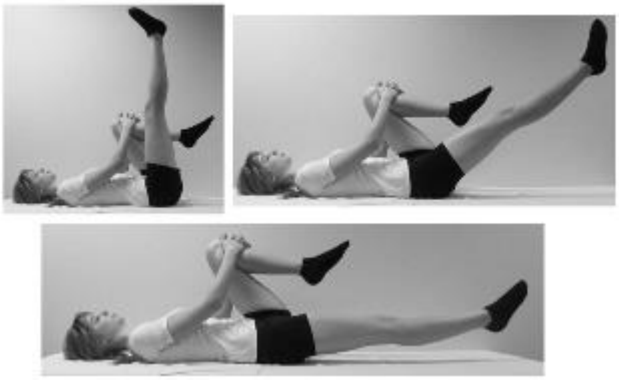

**Trunk Strength Phase 1**
**Activity: Single Leg Lowering**
**Position:** Lie on your back with one knee pulled in toward your chest and the other leg up straight (top left picture).
**Movement:** Slowly lower your straight leg down towards the surface; lower as far as your therapist instructed (top right picture, bottom picture). Return to the starting position.
**Key Concept:** Strengthen abdominals.

---

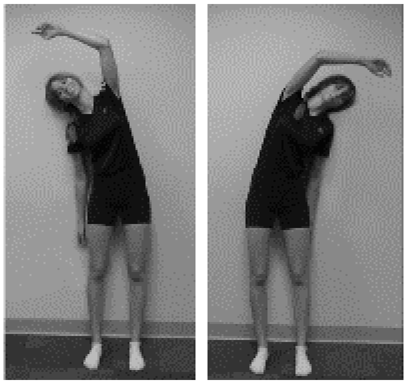

**Trunk Flexibility Phase 2**
**Activity: Trunk Side Bend in Standing – Arm Overhead**
**Position:** Stand with your feet shoulder width apart and your trunk and shoulders against a wall.

© 2020 van Dillen LR et al. *JAMA Neurology*.

**Movement:** Raise your left arm overhead and bend your trunk to the right side moving from the waist (left picture). As you side bend do not allow your trunk to bend forward, backward, or twist (i.e. keep your trunk and shoulders against the wall as much as possible).
Repeat to the opposite side raising your right arm and side bending to the left (right picture).
**Key Concept:** Stretch side of trunk.

---

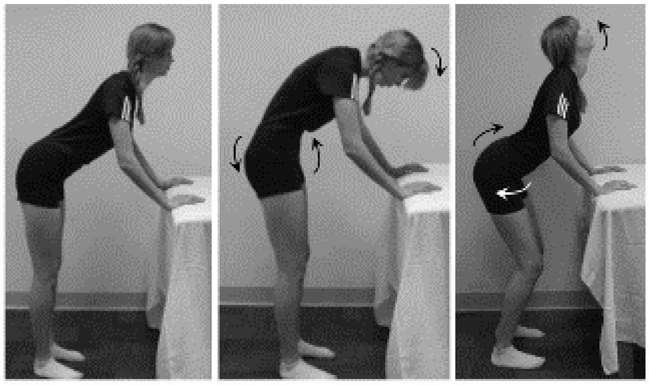

**Trunk Flexibility Phase 2**
**Activity: Trunk Flexion/Extension in Standing – Arm Support**
**Position:** Stand with your knees bent slightly and your upper body inclined forward with your hands resting on a support surface such as a table or countertop. Position your back in a straight alignment (left picture).
**Movement:** First, tighten your stomach muscles, squeeze your buttock muscles and round your lower back, tucking your tailbone under (middle picture). Second, arch your lower back while rolling your tailbone out, your head up, and your chest up and out (right picture).
**Key Concept:** Stretch low back.

---

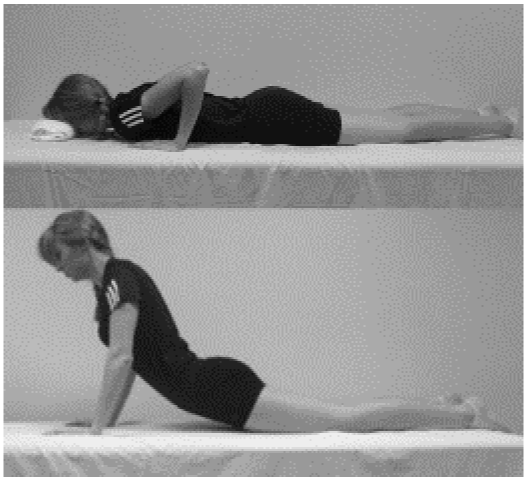

**Trunk Flexibility Phase 2**
**Activity: Trunk Extension in Face Lying – Arms Extended**
**Position:** Lie on your stomach with both hands at shoulder height and your elbows bent (top picture).
**Movement:** Straighten your elbows and lift your upper trunk as far up as you can (bottom picture). Keep your pelvis and legs relaxed. Return to lying on your stomach.
**Key Concept:** Stretch low back.

---

© 2020 van Dillen LR et al. *JAMA Neurology*.

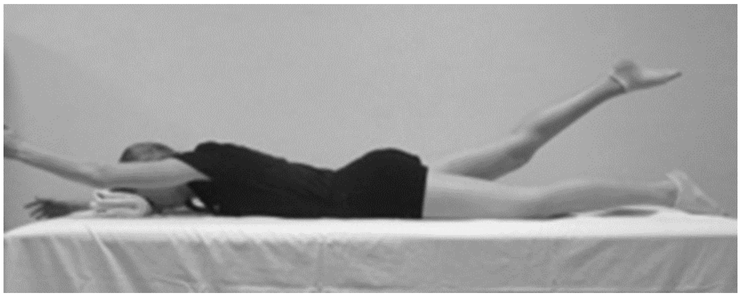

**Trunk Strength Phase 2**
**Activity: Opposite Arm and Leg Lift in Face Lying**
**Position:** Lie on your stomach as shown above (top picture). Place a 2 inch towel roll under your forehead or turn your head to either side, whatever direction is most comfortable for you. ***If instructed by your therapist, place a pillow under your chest to allow room to move your arm.
**Movement:** Squeeze your buttocks together and raise one arm and the opposite leg (bottom picture). Keep your thumb pointed up when lifting your arm and keep your arm and leg as straight as possible. Lift your arm and leg only as high as instructed by your therapist. Repeat with the opposite arm and leg.
**Key Concept:** Strengthen low back.

---

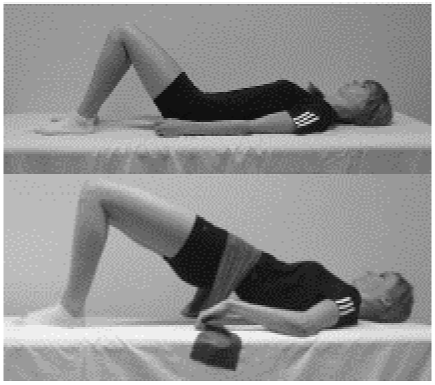

**Trunk Strength Phase 2**
**Activity: Bridging with Resistance**
**Position:** Lie on your back with your hips and knees bent and your feet flat on the floor or bed (top picture).
**Movement:** Place Theraband across your hips/pelvis and hold the band to the floor or bed with your hands. Squeeze your buttocks together and lift your buttocks off the floor or bed (bottom picture). Return slowly to the starting position.
**Key Concept:** Strengthen low back.

---

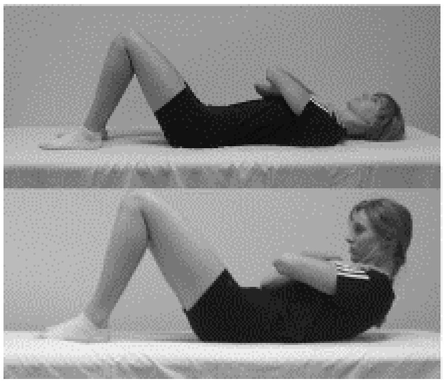

© 2020 van Dillen LR et al. *JAMA Neurology*.

**Trunk Strength Phase 2**
**Activity: Trunk Curl – Arms Crossed**
**Position:** Lie on your back with your hips and knees bent, your feet flat on the floor or bed, and your arms crossed over your chest (top picture).
**Movement:** Tighten your stomach muscles and lift your head and shoulders off the floor (bottom picture). Keep your chin tucked in. **Do not** go all the way up into a full sit up – only curl up as far as instructed by your therapist.
**Key Concept:** Strengthen abdominals.

---

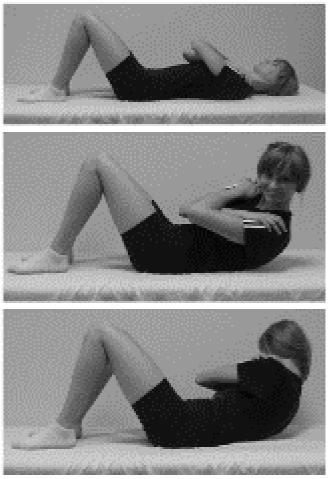

**Trunk Strength Phase 2**
**Activity: Trunk Curl With Rotation – Arms Crossed**
**Position:** Lie on your back with your hips and knees bent, your feet flat on the floor or bed, and your arms crossed over your chest (top picture).
**Movement:** Raise your head and shoulders while curling your trunk upward and to the side (middle picture). Keep your chin tucked in. **Do not** go all the way up into a full sit up – only curl up as far as instructed by your therapist. Repeat to the other side (bottom picture).
**Key Concept:** Strengthen abdominals.

---

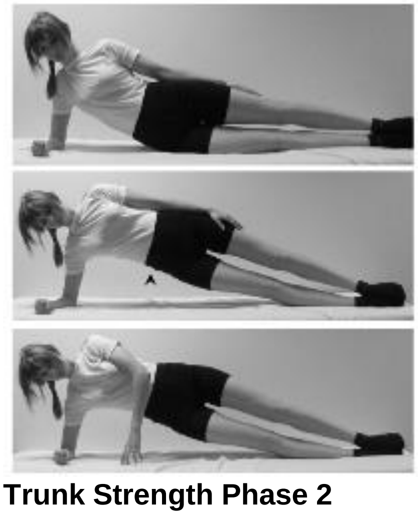

**Trunk Strength Phase 2**
**Activity: Side Plank on Elbow and Foot**
**Position:** Lie on your side, propped up on one elbow with your legs out straight (top picture). Make sure your shoulders and legs are in line with each other.

© 2020 van Dillen LR et al. *JAMA Neurology*.

**Movement:** Lift your hips up off the surface so your body is in a straight line (middle picture). ***If instructed by your therapist, place your top hand on the surface in front of you for additional support (bottom picture). Repeat on the other side.
**Key Concept:** Strengthen low back and abdominals.

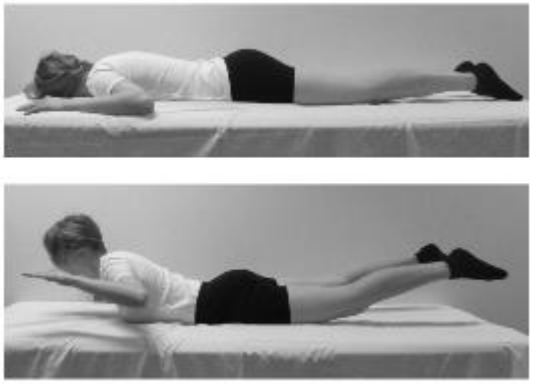

**Trunk Strength Phase 2**
**Activity: Arm and Leg Lift in Face Lying with Arms Bent**

**Position:** Lie on your stomach (top picture). You may place a towel roll under your forehead or turn your head to either side, whatever is most comfortable for you.
**Movement:** Lift your arms and legs up off the surface (bottom picture). Only lift as high as your therapist has instructed you to lift. Return to the starting position, staying in control of your movement.
**Key Concept:** Strengthen low back.

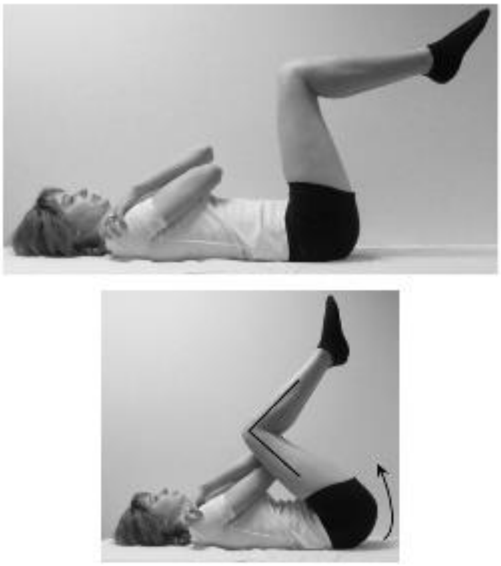

**Trunk Strength Phase 2**
**Activity: Double Knees to Chest Progression**
**Position:** Lie on your back with your legs bent (top picture).
**Movement:** Tighten your abdominal muscles and lift your buttocks off of the surface, making sure to keep your knees bent to about 90 degrees (bottom picture). Do not use momentum to lift your buttocks off the surface.
**Key Concept:** Strengthen abdominals.

© 2020 van Dillen LR et al. *JAMA Neurology*.

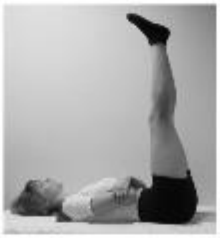
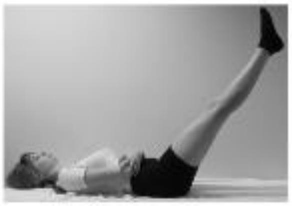
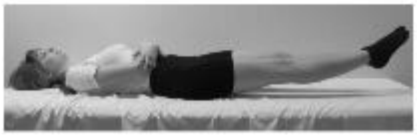

**Trunk Strength Phase 2**
**Activity: Bilateral Leg Lowering**
**Position:** Lie on your back with both legs up straight and your arms across your stomach/chest (top left picture).
**Movement:** Slowly lower both legs toward the surface; lower as far as your therapist instructed (top right picture, bottom picture). Return to the starting position.
**Key Concept:** Strengthen abdominals.

---

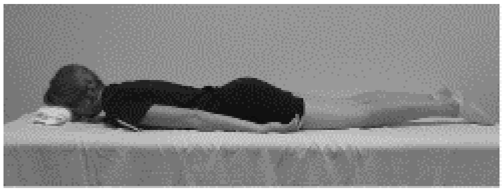
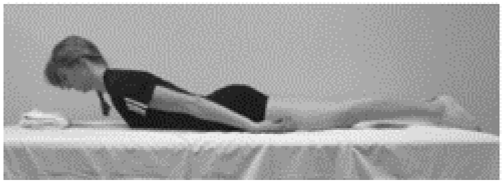

**Trunk Strength Phase 3**
**Activity: Active Trunk Extension in Face Lying**
**Position:** Lie on your stomach with your arms by your side (top picture). Place a 2 inch towel roll under your forehead or turn your head to either side, whatever direction is most comfortable for you.
**Movement:** Squeeze your buttocks, lift your upper trunk, and arch your low back (bottom picture).
**Key Concept:** Strengthen low back.

---

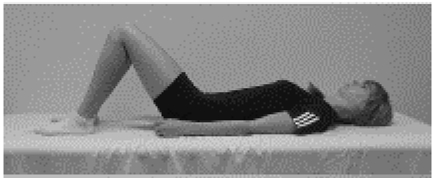
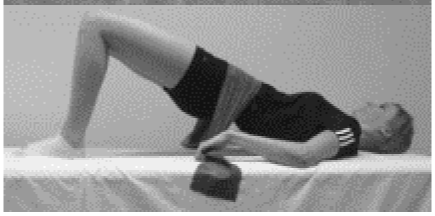

**Trunk Strength Phase 3**

© 2020 van Dillen LR et al. *JAMA Neurology*.

**Activity: Bridging with Increased Resistance**
**Position:** Lie on your back with your hips and knees bent and your feet flat on the floor or bed (top picture).
**Movement:** Place Theraband* across your hips/pelvis and hold the band to the floor or bed with your hands.  Squeeze your buttocks together and lift your buttocks off the floor or bed (bottom picture).  Return slowly to the starting position.
*Theraband strength should be increased relative to Phase 2.
**Key Concept:** Strengthen low back.

---

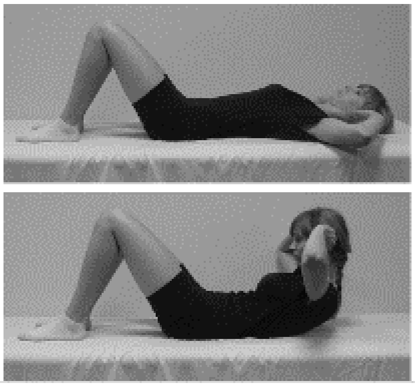

**Trunk Strength Phase 3**
**Activity: Trunk Curl – Hands Next to Head**
**Position:** Lie on your back with your hips and knees bent, your feet flat on the floor or bed, and your fingers just behind your ears with your elbows out (top picture).
**Movement:** Tighten your stomach muscles and, without pulling your head with your hands, raise your head and shoulders off the floor (bottom picture).  **Do not** go all the way up into a full sit up – only curl up as far as instructed by your therapist.
**Key Concept:** Strengthen abdominals.

---

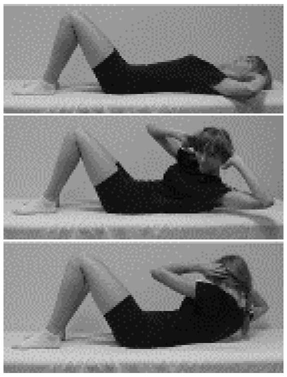

**Trunk Strength Phase 3**
**Activity: Trunk Curl with Rotation – Hands Next to Head**
**Position:** Lie on your back with your hips and knees bent, your feet flat on the floor or bed, and your fingers just behind your ears with your elbows out (top picture).
**Movement:** Without pulling your head with your hands, raise your head and shoulders and curl your trunk upward and to the side (middle picture).  **Do not** go all the way up into a full sit up – only curl up as far as instructed by your therapist.  Repeat to the other side (bottom picture).
**Key Concept:** Strengthen abdominals.

© 2020 van Dillen LR et al. *JAMA Neurology*.

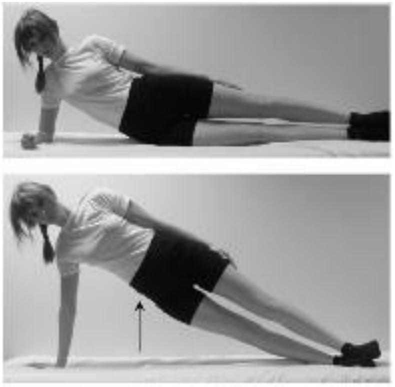

**Trunk Strength Phase 3**
**Activity: Side Plank on Hand and Foot**
**Position:** Lie on your side, propped up on one elbow with your legs out straight (top picture). Make sure your shoulders and legs are in line with each other.
**Movement:** Lift your hips up off the surface and straighten out your bottom arm so your body is in a straight line (bottom picture). If needed, use your top arm to help push yourself up.
**Key Concept:** Strengthen low back and abdominals.

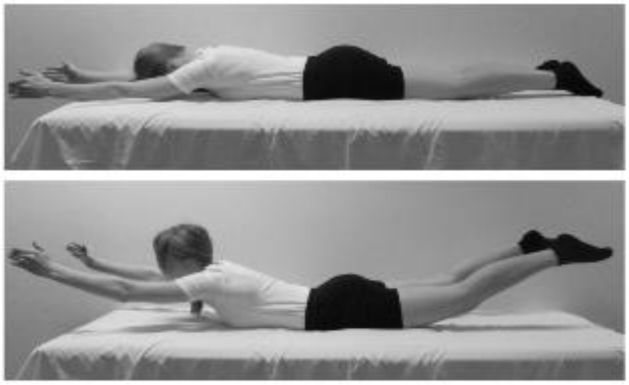

**Trunk Strength Phase 3**
**Activity: Arm and Leg Lift in Face Lying with Arms Straight**
**Position:** Lie on your stomach (top picture). You may place a towel roll under your forehead or turn your head to either side, whatever is most comfortable for you.
**Movement:** Lift your arms and legs up off the surface (bottom picture). Only lift as high as your therapist has instructed you to lift. Return to the starting position, staying in control of your movement.
**Key Concept:** Strengthen low back.

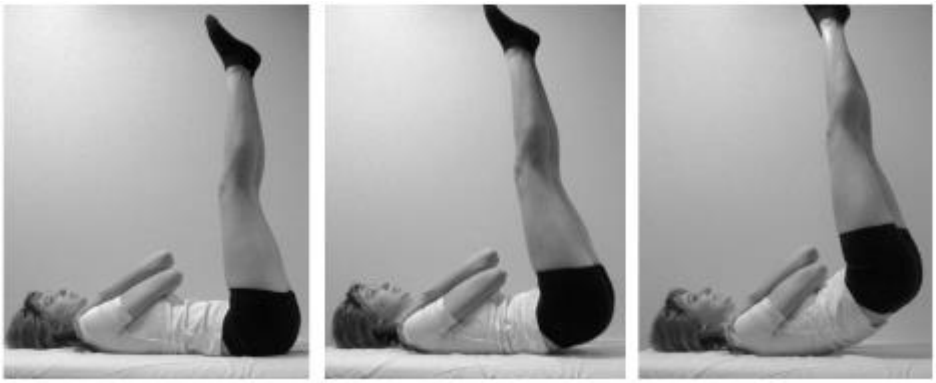

**Trunk Strength Phase 3**

© 2020 van Dillen LR et al. *JAMA Neurology*.

**Activity: Pelvic/Leg Lift**
**Position:** Lie on your back with your legs up straight (left picture).
**Movement:** Tighten your abdominal muscles and lift your legs up straight (middle and right pictures). This should result in your buttocks lifting up off the surface. Only go as high as your therapist has instructed. Keep your feet over your hips. Do not use momentum to lift your buttocks off the surface.
**Key Concept:** Strengthen abdominals.

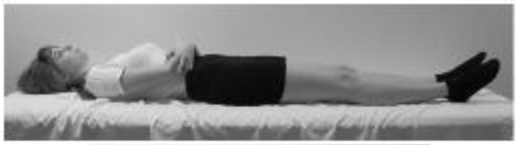

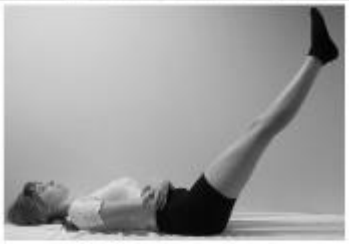

**Trunk Strength Phase 3**
**Activity: Bilateral Leg Raising**
**Position:** Lie on your back with your legs out straight and your arms across your stomach/chest (top picture).
**Movement:** Tighten your abdominal muscles and raise both legs away from the surface, lifting up as far as your therapist has instructed (bottom picture). Return to the starting position.
**Key Concept:** Strengthen abdominals.

© 2020 van Dillen LR et al. *JAMA Neurology*.

**References**


1. Fritz JM, Irrgang JJ. A comparison of a modified Oswestry Low Back Pain Disability Questionnaire and the Quebec Back Pain Disability Scale. *Phys Ther.* 2001;81(2):776-788.
2. Jensen MP, Turner JA, Romano JM. What is the maximum number of levels needed in pain intensity measurement? *Pain.* 1994;58(3):387-392.
3. Von Korff M. Studying the natural history of back pain. *Spine (Phila Pa 1976).* 1994;19(18 Suppl):2041S-2046S.
4. McGorry RW, Webster BS, Snook SH, Hsiang SM. The relation between pain intensity, disability, and the episodic nature of chronic and recurrent low back pain. *Spine (Phila Pa 1976).* 2000;25(7):834-841.
5. Ware JE, Jr., Sherbourne CD. The MOS 36-item short-form health survey (SF-36). I. Conceptual framework and item selection. *Med Care.* 1992;30(6):473-483.
6. McHorney CA, Ware JE, Jr., Raczek AE. The MOS 36-Item Short-Form Health Survey (SF-36): II. Psychometric and clinical tests of validity in measuring physical and mental health constructs. *Med Care.* 1993;31(3):247-263.
7. Patrick DL, Deyo RA, Atlas SJ, Singer DE, Chapin A, Keller RB. Assessing health-related quality of life in patients with sciatica. *Spine (Phila Pa 1976).* 1995;20(17):1899-1908.
8. Koopman C, Pelletier KR, Murray JF, et al. Stanford Presenteeism Scale: health status and employee productivity. *J Occup Environ Med.* 2002;44(1):14-20.
9. Turpin RS, Ozminkowski RJ, Sharda CE, et al. Reliability and validity of the Stanford Presenteeism Scale. *J Occup Environ Med.* 2004;46(11):1123-1133.
10. Van Dillen LR, Norton BJ, Sahrmann SA, et al. Efficacy of classification-specific treatment and adherence on outcomes in people with chronic low back pain. A one-year follow-up, prospective, randomized, controlled clinical trial. *Man Ther.* 2016;24(4):52-64.
11. Wewers ME, Lowe NK. A critical review of visual analogue scales in the measurement of clinical phenomena. *Res Nurs Health.* 1990;13(4):227-236.
12. Kleim JA, Jones TA. Principles of experience-dependent neural plasticity: Implications for rehabilitation after brain damage. *J Speech Lang Hear Res.* 2008;51(1):S225-S239.
13. Birkenmeier RL, Prager EM, Lang CE. Translating animal doses of task-specific training to people with chronic stroke in 1-hour therapy sessions: a proof-of-concept study. *Neurorehabil Neural Repair.* 2010;24(7):620-635.


© 2020 van Dillen LR et al. *JAMA Neurology*.
