# Supplementary material for: Effect of Motor Skill Training in Functional Activities vs Strength and Flexibility Exercise on Function in People With Chronic Low Back Pain: A Randomized Clinical Trial
Source: JAMA Neurol. 2020 Dec 28;78(4):1–11. doi: 10.1001/jamaneurol.2020.4821 (PMC7770617; doi:10.1001/jamaneurol.2020.4821)
Supplement: Supplement 3. — Data Sharing Statement [file jamaneurol-e204821-s003.pdf]

Data Sharing Statement

van Dillen. Effect of Motor Skill Training in Functional Activities vs Strength and Flexibility Exercise on Function in People With Chronic Low Back Pain. *JAMA Neurol*. Published December 28, 2020. doi:10.1001/jamaneurol.2020.4821

Data
Data available: No
